# Noncovalent Interaction Effects on the Acyl Radical Reaction of Bicyclo[2.2.2]octanone: Selective Formation of Rearrangement and Cyclization Products

**DOI:** 10.1021/acs.orglett.5c01574

**Published:** 2025-07-25

**Authors:** Chih-Ming Chen, Tung-Chun Kuo, Julakanti Satyanarayana Reddy, Yi Ning Teoh, Jyun-Sian Huang, Sheng-Kuo Lin, Mu-Jeng Cheng, Hsing-Pang Hsieh

**Affiliations:** † Institute of Biotechnology and Pharmaceutical Research, 50115 National Health Research Institutes, Miaoli County 350, Taiwan, ROC; ‡ Department of Chemistry, 34912 National Cheng Kung University, Tainan 701, Taiwan, ROC; § Department of Chemistry, National Tsing Hua University, Hsinchu 300, Taiwan, ROC; ∥ Biomedical Translation Research Center, Academia Sinica, Taipei City 115, Taiwan, ROC

## Abstract

The influence of allylic substituent orientation on the six-membered fused ring, as well as the steric hindrance at the alkene bridge (R^1^ and R^2^) and bridgehead (R^3^), on the thiol-mediated acyl radical rearrangement/cyclization of bicyclo[2.2.2]­octenone was investigated. A combined analysis using density functional theory calculations and interaction region indicator analysis suggests that reaction selectivity is driven by functional group noncovalent interactions.

## Graphical abstract

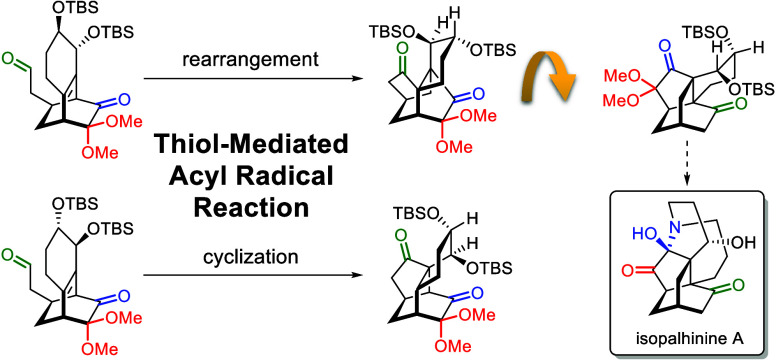

Recently, we reported a ring-strain-influenced radical rearrangement/cyclization reaction during a thiol-mediated acyl radical reaction of bicyclo[2.2.2]­octenone **1** (Scheme 1).^1,2^ Our study revealed that ring strain plays a critical role in determining whether the reaction yields a rearrangement product **RP1** or a cyclization product **CP1**. Among fused rings of different sizes, we found that only the six-membered fused ring favors the formation of the rearranged product **RP1**, owing to its lower ring strain compared to its cyclization counterpart. This unexpected discovery inspired the development of an atom-economic synthetic strategy for isopalhinine A.^3,4^

**Scheme 1:**
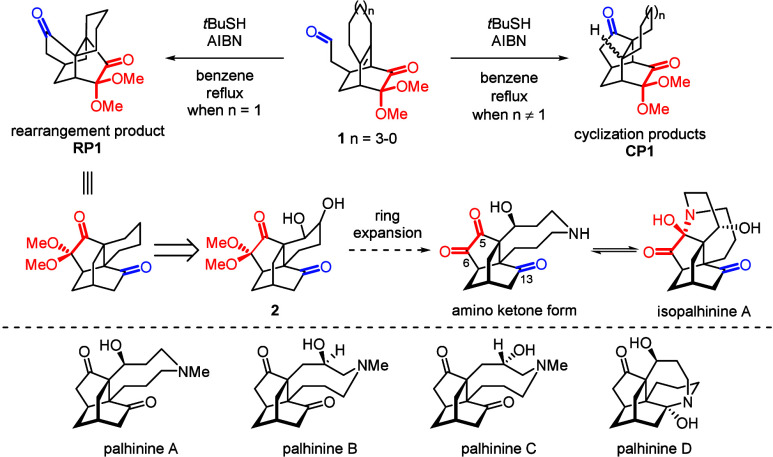
Thiol-Mediated Acyl Radical Rearrangement/Ring Expansion Strategy for the Total Synthesis of Isopalhinine A

The distinguishing feature of isopalhinine A, compared to other palhinines (as shown below in Scheme 1),^5^ is the presence of a dione moiety at C5 and C6 in the amino ketone form, which facilitates hemiaminal formation with the secondary amine in the azonane ring. Notably, through the thiol-mediated acyl radical rearrangement reaction, the protected dione moiety (indicated in red) originally located at the six-membered ring bridge of bicyclo[2.2.2]­octenone is repositioned to the five-membered ring bridge (C5 and C6) of the isotwistane skeleton (tricyclo­[4.3.1.0^3,7^]­decane), with the C13 carbonyl group derived from the original aldehyde group (indicated in blue). To facilitate ring expansion, enabling the construction of nine-membered azonane ring from the six-membered fused ring, a functional group is required, such as diol intermediate **2**. Herein, we describe the influence of steric hindrance when substitutions were introduced not only at the cyclohexane fused ring but also directly on the bicyclo[2.2.2]­octenone skeleton, supported by theoretical calculations to elucidate the steric effects.

The bicyclo[2.2.2]­octenone radical precursors **3** and **4** were synthesized through Diels–Alder reactions of the corresponding masked *ortho*-benzoquinone,^6^ followed by homologation to extend the aldehyde group, as detailed in the Supporting Information. After the diastereomers of diol precursors **3** and **4** were obtained, both were subjected to thiol-mediated acyl radical reactions (Scheme 2). A contrasting result was observed: isomer **3** yielded the rearranged product **RP3**, whereas its counterpart **4** yielded the cyclized product **CP4** along with the related alkene product **6**. The structure of the rearranged product **RP3** was further confirmed by X-ray analysis of its desilylation product **5** (CCDC 2344716). To validate the effect of the position and orientation of hydroxyl groups, the monoallylic alcohol precursors **7** and **8** were subjected to the radical reaction. Similar results were obtained: When the hydroxyl group was oriented toward the aldehyde, the rearranged product **RP7** was obtained. In contrast, when the hydroxyl group was oriented toward the ketone, the cyclized product **CP8** and the related alkene product **9** were formed. Although we intended to further investigate the influence of the homoallylic position, the corresponding Diels–Alder reaction unfortunately failed to produce the bicyclo[2.2.2]­octenone product.

**Scheme 2:**
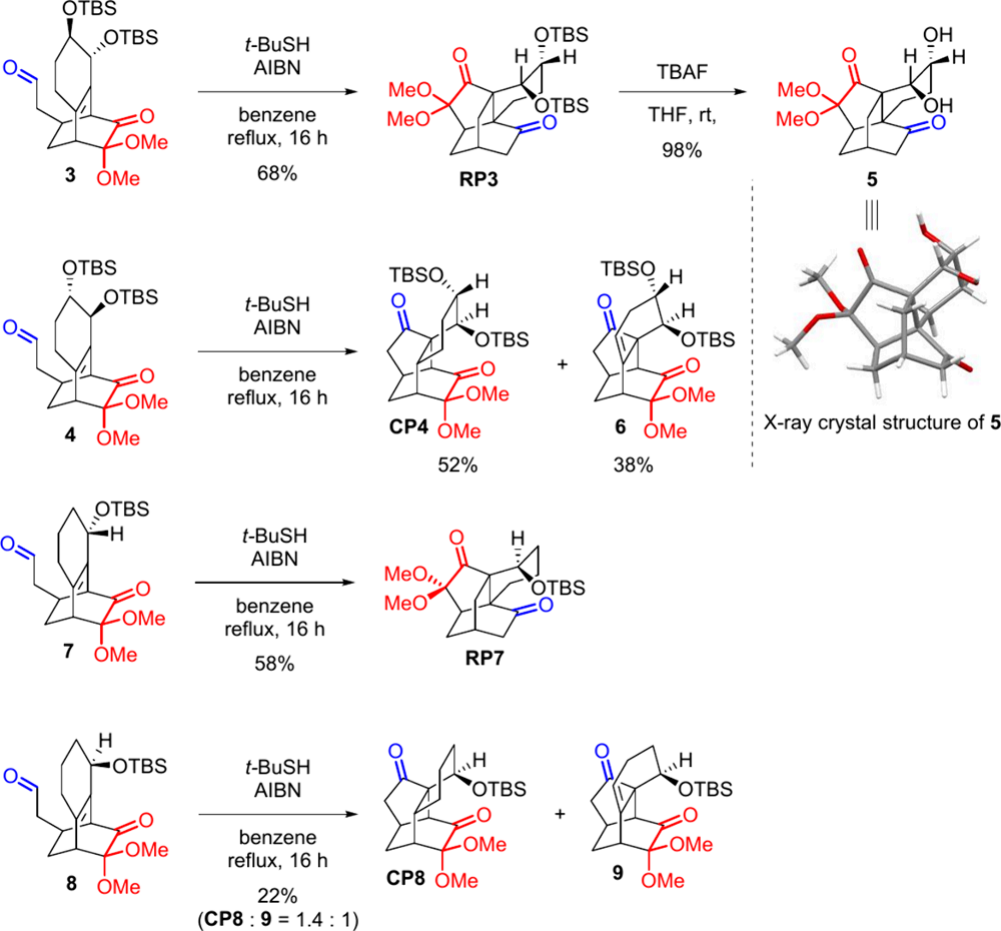
Thiol-Mediated Acyl Radical Reaction of Diol Precursors and Mono-hydroxyl Precursors

To understand why the primary products of **7** and **8** are the rearrangement and cyclization products, respectively, density functional theory (DFT) calculations were performed. In our previous study of similar systems, we found that the kinetic barriers are all readily surmounted and that the thermodynamic trends align more closely with the experimentally observed selectivity.^1^ Therefore, the present study focuses solely on thermodynamic analysis. The computational results reveal that the reaction energies (ΔE) for **7** forming the cyclization (**CP7**) and rearrangement (**RP7**) products are −13.0 and −20.8 kcal/mol, respectively, indicating a strong preference for the rearrangement product. This finding aligns with experimental observations, which show that only the rearrangement product is formed. In contrast, the ΔE values for **8** forming the cyclization (**CP8**) and rearrangement (**RP8**) products are −18.0 and −16.8 kcal/mol, respectively, suggesting a preference for the cyclization product, consistent with experimental data.

Using the two products of **8** as references, we find that changing the orientation of OTBS increases the energy of the cyclization product by 3.2 kcal/mol (**CP8** → **CP7**, Scheme 3), while it decreases the energy of the rearrangement product by 5.8 kcal/mol (**RP8** → **RP7**). Thus, altering the orientation of the OTBS group from facing the ketone to facing the aldehyde disfavors cyclization while promoting rearrangement, thereby shifting the selectivity from cyclization in compound **8** to rearrangement in compound **7**.

**Scheme 3:**
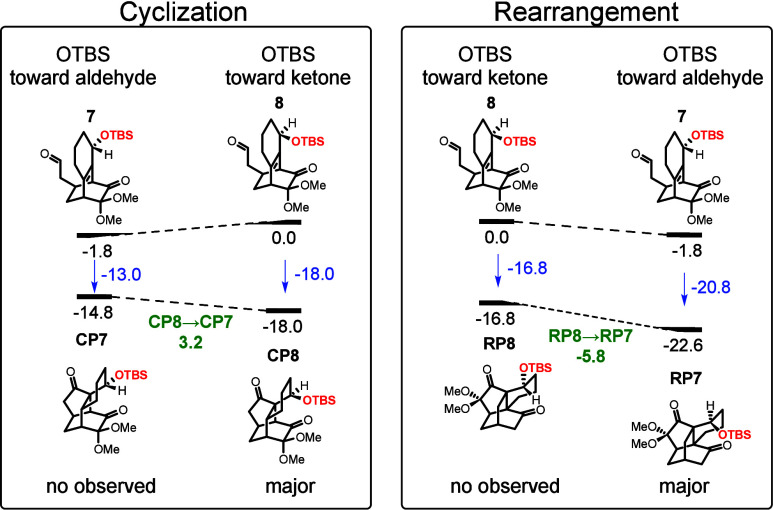
Computed Cyclization and Rearrangement Energies for Compounds **7** and **8**

To rationalize the influence of the OTBS orientation change in two cyclization products and two rearrangement products, we used the interaction region indicator (IRI) in Multiwfn^8^ to analyze the noncovalent interactions in these products (Figure 1). IRI analysis indicates that OTBS exhibits fewer attractive interactions (green areas) with the rest of the molecule in **CP7** than in **CP8**. In contrast, IRI analysis demonstrates that, relative to **RP8**, OTBS in **RP7** engages in a more pronounced interatomic attraction, enhancing its stability. These factors collectively explain why **7** strongly favors the formation of the rearrangement product.

**Figure 1:**
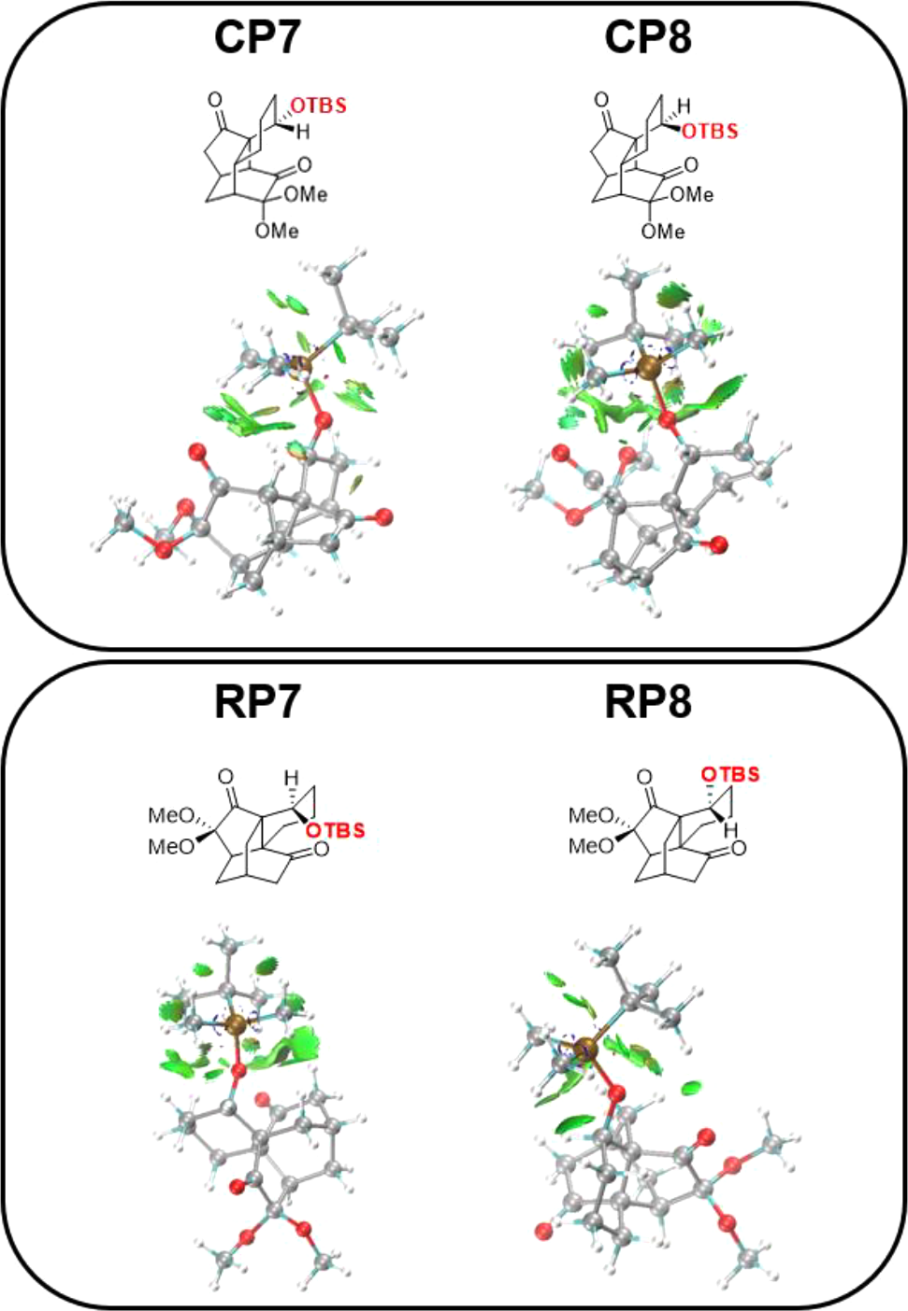
IRI analysis of rearrangement and cyclization products of **7** and **8**. The images display only the interactions surrounding OTBS.

The above results demonstrate that the orientation of allylic substitution on the fused cyclohexane ring can dictate the formation of either the rearranged product or the cyclized product. To further understand the impact of steric hindrance on this rearrangement/cyclization acyl radical reaction, a series of acyl radical precursors with mono- or disubstitutions at the alkene bridge or bridgehead, but without fused rings, was subjected to thiol-mediated acyl radical reactions (Table 1). The results of Entries 1–3 show that when R^1^ has a substitution, the reactions tend to follow the cyclization pathway, yielding cyclized products **CP10–12**. However, as the steric hindrance increased, a thiol ether side product was formed. The reaction mechanism remains unclear, but it is possible that the *tert*-butyl group shields the aldehyde group, hindering acyl radical formation. This could allow the thiol radical to add to the alkene bridge, followed by hydrogen atom abstraction. Entries 4–6 indicate that when R^2^ was substituted, the yield of cyclization products **CP13–15** decreased significantly with increasing steric hindrance. In contrast, the yield of rearrangement products **RP13–15** remained consistently around 50% regardless of the steric hindrance change. Entries 7–9 demonstrate that when a substituent was present at the bridgehead (R^3^), the yield of cyclized products **CP16–17** also decreased as steric hindrance increase, whereas the yield of rearranged products **RP16–17** increased. However, when the substitution is a *tert*-butyl group (**18**), it may hinder hydrogen atom abstraction at the adjacent carbon in rearranged product **RP18**. Consequently, the reaction underwent further decarbonylation, producing an acetal side product **22**. Entries 10–12 show the results for disubstituted precursors at alkene bridge and bridgehead. When disubstitution occurred solely at the bridge (R^1^ and R^2^, **19**), the ratio of rearranged product **RP19** to cyclized product **CP19** was 1.0:0.8. Conversely, for disubstitution involving both the bridgehead and the bridge, where one substituent is at the bridgehead (R^3^) and the other at either R^1^ (**20**) or R^2^ (**21**) on the bridge, the reaction consistently favored the formation of rearranged products **RP20** and **RP21**.

**Table 1:**
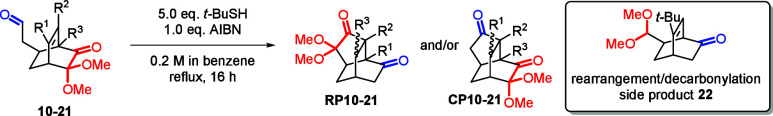
Results of the Thiol-Mediated Acyl Radical Reaction with Differently Sized Substituents at the Bridge and/or Bridgehead Positions of Bicyclo[2.2.2]­octanone

					yield^a^ (%)		
entry	aldehyde	R^1^	R^2^	R^3^	**RP**	**CP**	ratio **RP**:**CP** ^b^
1	**10**	Me	H	H	37	57	1.0:1.5
2	**11**	*i*-Pr	H	H	38^e^	58^e^	1.0:1.5
3^c^	**12**	*t*-Bu	H	H	–	25	only **CP**
4	**13**	H	Me	H	52	42	1.0:0.8
5	**14**	H	*i*-Pr	H	56	22	1.0:0.4
6^d^	**15**	H	*t*-Bu	H	45	9	1.0:0.2
7	**16**	H	H	Me	34	58	1.0:1.7
8	**17**	H	H	*i*-Pr	53^e^	20^e^	1.0:0.6
9	**18**	H	H	*t*-Bu	68% side product **22**		
10	**19**	Me	Me	H	52	40	1.0:0.8
11	**20**	Me	H	Me	62^e^	31^e^	1.0:0.5
12	**21**	H	Me	Me	57	20	1.0:0.3

a Isolated yield.

b The ratios were calculated from crude ^1^H NMR spectra.

c A thiol ether side product (51%) and starting material **12** (13%) were also isolated.

d Starting material **15** (21%) was recovered.

e An inseparable mixture of **RP** and **CP** was obtained; the individual yields were calculated based on NMR spectra.

To understand the substituent effect at the bridgehead (R^3^), we focused on two extreme cases—methyl and *tert*-butyl—to compare their effects (entries 7 and 9). DFT calculations indicate that for the methyl-substituted reactant (**16**), the reaction energies for forming the rearrangement (**RP16**) and cyclization (**CP16**) products are both approximately −23.9 kcal/mol, with the cyclization product only 0.03 kcal/mol more stable. This result aligns with experimental observations, where both products are formed but the cyclization product predominates (RP:CP = 1.0:1.7). In contrast, for the *tert*-butyl-substituted reactant (**18**), the reaction energies for forming the rearrangement (**RP18**) and cyclization (**CP18**) products are −30.3 and −23.3 kcal/mol, respectively, suggesting a strong preference for the rearrangement product, consistent with experimental data.

Since compounds **16** and **18** are not isomers, unlike **8** and **7**, their energies cannot be directly compared. Therefore, a homodesmotic reaction was employed to evaluate their relative stability.^9^ The results indicate that compound **18** is 1.7 kcal/mol higher in energy than compound **16**. Referencing to the two products of **16**, we find that replacing the methyl group with a *tert*-butyl group increases the energy of the cyclization product by 2.3 kcal/mol (**CP16** → **CP18**, Scheme 4) while it decreases the energy of the rearrangement product by −4.7 kcal/mol (**RP16** → **RP18**). Thus, this substitution disfavors cyclization while it promotes rearrangement, shifting the selectivity from a cyclization product distribution in **16** to a stronger preference for rearrangement in **18**.

**Scheme 4:**
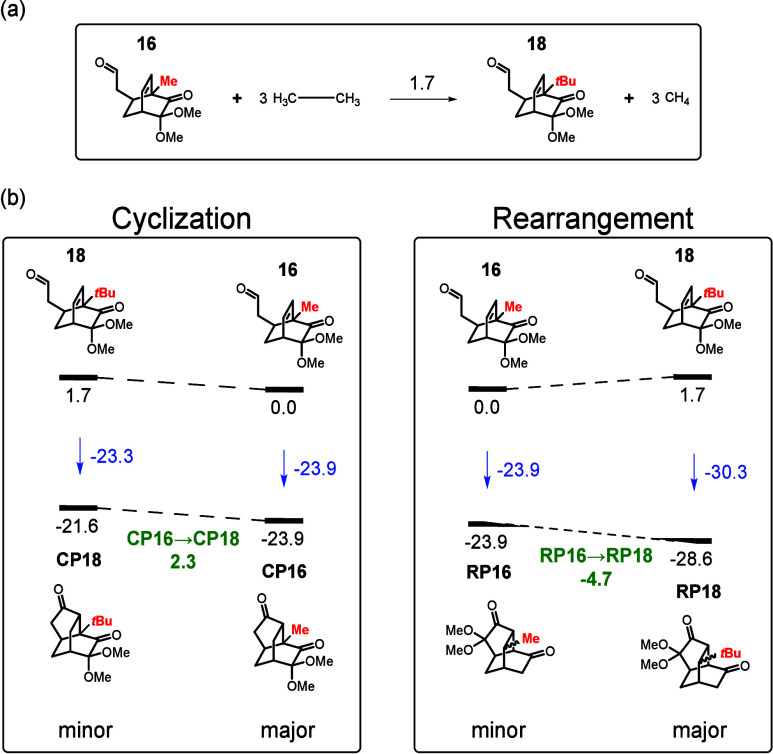
(a) Homodesmotic Reaction Used to Compare the Relative Stability of Compounds **16** and **18** and (b) Computed Cyclization and Rearrangement Energies for Compounds **16** and **18**

IRI analysis indicates that the *tert*-butyl group introduces more extensive repulsive interactions (orange and red regions) in **CP18** compared to **CP16** (Figure 2), destabilizing the cyclization product of **18**. In contrast, IRI analysis shows that, relative to **RP16**, the *tert*-butyl group in **RP18** enhances interatomic attraction, increasing its stability. These factors collectively explain why **18** exhibits a greater preference for the rearrangement product.

**Figure 2:**
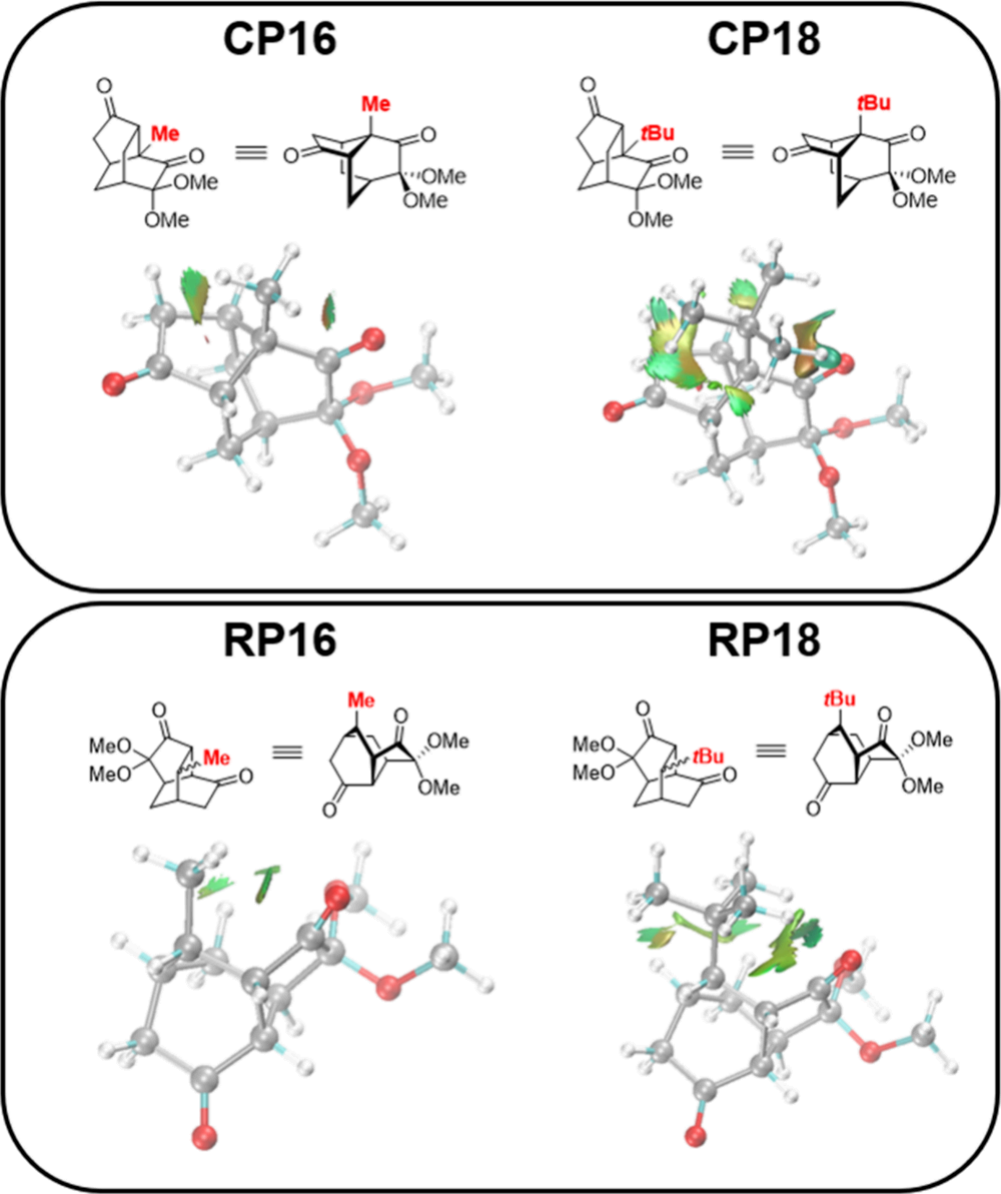
IRI analysis of substitution effect. The images show only the interactions in the vicinity of functional groups.

In summary, the orientation of the allylic substituents at the fused six-membered ring can control the reaction, leading to the formation of a rearranged product when the substituent points toward the aldehyde group or a cyclized product when it points toward the ketone group. The results with substituents of varying sizes at the alkene bridge (R^1^ and R^2^) and bridgehead (R^3^) positions show that increasing steric hindrance at R^2^ and R^3^ favors the formation of rearranged products, while the trend at the R^1^ position tends is the opposite. When a substituent is present at R^3^, adding a substituent at either R^1^ or R^2^ can drive the reaction toward a rearrangement, thereby favoring the formation of the rearranged product. DFT calculations combined with the IRI analysis indicate that interatomic interactions involving the orientation of OTBS toward the aldehyde or ketone play a key role in determining the preference for rearrangement or cyclization products. Moreover, larger substituent groups at the bridgehead position modify noncovalent interactions, introducing repulsion in the cyclization pathway and further favoring the formation of rearrangement products. Based on these rearrangement/cyclization results, we are currently developing an atom-economic synthetic strategy for isopalhinine A, which will be published in due course.

## Supplementary Material



## Data Availability

The data underlying this study are available in the published article and its Supporting Information.

## Notes

General procedures, experimental procedures, X-ray crystallographic data for **5**, characterization data for new compounds, and computational details including computational levels, energetics, and optimized structures (PDF)
