# Supplementary material for: Noncovalent Interaction Effects on the Acyl Radical Reaction of Bicyclo[2.2.2]octanone: Selective Formation of Rearrangement and Cyclization Products
Source: Org Lett. 2025 Jul 25;27(31):8394–8. doi: 10.1021/acs.orglett.5c01574 (PMC12340961; doi:10.1021/acs.orglett.5c01574)
Supplement: Supplementary file 1 [file ol5c01574_si_001.pdf]

Supporting Information

Non-Covalent Interaction Effects on the Acyl Radical Reaction of Bicyclo[2.2.2]octanone: Selective Formation of Rearrangement and Cyclization Products

Chih-Ming Chen,[a,‡] Tung-Chun Kuo,[b,‡] Julakanti Satyanarayana Reddy,[a,‡] Yi Ning Teoh,[a,c] Jyun-Sian Huang,[a,c] Sheng-Kuo Lin,[a,c] Mu-Jeng Cheng,[b,*] Hsing-Pang Hsieh[a,c,d,*]

a. Institute of Biotechnology and Pharmaceutical Research, National Health Research Institutes, Miaoli County 350, Taiwan, ROC.

b. Department of Chemistry, National Cheng Kung University, Tainan 701, Taiwan, ROC.

c. Department of Chemistry, National Tsing Hua University, Hsinchu 300, Taiwan, ROC.

d. Biomedical Translation Research Center, Academia Sinica, Taipei City 115, Taiwan, ROC.

Email: hphsieh@nhri.edu.tw

mjcheng@mail.ncku.edu.tw

Table of Contents

1. General Procedures........2
2. Experimental Procedures........3
3. Computational Details........28
4. X-Ray Crystal Data........46
5. ^{1}H- and ^{13}C-NMR Spectra........48

1. General Procedures

Unless otherwise mentioned, reagents were obtained from commercial sources and used without further purification. Reactions were heating, if need, in oil bath. All moisture- or oxygen-sensitive reactions were performed under positive pressure of anhydrous argon or nitrogen in anhydrous solvents, which were dried prior to use following standard procedures. Thin layer chromatography (TLC) was performed using Merck 5554 DC-Alufolien Kieselgel 60 F254. Flash column chromatography was performed using 230–400 mesh silica gel from Merck Art.9385 Kieselgel 60H. Except as otherwise indicated, yields were calculated after flash column chromatography. ^{1}H-NMR and ^{13}C-NMR were measured by using Varian Mercury-300 MHz, Varian Mercury-400 MHz, Bruker AVANCE NEO-400 MHz and Bruker AVANCE NEO-600 MHz spectrometers. Chemical shifts are reported as δ values in ppm, and calibrated using residual undeuterated solvent ($CDCl_3$ (7.27 ppm) or CD_3OD (3.31 ppm)) as internal reference for ^{1}H NMR and the deuterated solvent ($CDCl_3$ (77.00 ppm) or CD_3OD (49.00 ppm)) as internal standard for ^{13}C NMR. Coupling constants are reported in Hz; multiplicities are indicated as follows: s (singlet); d (doublet); t (triplet); m (multiplet). Infrared (IR) spectra were recorded on a Perkin Elmer FT-IR Spectrometer Spectrum RXI and are reported in wavenumbers (cm^{-1}). High resolution mass spectra (HRMS) were recorded using a VARIAN 901-MS (TOF). Melting points were determined with a Krüss Optronic KSP1N melting point meter. X-ray diffraction analysis was measured on a Bruker D8 Dual Single Crystal X-ray Diffractometer (D8 Venture *IuS* 3.0 Dual source) with monochromatic MoKα radiation.

2. Experimental Procedures

Compound S2:

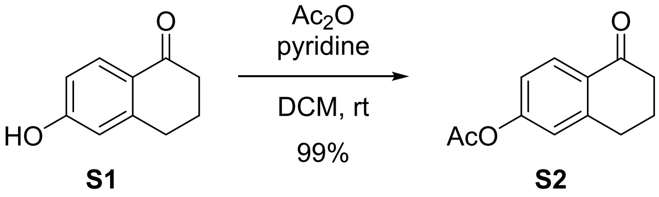

A solution of the phenol **S1** (60.00 g, 369.9 mmol) and pyridine (89.6 mL, 1110 mmol) in DCM (1.23 L) was stirred for 15 min at room temperature, followed by addition of Ac_2O (69.9 mL, 740 mmol) at the same temperature. The resulting mixture was stirred at room temperature for 24 h. The reaction mixture was concentrated under reduced pressure, and the residue was directly purified by silica gel column chromatography using ethyl acetate/hexane (3:7) as eluent, affording acetylation product **S2** (74.92 g, 366.9 mmol, 99%) as a yellow liquid.

$\mathbf{R_f}$ = 0.50 (EA/Hex = 3:7).

^{1}H NMR (600 MHz, $CDCl_3$): δ 8.06 (d, J = 8.4 Hz, 1H), 7.03-7.00 (m, 2H), 2.96 (t, J = 6.0 Hz, 2H), 2.64 (t, J = 6.6 Hz, 2H), 2.31 (s, 3H), 2.16-2.12 (m, 2H).

^{13}C NMR (100 MHz, $CDCl_3$): δ 197.0, 168.8, 154.2, 146.2, 130.3, 129.0, 121.3, 120.0, 38.8, 29.6, 23.0, 21.0.

IR (neat): 3520, 2947, 2871, 1766, 1681, 1370, 1186, 910, 812 cm^{-1}.

HRMS (ESI): *m/z* calcd for $C_{12}H_{12}NaO_3$ $[M+Na]^+$: 227.0684; found: 227.0683.

Compound S3

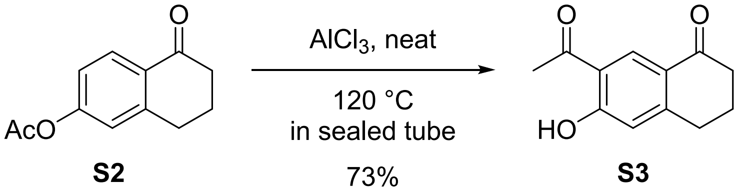

The liquid ketone **S2** (20.00 g, 97.93 mmol) was placed in a sealed tube, and $AlCl_3$ (40.48 g, 303.6 mmol) was carefully added in three portions at 0 ℃. The resulting mixture was stirred at room temperature for 10 min, then slowly heated to 120 ℃, and stirred for 6 h at the same temperature. The reaction was then quenched with 2 N HCl under an ice bath. After stirring for 30 min at room temperature, the mixture was extracted with ethyl acetate (3 × 200 mL), and the combined organic layers were washed with saturated $NaHCO_3$ aqueous solution (50 mL) and brine (50 mL), then dried over Na_2SO_4. After concentration under reduced pressure, the residue was purified by silica gel column chromatography using ethyl acetate/hexane (1:4) as eluent, affording

acetophenone **S3** (14.50 g, 71.00 mmol, 73%) as a light-yellow solid.

R_f = 0.50 (EA/Hex = 3:7).
Mp: 114.5 – 115.6 °C.
^{1}H NMR (600 MHz, $CDCl_3$): δ 12.62 (s, 1H), 8.50 (s, 1H), 6.80 (s, 1H), 2.94 (t, J = 6.0 Hz, 2H), 2.68 (s, 3H), 2.64 (t, J = 6.0 Hz, 2H), 2.14-210 (m, 2H).
^{13}C NMR (100 MHz, $CDCl_3$): δ 204.8, 196.4, 165.6, 153.1, 131.6, 124.8, 118.6, 117.1, 38.6, 30.1, 26.7, 22.6.
IR (neat): 3850, 2947, 2885, 1677, 1634, 1326, 1057, 829 cm^{-1}.
HRMS (ESI): *m/z* calcd for $C_{12}H_{11}O_3$ $[M-H]^-$: 203.0708; found: 203.0708.

Compound S4

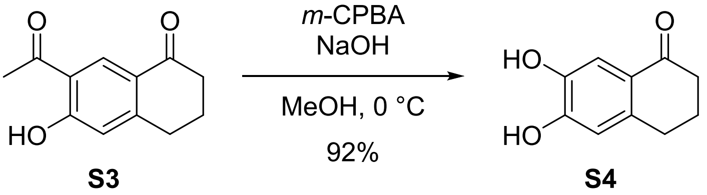

A solution of acetophenone **S3** (22.30 g, 109.2 mmol) in MeOH (728 mL) was stirred at 0 °C for 10 min, followed by portion-wise addition of *m*-CPBA (75 wt%, 37.68 g, 163.8 mmol) at the same temperature. After 10 min of stirring, 5N NaOH (65.51mL, 327.6 mmol) was added dropwise over 30 min at 0 °C. The resultant mixture was stirred at 0 °C for an additional 30 min. The reaction was then quenched with 2N HCl until pH reached 2-3 at 0 °C. The solvent was removed under reduced pressure, and water (100 mL) was added to the reaction mixture. The solution was extracted with DCM (2 × 450 mL), and the combined organic layers were washed with saturated $NaHCO_3$ aqueous solution (100 mL) and brine (100 mL), then dried over $NaSO_4$. The organic layer was concentrated under reduced pressure, and the residue was dissolved in ether (100 mL), stirred for 30 min at room temperature, and filtered to afford catechol **S4** (17.97 g, 100.9 mmol, 92%) as a pale-yellow solid.

R_f = 0.30 (EA/Hex = 1:1).
Mp: 189.0 – 191.0 °C.
^{1}H NMR (400 MHz, *d4*-MeOD): δ 7.35 (s, 1H), 6.64 (s, 1H), 2.81 (t, J = 6.0 Hz, 2H), 2.52 (t, J = 6.0 Hz, 2H), 2.08-2.01 (m, 2H).
^{13}C NMR (150 MHz, *d4*-MeOD): δ 200.1, 153.0, 145.4, 140.6, 125.9, 115.3, 113.7, 39.5, 30.0, 24.8.
IR (neat): 3306, 2943, 1651, 1586, 1461, 1286, 1181, 843, 804 cm^{-1}.
HRMS (ESI): *m/z* calcd for $C_{10}H_9O_3$ $[M-H]^-$: 177.0551; found: 177.0549.

Compound S5

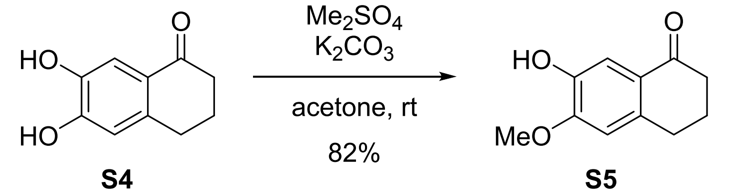

A solution of catechol **S4** (9.76 g, 54.8 mmol) and K_2CO_3 (8.32 g, 60.3 mmol) in acetone (137.0 mL) was stirred at 0 ℃ for 10 min. Me_2SO_4 (5.71 mL, 60.3 mmol) was then added dropwise at the same temperature. The reaction mixture was gradually warmed to room temperature and stirred for 8 h. The reaction mixture was filtered through a Celite pad and washed with acetone (100 mL). The filtrate was concentrated under reduced pressure, and the crude product was purified by silica gel column chromatography using ethyl acetate/hexane (1:4) as eluent, affording 2-methoxyphenol **S5** (8.59 g, 44.7 mmol, 82%) as a white solid.

R_f = 0.30 (EA/Hex = 3:7).
Mp: 152.0 – 153.5 ℃.
^{1}H NMR (600 MHz, $CDCl_3$): δ 7.56 (s, 1H), 6.66 (s, 1H), 5.53 (m, 1H), 3.94 (s, 3H), 2.87 (t, J = 6.0 Hz, 2H), 2.58 (t, J = 6.6 Hz, 2H), 2.12-2.08 (m, 2H).
^{13}C NMR (150 MHz, $CDCl_3$): δ 197.3, 151.1, 144.3, 138.4, 126.4, 112.2, 109.6, 55.9, 38.6, 29.5, 23.6.
IR (neat): 3400, 2942, 2862, 1656, 1608, 1508, 1323, 1274, 824 cm^{-1}.
HRMS (ESI): m/z calcd for $C_{11}H_{11}O_3$ $[M-H]^+$: 191.0708; found: 191.0708.

Compound S6

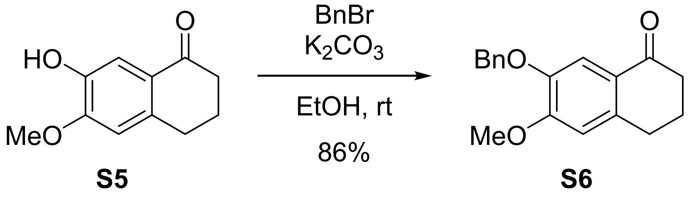

A solution of phenol **S5** (22.87 g, 119.0 mmol) and K_2CO_3 (82.22 g, 594.9 mmol) in ethanol (595 mL) was stirred at 0 ℃ for 5 min. Benzyl bromide (28.26 mL, 238.0 mmol) was then added dropwise at the same temperature. The resulting solution was gradually warmed to room temperature and stirred for 7 h. The reaction mixture was filtered through a Celite pad and washed with ethyl acetate (100 mL). The filtrate was concentrated under reduced pressure, and the crude product was purified by silica gel column chromatography using ethyl acetate/hexane (3:7) as eluent, affording ketone **S6** (29.00 g, 102.7 mmol, 86%) as a white solid.

R_f = 0.40 (EA/Hex = 3:7).

Mp: 115.0 – 116.0 ℃.

^{1}H NMR (600 MHz, $CDCl_3$): δ 7.59 (s, 1H), 7.45 (d, *J* = 7.5 Hz, 2H) 7.36 (t, *J* = 7.5 Hz, 2H), 7.31-7.28 (m, 1H), 6.68 (s, 1H), 5.15 (s, 2H), 3.92 (s, 3H), 2.88 (t, *J* = 6.0 Hz, 2H), 2.58 (t, *J* = 6.0 Hz, 2H), 2.13-2.09 (m, 2H).

^{13}C NMR (150 MHz, $CDCl_3$): δ 197.1, 153.9, 146.9, 139.5, 136.5, 128.4, 127.9, 127.4, 125.6, 110.4, 110.4, 70.6, 56.0, 38.4, 29.4, 23.5.

IR (neat): 3437, 2935, 2914, 1667, 1595, 1508, 1267, 1151, 1028, 825 cm^{-1}.

HRMS (ESI): *m/z* calcd for $C_{18}H_{18}NaO_3$ $[M+Na]^+$: 305.1153; found: 305.1156.

Compound S7

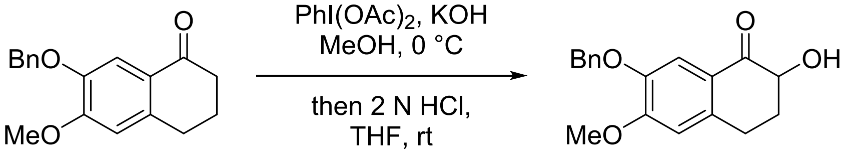

S6 **S7**

A solution of ketone **S6** (25.00 g, 88.55 mmol) in MeOH (500 mL) was stirred at 0 ℃ for 5 min. A solution of KOH (49.68 g, 885.5 mmol) in MeOH (208 mL) was then slowly added at the same temperature, and the mixture was stirred for an additional 10 min. $PhI(OAc)_2$ (34.22 g, 106.3 mmol) was added portion-wise at 0 ℃, and the reaction was stirred for 2 h at the same temperature, followed by 30 min at room temperature. The solvent was then removed under reduced pressure, and the residue was dissolved in ethyl acetate (300 mL) and 3% $NaHCO_3$ aqueous solution (300 mL). The aqueous layer was separated and further extracted with ethyl acetate (2 × 300 mL). The combined organic layers were dried over Na_2SO_4 and concentrated under reduced pressure. The resulting residue was dissolved in THF (90 mL) and 2 N HCl aqueous solution (30 mL) and stirred at room temperature for 2 h. The resulting mixture was then extracted with ethyl acetate (2 × 300 mL), dried over Na_2SO_4, and concentrated under reduced pressure. The crude product was recrystallized from ether/hexane (2:1), affording α-hydroxy ketone **S7** (24.28 g, 81.38 mmol, 92%) as a light-pink solid.

R_f = 0.50 (EA/Hex = 1:1).

Mp: 169.5 – 171.5 ℃.

^{1}H NMR (400 MHz, $CDCl_3$): δ 7.54 (s, 1H), 7.47-7.44 (m, 2H) 7.39-7.35 (m, 2H), 7.33-7.29 (m, 1H), 6.68 (s, 1H), 5.16 (m, 2H), 4.29 (dd, *J* = 13.2, 5.2 Hz, 1H), 3.93 (s, 3H), 3.12-3.03 (m, 1H), 2.96-2.90 (m, 1H), 2.53-2.46 (m, 1H), 2.06-1.95 (m, 1H).

^{13}C NMR (100 MHz, $CDCl_3$): δ 198.1, 154.6, 147.2, 139.6, 136.3, 128.5, 128.0, 127.4, 123.1, 110.4, 110.4, 73.2, 70.7, 56.0, 32.0, 27.5.

IR (neat): 3487, 2932, 2838, 1663, 1596, 1512, 1267, 1094, 1010, 821 cm^{-1}.
HRMS (ESI): *m/z* calcd for $C_{18}H_{18}NaO_4$ $[M+Na]^+$: 321.1102; found: 321.1102.

Compound S8

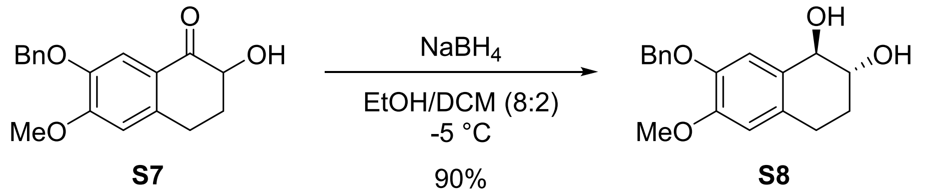

A solution of ketone **S7** (15.00 g, 50.28 mmol) in EtOH/DCM (8:2, 400 mL) was stirred at -10 ℃ for 5 min. $NaBH_4$ (2.853 g, 75.42 mmol) was the added portion-wise at the same temperature. The resulting solution was stirred at -5 ℃ for 24 h. The reaction was then quenched with water (50 mL) and saturated NH_4Cl aqueous solution (50 mL) at 0 ℃, then concentrated under reduced pressure. The aqueous layer was extracted with ethyl acetate (3 × 200 mL), and the combined organic layers were dried over Na_2SO_4, filtered, and concentrated under reduced pressure. The residue was purified by silica gel column chromatography using ethyl acetate/hexane (1:1) as eluent, affording diol **S8** (13.60 g, 45.28 mmol, 90%) as a white solid.

$\mathbf{R_f}$ = 0.25 (EA/Hex = 1:1).
Mp: 119.0 – 121.0 ℃.
^{1}**H NMR** (600 MHz, $CDCl_3$): δ 7.45-744 (m, 2H), 7.37-7.35 (m, 2H) 7.31-7.28 (m, 1H), 7.08 (s, 1H), 6.59 (s, 1H), 5.13 (m, 2H), 4.46 (d, J = 7.8 Hz, 1H), 3.85 (s, 3H), 3.79-3.75 (m, 1H), 2.89-2.79 (m, 2H), 2.25 (bs, 1H), 2.14-2.10 (m, 1H), 2.04 (bs, 1H), 1.87-1.80 (m, 1H).
^{13}**C NMR** (150 MHz, $CDCl_3$): δ 149.0, 146.9, 137.0, 128.8, 128.4, 127.7, 127.4, 112.2, 111.2, 74.8, 73.6, 71.0, 55.9, 28.7, 27.3.
IR (neat): 3384, 2914, 2849, 1609, 1513, 1454, 1263, 1116, 969 cm^{-1}.
HRMS (ESI): *m/z* calcd for $C_{18}H_{19}O_4$ $[M-H]^-$: 299.1283; found: 299.1283.

Compound S9

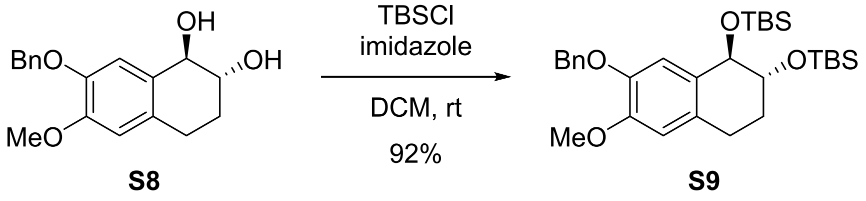

A solution of diol **S8** (12.00 g, 39.95 mmol) in DCM (500 mL) was stirred at room temperature for 5 min. Imidazole (16.31 g, 239.7 mmol) and TBSCl (18.06 g, 119.9

mmol) were then sequentially added at the same temperature. The resulting mixture was stirred at room temperature for 12 h. The reaction was quenched with water (100 mL) and extracted with DCM (2 × 200 mL). The combined organic layers were dried over Na_2SO_4, filtered, and concentrated under reduced pressure. The residue was purified by silica gel column chromatography using ethyl acetate/hexane (1:24) as eluent, affording **S9** (19.44 g, 36.76 mmol, 92%) as a colorless oil.

R_f = 0.50 (EA/Hex = 1:9).
^{1}H NMR (400 MHz, $CDCl_3$): *δ* 7.44-7.41 (m, 2H), 7.36-7.32 (m, 2H) 7.30-7.27 (m, 1H), 6.69 (s, 1H), 6.58 (s, 1H), 5.11 (m, 2H), 4.26 (d, *J* = 3.6 Hz, 1H), 3.96-3.93 (m, 1H), 3.86 (s, 3H), 2.92-2.84 (m, 1H), 2.57-2.51 (m, 1H), 2.11-2.03 (m, 1H), 1.76-1.70 (m, 1H), 0.84 (s, 9H), 0.81 (s, 9H), 0.10 (s, 3H), 0.06 (s, 3H), 0.03 (s, 3H), -0.00 (s, 3H).
^{13}C NMR (100 MHz, $CDCl_3$): *δ* 148.6, 146.0, 137.4, 129.8, 128.8, 128.4, 127.6, 127.0, 115.9, 111.3, 72.0, 71.1, 71.0, 55.7, 25.8, 25.7, 25.4, 23.8, 18.0, 17.9, -4.1, -4.5, -4.6.
IR (neat): 2952, 2856, 1610, 1516, 1360, 1256, 1126, 1005, 853 cm^{-1}.
HRMS (ESI): *m/z* calcd for $C_{30}H_{48}NaO_4Si_2$ $[M+Na]^+$: 551.2988; found: 551.3000.

Compound S10

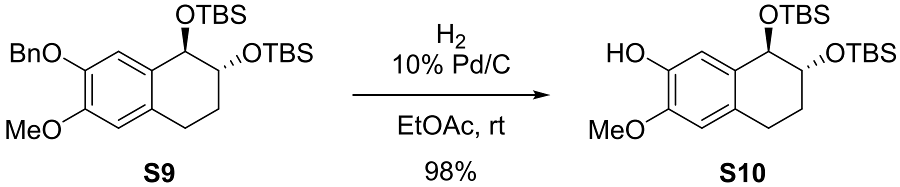

S9 S10

A solution of benzyl ether **S9** (23.91 g, 45.21 mmol) in ethyl acetate (300 mL) was stirred at room temperature, followed by the addition of Pd/C (4810. mg, 4.52 mmol) at the same temperature. The resulting mixture was stirred under a H_2 balloon room temperature for 5 h. The reaction mixture was then filtered through a Celite pad and washed with ethyl acetate (100 mL). The filtrate was concentrated under reduced pressure, affording 2-methoxyphenol **S10** (19.51 g, 44.47 mmol, 98%) as a white solid.

R_f = 0.40 (EA/Hex = 1:9).
Mp: 129.0 – 131.0 ℃.
^{1}H NMR (400 MHz, $CDCl_3$): *δ* 6.73 (s, 1H), 6.54 (s, 1H), 5.38 (s, 1H), 4.30 (d, *J* = 3.2 Hz, 1H), 3.97-3.95 (m, 1H), 3.85 (s, 3H), 2.92-2.84 (m, 1H), 2.55-2.49 (m, 1H), 2.13-2.05 (m, 1H), 1.76-1.69 (m, 1H), 0.86 (s, 9H), 0.81 (s, 9H), 0.15 (s, 3H), 0.11 (s, 3H), 0.06 (s, 3H), 0.04 (s, 3H).
^{13}C NMR (100 MHz, $CDCl_3$): *δ* 145.6, 143.3, 129.5, 128.5, 115.9, 110.1, 71.8, 70.9,

55.7, 25.8, 25.7, 25.1, 23.6, 18.0, 18.0, -4.0, -4.2, -4.5, -4.6.
IR (neat): 3520, 2951, 2856, 1515, 1360, 1255, 1080, 822 cm^{-1}.
HRMS (ESI): *m/z* calcd for $C_{23}H_{41}O_4Si_2$ [M-H]$^-$: 437.2543; found: 437.2542.

Compound S11 and S12

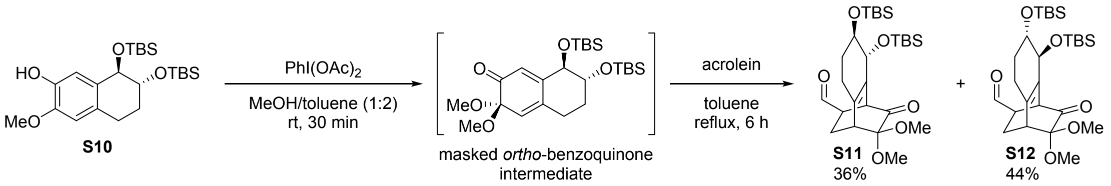

A solution of 2-methoxyphenol **S10** (8.00 g, 18.2 mmol) in MeOH (40 mL) and toluene (80 mL) was stirred at 0 °C for 10 min. PhI(OAc)$_2$ (7.04 g, 21.9 mmol) was then added portion-wise at the same temperature, and the mixture was stirred at room temperature for 30 min. Subsequently, toluene (650 mL) and acrolein (4.92 mL, 72.9 mmol) were added sequentially at room temperature. The reaction mixture was then heated to 100 °C and stirred for 6 h. The reaction mixture was concentrated under reduced pressure and purified by silica gel column chromatography using ethyl acetate/hexane (1:19) as eluent, affording aldehyde isomer **S11** (3.43 g, 6.54 mmol, 36%) as a colorless solid and aldehyde isomer **S12** (4.20 g, 8.00 mmol, 44%) as a colorless solid.

For isomer S11:
R$_f$ = 0.55 (EA/Hex = 1:4).
Mp: 96.2 – 97.5 °C.
^{1}H NMR (600 MHz, CDCl$_3$): δ 9.42 (s, 1H), 3.76 (t, J = 2.4 Hz, 1H), 3.63 (s, 1H), 3.30 (s, 6H), 3.24 (d, J = 2.4 Hz, 1H), 2.87 (t, J = 2.4 Hz, 1H), 2.84-2.82 (m, 1H), 2.41-2.35 (m, 1H), 2.04-1.94 (m, 2H), 1.83-1.73 (m, 2H), 1.64-1.61 (m, 1H), 0.85 (s, 9H), 0.2 (s, 9H), 0.08 (s, 3H), 0.07 (s, 3H), 0.02 (s, 3H), 0.00 (s, 3H).
^{13}C NMR (150 MHz, CDCl$_3$): δ 200.8, 200.5, 143.8, 125.1, 94.9, 70.3, 70.0, 51.2, 50.9, 49.5, 46.9, 42.8, 25.7, 25.5, 24.7, 23.7, 21.1, 17.8, 17.7, -4.0, -4.6, -4.7.
IR (neat): 2952, 2894, 1737, 1439, 1254, 1061, 828 cm^{-1}.
HRMS (ESI): *m/z* calcd for $C_{27}H_{47}O_6Si_2$ [M-H]$^-$: 523.2917; found: 523.2911.

For isomer S12:
R$_f$ = 0.50 (EA/Hex = 1:4).
Mp: 93.0 – 94.0 °C.
^{1}H NMR (400 MHz, CDCl$_3$): δ 9.44 (d, J = 1.6 Hz, 1H), 3.77-3.75 (m, 1H), 3.62 (dd, J = 4.0, 1.2 Hz, 1H), 3.34 (d, J = 2.4 Hz, 1H), 3.31 (s, 3H), 3.30 (s, 3H), 2.92-2.87 (m, 2H), 2.38.2.29 (m, 1H), 2.20-2.13 (m, 1H), 2.00-1.94 (m, 1H), 1.91-1.84 (m, 1H), 1.68-

1.64 (m, 1H), 1.63-1.56 (m, 1H), 0.85 (s, 9H), 0.83 (s, 9H), 0.14 (s, 3H), 0.08 (s, 3H), 0.03 (s, 3H), 0.03 (s, 3H).

^{13}C NMR (100 MHz, $CDCl_3$): δ 200.1, 199.8, 142.2, 126.7, 95.2, 70.5, 70.4, 51.0, 50.2, 49.5, 47.7, 42.8, 25.6, 25.5, 24.3, 23.3, 21.8, 17.9, 17.8, -4.3, -4.4, -4.7, -4.9.

IR (neat): 2952, 2929, 2891, 2857, 1738, 1730, 1472, 1463, 1439, 1390, 1361, 1255, 1205, 1170, 1131, 1092, 1065, 1012, 978, 940, 886, 865 cm^{-1}.

Acyl Radical Precursor 3

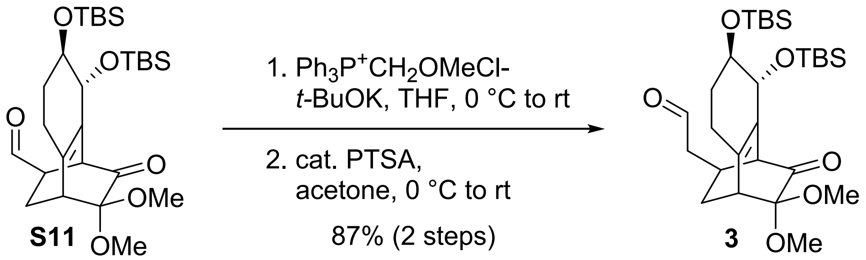

A solution of $PPh_3CH_2OCH_3Cl$ (261 mg, 0.76 mmol) in THF (2.00 mL) was stirred at 0 °C for 10 min. A 1.0 M solution of *t*-BuOK in THF (0.76 mL, 0.76 mmol) was then added dropwise at the same temperature, and the mixture was stirred for an additional 30 min. Subsequently, a solution of aldehyde **S11** (100 mg, 0.191 mmol) in THF (1.80 mL) was added at 0 °C, and the reaction mixture was stirred at the same temperature for 1.5 h. The reaction was quenched by water (10 mL), followed by extraction with ethyl acetate (2 × 25 mL). The combined organic layers were washed with brine (10 mL), dried over Na_2SO_4, filtered, and concentrated under reduced pressure. The residue was purified by silica gel column chromatography using ethyl acetate/hexane (1:19) as eluent, affording a 1:1 mixture of *E/Z* isomers of methyl enol ether intermediate (100 mg, 0.181 mmol, 95%) as a white solid.

A solution of methyl enol ether intermediate (100 mg, 0.181 mmol) and PTSA•H_2O (3 mg, 0.018 mmol) in acetone (6.00 mL) was stirred at room temperature for 5 h. The reaction was then quenched by adding saturated $NaHCO_3$ aqueous solution (1.00 mL), and the solvent was removed under reduced pressure. The residue was treated with water (10 mL) and extracted with ethyl acetate (2 × 50 mL). The combined organic layers were washed with brine (10 mL) and dried over Na_2SO_4, filtered, and concentrated under reduced pressure. The crude product was purified by silica gel column chromatography using ethyl acetate/hexane (1:19) as eluent, affording aldehyde **3** (90 mg, 0.17 mmol, 92%) as a white solid.

R_f = 0.40 (EA/Hex = 1:4).

^{1}H NMR (400 MHz, $CDCl_3$): δ 9.70 (t, *J* = 1.2 Hz, 1H), 3.80-3.78 (m, 1H), 3.59 (t, *J* = 1.2 Hz, 1H), 3.30 (s, 3H), 3.29 (s, 3H), 2.73 (dd, *J* = 5.6, 2.0 Hz, 2H), 2.66-2.60 (m,

1H), 2.49-2.47 (m, 2H), 2.43-2.34 (m, 1H), 2.31-2.24 (m, 2H), 1.97-1.91 (m, 1H), 1.86-1.78 (m, 1H), 1.68-1.62 (m, 1H), 0.89 (s, 9H), 0.82 (s, 9H), 0.10 (s, 3H), 0.07 (s, 3H), 0.03 (s, 3H), 0.00 (s, 3H).

^{13}C NMR (100 MHz, $CDCl_3$): δ 202.2, 201.1, 142.8, 125.3, 94.1, 70.3, 70.0, 56.0, 50.8, 50.7, 49.5, 42.9, 28.4, 27.9, 25.8, 25.5, 24.7, 23.7, 17.8, 17.8, -3.8, -4.6, -4.7.

IR (neat): 2926, 1735, 1654, 1459, 1254, 1047, 887, 822 cm^{-1}.

HRMS (ESI): *m/z* calcd for $C_{28}H_{49}O_6Si_2$ $[M-H]^-$: 537.3067; found: 537.3060.

Acyl Radical Precursor 4

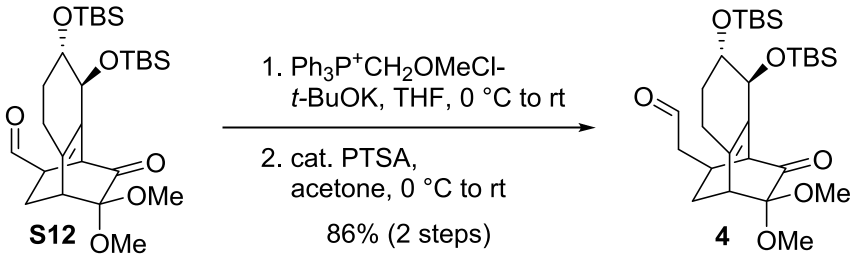

S12 **4**

Following the procedure for aldehyde **3**, aldehyde **S12** (750 mg, 1.43 mmol), $PPh_3CH_2OCH_3Cl$ (1.85 g, 5.71 mmol), and a solution of 1.0 M *t*-BuOK in THF (5.71 mL, 5.71 mmol) in THF (8.6 mL) were used to prepare methyl enol ether intermediate (725 mg, 1.31 mmol, 92%) as a viscous oil.

Subsequently, methyl enol ether intermediate (530 mg, 0.958 mmol) and PTSA•H_2O (9 mg, 0.05 mmol) in acetone (30.0 mL) were employed to afford aldehyde **4** (480 mg, 0.891 mmol, 93%) as a white solid.

^{1}H NMR (600 MHz, $CDCl_3$): δ 9.67 (t, J = 1.2 Hz, 1H), 3.82-3.81 (m, 1H), 3.57 (d, J = 2.4 Hz, 1H), 3.29 (s, 3H), 3.28 (s, 3H), 2.79 (d, J = 1.8 Hz, 1H), 2.75 (t, J = 3.0 Hz, 1H), 2.69-2.64 (m, 1H), 2.39 (dd, J = 7.2, 1.2 Hz, 2H), 2.36-2.30 (m, 1H), 2.27 (ddd, J = 12.6, 9.0, 2.4 Hz, 1H), 2.00 (dd, J = 6.0, 1.8 Hz, 1H), 1.96 (t, J = 6.0 Hz, 1H), 1.94-1.90 (m, 1H), 1.65-1.61 (m, 1H), 0.86 (s, 9H), 0.84 (s, 9H), 0.09 (s, 3H), 0.07 (s, 3H), 0.06 (s, 6H).

^{13}C NMR (150 MHz, $CDCl_3$): δ 201.5, 200.6, 141.2, 126.4, 94.7, 71.0, 70.9, 54.4, 50.9, 49.4, 49.4, 42.9, 30.1, 27.6, 25.8, 25.7, 24.1, 23.2, 18.0, 17.8, -4.4, -4.5, -4.7, -4.9.

Standard Procedure for the Thiol-Mediated Acyl Radical Reaction

A solution of acyl radical precursors **3**, **4**, **7**, **8**, and **10-21** (1.0 eq.), *t*-BuSH (4.0-5.0 eq.), and azobisisobutyronitrile (AIBN, 1.0 eq.) in benzene (0.1-0.2 M) was heated to reflux and stirred under an argon atmosphere for 16 h. After cooling to room temperature, the solvent was removed under reduced pressure. The crude residue was purified by silica gel column chromatography using ethyl acetate/hexane (1:19 to 1:3)

as eluent to afford the corresponding cyclized products **CP** and/or rearranged products **RP**.

*Acyl radical precursors **7**, **8**, and **10-21** were synthesized from the corresponding 2-methoxyphenol compounds, following the known procedure published in *Org. Lett.* **2023**, *25*, 7757-7762, as well as the procedure described above.

Thiol-Mediated Acyl Radical Reaction on Precursor 3 and Further Desilylation

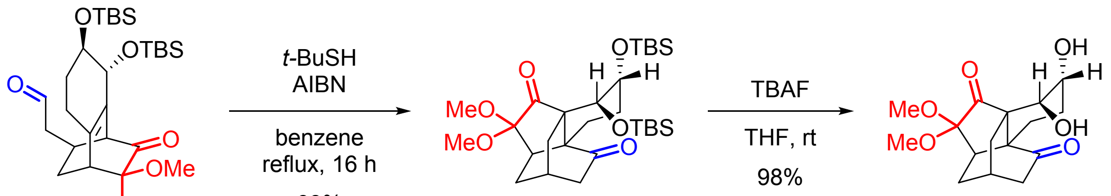

Following the standard procedure, aldehyde **3** (80 mg, 0.15 mmol), *t*-BuSH (74 μL, 0.59 mmol), and AIBN (24 mg, 0.15 mmol) in benzene (1.5 mL) were used to afford rearranged product **RP3** (54 mg, 0.10 mmol, 68%) as a white solid.

A solution of silyl ether **RP3** (1.10 g, 2.04 mmol) in THF (10 mL) was stirred at 0 °C for 10 min. A 1.0 M solution of TBAF in THF (8.16 mL, 8.16 mmol) was then added dropwise at the same temperature, and the resulting mixture was stirred at room temperature for 12 h. Subsequently, an additional portion of 1.0 M TBAF in THF (8.16 mL, 8.16 mmol) was added dropwise at 0 °C, and the reaction was further stirred at room temperature for another 12 h. The reaction was quenched with water (30 mL) and extracted with ethyl acetate (2 × 100 mL). The combined organic layers were washed with brine (30 mL), dried over Na_2SO_4, filtered, and concentrated under reduced pressure. The crude product was purified by silica gel column chromatography using ethyl acetate/hexane (1:1) as eluent, affording diol **5** (618 mg, 1.99 mmol, 98%) as a white solid.

For rearranged product **RP3**:
R_f = 0.45 (EA/Hex = 1:4).
Mp: 92.0 – 93.0 °C.
^{1}H NMR (600 MHz, $CDCl_3$): δ 3.88 (d, J = 3.6 Hz, 1H), 3.74 (q, J = 6.0, 3.0 Hz, 1H), 3.40 (s, 3H), 3.26 (s, 3H), 2.30-2.18 (m, 4H), 2.17-2.07 (m, 3H), 1.91-1.88 (m, 1H), 1.83-1.80 (m, 1H), 1.62-1.57 (m, 2H), 1.36-1.32 (m, 1H), 0.87 (s, 9H), 0.84 (s, 9H), 0.08 (s, 6H), 0.05 (s, 3H), 0.04 (s, 3H).
^{13}C NMR (150 MHz, $CDCl_3$): δ 212.8 (C), 208.5 (C), 102.8 (C), 71.5 (CH), 70.6 (CH), 55.7 (C), 51.1 (CH_3), 49.8 (CH_3), 48.7 (C), 45.6 (CH_2), 45.0 (CH), 35.7 (CH_2), 27.7 (CH_2), 26.2 (CH), 25.9 (CH_3), 25.8 (CH_3), 24.7 (CH_2), 18.5 (C), 18.4 (CH_2), 18.0 (C).

-3.9 (CH_3), -4.5 (CH_3), -4.8 (CH_3), -4.9 (CH_3).

IR (neat): 2928, 2855, 1756, 1719, 1460, 1254, 1078, 910, 812 cm^{-1}.

HRMS (ESI): *m/z* calcd for $C_{28}H_{49}O_6Si_2$ $[M-H]^-$: 537.3067; found: 537.3057.

For diol **5**:

$\mathbf{R_f}$ = 0.4 (EA/Hex = 1:1).

Mp: 200.0 – 201.5 ℃.

^{1}**H NMR** (400 MHz, $CDCl_3$): δ 5.16 (d, J = 4.4 Hz, 1H), 4.37 (d, J = 2.0 Hz, 1H), 3.65 (t, J = 4.0 Hz, 1H), 3.59-3.56 (m, 1H), 3.26 (s, 3H), 3.12 (s, 3H), 2.37-2.32 (m, 1H), 2.22-2.05 (m, 5H), 1.89-1.84 (m, 1H), 1.67-1.55 (m, 3H), 1.36-1.31 (m, 1H), 1.20.-1.15 (m, 1H).

^{13}**C NMR** (100 MHz, $CDCl_3$): δ 212.3, 208.5, 102.6, 68.3, 67.9, 54.6, 50.4, 49.0, 48.0, 45.1, 44.4, 35.3, 27.1, 25.5, 23.5, 18.1.

IR (neat): 3417, 2947, 2910, 2837, 1747, 1714, 1455, 1234, 1198, 1152, 1095, 1056, 1027, 976, 945 cm^{-1}.

HRMS (ESI): *m/z* calcd for $C_{16}H_{21}O_6$ $[M-H]^-$: 309.1338; found: 309.1341.

Thiol-Mediated Acyl Radical Reaction on Precursor 4

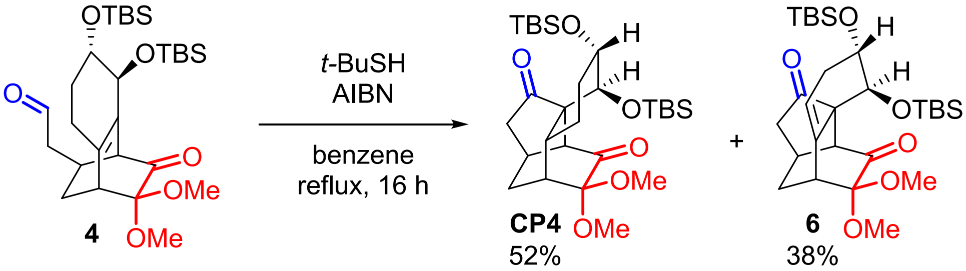

Following the standard procedure, aldehyde **4** (250 mg, 0.464 mmol), *t*-BuSH (200 µL, 1.86 mmol), and AIBN (76 mg, 0.46 mmol) in benzene (5.7 mL) were used to afford cyclized product **CP4** (130 mg, 0.241 mmol, 52%) as an oil, and olefin side product **6** (95 mg, 0.18 mmol, 38%) as a solid.

For cyclized product **CP4**:

$\mathbf{R_f}$ = 0.30 (EA/Hex = 1:9).

^{1}**H NMR** (600 MHz, $CDCl_3$): δ 3.98 (d, J = 7.8 Hz, 1H), 3.43 (dt, J = 7.8, 3.6 Hz, 1H), 3.30 (s, 3H), 3.24 (s, 3H), 3.14 (d, J = 3.6 Hz, 1H), 2.78-2.72 (m, 1H), 2.52-2.45 (m, 1H), 2.29 (d, J = 16.8 Hz, 1H), 2.25-2.18 (m, 2H), 2.05-1.95 (m, 2H), 1.84-1.77 (m, 1H), 1.73-1.65 (m, 1H), 1.32-1.25 (m, 1H), 0.99 (dt, J = 7.8, 3.0 Hz, 1H), 0.88 (s, 9H), 0.86 (s, 9H), 0.06 (s, 3H), 0.05 (s, 6H), -0.10 (s, 3H).

^{13}**C NMR** (150 MHz, $CDCl_3$): δ 219.1 (C), 205.8 (C), 96.1 (C), 76.5 (CH), 73.4 (CH), 62.3 (C), 50.8 (CH), 49.4 (CH_3), 49.0 (CH_3), 47.6 (CH_2), 41.5 (CH), 38.2 (CH), 31.5

(CH_2), 30.9 (CH_2), 30.7 (CH), 26.4 (CH_3), 26.3 (CH_3), 24.3 (CH_2), 18.4 (C), 18.3 (C), -2.8 (CH_3), -3.4 (CH_3), -3.9 (CH_3), -4.3 (CH_3).
IR (neat): 2930, 2886, 2853, 1738, 1730, 1470, 1463, 1408, 1389, 1361, 1257, 1174 cm^{-1}.
LRMS (ESI): *m/z* calcd for $C_{28}H_{50}NaO_6Si_2$ $[M+Na]^+$: 561.3038; found: 561.3.

For olefin side product **6:**
R_f = 0.28 (EA/Hex = 1:9).
^{1}H NMR (600 MHz, $CDCl_3$): δ 5.76 (dd, J = 8.4, 3.6 Hz, 1H), 4.19 (d, J = 7.2 Hz, 1H), 3.47 (ddd, J = 11.4, 7.2, 4.2 Hz, 1H), 3.30 (s, 3H), 3.29 (s, 3H), 3.00 (d, J = 4.8 Hz, 1H), 2.86-2.80 (m, 2H), 2.40-2.29 (m, 4H), 2.12 (ddd, J = 15.0, 8.4, 4.2 Hz, 1H), 1.41 (dd, J = 13.8, 3.6 Hz, 1H), 0.92 (s, 9H), 0.91 (s, 9H), 0.10 (s, 3H), 0.08 (s, 3H), 0.07 (s, 3H), -0.04 (s, 3H).
^{13}C NMR (150 MHz, $CDCl_3$): δ 214.1 (C), 204.1 (C), 133.6 (C), 126.1 (CH), 95.7 (C), 76.3 (CH), 75.5 (CH), 64.6 (C), 52.6 (CH), 50.2 (CH_3), 49.2 (CH_3), 46.1 (CH_2), 42.5 (CH), 31.8 (CH), 31.3 (CH_2), 29.7 (CH_2), 28.4 (CH_2), 26.3 (CH_3), 26.3 (CH_3), 18.4 (C), 18.0 (C), -3.0 (CH_3), -3.5 (CH_3), -3.6 (CH_3), -4.1 (CH_3).
IR (neat): 2930, 2890, 2861, 1747, 1732, 1471, 1464, 1409, 1392, 1361, 1258 cm^{-1}.
LRMS (ESI): *m/z* calcd for $C_{28}H_{48}NaO_6Si_2$ $[M+Na]^+$: 559.2882; found: 559.3.

Thiol-Mediated Acyl Radical Reaction on Precursor 7

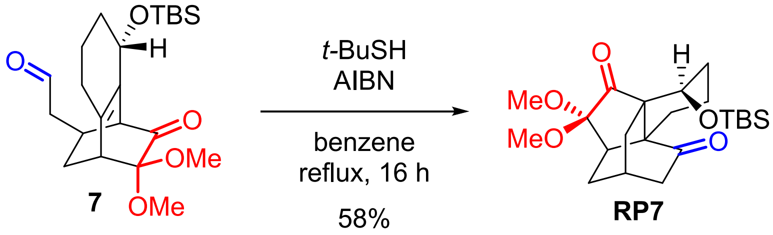

Following the standard procedure, aldehyde **7** (80 mg, 0.20 mmol), *t*-BuSH (88 µL, 0.80 mmol), and AIBN (32 mg, 0.20 mmol) in benzene (1.0 mL) were used to afford rearranged product **RP7** (46 mg, 0.11 mmol, 58%) as a colorless oil.

R_f = 0.25 (EA/Hex = 1:5).
^{1}H NMR (600 MHz, $CDCl_3$): δ 4.01 (dd, J = 2.4, 2.4 Hz, 1H), 3.36 (s, 3H), 3.34 (s, 3H), 2.39 (dd, J = 18.0, 2.4, 2.4 Hz, 1H), 2.26-2.14 (m, 5H), 2.10 (ddd, J = 13.8, 2.4, 2.4 Hz, 1H), 1.72-1.66 (m, 2H), 1.63-1.52 (m, 3H), 1.33-1.23 (m, 2H), 0.86 (s, 9H), 0.06 (s, 3H), 0.06 (s, 3H).
^{13}C NMR (150 MHz, $CDCl_3$): δ 212.2 (C), 210.1 (C), 101.9 (C), 68.7 (CH), 57.6 (C), 50.3 (CH_3), 50.2 (CH_3), 48.4 (C), 45.9 (CH_2), 44.5 (CH), 33.5 (CH_2), 30.1 (CH_2), 27.9

(CH_2), 26.4 (CH), 25.8 (CH_3), 25.0 (CH_2), 18.1 (C), 16.2 (CH_2), -4.2 (CH_3), -4.9 (CH_3).
HRMS (ESI): *m/z* calcd for $C_{22}H_{36}NaO_5Si$ $[M+Na]^+$: 431.2224; found 431.2227.

Thiol-Mediated Acyl Radical Reaction on Precursor 8

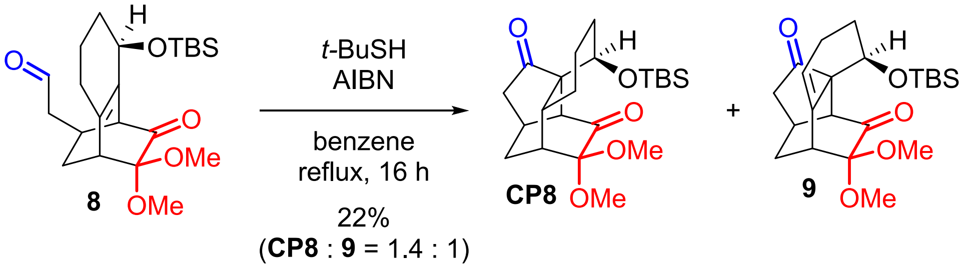

Following the standard procedure, aldehyde **8** (136 mg, 0.333 mmol), *t*-BuSH (150 μL, 1.36 mmol), and AIBN (109 mg, 0.666 mmol) in benzene (1.7 mL) were used to afford a mixture of cyclized product **CP8** and olefin side product **9** (1.4:1.0, 30 mg, 22%).

For cyclized product **CP8**:
$\mathbf{R_f}$ = 0.35 (EA/Hex = 1:5).
^{1}H NMR (600 MHz, $CDCl_3$): δ 3.97-3.93 (m, 1H), 3.30 (s, 3H), 3.24 (s, 3H), 2.90-2.88 (m, 1H), 2.80-2.71 (m, 1H), 2.43-2.32 (m, 2H), 2.32-2.02 (m, 5H), 1.69-1.62 (m, 2H), 1.60-1.47 (m, 2H), 1.36-1.25 (m, 1H), 0.83 (s, 9H), 0.01 (s, 3H), -0.03 (s, 3H).
^{13}C NMR (150 MHz, $CDCl_3$): δ 220.1 (C), 205.9 (C), 95.7 (C), 71.3 (CH), 61.0 (C), 52.0 (CH), 49.3 (CH_3), 48.9 (CH_3), 48.0 (CH_2), 41.8 (CH), 38.0 (CH), 31.6 (CH_2), 30.7 (CH), 28.5 (CH), 26.0 (CH_3), 24.3 (CH_2), 19.8 (CH_2), 18.0 (C), -4.5 (CH_3), -4.9 (CH_3).

For olefin side product **9**:
$\mathbf{R_f}$ = 0.35 (EA/Hex = 1:5).
^{1}H NMR (600 MHz, $CDCl_3$) δ 5.78-5.76 (m, 1H), 3.90-3.87 (m, 1H), 3.31 (s, 3H), 3.29 (s, 3H), 2.80-2.71 (m, 2H), 2.66-2.56 (m, 2H), 2.53-2.46 (m, 2H), 2.32-2.02 (m, 3H), 2.01-1.94 (m, 2H), 1.92-1.84 (m, 2H), 1.36-1.25 (m, 1H), 0.84 (s, 9H), 0.03 (s, 3H), 0.02 (s, 3H).
^{13}C NMR (150 MHz, $CDCl_3$): δ 214.5 (C), 204.3 (C), 129.1 (C), 128.2 (CH), 95.6 (C), 68.7 (CH), 61.8 (C), 56.1(CH), 50.2 (CH_3), 49.2 (CH_3), 48.3 (CH_2), 42.8 (CH), 30.9 (CH), 28.4 (CH_2), 27.0 (CH_2), 26.0 (CH_3), 20.8 (CH_2), 18.2 (C), -4.4 (CH_3), -4.9 (CH_3).

Thiol-Mediated Acyl Radical Reaction on Precursor 10

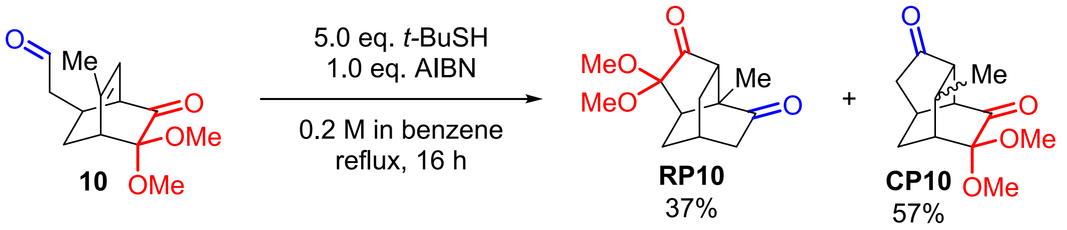

Following the standard procedure, aldehyde **10** (0.75 g, 3.2 mmol), *t*-BuSH (1.80 mL, 15.8 mmol), and AIBN (0.52 g, 3.2 mmol) in benzene (16.0 mL) were used to afford rearranged product **RP10** (0.28 g, 1.2 mmol, 37%), and a diastereomeric mixture of cyclized products **CP10** (0.43 g, 1.8 mmol, 57%).

For rearrangement product **RP10:**
$\mathbf{R_f}$ = 0.38 (EA/Hex = 1:2).
^{1}H NMR (300 MHz, $CDCl_3$) δ 3.37 (s, 3H), 3.31 (s, 3H), 2.43-2.31 (m, 3H), 2.28-2.17 (m, 3H), 2.02-1.96 (m, 1H), 1.86-1.76 (m, 2H), 1.18 (s, 3H).
^{13}C NMR (75 MHz, $CDCl_3$) δ 213.5 (C), 210.5 (C), 101.3 (C), 51.2 (CH), 50.3 (CH_3), 50.1 (CH_3), 46.7 (C), 46.3 (CH_2), 45.7 (CH), 31.0 (CH_2), 27.6 (CH_2), 26.0 (CH), 18.2 (CH_3).
IR (neat) 2942, 2878, 2836, 1756, 1719, 1454, 1385, 1340, 1219, 1136, 1074, 1055, 1007, 995, 978, 926, 914 cm^{-1}.
HRMS (ESI): *m/z* calcd for $C_{13}H_{18}NaO_4$ $[M+Na]^+$: 261.1103; found 261.1106.

For the diastereomeric mixture of cyclized products **CP10:**
$\mathbf{R_f}$ = 0.38 (EA/Hex = 1:2).
For the major diastereomer:
^{1}H NMR (300 MHz, $CDCl_3$) δ 3.34 (s, 3H), 3.29 (s, 3H), 2.83-2.75 (m, 1H), 2.69-2.62 (m, 1H), 2.60-2.51 (m, 1H), 2.33-2.19 (m, 4H), 2.08-1.94 (m, 1H), 1.30-1.20 (m, 1H, overlapping), 1.26 (d, *J* = 7.6 Hz, 3H, overlapping).
^{13}C NMR (75 MHz, $CDCl_3$) δ 216.1 (C), 205.9 (C), 95.2 (C), 55.4 (CH), 50.6 (CH), 49.3 (CH_3), 49.1 (CH_3), 46.8 (CH_2), 38.3 (CH), 34.8 (CH), 31.6 (CH), 25.6 (CH_2), 21.0 (CH_3).
For the minor diastereomer:
^{1}H NMR (300 MHz, $CDCl_3$) δ 3.35 (s, 3H), 3.34 (s, 3H), 2.83-2.75 (m, 1H), 2.69-2.62 (m, 1H), 2.60-2.47 (m, 1H), 2.51-2.33 (m, 2H), 2.33-2.19 (m, 1H), 2.14 (d, *J* = 18.0 Hz, 1H), 2.08-1.94 (m, 1H), 1.60-1.49 (m, 1H), 1.06 (d, *J* = 6.6 Hz, 3H).
^{13}C NMR (75 MHz, $CDCl_3$) δ 217.0 (C), 206.6 (C), 95.9 (C), 52.4 (CH), 51.3 (CH), 49.9 (CH_3), 49.7 (CH_3), 47.6 (CH_2), 40.5 (CH), 32.8 (CH), 31.7 (CH_2), 28.7 (CH), 15.8 (CH_3).

Thiol-Mediated Acyl Radical Reaction on Precursor 11

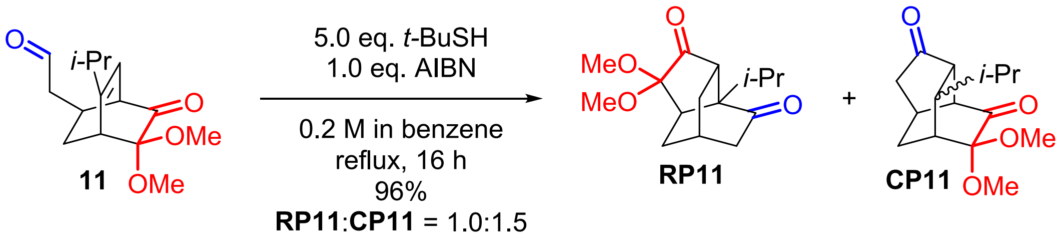

Following the standard procedure, aldehyde **11** (0.77 g, 2.9 mmol), *t*-BuSH (1.63 mL, 14.5 mmol), and AIBN (0.50 g, 2.9 mmol) in benzene (14.5 mL) were used to afford a mixture of rearranged product **RP11** and the minor diastereomer of cyclized products **CP11** (626 mg, 2.35 mmol, 81%), and the major diastereomer of cyclized products **CP11** (114 mg, 0.428 mmol, 15%).

For the mixture of rearranged product **RP11** and the minor cyclized product **CP11:**
$\mathbf{R_f}$ = 0.38 (EA/Hex = 1:2).
For rearranged product **RP11**:
^{1}H NMR (300 MHz, $CDCl_3$) δ 3.35 (s, 3H), 3.31 (s, 3H), 2.86-2.69 (m, 2H), 2.52-1.36 (m, 8H), 1,17 (d, J = 6.6 Hz, 3H), 1.01 (d, J = 6.9 Hz, 3H).
^{13}C NMR (75 MHz, $CDCl_3$) δ 212.6 (C), 210.7 (C), 100.6 (C), 52.8 (C), 50.4 (CH_3), 50.2 (CH_3), 48.8 (CH), 48.2 (CH_2), 42.9 (CH), 30.8 (CH), 30.8 (CH_2), 27.8 (CH_2), 26.0 (CH), 17.4 (CH_3), 17.3 (CH_3).
For the minor diastereomer of cyclized product **CP11**:
^{1}H NMR (300 MHz, $CDCl_3$) δ 3.36 (s, 3H), 3.30 (s, 3H), 2.86-2.69 (m, 1H), 2.61 (dd, J = 7.8, 3.6 Hz1H), 2.52-1.36 (m, 8H), 1.10 (d, J = 6.3 Hz, 3H), 0.93 (d, J = 6.6 Hz, 3H).
^{13}C NMR (75 MHz, $CDCl_3$) δ 216.7 (C), 206.5 (C), 95.9 (C), 52.8 (CH), 50.8 (CH), 50.3 (CH_3), 49.1 (CH_3), 47.7 (CH_2), 43.0 (CH), 35.9 (CH), 33.8 (CH), 31.8 (CH), 26.5 (CH_2), 21.7 (CH_3), 21.5 (CH_3).

For the major diastereomer of cyclized product **CP11**:
$\mathbf{R_f}$ = 0.38 (EA/Hex = 1:2).
^{1}H NMR (300 MHz, $CDCl_3$) δ 3.38 (s, 3H), 3.26 (s, 3H), 2.85-2.71 (m, 1H), 2.59 (dd, J = 3.9, 3.9 Hz, 1H), 2.54-2.45 (m, 2H), 2.45-2.20 (m, 3H), 2.02-1.83 (m, 1H), 1.32-1.21 (m, 2H), 0.97 (d, J = 7.2 Hz, 3H), 0.96 (d, J = 6.9 Hz, 3H).
^{13}C NMR (75 MHz, $CDCl_3$) δ 214.5 (C), 206.5 (C), 95.3 (C), 51.5 (CH), 50.4 (CH_3), 50.2 (CH), 48.4 (CH_3), 47.1 (CH), 45.8 (CH_2), 35.4 (CH), 32.5 (CH), 32.3 (CH), 31.7 (CH_2), 21.5 (CH_3), 21.1 (CH_3).
IR (neat) 2956, 2871, 2836, 2360, 2340, 1735, 1565, 1455, 1409, 1387, 1368, 1218,

1194, 1123, 1090, 1059, 1042, 1025, 973, 902 cm^{-1}

HRMS (ESI): *m/z* calcd for $C_{15}H_{22}NaO_4$ $[M+Na]^+$: 289.1416; found 289.1402.

Thiol-Mediated Acyl Radical Reaction on Precursor 12

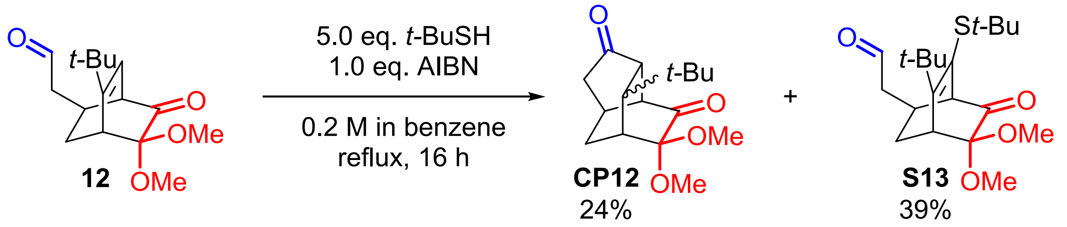

Following the standard procedure, aldehyde **12** (0.18 g, 0.64 mmol), *t*-BuSH (0.36 mL, 3.2 mmol), and AIBN (104 mg, 0.63 mmol) in benzene (14.5 mL) were used to afford a diastereomeric mixture of cyclized products **CP12** (44 mg, 0.16 mmol, 24%), thiol ether side product **S13** (92 mg, 0.25 mmol, 39%), and recovered starting material **12** (23 mg, 0.082 mmol, 13%).

For the diastereomeric mixture of cyclized products **CP12**:

For the major diastereomer:

$\mathbf{R_f}$ = 0.38 (EA/Hex = 1:2).

^{1}H NMR (300 MHz, $CDCl_3$) δ 3.33 (s, 3H), 3.33 (s, 3H), 2.88-2.69 (m, 2H), 2.67-2.48 (m, 2H), 2.37 (dd, *J* = 3.0, 3.0 Hz, 1H), 2.31-2.22 (m, 2H), 2.16 (dd, *J* = 9.0, 1.8 Hz, 1H), 1.64 (ddd, *J* = 14.4, 3.0, 3.0 Hz, 1H, overlapping with H_2O), 1.03 (s, 9H).

^{13}C NMR (75 MHz, $CDCl_3$) δ 219.3 (C), 206.8 (C), 97.3 (C), 54.1 (CH), 51.4 (CH), 50.5 (CH_3), 49.1 (CH_3), 48.3 (CH), 48.2 (CH_2), 35.3 (CH), 34.1 (C), 31.2 (CH), 28.6 (CH_2), 28.5 (CH_3).

For thiol ether side product **S13**:

$\mathbf{R_f}$ = 0.43 (EA/Hex = 1:2).

^{1}H NMR (300 MHz, $CDCl_3$) δ 9.74 (s, 1H), 3.39, (dd, *J* = 3.0, 3.0 Hz, 1H), 3.34-3.27 (m, 1H, overlapping), 3.32 (s, 3H, overlapping), 3.30 (s, 3H, overlapping), 2.76-2.57 (m, 2H), 2.43-2.21 (m, 2H), 1.34 (s, 9H), 1.32 (s, 9H), 0.798 (ddd, *J* = 13.2, 4.5, 3.3 Hz, 1H).

^{13}C NMR (75 MHz, $CDCl_3$) δ 203.4 (C), 200.7 (CH), 159.84 (C), 121.3 (C), 94.8 (C), 62.6 (CH), 50.7 (CH_3), 49.2 (CH_3), 49.1 (C), 48.1 (CH_2), 42.5 (CH), 37.3 (C), 32.2 (CH_3), 31.2 (CH_3), 29.7 (CH), 27.2 (CH_2).

LRMS of methyl hemiacetal form (ESI): *m/z* calcd for $C_{21}H_{36}NaO_5S$ $[M+MeOH+Na]^+$: 423.22; found 423.4.

Thiol-Mediated Acyl Radical Reaction on Precursor 13

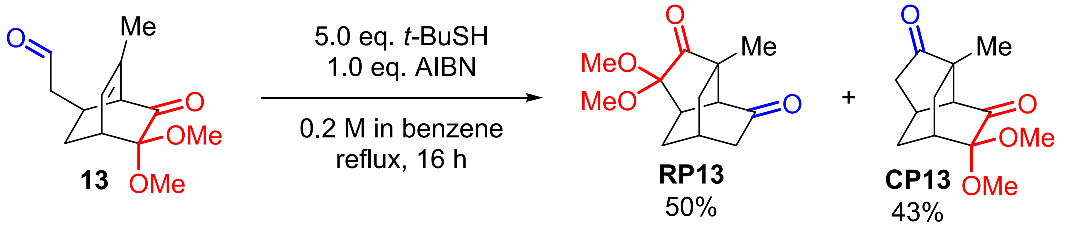

Following the standard procedure, aldehyde **13** (0.40 g, 1.7 mmol), *t*-BuSH (0.95 mL, 8.4 mmol), and AIBN (0.28 g, 1.7 mmol) in benzene (8.4 mL) were used to afford rearranged product **RP13** (0.20 g, 0.84 mmol, 50%), and cyclized product **CP13** (0.17 g, 0.71 mmol, 43%).

For rearranged product **RP13**:

R_f = 0.38 (EA/Hex = 1:2).

^{1}H NMR (300 MHz, $CDCl_3$) δ 3.30 (s, 3H), 3.26 (s, 3H), 2.69-2.58 (m, 2H), 2.32-2.11 (m, 3H), 1.98-1.87 (m, 1H), 1.87-1.69 (m, 2H), 1.44 (dd, *J* = 14.1, 3.0 Hz, 1H), 1.09 (s, 3H).

^{13}C NMR (75 MHz, $CDCl_3$) δ 212.6 (C), 209.7 (C), 102.5 (C), 52.4 (CH), 51.0 (CH_3), 50.2 (CH_3), 48.0 (C), 45.5 (CH_2), 40.5 (CH_2), 38.3 (CH), 26.9 (CH), 25.2 (CH_2), 21.4 (CH_3).

IR (neat) 2946, 2872, 2837, 1756, 1723, 1454, 1405, 1378, 1337, 1227, 1206, 1152, 1114, 1092, 1057, 1038, 982, 969, 924, 900 cm^{-1}

HRMS (ESI): *m/z* calcd for $C_{13}H_{18}NaO_4$ $[M+Na]^+$: 261.1103; found 261.1109.

For cyclized product **CP13**:

R_f = 0.38 (EA/Hex = 1:2).

^{1}H NMR (300 MHz, $CDCl_3$) δ 3.34 (s, 3H), 3.34 (s, 3H), 2.86-2.72 (m, 1H), 2.46-2.24 (m, 5H), 1.90 (ddd, *J* = 14.7, 2.4, 2.4 Hz, 1H), 1.73 (dd, *J* = 14.7, 3.9 Hz, 1H), 1.36 (ddd, *J* = 14.1, 3.6, 1.8 Hz, 1H), 1.06 (s, 3H).

^{13}C NMR (75 MHz, $CDCl_3$) δ 218.0 (C), 206.2 (C), 95.4 (C), 57.1 (CH), 49.8 (CH_3), 49.6 (CH_3), 49.3 (C), 46.1 (CH_2), 33.7 (CH), 33.0 (CH_2), 30.6 (CH), 28.9 (CH_2), 20.2 (CH_3).

IR (neat) 2951, 2922, 2868, 2842, 2360, 2341, 1738, 1728, 1447, 1132, 1115, 1083, 1068, 1049, 1016, 998, 946 cm^{-1}

HRMS (ESI): *m/z* calcd for $C_{13}H_{18}NaO_4$ $[M+Na]^+$: 261.1103; found 261.1107.

Thiol-Mediated Acyl Radical Reaction on Precursor 14

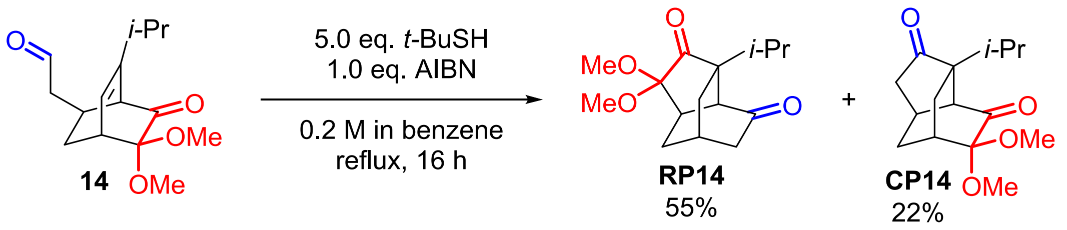

14 → RP14 55% + CP14 22%

Following the standard procedure, aldehyde **14** (0.13 g, 0.49 mmol), *t*-BuSH (0.28 mL, 2.5 mmol), and AIBN (80 mg, 0.49 mmol) in benzene (2.5 mL) were used to afford rearranged product **RP14** (72 mg, 0.27 mmol, 55%), and cyclized product **CP14** (28 mg, 0.11 mmol, 22%).

For rearranged product **RP14**:

$\mathbf{R_f}$ = 0.38 (EA/Hex = 1:2).

^{1}H NMR (300 MHz, $CDCl_3$) δ 3.28 (s, 3H), 3.25 (s, 3H), 2.94 (d, J = 3.9 Hz, 1H), 2.58 (ddd, J = 10.5, 3.9, 3.0 Hz, 1H), 2.31-2.10 (m, 3H), 1.93 (qq, J = 6.9 Hz, 1H), 1.87-1.64 (m, 4H), 0.86 (d, J = 6.9 Hz, 3H, overlapping), 0.84 (d, J = 6.9 Hz, 3H, overlapping).

^{13}C NMR (75 MHz, $CDCl_3$) δ 214.0 (C), 212.1 (C), 102.9 (C), 55.8 (C), 50.8 (CH_3), 50.2 (CH_3), 47.0(CH), 45.4 (CH_2), 38.6 (CH), 37.6 (CH_2), 32.0 (C), 26.7 (CH), 25.4 (CH_2), 17.5 (CH_3), 17.4(CH_3).

IR (neat) 2946, 2880, 2836, 1750, 1721, 1471, 1404, 1390, 1372, 1337, 1281, 1223, 1153, 1090, 1062, 1041, 1006, 931, 906 cm^{-1}

HRMS (ESI): *m/z* calcd for $C_{15}H_{22}NaO_4$ $[M+Na]^+$: 289.1416; found 289.1419.

For cyclized product **CP14**:

$\mathbf{R_f}$ = 0.38 (EA/Hex = 1:2).

^{1}H NMR (400 MHz, $CDCl_3$) δ 3.36 (s, 3H), 3.31 (s, 3H), 2.81-2.68 (m, 2H), 2.36-2.19 (m, 5H), 1.90 (qq, J = 6.8, 6.8 Hz, 1H), 1.67 (dd, J = 14.8, 4.0 Hz, 1H), 1.41-1.14 (m, 1H), 0.88 (d, J = 6.8 Hz, 3H), 0.84 (d, J = 6.8 Hz, 3H).

^{13}C NMR (75 MHz, $CDCl_3$) δ 220.4 (C), 207.5 (C), 95.2 (C), 57.2 (C), 52.4 (CH), 49.9 (CH_3), 49.2 (CH_3), 48.3 (CH_2), 33.7 (CH), 31.4 (CH), 30.9 (CH), 30.7 (CH_2), 28.3 (CH_2), 17.6 (CH_3), 17.4 (CH_3).

IR (neat) 2963, 2945, 2879, 2835, 2360, 1738, 1732, 1565, 1470, 1410, 1391, 1362, 1332, 1284, 1132, 1097, 1058, 1031, 1003, 975, 923, 894 cm^{-1}

HRMS (ESI): *m/z* calcd for $C_{15}H_{22}NaO_4$ $[M+Na]^+$: 289.1416; found 289.1414.

Thiol-Mediated Acyl Radical Reaction on Precursor 15

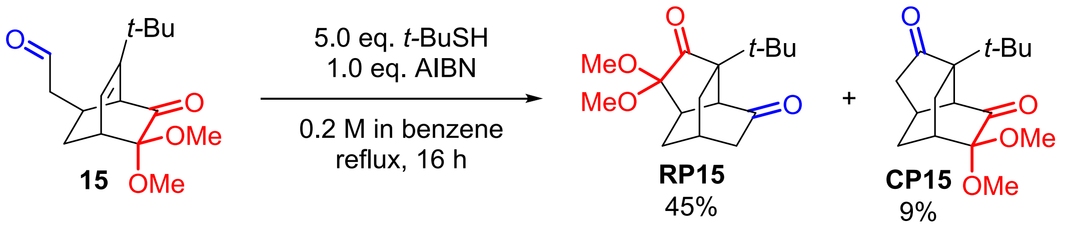

Following the standard procedure, aldehyde **15** (0.70 g, 2.5 mmol), *t*-BuSH (1.41 mL, 12.5 mmol), and AIBN (0.41 g, 2.5 mmol) in benzene (12.5 mL) were used to afford rearranged product **RP15** (313 mg, 1.12 mmol, 45%), cyclized product **CP15** (63 mg, 0.22 mmol, 9%), and recovered starting material **15** (145 mg, 0.517 mmol, 21%).

For rearranged product **RP15**:
R_f = 0.38 (EA/Hex = 1:2).
^{1}H NMR (300 MHz, $CDCl_3$) δ 3.26 (s, 3H), 3.24 (s, 3H), 3.03 (d, *J* = 3.9 Hz, 1H), 2.53 (ddd, *J* = 9.9, 3.6, 3.6 Hz, 1H), 2.32-2.08 (m, 3H), 1.97-1.61 (m, 4H), 0.92 (s, 9H).
^{13}C NMR (75 MHz, $CDCl_3$) δ 214.6 (C), 212.5 (C), 102.7 (C), 57.3 (C), 50.7 (CH_3), 50.1 (CH_3), 48.6 (CH), 45.4 (CH_2), 38.4 (CH), 36.0 (C), 35.8 (CH_2), 26.6 (CH), 25.5 (CH_3), 25.0 (CH_2).
IR (neat) 2953, 2879, 2837, 2360, 2341, 1748, 1722, 1565, 1472, 1399, 1369, 1337, 1280, 1243, 1223, 1154, 1098, 1060, 1034, 999, 984, 902 cm^{-1}
HRMS (ESI): *m/z* calcd for $C_{16}H_{24}NaO_4$ $[M+Na]^+$: 303.1572; found 303.1575.

For cyclized product **CP15**:
R_f = 0.38 (EA/Hex = 1:2).
^{1}H NMR (300 MHz, $CDCl_3$) δ 3.35 (s, 3H), 3.31 (s, 3H), 2.81 (d, *J* = 3.9 Hz, 1H), 2.75-2.63 (m, 1H), 2.42-2.31 (m, 1H), 2.31-2.16 (m, 4H), 1.73 (dd, *J* = 14.7, 4.5 Hz, 1H), 1.27-1.10 (m, 1H), 0.92 (s, 9H).
^{13}C NMR (75 MHz, $CDCl_3$) δ 221.1 (C), 208.0 (C), 95.2 (C), 58.7 (C), 54.6 (CH), 50.0 (CH_3), 49.3 (CH_2), 49.1 (CH_3), 35.3 (C), 33.7 (CH), 31.0 (CH), 29.4 (CH_2), 27.6 (CH_2), 25.4 (CH_3).
IR (neat) 2957, 2939, 2875, 2831, 2360, 1733, 1416, 1398, 1369, 1179, 1136, 1099, 1055, 1046, 1017, 992, 977, 924, 885 cm^{-1}
HRMS (ESI): *m/z* calcd for $C_{16}H_{24}NaO_4$ $[M+Na]^+$: 303.1572; found 303.1572.

Thiol-Mediated Acyl Radical Reaction on Precursor 16

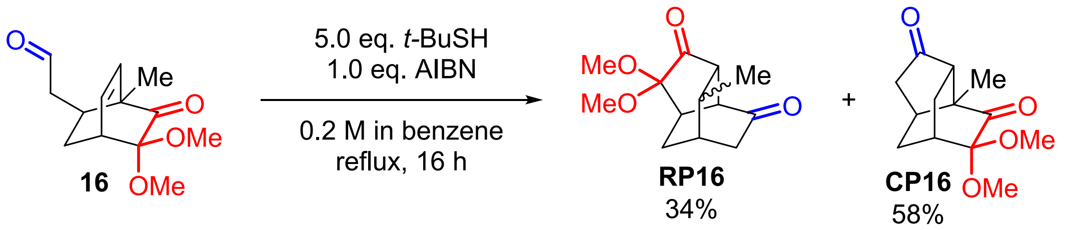

Following the standard procedure, aldehyde **16** (0.17 g, 0.71 mmol), *t*-BuSH (0.40 mL, 3.6 mmol), and AIBN (0.12 g, 0.71 mmol) in benzene (3.6 mL) were used to afford a diastereomeric mixture of rearranged products **RP16** (57 mg, 0.24 mmol, 34%), and cyclized product **CP16** (98 mg, 0.41 mmol, 58%).

For the major diastereomer of the mixture of rearranged products **RP16**:
R_f = 0.38 (EA/Hex = 1:2).
^{1}H NMR (300 MHz, $CDCl_3$) δ 3.29 (s, 3H), 3.29 (s, 3H), 2.88 (dd, *J* = 4.2, 4.2 Hz, 1H), 2.67-2.55 (m, 2H), 2.35-2.25 (m, 2H), 2.16-1.99 (m, 2H), 1.98-1.85 (m, 1H), 1.84-1.70 (m, 1H), 0.99 (d, *J* = 7.2 Hz, 3H).
^{13}C NMR (75 MHz, $CDCl_3$) δ 213.0 (C), 208.4 (C), 101.6 (C), 50.8 (CH_3), 50.2 (CH_3), 48.8 (CH), 47.1 (CH), 47.0 (CH_2), 40.0 (CH), 35.7 (CH), 33.4 (CH), 22.1 (CH_2), 16.3 (CH_3).

For cyclized product **CP16**:
R_f = 0.38 (EA/Hex = 1:2).
Mp: 85.0 – 86.4 °C
^{1}H NMR (300 MHz, $CDCl_3$) δ 3.34 (s, 3H), 3.33 (s, 3H), 2.62-2.49 (m, 1H), 2.49-2.14 (m, 6H), 1.66 (ddd, *J* = 14.4, 3.9, 1.8 Hz, 1H, overlapping with H_2O), 1.57-1.42 (m, 1H), 1.05 (s, 3H).
^{13}C NMR (75 MHz, $CDCl_3$) δ 217.6 (C), 207.2 (C), 95.2 (C), 51.7 (CH), 50.6 (C), 49.9 (CH_3), 49.2 (CH_3), 44.3 (CH_2), 38.2 (CH), 32.7 (CH), 30.9 (CH_2), 25.1 (CH_2), 16.7 (CH_3).
IR (neat) 2971, 2942, 2877, 2835, 1747, 1728, 1458, 1418, 1382, 1197, 1105, 1058, 1043, 990, 975, 934, 918 cm^{-1}
HRMS (ESI): *m/z* calcd for $C_{13}H_{18}NaO_4$ $[M+Na]^+$: 261.1103; found 261.1109.

Thiol-Mediated Acyl Radical Reaction on Precursor 17

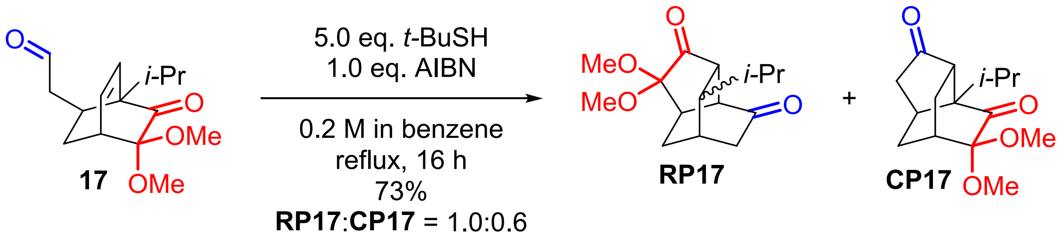

Following the standard procedure, aldehyde **17** (0.14 g, 0.53 mmol), *t*-BuSH (0.30 mL, 2.7 mmol), and AIBN (87 mg, 0.53 mmol) in benzene (2.7 mL) were used to afford the major diastereomer of rearranged products **RP17** (66 mg, 0.25 mmol, 47%), and a mixture of cyclized product **CP17** and the minor diastereomer of rearranged products **RP17** (36 mg, 0.14 mmol, 26%).

For the major diastereomer of rearranged products **RP17**:
R_f = 0.38 (EA/Hex = 1:2).
^{1}H NMR (300 MHz, $CDCl_3$) δ 3.30 (s, 3H), 3.29 (s, 3H), 2.86 (dd, *J* = 4.8, 3.9 Hz, 1H), 2.68-2.56 (m, 1H), 2.56-2.49 (m, 1H), 2.40 (ddd, *J* = 19.5, 3.0, 3.0 Hz, 1H), 2.27 (ddddd, *J* = 3.0, 3.0, 3.0, 3.0, 3.0 Hz, 1H), 2.09 (ddd, *J* = 19.5, 3.0, 1.2 Hz, 1H), 1.96-1.78 (m, 2H), 1.45-1.33 (m, 1H), 1.31-1.19 (m, 1H), 1.01 (d, *J* = 6.3 Hz, 3H), 0.90 (d, *J* = 6.3 Hz, 3H).
^{13}C NMR (75 MHz, $CDCl_3$) δ 212.8 (C), 208.4 (C), 102.9 (C), 50.9 (CH_3), 50.4 (CH_3), 49.8 (CH), 49.1 (CH), 45.9 (CH), 39.5 (CH), 39.3 (CH_2), 30.5 (CH), 28.9 (CH), 26.7 (CH_2), 20.8 (CH_3), 20.7 (CH_3).
IR (neat) 2958, 2926, 2855, 1755, 1724, 1463, 1226, 1148, 1122, 1085, 1065, 1042, 968, 879 cm^{-1}.

For the mixture of cyclized product **CP17** and the minor diastereomer of rearranged products **RP17**:
R_f = 0.38 (EA/Hex = 1:2).
For cyclized product **CP17**:
^{1}H NMR (300 MHz, $CDCl_3$) δ 3.30 (s, 3H), 3.28 (s, 3H), 2.90-2.74 (m, 1H), 2.65-2.53 (m, 1H), 2.53-2.33 (m, 3H), 2.33-2.20 (m, 1H), 2.14 (d, *J* = 18.6 Hz, 1H), 1.64 (ddd, *J* = 14.1, 3.9, 1.5 Hz, 1H, overlapping with H_2O), 1.49 (dd, *J* = 14.1, 3.9 Hz, 1H), 1.33 (qq, *J* = 6.6, 6.6 Hz, 1H), 1.12 (d, *J* = 6.6 Hz, 3H), 1.04 (d, *J* = 6.6 Hz, 3H).
^{13}C NMR (75 MHz, $CDCl_3$) δ 217.7 (C), 206.3 (C), 95.6 (C), 56.5 (C), 50.0 (CH_3), 49.6 (CH), 49.4 (CH_3), 43.4 (CH_2), 35.5 (CH), 32.3 (CH), 31.2 (CH_2), 29.9 (CH), 25.2 (CH_2), 17.1 (CH_3), 16.9 (CH_3).

For the minor diastereomer of rearranged products **RP17**:

^{1}H NMR (300 MHz, $CDCl_3$) δ 3.29 (s, 3H), 3.28 (s, 3H), 2.92-2.77 (m, 2H), 2.60 (dddd, J = 10.8, 3.3, 3.3, 3.3 Hz, 1H), 2.40-2.13 (m, 3H), 1.98 (dddd, J = 14.4, 2.7, 2.7, 2.7 Hz, 1H), 1.83-1.70 (m, 1H), 1.55-1.37 (m, 2H), 1.06 (d, J = 5.4 Hz, 3H), 0.99 (d, J = 6.0 Hz, 3H).

^{13}C NMR (75 MHz, $CDCl_3$) δ 213.1 (C), 208.5 (C), 101.5 (C), 50.7 (CH_3), 50.3 (CH), 50.2 (CH_3), 48.0 (CH), 47.5 (CH), 47.3 (CH_2), 39.4 (CH), 28.8 (CH), 28.2 (CH), 22.5 (CH_2), 21.7 (CH_3), 21.5 (CH_3).

Thiol-Mediated Acyl Radical Reaction on Precursor 18

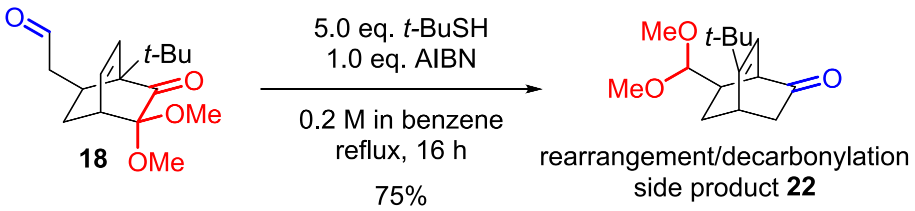

Following the standard procedure, aldehyde **18** (0.11 g, 0.39 mmol), *t*-BuSH (0.22 mL, 2.0 mmol), and AIBN (64 mg, 0.39 mmol) in benzene (2.0 mL) were used to afford rearrangement/decarbonylation side product **22** (74 mg, 0.29 mmol, 75%).

^{1}H NMR (300 MHz, $CDCl_3$) δ 5.64 (dd, J = 6.6, 2.4 Hz, 1H), 3.96 (d, J = 8.7 Hz, 1H), 3.30 (s, 3H), 3.26 (s, 3H), 3.20 (dd, J = 6.6, 1.8 Hz, 1H), 3.06 (dddd, J = 2.4, 2.4, 2.4, 2.4 Hz, 1H), 2.43-2.28 (m, 1H), 2.07 (dd, J = 18.3, 2.4 Hz, 1H), 2.00-1.82 (m, 2H), 1.30-1.19 (m, 1H), 1.07 (s, 9H).

^{13}C NMR (100 MHz, $CDCl_3$) δ 212.6 (C), 159.0 (C), 113.9 (CH), 105.6 (CH), 52.9 (CH_3), 52.1 (CH_3), 50.4 (CH), 40.1 (CH_2), 37.00 (CH), 35.1 (C), 33.3 (CH), 28.9 (CH_2), 27.6 (CH_3).

LRMS (ESI): *m/z* calcd for $C_{15}H_{24}NaO_3$ $[M+Na]^+$: 275.1618; found 275.1.

Thiol-Mediated Acyl Radical Reaction on Precursor 19

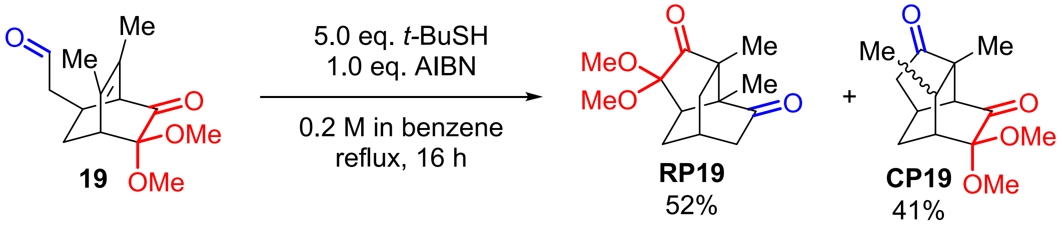

Following the standard procedure, aldehyde **19** (0.44 g, 1.7 mmol), *t*-BuSH (0.98 mL, 8.7 mmol), and AIBN (0.29 g, 1.7 mmol) in benzene (8.7 mL) were used to afford rearranged product **RP19** (0.23 g, 0.91 mmol, 52%), and cyclized product **CP19** (0.18 g, 0.71 mmol, 41%).

For rearranged product **RP19**:

R$_f$ = 0.38 (EA/Hex = 1:2).

Mp: 51.4 – 55.1 °C

^{1}H NMR (400 MHz, $CDCl_3$) δ 3.34 (s, 3H), 3.32 (s, 3H), 2.36 (ddd, *J* = 18.0, 2.4, 2.4 Hz, 1H), 2.32-2.27 (m, 1H), 2.27-2.10 (m, 3H), 1.97 (ddd, *J* = 14.4, 3.2, 3.2 Hz, 1H), 1.73 (ddd, *J* = 13.2, 3.2, 3.2 Hz, 1H), 1.49 (ddd, *J* = 14.4, 3.2, 2.0 Hz, 1H), 1.05 (s, 3H), 0.94 (s, 3H).

^{13}C NMR (75 MHz, $CDCl_3$) δ 213.5 (C), 210.8 (C), 101.5 (C), 52.2 (C), 50.4 (C), 50.2 (CH_3), 50.1 (CH_3), 46.0 (CH_2), 45.1 (CH), 39.1 (CH_2), 27.4 (CH_2), 26.5 (CH), 18.1 (CH_3), 15.5 (CH_3).

IR (neat) 2973, 2942, 2836, 1754, 1722, 1715, 1462, 1454, 1407, 1387, 1343, 1268, 1218, 1205, 1134, 1116, 1102, 1059, 1030, 987, 960, 929, 907 cm^{-1}

HRMS (ESI): *m/z* calcd for $C_{14}H_{20}NaO_4$ $[M+Na]^+$: 275.1259; found 275.1263.

For cyclized product **CP19**:

R$_f$ = 0.38 (EA/Hex = 1:2).

^{1}H NMR (400 MHz, $CDCl_3$) δ 3.34 (s, 3H), 3.29 (s, 3H), 2.80-2.69 (m, 1H), 2.53-2.31 (m, 3H), 2.29 (d, *J* = 4.0 Hz, 1H), 2.22 (ddd, J = 4.0, 2.0, 2.0 Hz, 1H), 1.96 (qd, *J* = 7.6, 2.0 Hz, 1H), 1.36 (ddd, *J* = 13.6, 4.0, 1.2 Hz, 1H), 1.14 (d, *J* = 7.6 Hz, 3H), 1.01 (s, 3H).

^{13}C NMR (75 MHz, $CDCl_3$) δ 216.6 (C), 206.0 (C), 95.6 (C), 57.6 (CH), 52.6 (C), 49.3 (CH_3), 49.2 (CH_3), 46.0 (CH_2), 39.9 (CH), 36.1 (CH), 32.1 (CH_2), 30.3 (CH), 15.9 (CH_3), 14.7 (CH_3).

IR (neat) 2951, 2937, 2873, 2837, 1729, 1464, 1404, 1384, 1334, 1285, 1272, 1256, 1225, 1198, 1176, 1133, 1120, 1093, 1085, 1044, 1024, 1007, 954 cm^{-1}

HRMS (ESI): *m/z* calcd for $C_{14}H_{20}NaO_4$ $[M+Na]^+$: 275.1259; found 275.1259.

Thiol-Mediated Acyl Radical Reaction on Precursor 20

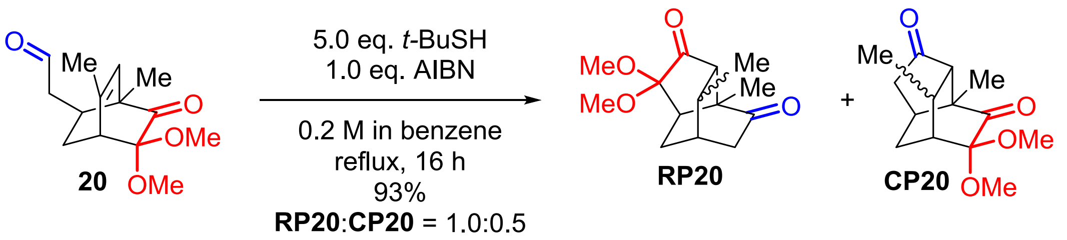

Following the standard procedure, aldehyde **20** (0.60 g, 2.4 mmol), *t*-BuSH (1.35 mL, 12.0 mmol), and AIBN (0.39 g, 2.4 mmol) in benzene (12.0 mL) were used to afford a mixture of rearranged products **RP20** and cyclized products **CP20** (0.56 g, 2.2 mmol, 93%).

For the mixture of rearranged products **RP20** and cyclized products **CP20**:
R_f = 0.38 (EA/Hex = 1:2).
For the major diastereomer of rearranged products **RP20**:
^{1}H NMR (300 MHz, $CDCl_3$) δ 3.37 (s, 3H), 3.30 (s, 3H), 2.47-2.25 (m, 5H), 2.17-2.08 (m, 1H), 1.97-1.87 (m, 1H), 1.77 (dddd, J = 14.1, 10.2, 1.8, 1.8 Hz, 1H) 1.15 (s, 3H), 1.01 (d, J = 7.5 Hz, 3H).
^{13}C NMR (75 MHz, $CDCl_3$) δ 213.8 (C), 210.5 (C), 100.5 (C), 57.1 (CH), 50.2 (CH_3), 50.0 (CH_3), 48.0 (C), 47.7 (CH_2), 45.6 (CH), 35.8 (CH), 32.8 (CH), 24.4 (CH_2), 18.5 (CH_3), 16.9 (CH_3).

For the major diastereomer of cyclized products **RP20**:
R_f = 0.38 (EA/Hex = 1:2).
^{1}H NMR (300 MHz, $CDCl_3$) δ 3.34 (s, 3H), 3.28 (s, 3H), 2.65-2.40 (m, 3H), 2.25-2.16 (m, 2H), 2.08-1.96 (m, 1H), 1.96-1.87 (m, 1H), 1.40 (dd, J = 14.4, 3.6 Hz, 1H), 1.26 (d, J = 7.2 Hz, 3H), 1.05 (s, 3H).

Thiol-Mediated Acyl Radical Reaction on Precursor 21

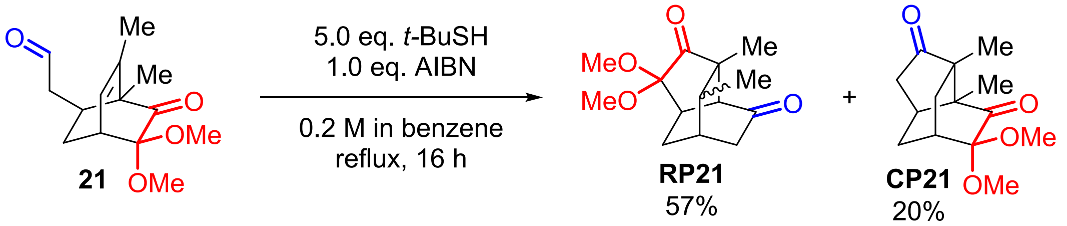

Following the standard procedure, aldehyde **21** (0.14 g, 0.55 mmol), *t*-BuSH (0.31 mL, 2.8 mmol), and AIBN (90 mg, 0.55 mmol) in benzene (2.8 mL) were used to afford rearranged product **RP21** (80 mg, 0.32 mmol, 57%), and cyclized product **CP21** (28 mg, 0.11 mmol, 20%).

For rearranged product **RP21**:
R_f = 0.38 (EA/Hex = 1:2).
^{1}H NMR (300 MHz, $CDCl_3$) δ 3.30(s, 3H), 3.26 (s, 3H), 2.65-2.48 (m, 2H), 2.39-2.19 (m, 2H), 2.10-1.96 (m, 1H), 1.97-1.87 (m, 1H), 1.81-1.65 (m, 1H), 1.63-1.50 (m, 1H), 1.04 (s, 3H), 0.90 (d, J = 7.2 Hz, 3H).
^{13}C NMR (75 MHz, $CDCl_3$) δ 212.8 (C), 209.6 (C), 102.0 (C), 53.5 (CH), 52.1 (C), 50.8 (CH_3), 50.2 (CH_3), 47.0 (CH_2), 44.3 (CH), 38.6 (CH), 33.8 (CH), 21.6 (CH_2), 21.0 (CH_3), 15.2 (CH_3).
IR (neat) 2963, 2837, 1750, 1718, 1455, 1405, 1385, 1356, 1320, 1226, 1212, 1188, 1154, 1145, 1121, 1092, 1079, 1057, 1036, 967, 948 938, 916cm^{-1}

HRMS (ESI): *m/z* calcd for $C_{14}H_{20}NaO_4$ $[M+Na]^+$: 275.1259; found 275.1261.

For cyclized product **CP21**:

$\mathbf{R_f}$ = 0.38 (EA/Hex = 1:2).

^{1}**H NMR** (300 MHz, $CDCl_3$) δ 3.33 (s, 3H), 3.32 (s, 3H), 2.62-2.48 (m, 1H), 2.48-2.21 (m, 4H), 1.94 (ddd, *J* = 15.0, 2.4, 2.4 Hz, 1H), 1.75 (dd, *J* = 15.0, 3.6 Hz, 1H), 1.49 (dd, *J* = 12.9, 4.8 Hz, 1H), 0.93 (s, 3H), 0.92 (s, 3H).

^{13}**C NMR** (75 MHz, $CDCl_3$) δ 217.5 (C), 207.5 (C), 95.3 (C), 53.9 (C), 52.5 (C), 49.8 (CH_3), 49.4 (CH_3), 44.5 (CH_2), 37.1 (CH), 33.3 (CH), 33.1 (CH_2), 30.8 (CH_2), 17.0 (CH_3), 14.3 (CH_3).

IR (neat) 2970, 2943, 2835, 1743, 1724, 1448, 1386, 1373, 1246, 1196, 1158, 1138, 1040, 998, 979, 952, 922 cm^{-1}

HRMS (ESI): *m/z* calcd for $C_{14}H_{20}NaO_4$ $[M+Na]^+$: 275.1259; found 275.1255.

3. Computational Details

All calculations were performed using Jaguar. Geometries were optimized with the B3LYP functional combined with the 6-31G** basis set to obtain electronic energies. All calculations included the D3 correction. The electronic energies are presented in the table below, with all values expressed in Hartree units.

Species	E_{elec}
7	-1525.77466
8	-1525.77177
CP7	-1525.79537
RP7	-1525.80785
CP8	-1525.800415
RP8	-1525.798618
16	-807.10308
18	-925.04722
CP16	-807.14120
RP16	-807.14116
CP18	-925.08437
RP18	-925.09552
Methane	-40.52337
Ethane	-79.83898

7

C	2.31160000	1.20580000	-1.69240000
C	2.78070000	-0.18250000	-1.19420000
C	1.03590000	0.79120000	0.46730000
C	1.28730000	1.81490000	-0.68890000
H	3.17010000	1.87230000	-1.80810000
H	1.85900000	1.08110000	-2.68150000
H	1.70870000	2.71800000	-0.23530000
C	3.45270000	-0.00680000	0.19070000
C	2.35870000	0.52240000	1.17340000
H	3.49220000	-0.63980000	-1.88720000
C	0.66740000	-0.55990000	-0.11470000
C	1.55900000	-1.05530000	-0.99160000
C	-0.01760000	2.19960000	-1.39520000
H	0.18940000	2.72890000	-2.33680000
H	-0.57760000	1.29490000	-1.67920000
C	-0.95730000	3.06460000	-0.59570000

O	-0.84600000	3.30810000	0.58850000
O	2.54930000	0.64390000	2.36440000
O	3.95000000	-1.27690000	0.53820000
O	4.45380000	0.99010000	0.22720000
C	4.53620000	-1.40590000	1.83910000
H	5.13120000	-0.52420000	2.09360000
H	3.77140000	-1.54310000	2.60900000
H	5.17880000	-2.28920000	1.79190000
C	5.58000000	0.75410000	-0.61450000
H	5.34280000	0.92540000	-1.67330000
H	6.34780000	1.46680000	-0.30620000
H	5.95970000	-0.26630000	-0.49670000
H	-1.80630000	3.48540000	-1.17830000
C	-0.56370000	-1.29310000	0.33730000
O	-1.72750000	-0.59960000	-0.14920000
Si	-3.09300000	-0.39270000	0.82110000
C	-4.44490000	0.19280000	-0.38490000
C	-3.58050000	-2.03100000	1.62660000
H	-3.85540000	-2.78900000	0.88650000
H	-2.76190000	-2.43650000	2.23130000
H	-4.43720000	-1.88720000	2.29450000
C	-2.73010000	0.86100000	2.17720000
H	-1.92750000	0.49850000	2.83020000
H	-2.41010000	1.82580000	1.77440000
H	-3.61170000	1.02420000	2.80770000
C	-4.02660000	1.52350000	-1.04100000
H	-3.86070000	2.31170000	-0.29780000
H	-3.10660000	1.40410000	-1.62260000
H	-4.80890000	1.87690000	-1.72730000
C	-4.64010000	-0.86870000	-1.48750000
H	-3.70870000	-1.05060000	-2.03370000
H	-4.98200000	-1.82600000	-1.07780000
H	-5.39660000	-0.53470000	-2.21100000
C	-5.77080000	0.39640000	0.37780000
H	-6.11770000	-0.52940000	0.85120000
H	-5.68050000	1.16030000	1.15900000
H	-6.56100000	0.72600000	-0.31080000
H	0.30830000	1.17600000	1.18100000

C	1.37860000	-2.35990000	-1.71060000
H	1.67810000	-2.24740000	-2.76160000
H	2.07290000	-3.09720000	-1.27890000
C	-0.07170000	-2.85170000	-1.60130000
C	-0.55200000	-2.75200000	-0.14870000
H	-0.71820000	-2.22520000	-2.22810000
H	-0.15760000	-3.87970000	-1.96870000
H	0.12140000	-3.32950000	0.49860000
H	-1.55510000	-3.17110000	-0.03240000
H	-0.57040000	-1.28520000	1.43950000

8

C	-3.34310000	0.35560000	-0.95670000
C	-2.78610000	-0.12450000	0.41070000
C	-0.82490000	0.17190000	-1.26230000
C	-2.16450000	0.61660000	-1.94200000
H	-4.00820000	-0.41480000	-1.35060000
H	-3.94870000	1.25310000	-0.80590000
H	-2.30090000	-0.00280000	-2.83550000
C	-2.11530000	-1.50660000	0.18240000
C	-0.96950000	-1.28850000	-0.85420000
H	-3.59910000	-0.22720000	1.13390000
C	-0.65820000	0.93760000	0.03540000
C	-1.70020000	0.82360000	0.88220000
C	-2.08190000	2.08260000	-2.42840000
H	-1.21100000	2.23230000	-3.07380000
H	-2.98430000	2.28780000	-3.02440000
C	-2.04120000	3.13500000	-1.33910000
O	-1.17540000	3.98110000	-1.23550000
O	-0.29020000	-2.20100000	-1.27700000
O	-1.48780000	-2.03240000	1.32860000
O	-3.11340000	-2.36810000	-0.31660000
C	-2.34680000	-2.29250000	2.43700000
H	-2.63120000	-1.36900000	2.95730000
H	-3.25390000	-2.82150000	2.12530000
H	-1.77530000	-2.92120000	3.12260000
C	-2.70890000	-3.70240000	-0.65000000
H	-2.23170000	-3.74000000	-1.63380000

H	-2.01010000	-4.09960000	0.09130000
H	-3.62470000	-4.29860000	-0.65810000
H	-2.89860000	3.12770000	-0.63250000
C	0.58980000	1.75060000	0.31060000
H	0.03130000	0.27860000	-1.92880000
C	-1.78020000	1.56270000	2.18930000
H	-2.79280000	1.96660000	2.32560000
H	-1.62730000	0.85320000	3.01720000
C	-0.72810000	2.68390000	2.25080000
C	0.63990000	2.15850000	1.79090000
H	-1.03550000	3.50820000	1.59500000
H	-0.66220000	3.08710000	3.26690000
H	0.90780000	1.28380000	2.39560000
H	1.42550000	2.90650000	1.93550000
H	0.53760000	2.66320000	-0.29700000
O	1.75050000	1.06460000	-0.14570000
Si	2.73870000	-0.08630000	0.57860000
C	3.81570000	0.71460000	1.91000000
H	4.25150000	1.65240000	1.55130000
H	3.25130000	0.93190000	2.82230000
H	4.64100000	0.04670000	2.18410000
C	3.83000000	-0.70170000	-0.86000000
C	1.76020000	-1.50150000	1.34550000
H	0.89690000	-1.14950000	1.91750000
H	1.37050000	-2.18570000	0.58780000
H	2.40470000	-2.07390000	2.02280000
C	4.55830000	-1.99250000	-0.42870000
H	5.18590000	-1.83710000	0.45810000
H	3.85480000	-2.80170000	-0.20480000
H	5.21830000	-2.34440000	-1.23350000
C	2.95340000	-0.99740000	-2.09510000
H	2.14950000	-1.70920000	-1.88000000
H	2.48910000	-0.08000000	-2.47110000
H	3.56760000	-1.41790000	-2.90420000
C	4.87520000	0.37150000	-1.23010000
H	5.48260000	0.03570000	-2.08240000
H	4.39780000	1.31540000	-1.51590000
H	5.56230000	0.57890000	-0.40160000

CP7

C	-3.21263614	-0.81968709	1.65396889
C	-3.04778102	-0.73661103	0.11658198
C	-0.90888891	0.16858899	1.31435087
C	-1.87004625	-0.45925018	2.36313308
H	-4.00293907	-0.13647322	1.96796997
H	-3.52905926	-1.83226405	1.92934903
C	-2.81957704	0.74400522	-0.27217597
C	-1.52144809	1.21630491	0.43247328
H	-3.95998047	-1.09255522	-0.37312129
C	-0.53988198	-1.08564629	0.46919289
C	-1.84396998	-1.63021836	-0.26448491
C	-1.04719997	-1.68415634	2.81628238
C	-0.25191906	-2.11140135	1.57318893
O	-1.04701423	2.32272828	0.25184498
O	-2.60149198	0.97514508	-1.64649321
O	-3.94056919	1.46101605	0.20711892
C	-3.69994329	0.66673793	-2.50590405
H	-3.47790952	1.14261804	-3.46318304
H	-3.80380121	-0.41351022	-2.66247119
H	-4.63919111	1.06395213	-2.10843901
C	-3.91516815	2.88283118	0.02971912
H	-3.53210203	3.15259939	-0.95838233
H	-4.95116650	3.21396649	0.13359185
H	-3.29323830	3.37012034	0.78734219
C	0.60107603	-0.81951987	-0.52169997
O	1.74164404	-0.36514077	0.18885119
Si	2.60925130	1.05373213	-0.07712917
C	4.42747258	0.49919724	-0.21325314
C	2.00851500	1.90211810	-1.64960688
H	0.98101418	2.26073199	-1.52685523
H	2.63834335	2.77175635	-1.86931599
H	2.04230826	1.23981414	-2.52112530
C	2.34491933	2.19758421	1.39430215
H	2.53479924	1.68066588	2.34110714
H	3.00788846	3.06896028	1.34937235
H	1.31128429	2.55889903	1.39427833
C	4.58197011	-0.44850682	-1.42063930

H	4.33686950	0.05037079	-2.36601128
H	5.61828544	-0.80547180	-1.49934118
H	3.93487947	-1.32638013	-1.32406127
C	5.35627549	1.71651923	-0.39563990
H	5.29367753	2.41093208	0.45024200
H	6.40330136	1.39137315	-0.47280611
H	5.12318561	2.27769742	-1.30856908
C	4.81713913	-0.25964717	1.07299683
H	5.85123826	-0.62524515	1.00429110
H	4.75675524	0.38280788	1.95951213
H	4.16556424	-1.12413017	1.23777998
H	-0.01240180	0.57532680	1.78594623
C	-1.57885536	-1.91931689	-1.75301394
H	-2.44575141	-2.45001605	-2.16581135
H	-1.47214306	-0.99203218	-2.31995592
C	-0.31283583	-2.78350302	-1.88901038
C	0.95137285	-2.04460807	-1.37760925
H	-0.45363984	-3.70049046	-1.30412611
H	-0.18100296	-3.09376123	-2.93064340
H	1.57050917	-1.69825997	-2.21269540
H	1.56451730	-2.70947906	-0.76635185
H	0.24378296	-0.02440301	-1.19052922
H	-0.32010302	-1.40639808	3.58963048
H	-1.63835922	-2.51606319	3.21036808
H	-2.05434228	0.22460399	3.19511720
O	0.42649093	-3.10918040	1.47386924
H	-2.07402715	-2.61260629	0.17053319

RP7

C	-2.35557220	2.28051317	-0.36846189
C	-2.53972906	1.05936628	0.57865479
C	-0.70185784	0.82852700	-1.59300132
C	-0.96386725	2.23663591	-1.03278095
H	-3.13080734	2.28552024	-1.13606629
H	-2.45692922	3.20951240	0.20382426
H	-0.90880583	2.97347025	-1.84155217
C	-3.27922304	-0.12940606	-0.11408903
C	-2.11595333	-0.97668393	-0.71998428

C	-0.81100329	-0.24863499	-0.44555825
C	-1.12264424	0.45098602	0.92289200
C	0.08581403	2.53628940	0.04164784
H	1.10681814	2.39495092	-0.32853705
H	0.01442220	3.56101733	0.42321109
C	-0.11964486	1.57796898	1.20158320
O	0.44409084	1.71398394	2.27148435
O	-2.25441944	-2.03330219	-1.29617734
O	-3.96852033	-0.82616105	0.90028820
O	-4.12980509	0.19622422	-1.18646196
C	-4.54188917	-2.08720313	0.52860309
H	-3.79626625	-2.88741703	0.56789291
H	-5.33271917	-2.28301829	1.25670495
H	-4.95896816	-2.05104921	-0.48150790
C	-5.34453464	0.85100221	-0.81338830
H	-5.84282063	1.11512516	-1.74803809
H	-5.99264236	0.19277610	-0.22613384
H	-5.15977822	1.76388589	-0.23327985
H	-1.41137287	0.60532797	-2.39748145
H	0.30099284	0.77664805	-2.02181005
H	-3.07853416	1.33651229	1.48732304
C	-1.13480632	-0.55476509	2.09091425
H	-1.20954414	0.01092381	3.02434217
H	-2.02651920	-1.18246085	2.02492214
C	0.41331177	-1.17291185	-0.39074924
H	0.47103125	-1.71744794	-1.34388002
C	0.31405611	-2.16860541	0.77238394
H	-0.52317479	-2.84971738	0.57777318
H	1.22571633	-2.77563733	0.78967322
C	0.12111227	-1.43913986	2.10917298
H	0.99597508	-0.81665491	2.31443925
H	0.04385980	-2.16787620	2.92388898
O	1.55737288	-0.33990325	-0.20151810
Si	3.07673813	-0.47442381	-0.90131321
C	4.27384608	0.23095624	0.39867526
C	3.45535228	-2.27561299	-1.31331527
H	2.72660641	-2.68677524	-2.02049928
H	4.44506508	-2.36473543	-1.77503406

H	3.44555721	-2.90566518	-0.41852099
C	3.10594607	0.54938917	-2.48672137
H	4.09375542	0.53056793	-2.96003232
H	2.38377417	0.16392218	-3.21503119
H	2.85067202	1.59559800	-2.28677916
C	4.30942954	-0.70000519	1.62879223
H	3.31482926	-0.82241183	2.06936404
H	4.69538126	-1.69514629	1.37877397
H	4.96496215	-0.28121008	2.40447400
C	5.69235067	0.33924918	-0.20042482
H	5.72588252	1.02647421	-1.05361783
H	6.39523935	0.72095585	0.55265790
H	6.07396473	-0.63292880	-0.53595283
C	3.79348331	1.62968019	0.84285394
H	2.81167219	1.59073909	1.32398613
H	4.49929252	2.05898793	1.56729707
H	3.73601837	2.32892230	-0.00086520

CP8

C	3.59694741	0.07778218	-0.24761103
C	2.52412626	0.45133104	0.80369588
C	1.37948624	-0.52571590	-1.32817616
C	2.93330040	-0.64800083	-1.46207017
H	4.11128335	0.98100096	-0.57707308
H	4.34607348	-0.57285395	0.21781888
C	1.60278211	1.52767122	0.17734317
C	0.96473193	0.90186303	-1.09090313
H	3.00648720	0.87628789	1.68993601
C	1.12094717	-1.47774916	-0.12902400
C	1.76679424	-0.84391124	1.18054523
C	3.12203041	-2.17376542	-1.42834200
C	2.00850230	-2.67572814	-0.50346823
O	0.29022620	1.55055496	-1.86669497
O	0.52908993	1.95946239	0.97849107
O	2.45466910	2.60437123	-0.16882818
C	0.87789924	2.64508930	2.18057593
H	-0.04881078	3.09024908	2.54773498
H	1.26741232	1.95879139	2.94134231

H	1.61614595	3.43130435	1.99412297
C	1.86095816	3.72403409	-0.83660330
H	0.87709118	3.96067528	-0.42203526
H	2.54453398	4.56172220	-0.67470893
H	1.74930123	3.53670852	-1.90842801
C	-0.35686708	-1.86424488	0.05355221
H	0.87403783	-0.88549716	-2.23044718
C	0.75906931	-0.76229517	2.33712296
H	1.28996109	-0.43249815	3.23932307
H	-0.01434176	-0.03069016	2.11177018
C	0.12667180	-2.13979912	2.58044607
C	-0.59572182	-2.69670419	1.32399513
H	0.91569890	-2.84024405	2.88256236
H	-0.57043984	-2.07884531	3.42233850
H	-1.67638907	-2.73450809	1.48382729
H	-0.25680813	-3.71528943	1.12204098
H	2.95079341	-2.62157425	-2.41576293
H	4.10465435	-2.51141225	-1.08611619
H	3.28326648	-0.21595618	-2.40238798
O	1.86150639	-3.82227637	-0.13747390
H	2.54657343	-1.53780602	1.52725793
H	-0.65093723	-2.46633535	-0.82070629
O	-1.11516326	-0.65585699	0.09226311
Si	-2.61699634	-0.43067990	-0.64018699
C	-2.39903723	-0.38035779	-2.50987129
H	-2.08578441	-1.35789105	-2.89504300
H	-3.33636730	-0.11654388	-3.01316648
H	-1.63661611	0.35530125	-2.78070420
C	-3.76051028	-1.86669920	-0.18823205
H	-4.70723325	-1.79122900	-0.73434297
H	-3.30992008	-2.83170313	-0.44522328
H	-3.99197611	-1.88097799	0.88225701
C	-3.28618542	1.20685486	0.06925396
C	-2.59146936	2.42379532	-0.57746309
H	-2.74466935	2.45346734	-1.66196529
H	-3.00742808	3.35311523	-0.16154776
H	-1.51528794	2.42235913	-0.39267227
C	-3.03873738	1.24175516	1.59187735

H	-1.96747625	1.23601623	1.81045190
H	-3.47137100	2.15486532	2.02494490
H	-3.49688105	0.38832402	2.10704040
C	-4.80387424	1.28715698	-0.20682681
H	-5.03003825	1.24939225	-1.27992207
H	-5.35780217	0.47780313	0.28165300
H	-5.20649928	2.23574213	0.17376382

RP8

C	-2.87987837	-0.20516890	2.02893013
C	-2.98125603	-0.14009015	0.47921392
C	-0.38157013	-0.11976392	1.62147688
C	-1.50636707	-0.78096402	2.43697024
H	-3.03616822	0.78709983	2.45165015
H	-3.67165718	-0.85467682	2.42033238
H	-1.33029710	-0.62630615	3.50679624
C	-2.54929023	1.23773289	-0.11532624
C	-1.03889930	1.04177696	-0.46197703
C	-0.62229392	-0.32551386	0.07606500
C	-1.94192916	-1.14106331	-0.15591096
C	-1.51746815	-2.27739420	2.10272707
H	-0.52837184	-2.73551723	2.23802500
H	-2.21811417	-2.84554006	2.72447224
C	-1.92432237	-2.44626101	0.64660803
O	-2.22003138	-3.52565612	0.16981298
O	-0.36437029	1.87090034	-1.02688803
O	-3.17328017	1.55656588	-1.34103305
O	-2.74020045	2.25434694	0.83988314
C	-4.58468426	1.75452792	-1.28711306
H	-4.85465515	2.28261400	-2.20403541
H	-5.12962517	0.80194325	-1.25580693
H	-4.86984836	2.35951404	-0.41985188
C	-2.29282606	3.56466707	0.46796520
H	-1.20471593	3.65102403	0.54738412
H	-2.58768627	3.80669052	-0.55685110
H	-2.77271627	4.25182807	1.16852285
H	-0.31094826	0.94570589	1.86272191
H	0.58833503	-0.55368980	1.88168392

H	-3.99482414	-0.38482510	0.15631420
C	-2.17293937	-1.42404508	-1.65259303
H	-3.03017529	-2.09808037	-1.74506411
H	-2.43291622	-0.49300481	-2.16410105
C	0.58932830	-0.95568723	-0.62554731
C	0.31827895	-1.18610212	-2.11632640
H	0.20594996	-0.20765021	-2.59627217
H	1.19588184	-1.66493292	-2.56462843
C	-0.93679031	-2.04922038	-2.32087299
H	-0.77237494	-3.05001838	-1.90588108
H	-1.12742113	-2.18055423	-3.39176740
H	0.75471894	-1.93919596	-0.14809978
O	1.70571237	-0.11660315	-0.41833102
Si	3.30398430	-0.51216685	-0.13472694
C	4.05909325	1.18135117	0.28303627
C	4.09177947	-1.28415708	-1.66415608
H	3.63541542	-2.25206513	-1.89988497
H	3.96651739	-0.63468514	-2.53634126
H	5.16485832	-1.45297414	-1.51874717
C	3.41604711	-1.72747108	1.30731387
H	2.88550247	-2.66051006	1.08511817
H	4.45863848	-1.98901800	1.51998915
H	2.98437024	-1.30645608	2.22143106
C	3.35399526	1.75352724	1.53130719
H	3.50718201	1.12388410	2.41604393
H	3.74953726	2.75067710	1.76869611
H	2.27640093	1.85135836	1.36472590
C	5.57161518	1.05753904	0.55309024
H	6.11306669	0.67208209	-0.31890814
H	5.99972539	2.04047837	0.79349017
H	5.78478735	0.39457625	1.40069302
C	3.81291417	2.13707809	-0.90513493
H	4.20703745	3.13697702	-0.67595726
H	4.31434031	1.79177300	-1.81723119
H	2.74376234	2.23324441	-1.12026506

16

C	2.23310000	1.11720000	-1.66200000

C	2.75590000	-0.25480000	-1.17020000
C	0.94860000	0.66650000	0.51080000
C	1.22320000	1.71730000	-0.63930000
H	3.06740000	1.80770000	-1.81090000
H	1.75550000	0.96610000	-2.63520000
H	1.66820000	2.59550000	-0.16090000
C	3.41940000	-0.05280000	0.21230000
C	2.31070000	0.44140000	1.19140000
H	3.48290000	-0.68200000	-1.86450000
C	0.63720000	-0.68030000	-0.12430000
C	1.56120000	-1.15770000	-0.96500000
C	-0.06950000	2.17640000	-1.33010000
H	0.15140000	2.54210000	-2.34590000
H	-0.76590000	1.34020000	-1.48290000
C	-0.80510000	3.31950000	-0.66520000
O	-0.37510000	3.99010000	0.24740000
O	2.51500000	0.60240000	2.37490000
O	3.95970000	-1.30060000	0.56880000
O	4.38330000	0.98070000	0.24300000
C	4.54450000	-1.40360000	1.87230000
H	5.11070000	-0.50260000	2.12480000
H	3.78100000	-1.56210000	2.63980000
H	5.21370000	-2.26710000	1.83210000
C	5.51780000	0.77870000	-0.59720000
H	5.27650000	0.94100000	-1.65660000
H	6.26210000	1.51550000	-0.28900000
H	5.92840000	-0.22940000	-0.47710000
H	-1.80070000	3.54510000	-1.11290000
H	-0.27070000	-1.21120000	0.14760000
H	1.48070000	-2.11420000	-1.47070000
C	-0.09280000	1.10910000	1.53460000
H	0.14710000	2.09320000	1.93950000
H	-1.08930000	1.15490000	1.08420000
H	-0.12490000	0.39590000	2.36260000

18

C	2.32860000	1.27800000	-1.56170000
C	2.78720000	-0.14470000	-1.16500000

C	0.97350000	0.76250000	0.57800000
C	1.18400000	1.79960000	-0.62710000
H	3.18790000	1.95200000	-1.51140000
H	1.99370000	1.25750000	-2.60180000
H	1.49490000	2.75250000	-0.19140000
C	3.46220000	-0.00580000	0.20990000
C	2.40850000	0.62030000	1.17570000
H	3.49140000	-0.55870000	-1.89020000
C	0.68710000	-0.58270000	-0.08080000
C	1.58030000	-1.02620000	-0.96960000
C	-0.08620000	2.06940000	-1.46310000
H	0.17470000	2.14610000	-2.53020000
H	-0.80140000	1.24100000	-1.42750000
C	-0.81170000	3.36670000	-1.19150000
O	-0.40710000	4.27190000	-0.49430000
O	2.74910000	0.94280000	2.29350000
O	3.85190000	-1.30010000	0.60740000
O	4.54220000	0.90160000	0.23760000
C	4.41090000	-1.42560000	1.91860000
H	5.09700000	-0.60290000	2.13880000
H	3.63000000	-1.44280000	2.68630000
H	4.94990000	-2.37610000	1.92440000
C	5.63990000	0.56440000	-0.60690000
H	5.43120000	0.79970000	-1.65960000
H	6.48310000	1.17200000	-0.27200000
H	5.89770000	-0.49670000	-0.52190000
H	-1.77600000	3.46850000	-1.74140000
H	-0.20590000	-1.14080000	0.17650000
H	1.47870000	-1.96890000	-1.49670000
C	-0.08490000	1.13390000	1.69570000
C	0.20090000	2.51190000	2.33220000
H	-0.57100000	2.72910000	3.07940000
H	1.16890000	2.52700000	2.83140000
H	0.17140000	3.31320000	1.59110000
C	-0.05500000	0.05140000	2.80680000
H	0.91650000	-0.00780000	3.29520000
H	-0.79770000	0.30080000	3.57200000
H	-0.31000000	-0.94100000	2.41950000

C	-1.53950000	1.16430000	1.16400000
H	-2.22460000	1.18900000	2.01780000
H	-1.75460000	2.05760000	0.57700000
H	-1.80140000	0.28450000	0.56800000

CP16

C	-3.17262346	-0.78410892	1.68966031
C	-3.05013427	-0.70533401	0.15383500
C	-0.82855081	0.17042524	1.30439017
C	-1.81125625	-0.44557783	2.37494432
H	-3.94925246	-0.08841282	2.01298020
H	-3.49988519	-1.78886199	1.97892129
C	-2.82304897	0.76756525	-0.23772917
C	-1.53304131	1.24077407	0.48292398
H	-3.97669506	-1.05599807	-0.31046194
C	-0.56077919	-1.11492196	0.46235380
C	-1.84161991	-1.51742423	-0.34607716
C	-1.06594502	-1.72047218	2.81355711
C	-0.33289006	-2.17644411	1.54593207
O	-1.14325834	2.38860238	0.40355110
O	-2.55134238	0.96633005	-1.61006623
O	-3.96303117	1.48033208	0.18529194
C	-3.58692850	0.55937210	-2.50386407
H	-3.35990352	1.02315627	-3.46597604
H	-3.60940424	-0.53095317	-2.63305211
H	-4.56875550	0.89886683	-2.15746834
C	-3.96128806	2.89717823	-0.02977098
H	-3.53534536	3.14786431	-1.00512033
H	-5.00894313	3.20576530	0.01166783
H	-3.38936017	3.41808021	0.74380625
H	-0.32063008	-1.52419505	3.59186149
H	-1.71790515	-2.51391844	3.19030750
H	-1.96053503	0.24831219	3.20623838
O	0.29487079	-3.20189701	1.40979011
H	-2.03286933	-2.59080101	-0.24452381
H	0.31211192	-1.04073184	-0.18929517
H	-1.67519208	-1.31925687	-1.40702833
C	0.45091193	0.74710408	1.91560841

H	1.08444717	1.16695014	1.13027709
H	0.21284197	1.55660927	2.61096108
H	1.02925927	-0.01191284	2.44896510

RP16

C	-2.36180802	2.23584002	-0.55599807
C	-2.59068724	1.10949313	0.49672810
C	-0.50994483	0.82524081	-1.59095605
C	-0.89760421	2.22368218	-1.04595111
H	-3.04855097	2.10665939	-1.39632837
H	-2.58508801	3.21208050	-0.11290420
H	-0.76427430	2.97193246	-1.83539096
C	-3.36870215	-0.08997389	-0.09150110
C	-2.26922105	-0.87679114	-0.85835513
C	-0.91925497	-0.25850891	-0.51765600
C	-1.22052827	0.44309493	0.83981382
C	0.01448623	2.54384235	0.14878295
H	1.07346727	2.57807694	-0.13397392
H	-0.22302122	3.51653628	0.59741307
C	-0.16738406	1.46640995	1.21280705
O	0.46213684	1.43317709	2.25145393
O	-2.46546538	-1.84519291	-1.55728398
O	-3.80548611	-0.84709794	1.02249004
O	-4.40507939	0.20065078	-0.98626204
C	-4.42659944	-2.10273237	0.71991495
H	-3.68827123	-2.85783020	0.43083389
H	-4.93266646	-2.41345096	1.63653938
H	-5.15325452	-1.99841301	-0.09146300
C	-5.48397655	0.94712620	-0.42122086
H	-6.25928366	0.99279420	-1.18791931
H	-5.87959369	0.45807911	0.47496727
H	-5.17625257	1.96864203	-0.16164195
H	-3.09652407	1.47943724	1.39054605
H	-1.33100831	-0.27773497	1.65138016
H	-0.16059101	-1.04186110	-0.46227496
C	-1.06343725	0.54510813	-2.99317204
H	-0.82176623	-0.47131701	-3.31695549
H	-2.15297828	0.64246299	-3.03027213

H	-0.63484601	1.25024105	-3.71257528
H	0.58489697	0.79478983	-1.65699420

CP18

C	-3.26300640	-0.64967990	1.70395339
C	-3.10665305	-0.67455600	0.16744809
C	-0.84141511	0.16141017	1.36745434
C	-1.88723922	-0.43546207	2.41819345
H	-3.95953754	0.15160399	1.96100018
H	-3.71360348	-1.58492925	2.05114923
C	-2.81640409	0.76946182	-0.26094101
C	-1.60965506	1.25247418	0.59036919
H	-4.03637038	-1.01091905	-0.30126114
C	-0.63055015	-1.14640800	0.53232321
C	-1.91790716	-1.53980790	-0.27283797
C	-1.28951711	-1.79505496	2.82512334
C	-0.48748020	-2.23667930	1.59948798
O	-1.38228929	2.44432792	0.65569083
O	-2.37071937	0.92173628	-1.59459309
O	-3.99181841	1.50020427	-0.01105187
C	-3.31035401	0.53229728	-2.59650713
H	-2.90362943	0.88299204	-3.54694122
H	-3.43256326	-0.55787400	-2.64303927
H	-4.28968012	0.99070395	-2.42464835
C	-3.98800252	2.90593823	-0.29078713
H	-3.36287432	3.13603403	-1.15732229
H	-5.02816919	3.17156140	-0.50036408
H	-3.62272522	3.47909911	0.56534598
H	-0.62509698	-1.73756038	3.68951321
H	-2.04769211	-2.54685813	3.06582543
H	-2.01447830	0.23154521	3.27170026
O	0.10365789	-3.28455133	1.46662798
H	-2.14247517	-2.60135704	-0.12497420
H	0.24166202	-1.12638235	-0.12089319
H	-1.72888928	-1.40294784	-1.33859830
C	0.52217518	0.74601292	1.95031133
C	1.33416327	1.40264992	0.80198188
H	0.83782993	2.27697932	0.38617715

H	2.30990896	1.71956729	1.18457491
H	1.52220587	0.69205589	-0.01045813
C	0.25239903	1.79051091	3.05644048
H	1.20205417	2.22766106	3.38419805
H	-0.38912520	2.59858309	2.70798837
H	-0.20755496	1.32816293	3.93674641
C	1.46952205	-0.31546796	2.56485122
H	1.07470502	-0.77063395	3.47452543
H	1.73893835	-1.11202207	1.86718393
H	2.40105498	0.18479928	2.84925118

RP18

C	-2.78406025	2.12508217	-0.33103424
C	-2.67985942	0.94640229	0.68020126
C	-0.89651093	1.06128614	-1.73826101
C	-1.43192400	2.34952211	-1.04250829
H	-3.57624335	1.92436788	-1.05321407
H	-3.06299434	3.04428210	0.19497007
H	-1.53528131	3.15944642	-1.76907711
C	-3.42560617	-0.30444096	0.17490525
C	-2.42449118	-0.90418188	-0.84980416
C	-1.10058284	-0.14869193	-0.74272091
C	-1.20810584	0.42879285	0.70667917
C	-0.40221704	2.73684705	0.03374775
H	0.57218613	3.00286604	-0.39116676
H	-0.73508382	3.59796026	0.62784711
C	-0.21784428	1.54772598	0.97153133
O	0.62810694	1.49887815	1.84193106
O	-2.64930420	-1.89045291	-1.51787985
O	-3.54244215	-1.16335120	1.29375529
O	-4.64755740	-0.10401984	-0.47805926
C	-4.08183305	-2.46609616	1.03653917
H	-3.34383823	-3.12084717	0.56213229
H	-4.36103544	-2.87007113	2.01217797
H	-4.96257027	-2.40832217	0.38992796
C	-5.66116361	0.49522524	0.33015421
H	-6.57986135	0.46512087	-0.25816124
H	-5.80279420	-0.05810790	1.26437326

H	-5.42148653	1.54006508	0.56734098
H	-3.03306725	1.22028327	1.67605728
H	-1.08273899	-0.35092178	1.45945604
H	-0.28530909	-0.86330506	-0.87024204
C	-1.29325998	0.85217910	-3.23475839
H	0.19576383	1.17534818	-1.77155419
C	-0.61918818	1.96462240	-4.06983819
H	0.47086615	1.94116503	-3.95766426
H	-0.84721013	1.82694635	-5.13202023
H	-0.96543891	2.96496901	-3.79087923
C	-0.73527380	-0.50016722	-3.72954229
H	0.34409380	-0.56645514	-3.54438053
H	-1.22013986	-1.34925329	-3.24552027
H	-0.88948028	-0.59577579	-4.80998941
C	-2.81269297	0.92062183	-3.48870103
H	-3.38128228	0.20047721	-2.89792702
H	-3.20518744	1.92033926	-3.27470228
H	-3.01871702	0.71156930	-4.54417416

Methane

C	0.00000000	0.00000000	0.00000000
H	0.63060000	0.63060000	-0.63060000
H	-0.63060000	0.63060000	0.63060000
H	0.63060000	-0.63060000	0.63060000
H	-0.63060000	-0.63060000	-0.63060000

Ethane

C	0.00000000	0.00000000	0.76570000
H	0.51010000	0.88360000	1.16450000
H	0.51010000	-0.88360000	1.16450000
H	-1.02030000	0.00000000	1.16450000
C	0.00000000	0.00000000	-0.76570000
H	1.02030000	0.00000000	-1.16450000
H	-0.51010000	-0.88360000	-1.16450000
H	-0.51010000	0.88360000	-1.16450000

4. X-Ray Crystal Data

The sample of compound **5** for X-ray analysis was obtained via slow evaporation in ether. The thermal ellipsoids are shown at the 30% possibility level.

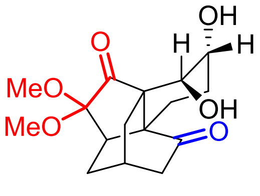

5

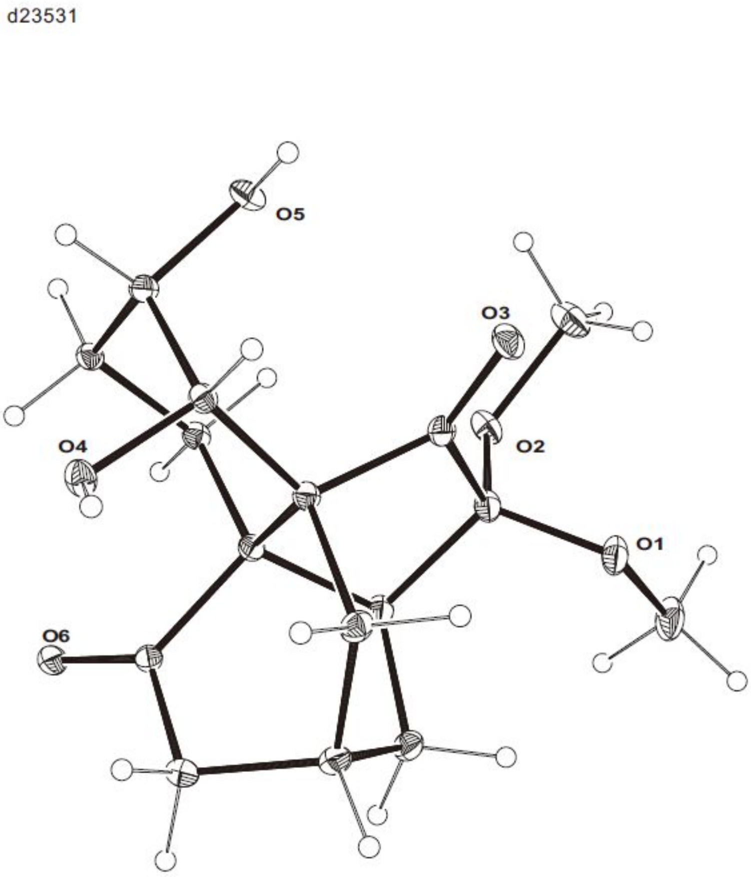

Table S2. Crystal data and structure refinement for compound **22** (CCDC 2344716).

Identification code	shelx	
Empirical formula	C16 H22 O6	
Formula weight	310.33	
Temperature	200(2) K	
Wavelength	0.71073 Å	
Crystal system	Monoclinic	
Space group	P 21/n	
Unit cell dimensions	a = 9.4319(3) Å	α= 90°.
	b = 10.9493(4) Å	β= 107.4110(10)°.
	c = 15.0006(6) Å	γ = 90°.
Volume	1478.17(9) $Å^3$	
Z	4	
Density (calculated)	1.394 Mg/m^3	
Absorption coefficient	0.106 mm^{-1}	
F(000)	664	
Crystal size	0.220 x 0.150 x 0.080 mm^3	
Theta range for data collection	2.284 to 25.058°.	
Index ranges	-11<=h<=11, -13<=k<=12, -17<=l<=17	
Reflections collected	28744	
Independent reflections	2608 [R(int) = 0.0494]	
Completeness to theta = 25.058°	99.7 %	
Refinement method	Full-matrix least-squares on F^2	
Data / restraints / parameters	2608 / 0 / 205	
Goodness-of-fit on F^2	1.034	
Final R indices [I>2sigma(I)]	R1 = 0.0360, wR2 = 0.0920	
R indices (all data)	R1 = 0.0446, wR2 = 0.0998	
Extinction coefficient	n/a	
Largest diff. peak and hole	0.285 and -0.186 e.$Å^{-3}$	

5. ^{1}H- and ^{13}C-NMR Spectra

	Parameter	Value
1	Sample Name	060901-11BP-001-036.1.fid
2	Origin	Bruker BioSpin GmbH
3	Owner	nmrsu
4	Site	
5	Spectrometer	Avance
6	Author	
7	Solvent	CDCl3
8	Temperature	294.7
9	Pulse Sequence	zg30
10	Experiment	1D
11	Number of Scans	32
12	Receiver Gain	101
13	Relaxation Delay	1.0000
14	Pulse Width	10.5200
15	Acquisition Time	2.7525
16	Acquisition Date	2022-07-27T10:21:22
17	Modification Date	2022-07-27T10:30:01
18	Spectrometer Frequency	600.14
19	Spectral Width	11904.8
20	Lowest Frequency	-2246.5
21	Nucleus	1H
22	Acquired Size	32768
23	Spectral Size	131072

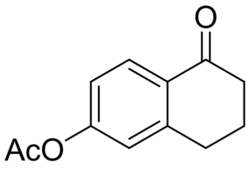

S2

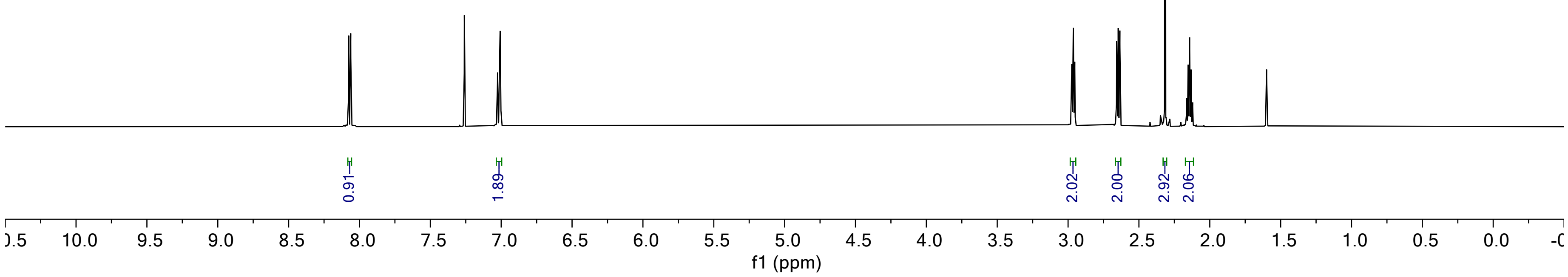

	Parameter	Value
1	Sample Name	060901-11BP-001-036.2.fid
2	Origin	Bruker BioSpin GmbH
3	Owner	nmrsu
4	Site	
5	Spectrometer	Avance
6	Author	
7	Solvent	CDCl3
8	Temperature	294.7
9	Pulse Sequence	zgpg30
10	Experiment	1D
11	Number of Scans	6000
12	Receiver Gain	101
13	Relaxation Delay	2.0000
14	Pulse Width	8.0000
15	Acquisition Time	1.3763
16	Acquisition Date	2022-07-29T00:03:00
17	Modification Date	2022-07-29T10:02:55
18	Spectrometer Frequency	100.63
19	Spectral Width	23809.5
20	Lowest Frequency	-848.3
21	Nucleus	13C
22	Acquired Size	32768
23	Spectral Size	65536

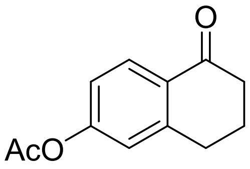

S2

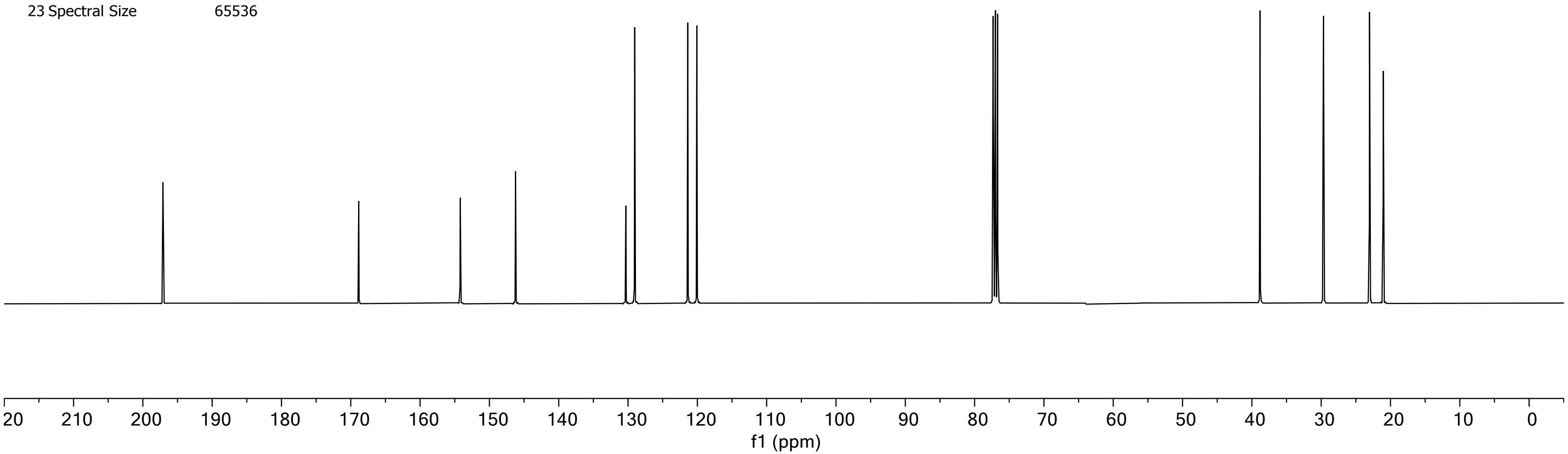

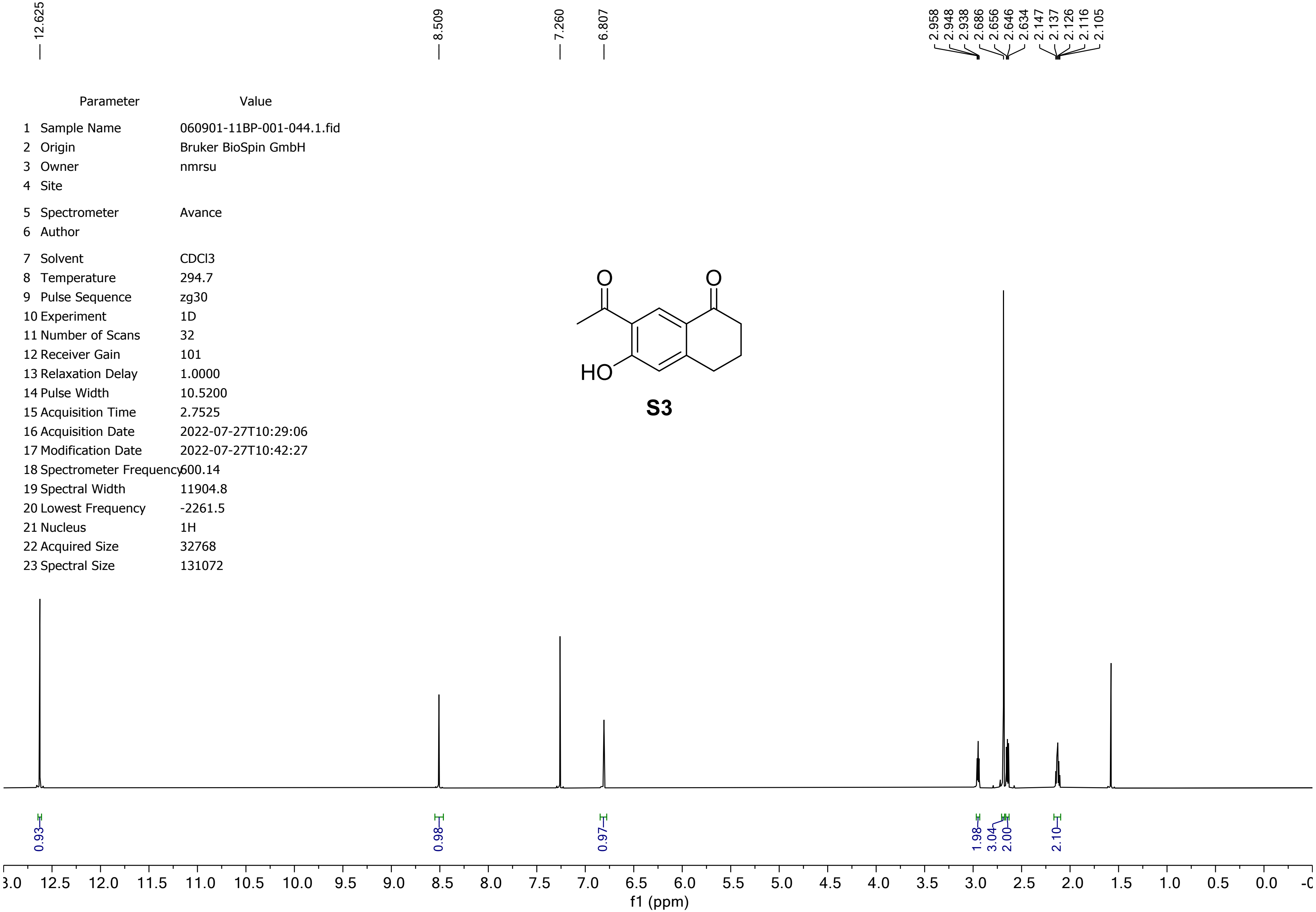

Parameter	Value
1 Sample Name	060901-11BP-001-044.1.fid
2 Origin	Bruker BioSpin GmbH
3 Owner	nmrsu
4 Site	
5 Spectrometer	Avance
6 Author	
7 Solvent	CDCl3
8 Temperature	294.7
9 Pulse Sequence	zg30
10 Experiment	1D
11 Number of Scans	32
12 Receiver Gain	101
13 Relaxation Delay	1.0000
14 Pulse Width	10.5200
15 Acquisition Time	2.7525
16 Acquisition Date	2022-07-27T10:29:06
17 Modification Date	2022-07-27T10:42:27
18 Spectrometer Frequency	600.14
19 Spectral Width	11904.8
20 Lowest Frequency	-2261.5
21 Nucleus	1H
22 Acquired Size	32768
23 Spectral Size	131072

S3

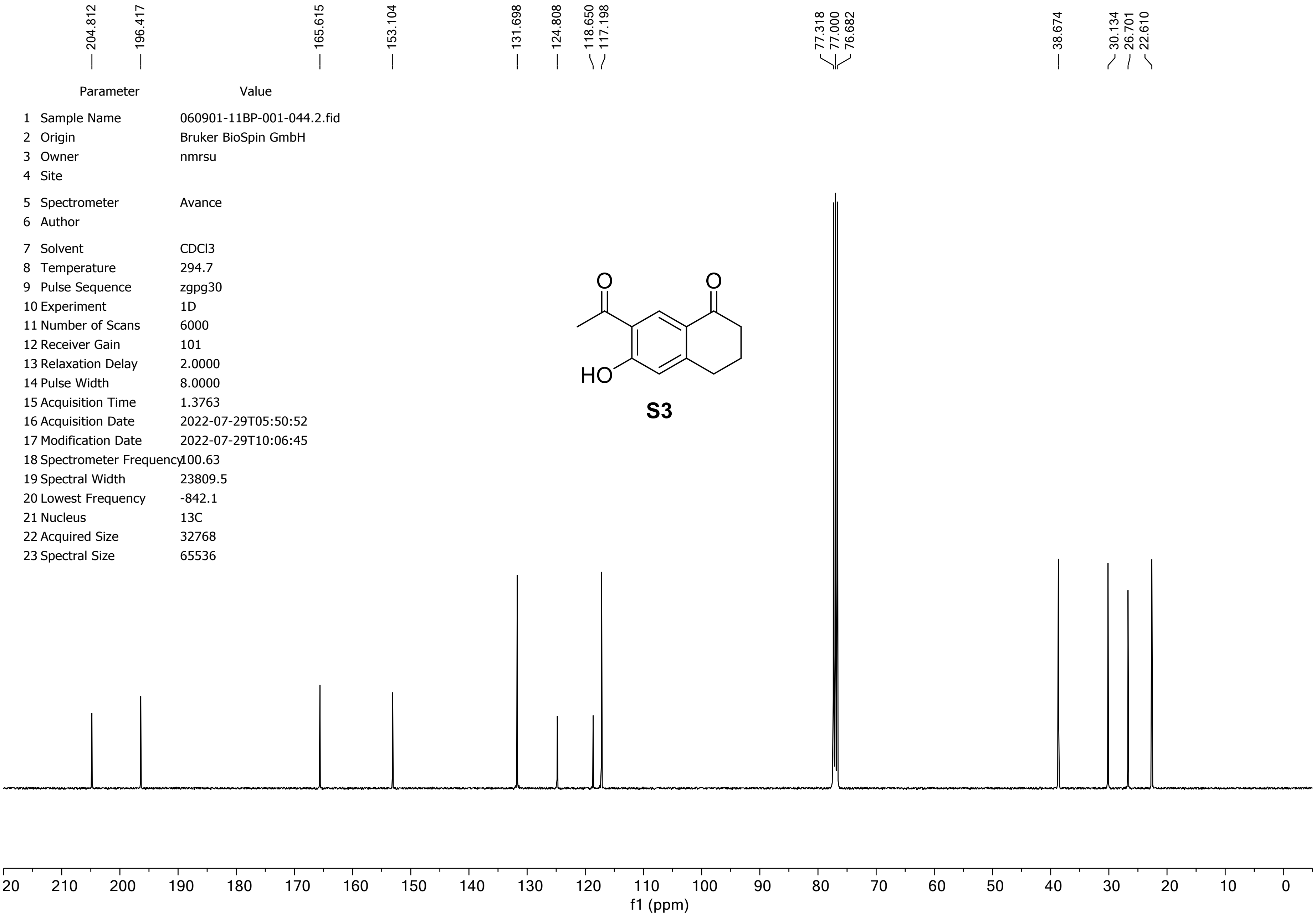

	Parameter	Value
1	Sample Name	060901-11BP-001-044.2.fid
2	Origin	Bruker BioSpin GmbH
3	Owner	nmrsu
4	Site	
5	Spectrometer	Avance
6	Author	
7	Solvent	CDCl3
8	Temperature	294.7
9	Pulse Sequence	zgpg30
10	Experiment	1D
11	Number of Scans	6000
12	Receiver Gain	101
13	Relaxation Delay	2.0000
14	Pulse Width	8.0000
15	Acquisition Time	1.3763
16	Acquisition Date	2022-07-29T05:50:52
17	Modification Date	2022-07-29T10:06:45
18	Spectrometer Frequency	100.63
19	Spectral Width	23809.5
20	Lowest Frequency	-842.1
21	Nucleus	13C
22	Acquired Size	32768
23	Spectral Size	65536

	Parameter	Value
1	Sample Name	060901-11BP-001-046-MeOD.1.fid
2	Origin	Bruker BioSpin GmbH
3	Owner	nmrsu
4	Site	
5	Spectrometer	Avance
6	Author	
7	Solvent	MeOD
8	Temperature	294.7
9	Pulse Sequence	zg30
10	Experiment	1D
11	Number of Scans	32
12	Receiver Gain	101
13	Relaxation Delay	1.0000
14	Pulse Width	7.9700
15	Acquisition Time	4.1943
16	Acquisition Date	2022-08-28T23:18:01
17	Modification Date	2022-08-28T23:25:56
18	Spectrometer Frequency	400.17
19	Spectral Width	7812.5
20	Lowest Frequency	-1435.2
21	Nucleus	1H
22	Acquired Size	32768
23	Spectral Size	65536

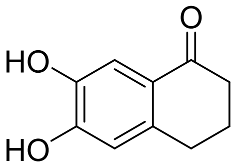

S4

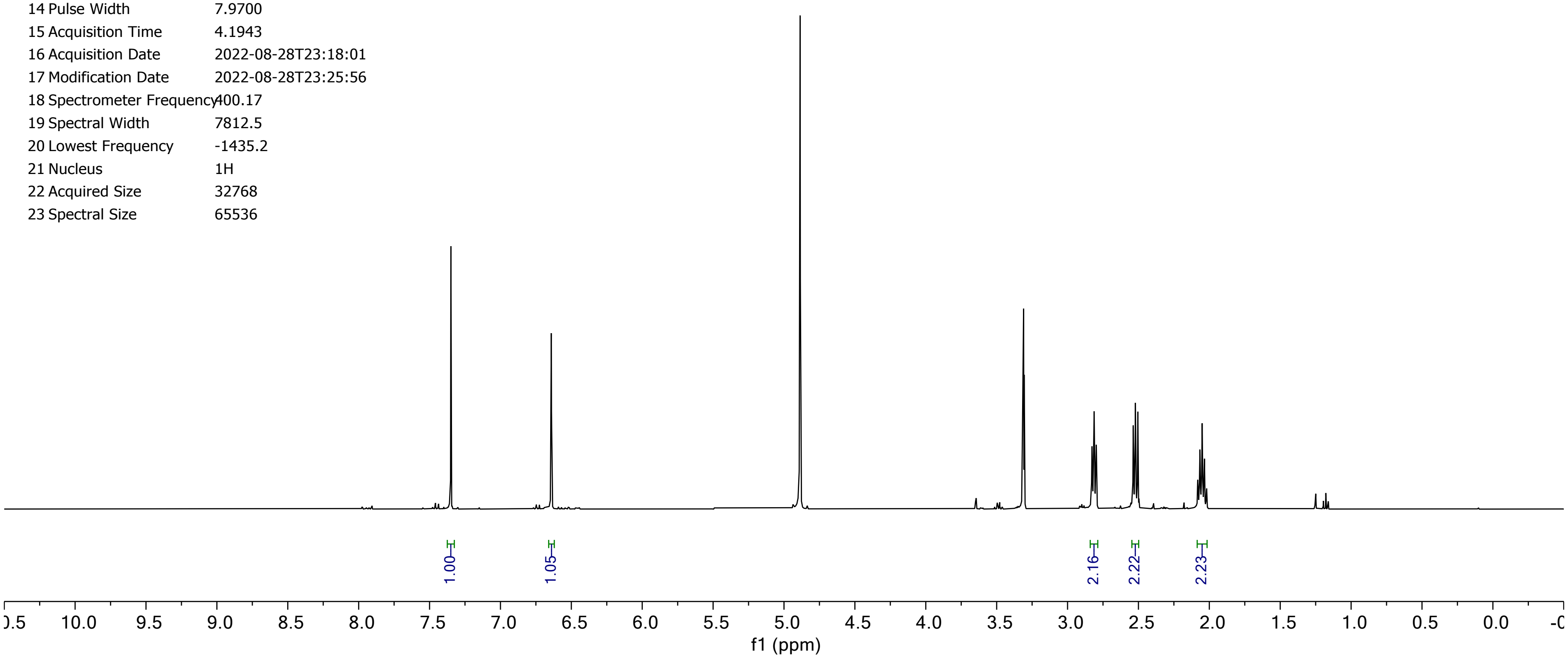

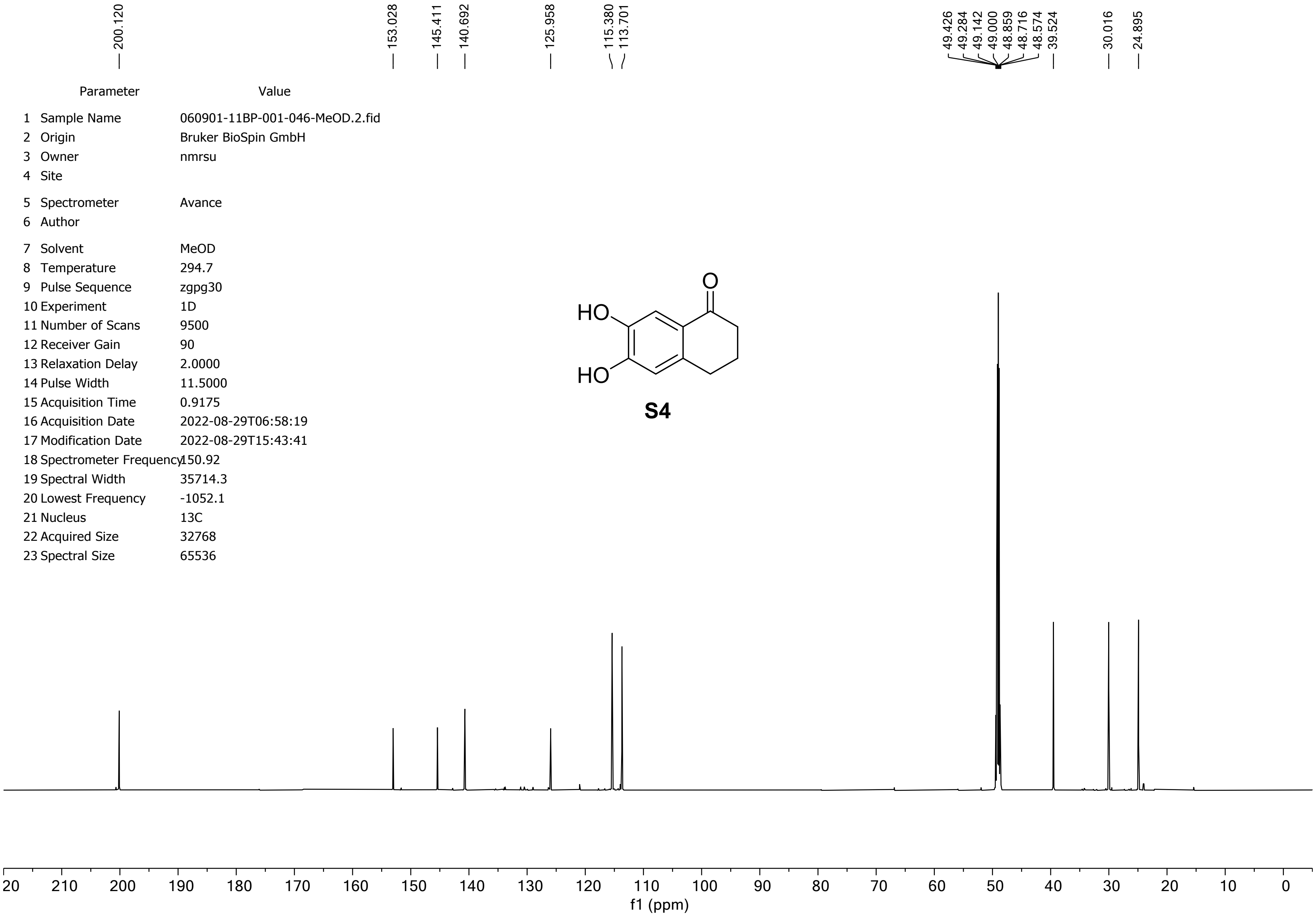

	Parameter	Value
1	Sample Name	060901-11BP-001-046-MeOD.2.fid
2	Origin	Bruker BioSpin GmbH
3	Owner	nmrsu
4	Site	
5	Spectrometer	Avance
6	Author	
7	Solvent	MeOD
8	Temperature	294.7
9	Pulse Sequence	zgpg30
10	Experiment	1D
11	Number of Scans	9500
12	Receiver Gain	90
13	Relaxation Delay	2.0000
14	Pulse Width	11.5000
15	Acquisition Time	0.9175
16	Acquisition Date	2022-08-29T06:58:19
17	Modification Date	2022-08-29T15:43:41
18	Spectrometer Frequency	150.92
19	Spectral Width	35714.3
20	Lowest Frequency	-1052.1
21	Nucleus	13C
22	Acquired Size	32768
23	Spectral Size	65536

S4

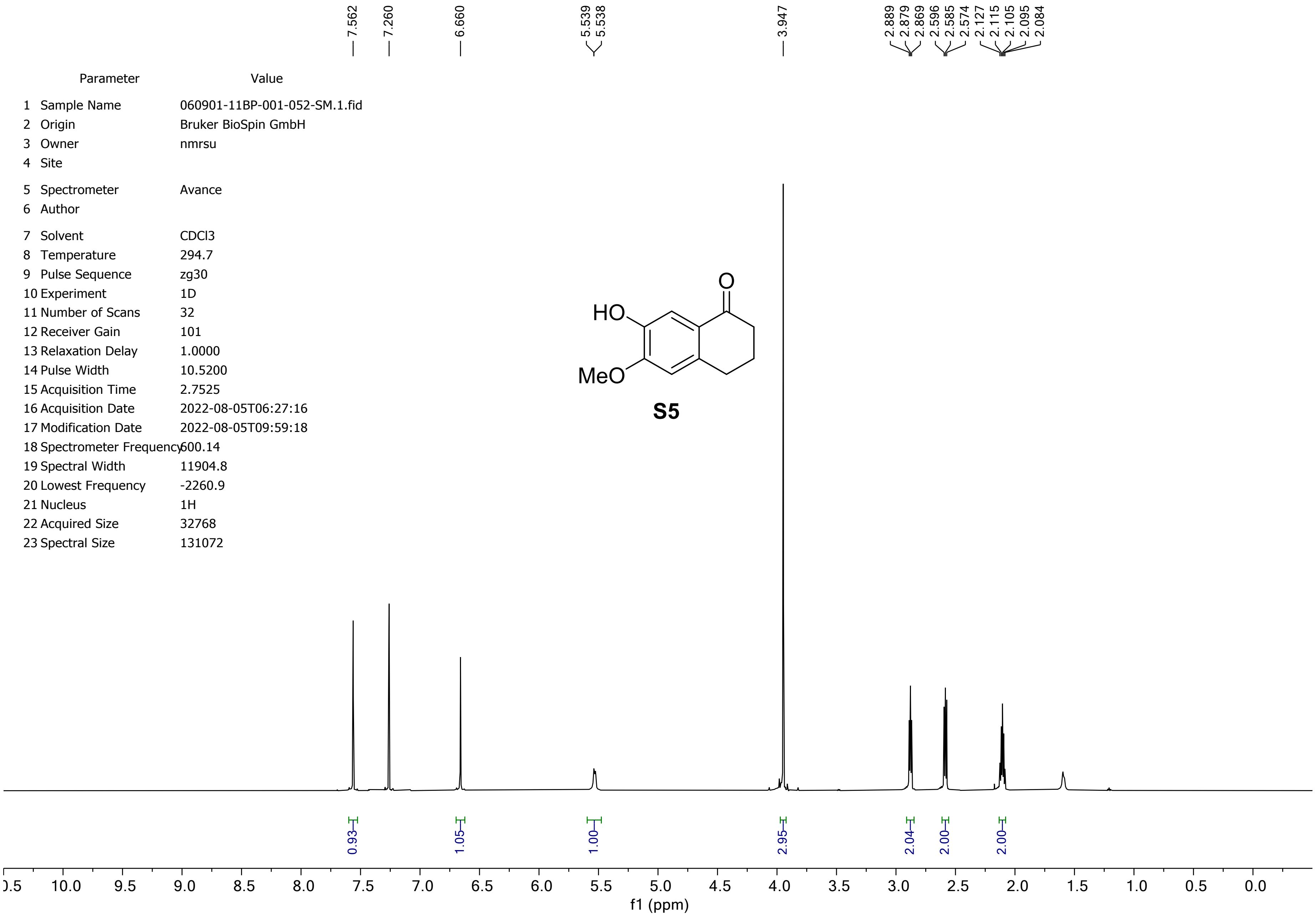

	Parameter	Value
1	Sample Name	060901-11BP-001-052-SM.1.fid
2	Origin	Bruker BioSpin GmbH
3	Owner	nmrsu
4	Site	
5	Spectrometer	Avance
6	Author	
7	Solvent	CDCl3
8	Temperature	294.7
9	Pulse Sequence	zg30
10	Experiment	1D
11	Number of Scans	32
12	Receiver Gain	101
13	Relaxation Delay	1.0000
14	Pulse Width	10.5200
15	Acquisition Time	2.7525
16	Acquisition Date	2022-08-05T06:27:16
17	Modification Date	2022-08-05T09:59:18
18	Spectrometer Frequency	600.14
19	Spectral Width	11904.8
20	Lowest Frequency	-2260.9
21	Nucleus	1H
22	Acquired Size	32768
23	Spectral Size	131072

S5

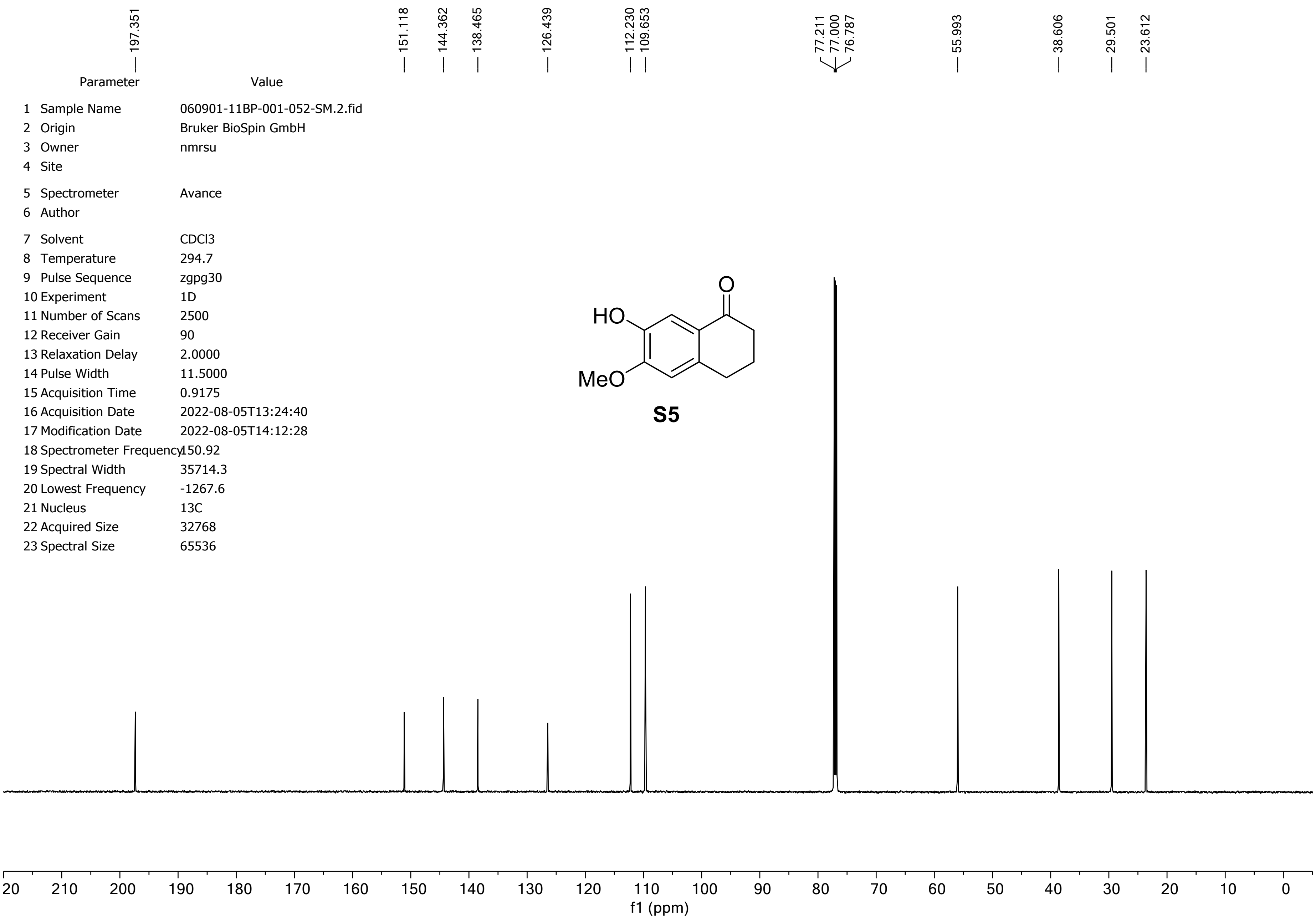
197.351
151.118
144.362
138.465
126.439
112.230
109.653
77.211
77.000
76.787
55.993
38.606
29.501
23.612
Parameter
Value
1 Sample Name 060901-11BP-001-052-SM.2.fid
2 Origin Bruker BioSpin GmbH
3 Owner nmrsu
4 Site
5 Spectrometer Avance
6 Author
7 Solvent CDCl3
8 Temperature 294.7
9 Pulse Sequence zgpg30
10 Experiment 1D
11 Number of Scans 2500
12 Receiver Gain 90
13 Relaxation Delay 2.0000
14 Pulse Width 11.5000
15 Acquisition Time 0.9175
16 Acquisition Date 2022-08-05T13:24:40
17 Modification Date 2022-08-05T14:12:28
18 Spectrometer Frequency 150.92
19 Spectral Width 35714.3
20 Lowest Frequency -1267.6
21 Nucleus 13C
22 Acquired Size 32768
23 Spectral Size 65536
HO
MeO
O
S5
20 210 200 190 180 170 160 150 140 130 120 110 100 90 80 70 60 50 40 30 20 10 0
f1 (ppm)

	Parameter	Value
1	Sample Name	060901-11BP-001-052.1.fid
2	Origin	Bruker BioSpin GmbH
3	Owner	nmrsu
4	Site	
5	Spectrometer	Avance
6	Author	
7	Solvent	CDCl3
8	Temperature	294.7
9	Pulse Sequence	zg30
10	Experiment	1D
11	Number of Scans	32
12	Receiver Gain	101
13	Relaxation Delay	1.0000
14	Pulse Width	10.5200
15	Acquisition Time	2.7525
16	Acquisition Date	2022-08-08T09:25:16
17	Modification Date	2022-08-08T09:57:12
18	Spectrometer Frequency	600.14
19	Spectral Width	11904.8
20	Lowest Frequency	-2261.3
21	Nucleus	1H
22	Acquired Size	32768
23	Spectral Size	131072

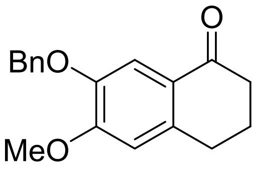

S6

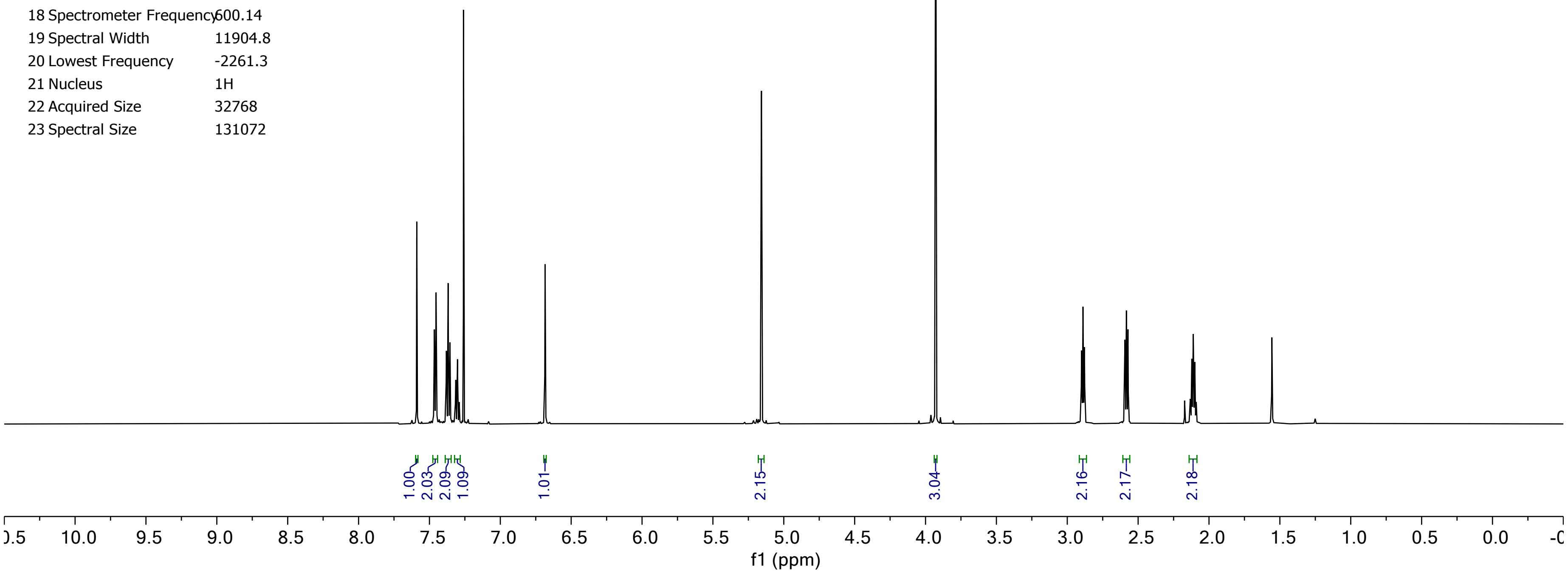

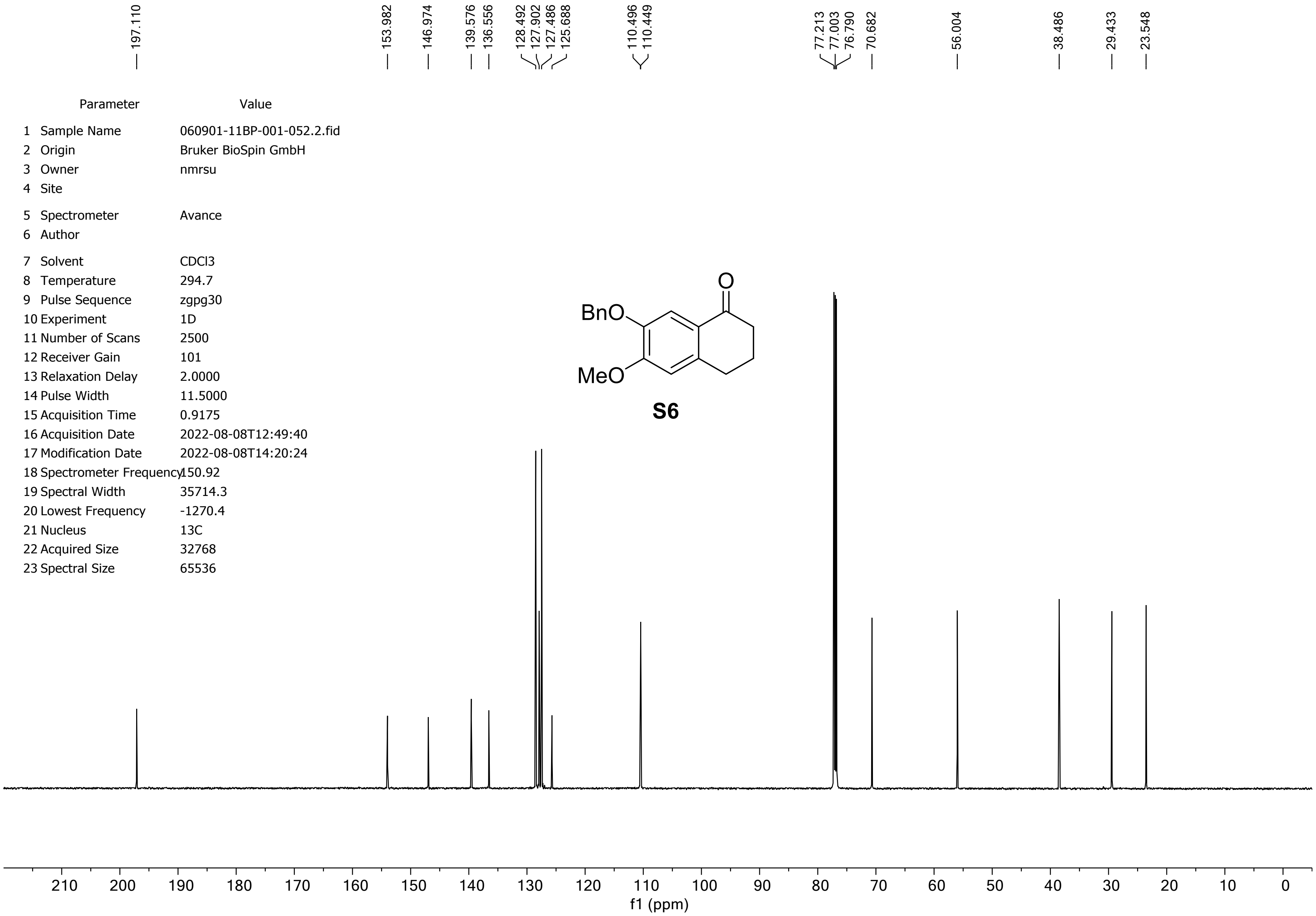
197.110
153.982
146.974
139.576
136.556
128.492
127.902
127.486
125.688
110.496
110.449
77.213
77.003
76.790
70.682
56.004
38.486
29.433
23.548
Parameter Value
1 Sample Name 060901-11BP-001-052.2.fid
2 Origin Bruker BioSpin GmbH
3 Owner nmrsu
4 Site
5 Spectrometer Avance
6 Author
7 Solvent CDCl3
8 Temperature 294.7
9 Pulse Sequence zgpg30
10 Experiment 1D
11 Number of Scans 2500
12 Receiver Gain 101
13 Relaxation Delay 2.0000
14 Pulse Width 11.5000
15 Acquisition Time 0.9175
16 Acquisition Date 2022-08-08T12:49:40
17 Modification Date 2022-08-08T14:20:24
18 Spectrometer Frequency 150.92
19 Spectral Width 35714.3
20 Lowest Frequency -1270.4
21 Nucleus 13C
22 Acquired Size 32768
23 Spectral Size 65536
BnO
MeO
O
S6
210 200 190 180 170 160 150 140 130 120 110 100 90 80 70 60 50 40 30 20 10 0
f1 (ppm)

	Parameter	Value
1	Sample Name	060901-11BP-001-056.3.fid
2	Origin	Bruker BioSpin GmbH
3	Owner	nmrsu
4	Site	
5	Spectrometer	Avance
6	Author	
7	Solvent	CDCl3
8	Temperature	294.7
9	Pulse Sequence	zg30
10	Experiment	1D
11	Number of Scans	32
12	Receiver Gain	101
13	Relaxation Delay	1.0000
14	Pulse Width	7.9700
15	Acquisition Time	4.1943
16	Acquisition Date	2022-08-11T00:16:43
17	Modification Date	2022-08-11T00:17:52
18	Spectrometer Frequency	400.17
19	Spectral Width	7812.5
20	Lowest Frequency	-1444.8
21	Nucleus	1H
22	Acquired Size	32768
23	Spectral Size	65536

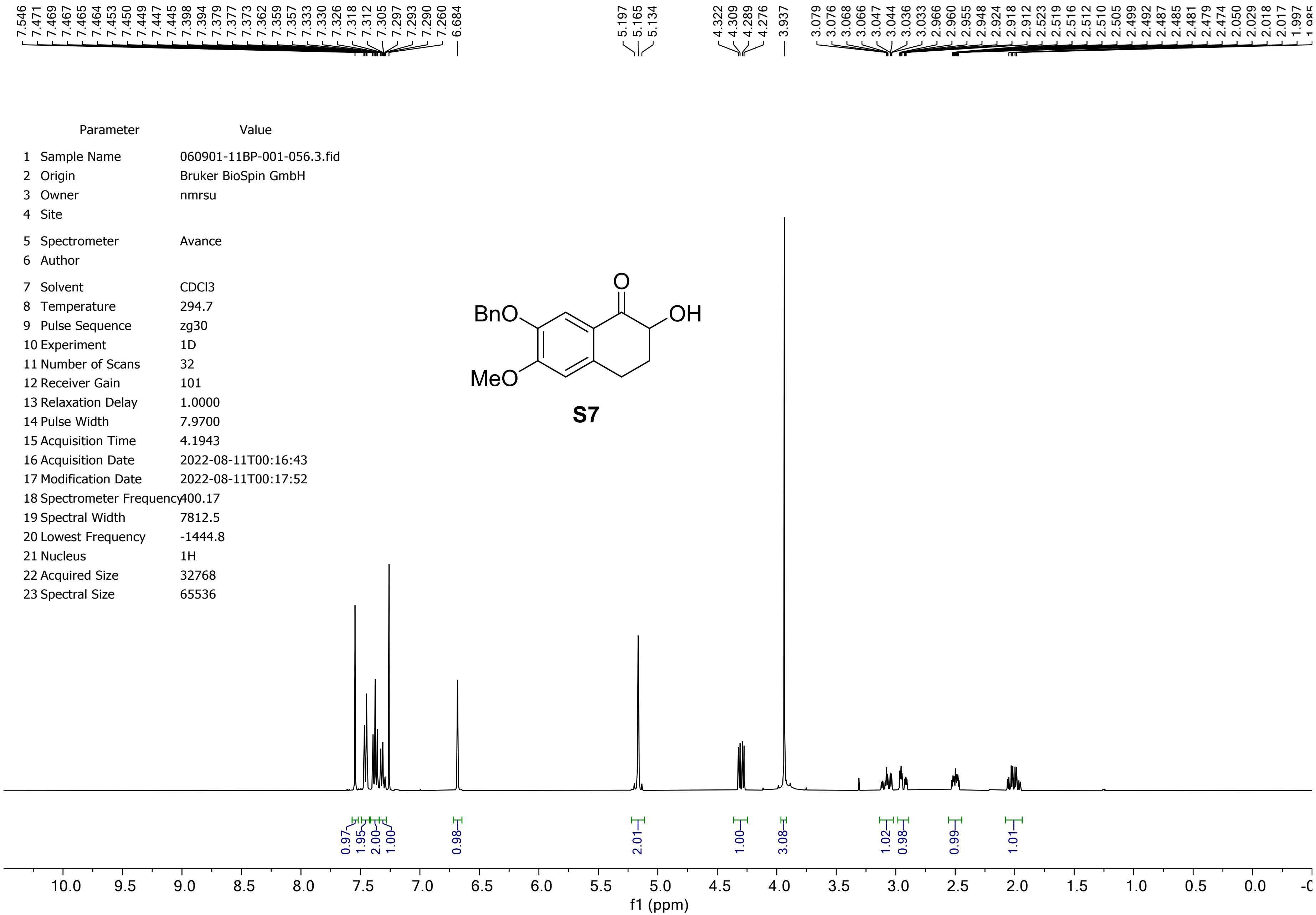

	Parameter	Value
1	Sample Name	060901-11BP-001-056.4.fid
2	Origin	Bruker BioSpin GmbH
3	Owner	nmrsu
4	Site	
5	Spectrometer	Avance
6	Author	
7	Solvent	CDCl3
8	Temperature	294.7
9	Pulse Sequence	zgpg30
10	Experiment	1D
11	Number of Scans	7500
12	Receiver Gain	101
13	Relaxation Delay	2.0000
14	Pulse Width	8.0000
15	Acquisition Time	1.3763
16	Acquisition Date	2022-08-11T07:40:00
17	Modification Date	2022-08-11T09:41:11
18	Spectrometer Frequency	100.63
19	Spectral Width	23809.5
20	Lowest Frequency	-836.3
21	Nucleus	13C
22	Acquired Size	32768
23	Spectral Size	65536

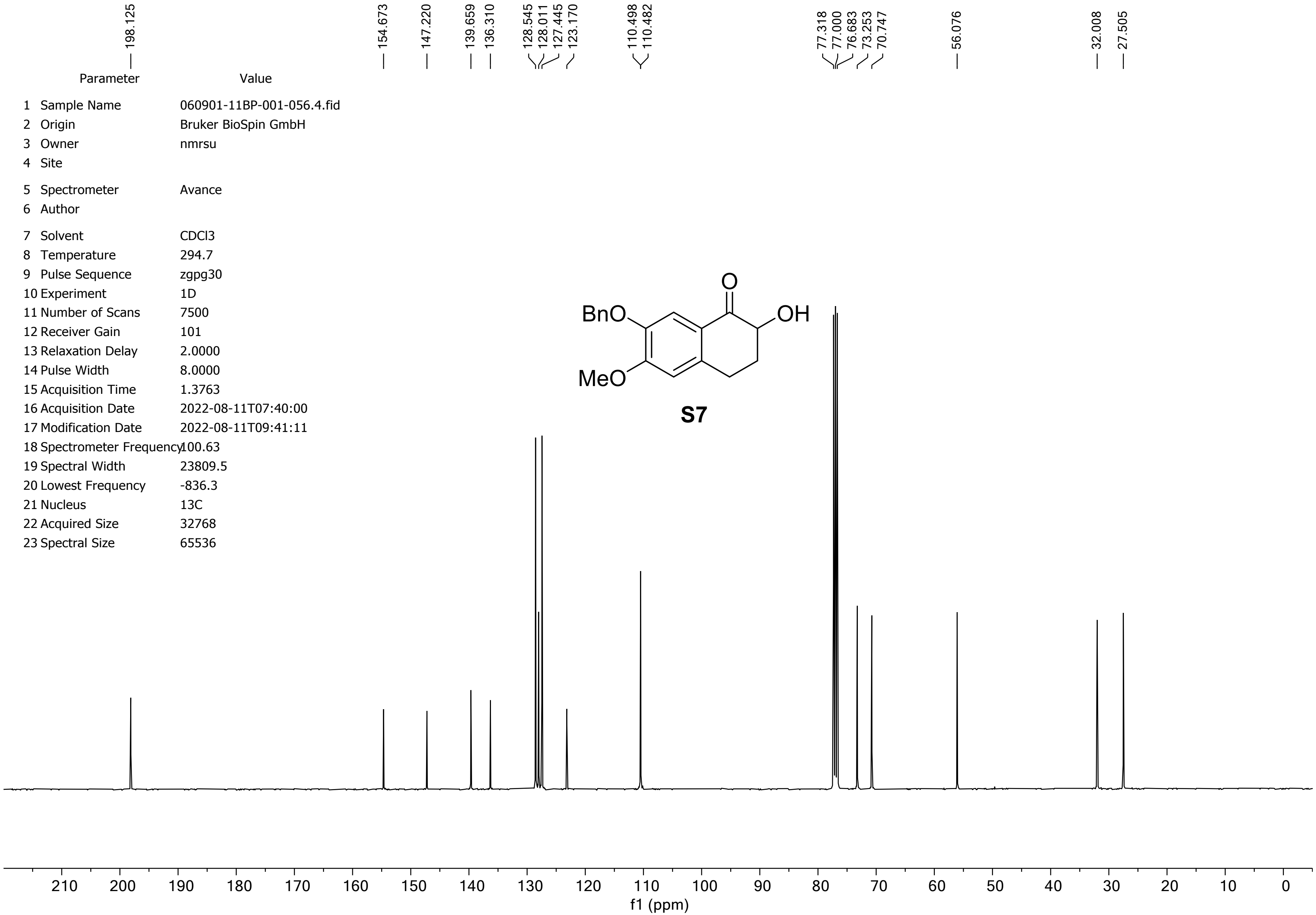

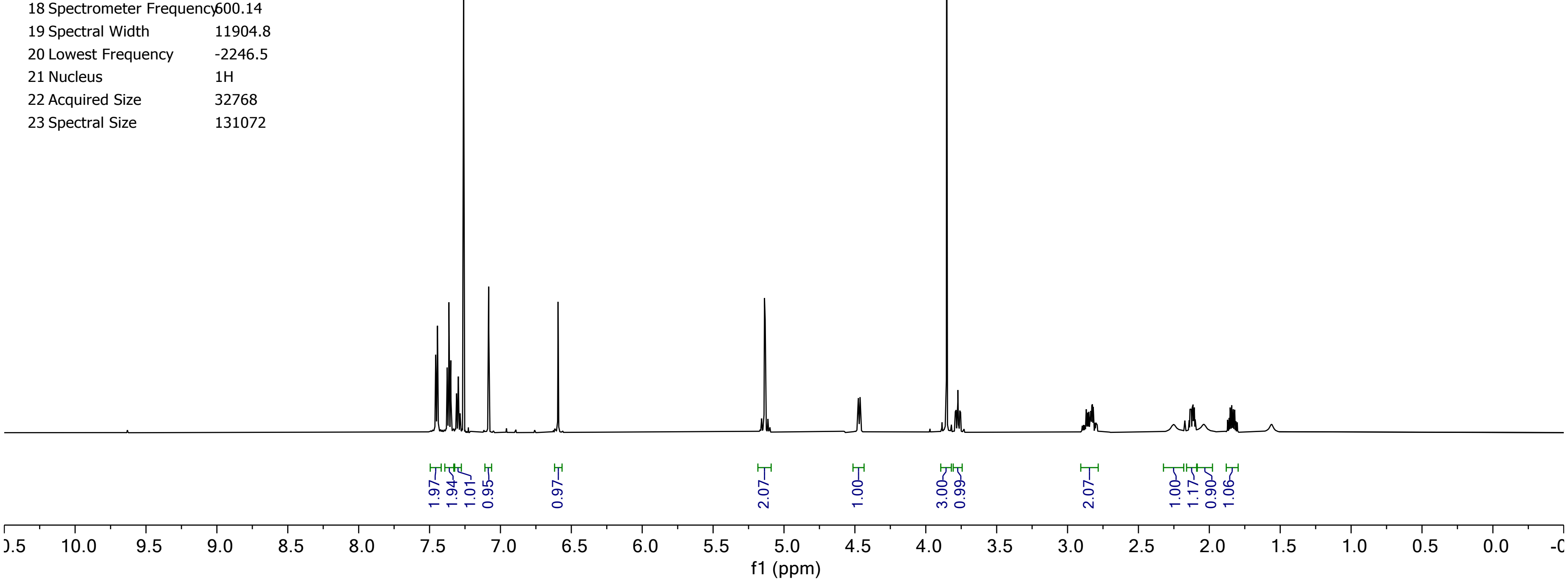

	Parameter	Value
1	Sample Name	060901-11BP-001-058.2.fid
2	Origin	Bruker BioSpin GmbH
3	Owner	nmrsu
4	Site	
5	Spectrometer	Avance
6	Author	
7	Solvent	CDCl3
8	Temperature	294.7
9	Pulse Sequence	zg30
10	Experiment	1D
11	Number of Scans	32
12	Receiver Gain	101
13	Relaxation Delay	1.0000
14	Pulse Width	10.5200
15	Acquisition Time	2.7525
16	Acquisition Date	2022-08-09T11:29:27
17	Modification Date	2022-08-09T11:32:12
18	Spectrometer Frequency	600.14
19	Spectral Width	11904.8
20	Lowest Frequency	-2246.5
21	Nucleus	1H
22	Acquired Size	32768
23	Spectral Size	131072

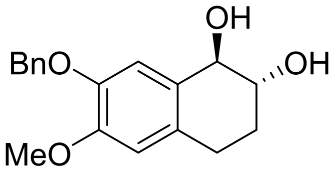

S8

Parameter	Value
1 Sample Name	060901-11BP-001-058.3.fid
2 Origin	Bruker BioSpin GmbH
3 Owner	nmrsu
4 Site	
5 Spectrometer	Avance
6 Author	
7 Solvent	CDCl3
8 Temperature	294.7
9 Pulse Sequence	zgpg30
10 Experiment	1D
11 Number of Scans	3000
12 Receiver Gain	90
13 Relaxation Delay	2.0000
14 Pulse Width	11.5000
15 Acquisition Time	0.9175
16 Acquisition Date	2022-08-09T14:03:01
17 Modification Date	2022-08-09T14:57:12
18 Spectrometer Frequency	150.92
19 Spectral Width	35714.3
20 Lowest Frequency	-1266.5
21 Nucleus	13C
22 Acquired Size	32768
23 Spectral Size	65536

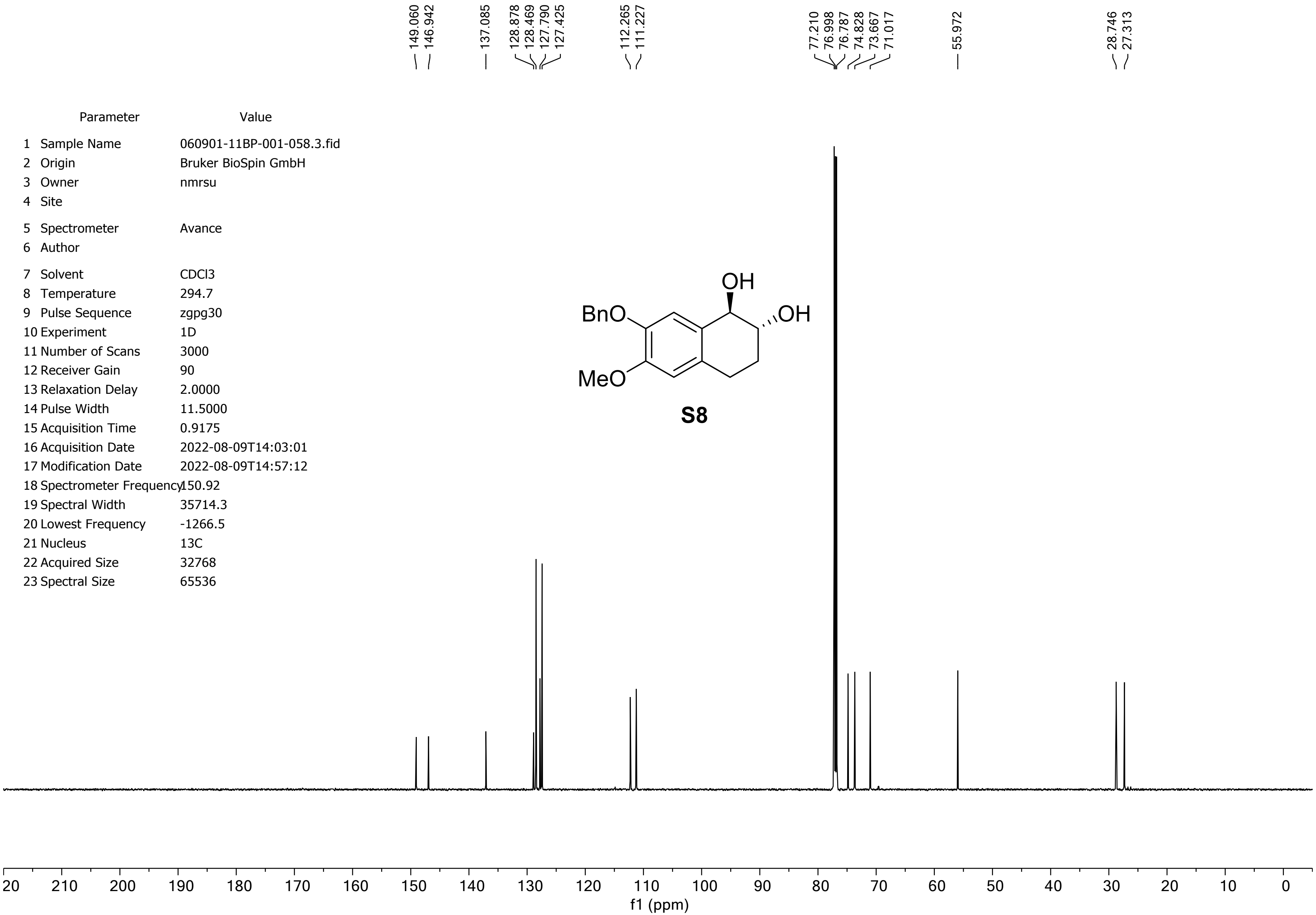

	Parameter	Value
1	Sample Name	060901-11BP-001-060.1.fid
2	Origin	Bruker BioSpin GmbH
3	Owner	nmrsu
4	Site	
5	Spectrometer	Avance
6	Author	
7	Solvent	CDCl3
8	Temperature	294.7
9	Pulse Sequence	zg30
10	Experiment	1D
11	Number of Scans	32
12	Receiver Gain	101
13	Relaxation Delay	1.0000
14	Pulse Width	7.9700
15	Acquisition Time	4.1943
16	Acquisition Date	2022-08-11T18:03:42
17	Modification Date	2022-08-11T18:12:35
18	Spectrometer Frequency	400.17
19	Spectral Width	7812.5
20	Lowest Frequency	-1444.8
21	Nucleus	1H
22	Acquired Size	32768
23	Spectral Size	65536

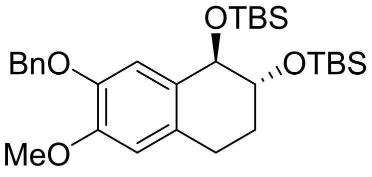

S9

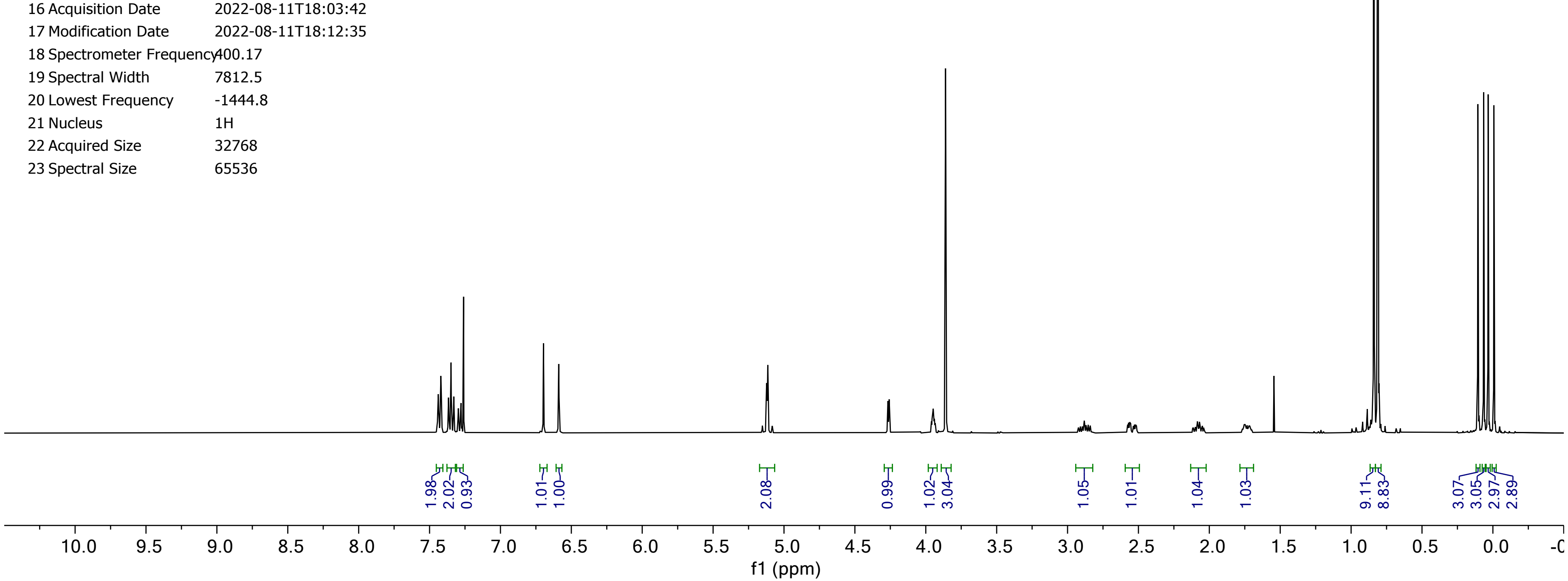

	Parameter	Value
1	Sample Name	060901-11BP-001-060.2.fid
2	Origin	Bruker BioSpin GmbH
3	Owner	nmrsu
4	Site	
5	Spectrometer	Avance
6	Author	
7	Solvent	CDCl3
8	Temperature	294.7
9	Pulse Sequence	zgpg30
10	Experiment	1D
11	Number of Scans	10000
12	Receiver Gain	101
13	Relaxation Delay	2.0000
14	Pulse Width	8.0000
15	Acquisition Time	1.3763
16	Acquisition Date	2022-08-12T03:57:04
17	Modification Date	2022-08-12T11:35:03
18	Spectrometer Frequency	100.63
19	Spectral Width	23809.5
20	Lowest Frequency	-842.1
21	Nucleus	13C
22	Acquired Size	32768
23	Spectral Size	65536

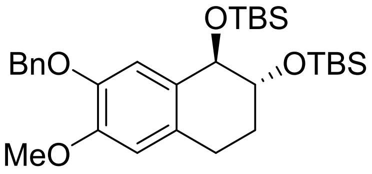

S9

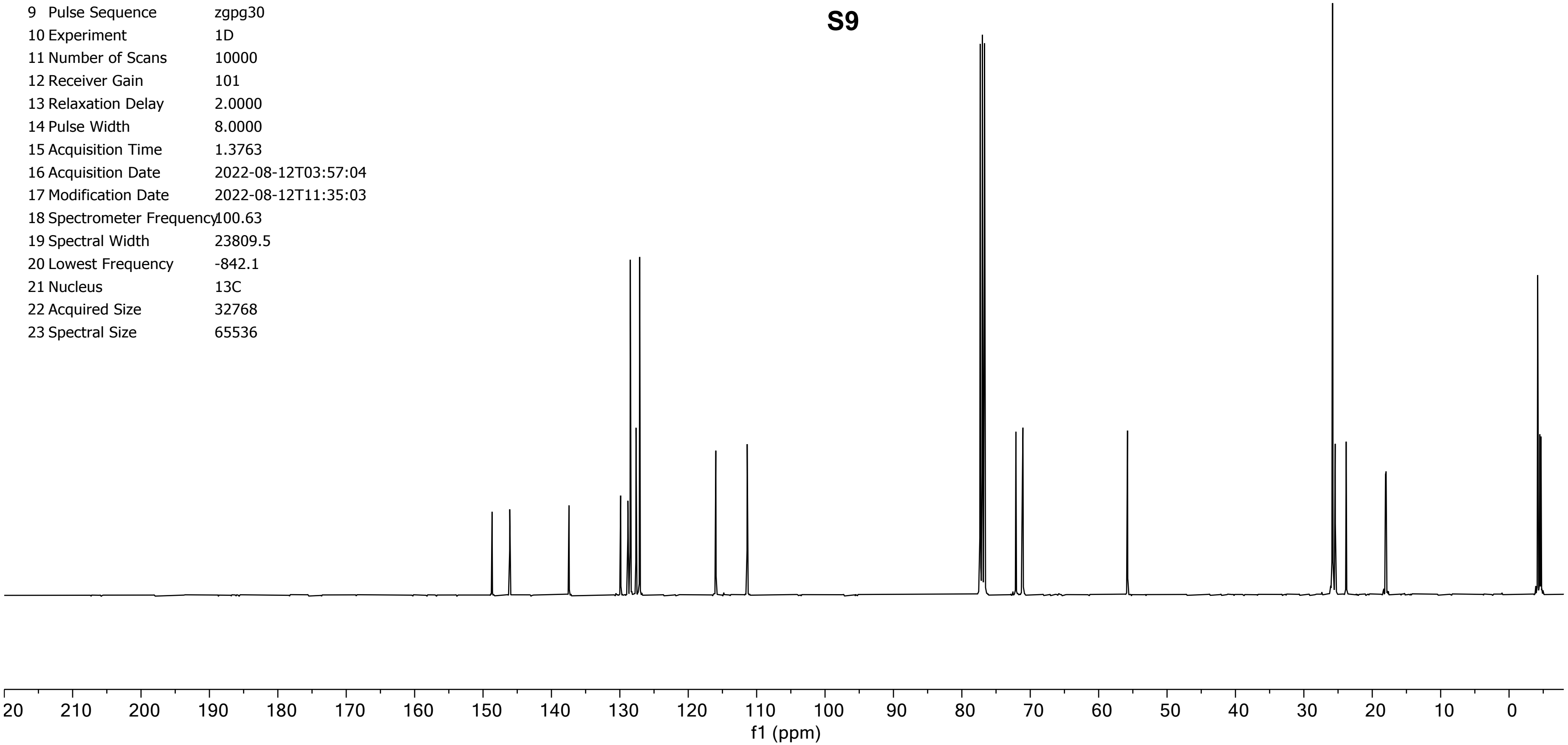

	Parameter	Value
1	Sample Name	060901-11BP-001-064.2.fid
2	Origin	Bruker BioSpin GmbH
3	Owner	nmrsu
4	Site	
5	Spectrometer	Avance
6	Author	
7	Solvent	CDCl3
8	Temperature	294.7
9	Pulse Sequence	zg30
10	Experiment	1D
11	Number of Scans	64
12	Receiver Gain	101
13	Relaxation Delay	1.0000
14	Pulse Width	7.9700
15	Acquisition Time	4.1943
16	Acquisition Date	2022-08-13T20:33:25
17	Modification Date	2022-08-13T20:33:31
18	Spectrometer Frequency	400.17
19	Spectral Width	7812.5
20	Lowest Frequency	-1444.8
21	Nucleus	1H
22	Acquired Size	32768
23	Spectral Size	65536

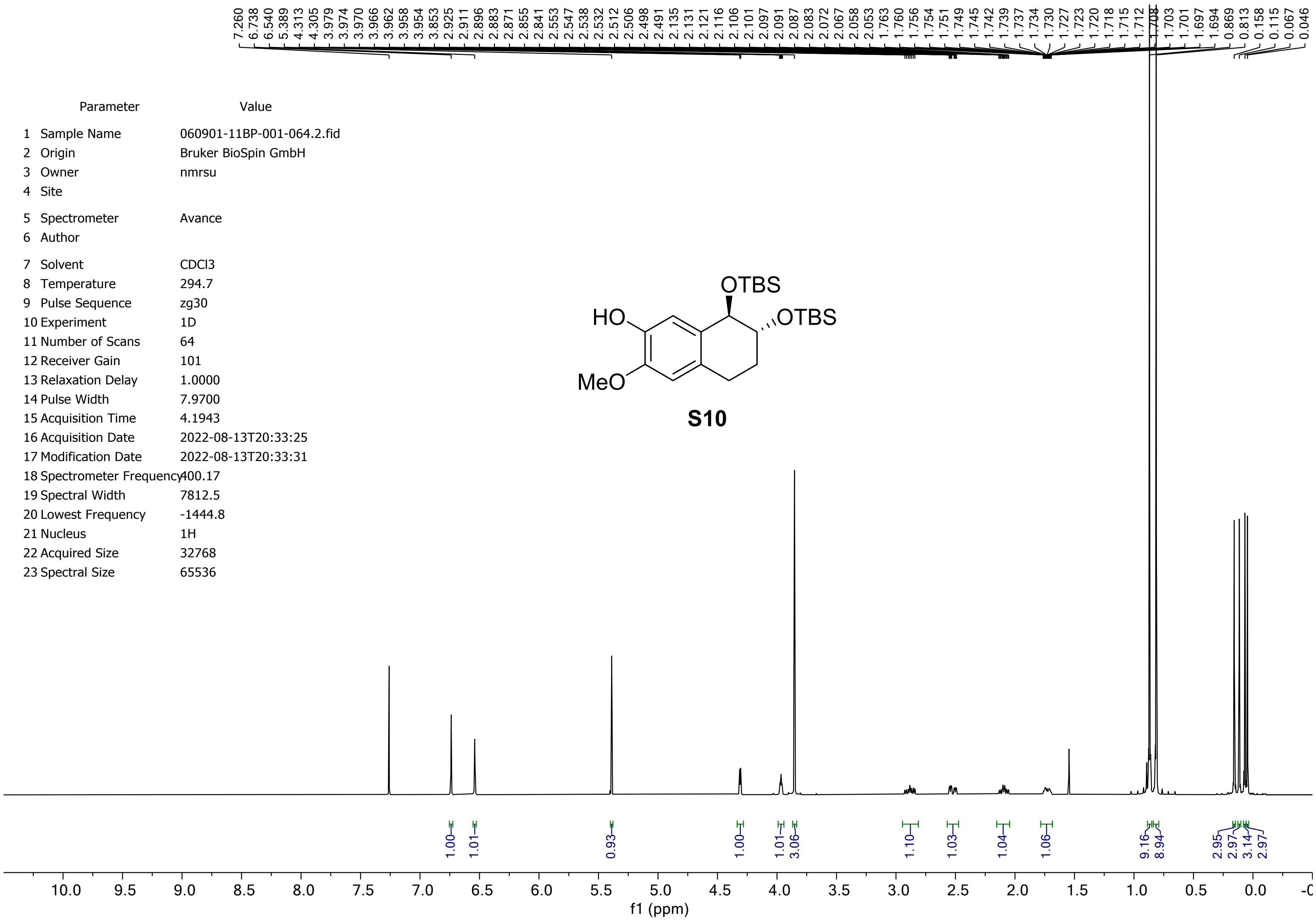

	Parameter	Value
1	Sample Name	060901-11BP-001-064.3.fid
2	Origin	Bruker BioSpin GmbH
3	Owner	nmrsu
4	Site	
5	Spectrometer	Avance
6	Author	
7	Solvent	CDCl3
8	Temperature	294.7
9	Pulse Sequence	zgpg30
10	Experiment	1D
11	Number of Scans	12000
12	Receiver Gain	101
13	Relaxation Delay	2.0000
14	Pulse Width	8.0000
15	Acquisition Time	1.3763
16	Acquisition Date	2022-08-14T08:04:44
17	Modification Date	2022-08-14T12:47:31
18	Spectrometer Frequency	100.63
19	Spectral Width	23809.5
20	Lowest Frequency	-840.0
21	Nucleus	13C
22	Acquired Size	32768
23	Spectral Size	65536

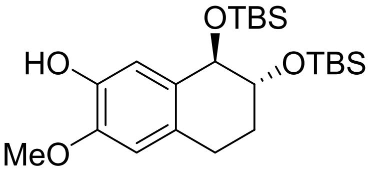

S10

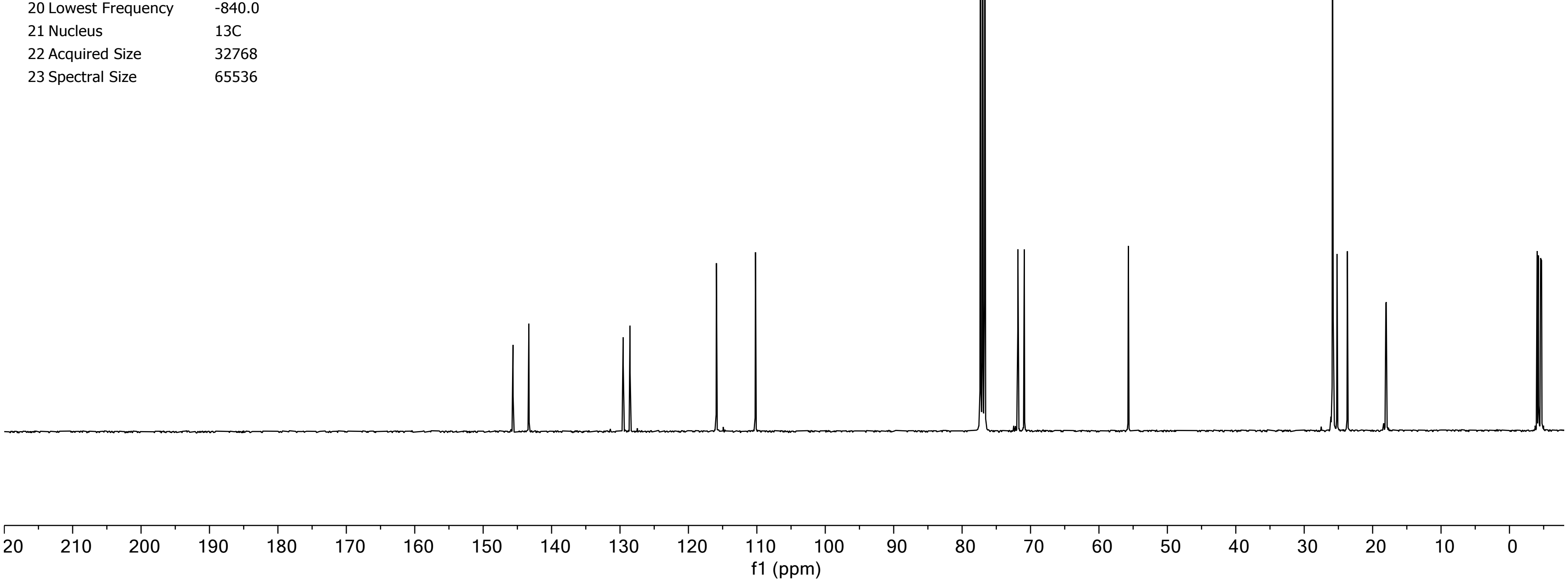

	Parameter	Value
1	Sample Name	I68YyI8gQX+DgZdrxzQbaA.1.fid
2	Origin	Bruker BioSpin GmbH
3	Owner	nmrsu
4	Site	
5	Spectrometer	Avance
6	Author	
7	Solvent	CDCl3
8	Temperature	298.0
9	Pulse Sequence	zg30
10	Experiment	1D
11	Number of Scans	16
12	Receiver Gain	32
13	Relaxation Delay	1.0000
14	Pulse Width	10.0000
15	Acquisition Time	2.7525
16	Acquisition Date	2021-02-25T11:47:33
17	Modification Date	2022-08-22T17:56:00
18	Spectrometer Frequency	600.14
19	Spectral Width	11904.8
20	Lowest Frequency	-2260.4
21	Nucleus	1H
22	Acquired Size	32768
23	Spectral Size	131072

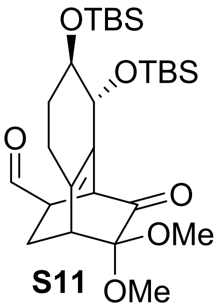

S11

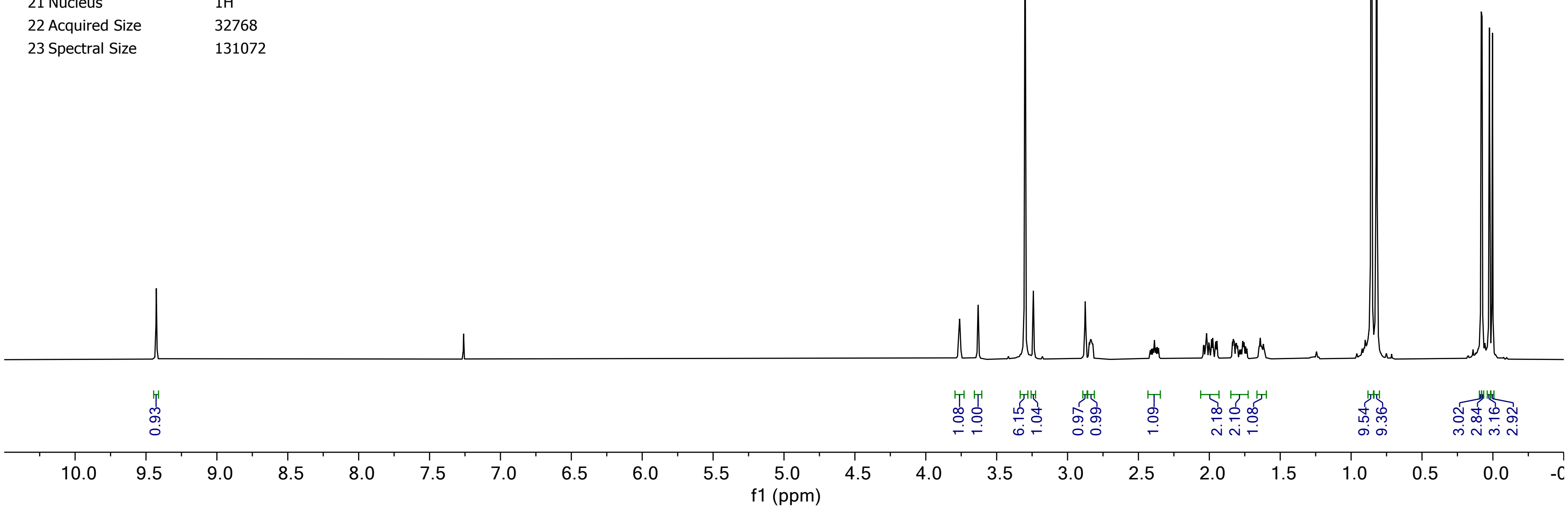

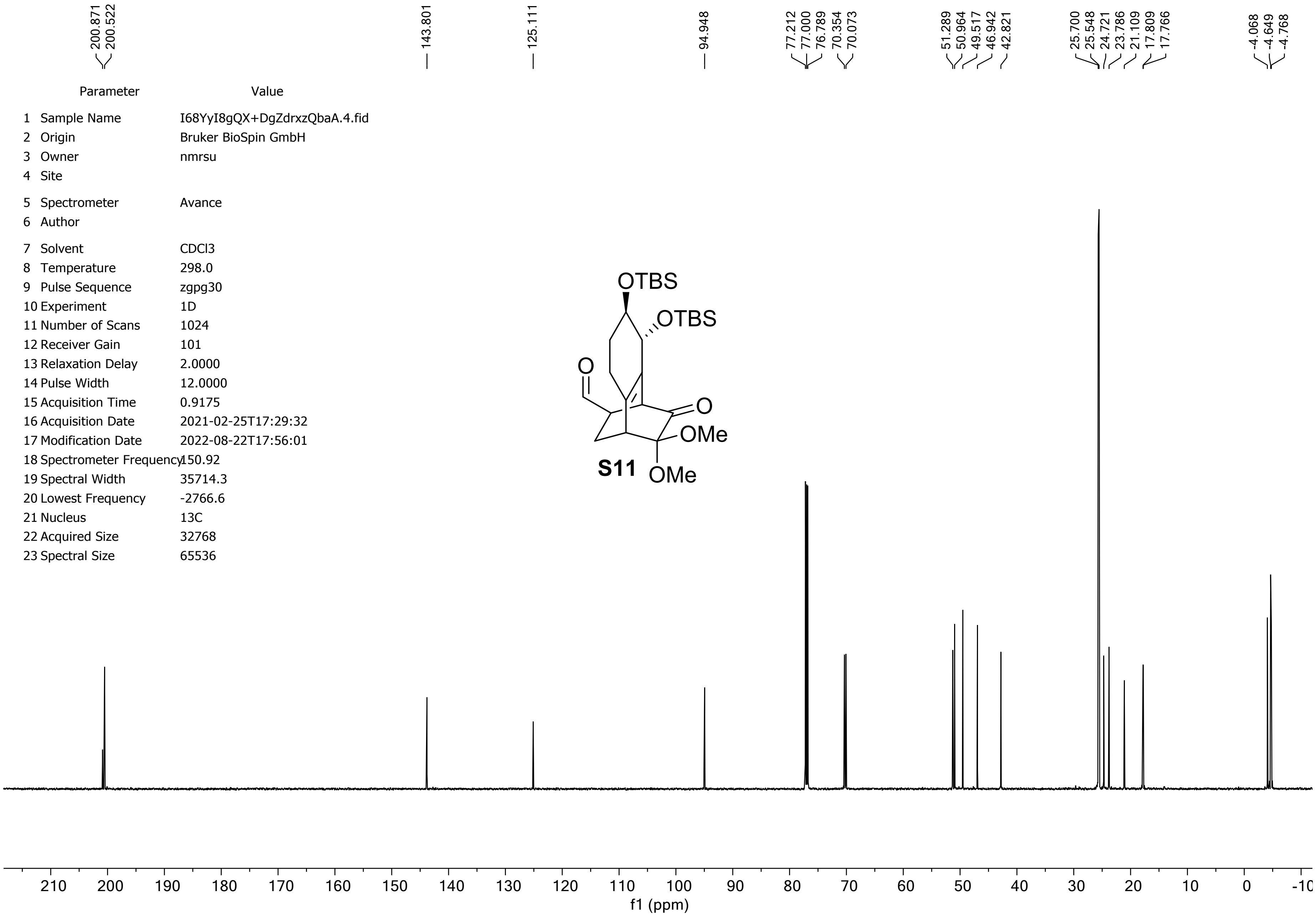

	Parameter	Value
1	Sample Name	I68YyI8gQX+DgZdrxzQbaA.4.fid
2	Origin	Bruker BioSpin GmbH
3	Owner	nmrsu
4	Site	
5	Spectrometer	Avance
6	Author	
7	Solvent	CDCl3
8	Temperature	298.0
9	Pulse Sequence	zgpg30
10	Experiment	1D
11	Number of Scans	1024
12	Receiver Gain	101
13	Relaxation Delay	2.0000
14	Pulse Width	12.0000
15	Acquisition Time	0.9175
16	Acquisition Date	2021-02-25T17:29:32
17	Modification Date	2022-08-22T17:56:01
18	Spectrometer Frequency	150.92
19	Spectral Width	35714.3
20	Lowest Frequency	-2766.6
21	Nucleus	13C
22	Acquired Size	32768
23	Spectral Size	65536

S11

	Parameter	Value
1	Sample Name	060901-12BP-030-014-B-PURE.1.fid
2	Origin	Bruker BioSpin GmbH
3	Owner	nmrsu
4	Site	
5	Spectrometer	Avance
6	Author	
7	Solvent	CDCl3
8	Temperature	303.0
9	Pulse Sequence	zg30
10	Experiment	1D
11	Number of Scans	32
12	Receiver Gain	90
13	Relaxation Delay	1.0000
14	Pulse Width	8.0000
15	Acquisition Time	4.1943
16	Acquisition Date	2023-08-17T00:01:17
17	Modification Date	2023-08-17T11:51:07
18	Spectrometer Frequency	400.17
19	Spectral Width	7812.5
20	Lowest Frequency	-1435.2
21	Nucleus	1H
22	Acquired Size	32768
23	Spectral Size	65536

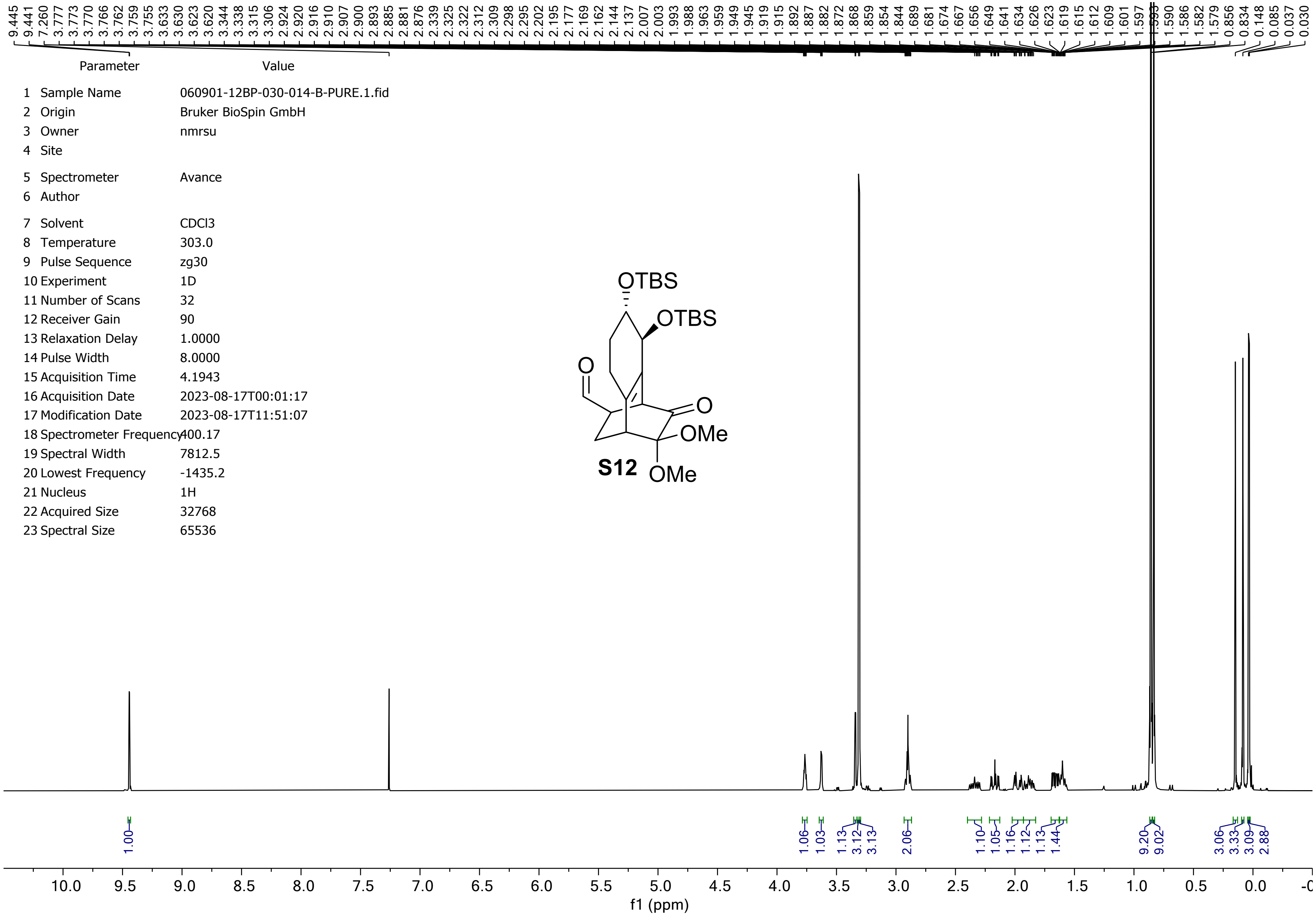

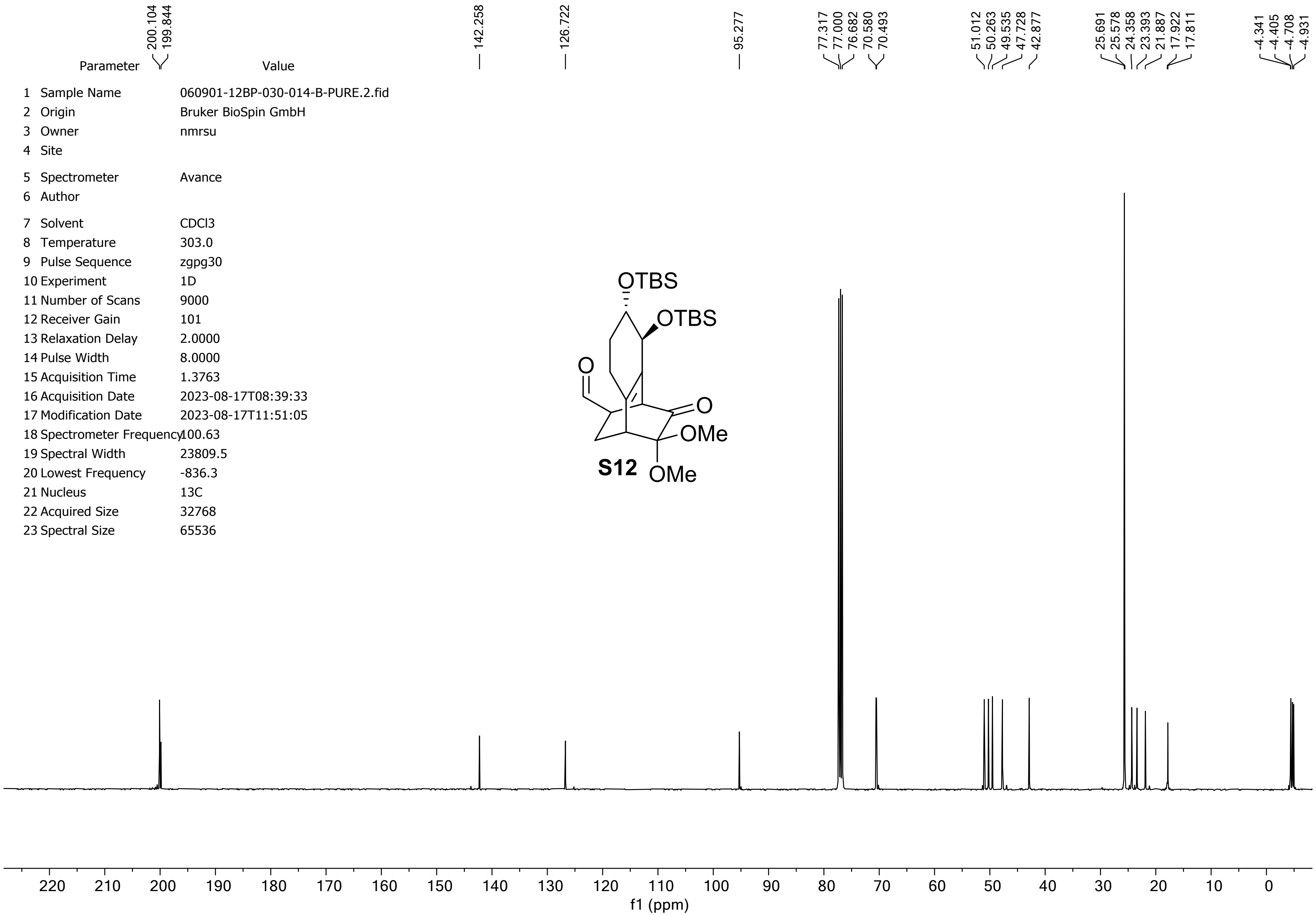

	Parameter	Value
1	Sample Name	060901-12BP-030-014-B-PURE.2.fid
2	Origin	Bruker BioSpin GmbH
3	Owner	nmrsu
4	Site	
5	Spectrometer	Avance
6	Author	
7	Solvent	CDCl3
8	Temperature	303.0
9	Pulse Sequence	zgpg30
10	Experiment	1D
11	Number of Scans	9000
12	Receiver Gain	101
13	Relaxation Delay	2.0000
14	Pulse Width	8.0000
15	Acquisition Time	1.3763
16	Acquisition Date	2023-08-17T08:39:33
17	Modification Date	2023-08-17T11:51:05
18	Spectrometer Frequency	100.63
19	Spectral Width	23809.5
20	Lowest Frequency	-836.3
21	Nucleus	13C
22	Acquired Size	32768
23	Spectral Size	65536

S12

	Parameter	Value
1	Sample Name	060901-09BP-086-046-3.1.fid
2	Origin	Bruker BioSpin GmbH
3	Owner	nmrsu
4	Site	
5	Spectrometer	Avance
6	Author	
7	Solvent	CDCl3
8	Temperature	294.7
9	Pulse Sequence	zg30
10	Experiment	1D
11	Number of Scans	32
12	Receiver Gain	90
13	Relaxation Delay	1.0000
14	Pulse Width	7.9700
15	Acquisition Time	4.1943
16	Acquisition Date	2022-08-29T22:25:22
17	Modification Date	2022-08-29T22:29:01
18	Spectrometer Frequency	400.17
19	Spectral Width	7812.5
20	Lowest Frequency	-1435.2
21	Nucleus	1H
22	Acquired Size	32768
23	Spectral Size	65536

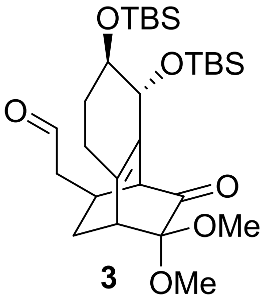

3

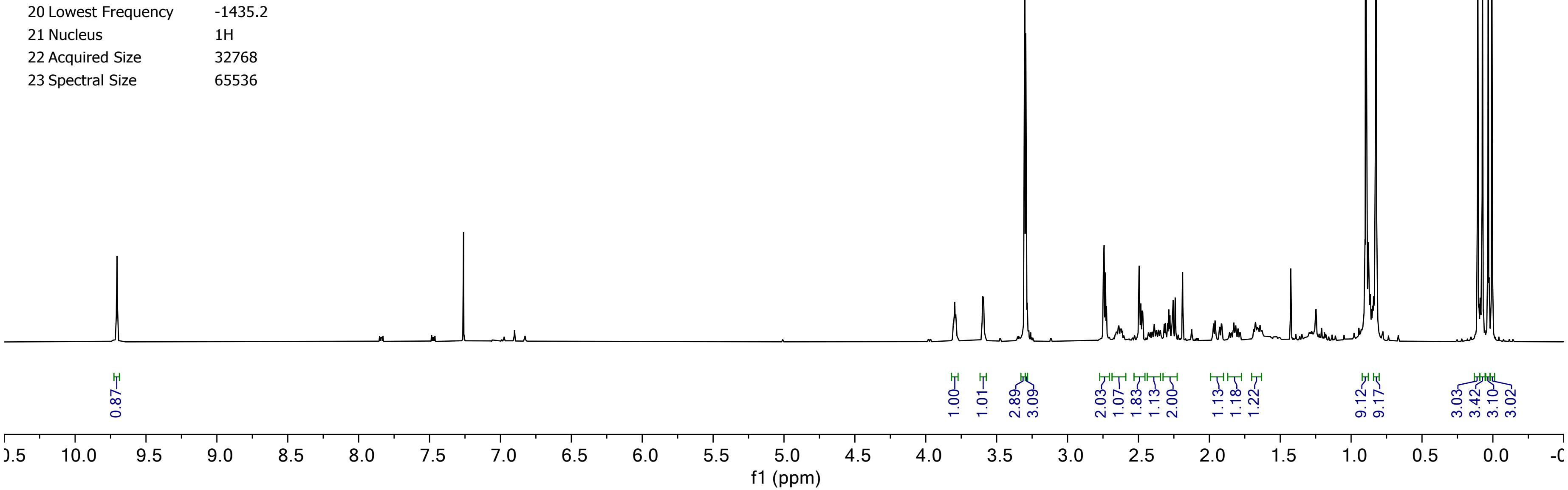

Parameter		Value
1	Sample Name	060901-09BP-086-046-13C.1.fid
2	Origin	Bruker BioSpin GmbH
3	Owner	nmrsu
4	Site	
5	Spectrometer	Avance
6	Author	
7	Solvent	CDCl3
8	Temperature	294.7
9	Pulse Sequence	zgpg30
10	Experiment	1D
11	Number of Scans	9000
12	Receiver Gain	101
13	Relaxation Delay	2.0000
14	Pulse Width	8.0000
15	Acquisition Time	1.3763
16	Acquisition Date	2022-08-30T07:21:36
17	Modification Date	2022-08-30T09:21:04
18	Spectrometer Frequency	100.63
19	Spectral Width	23809.5
20	Lowest Frequency	-840.3
21	Nucleus	13C
22	Acquired Size	32768
23	Spectral Size	65536

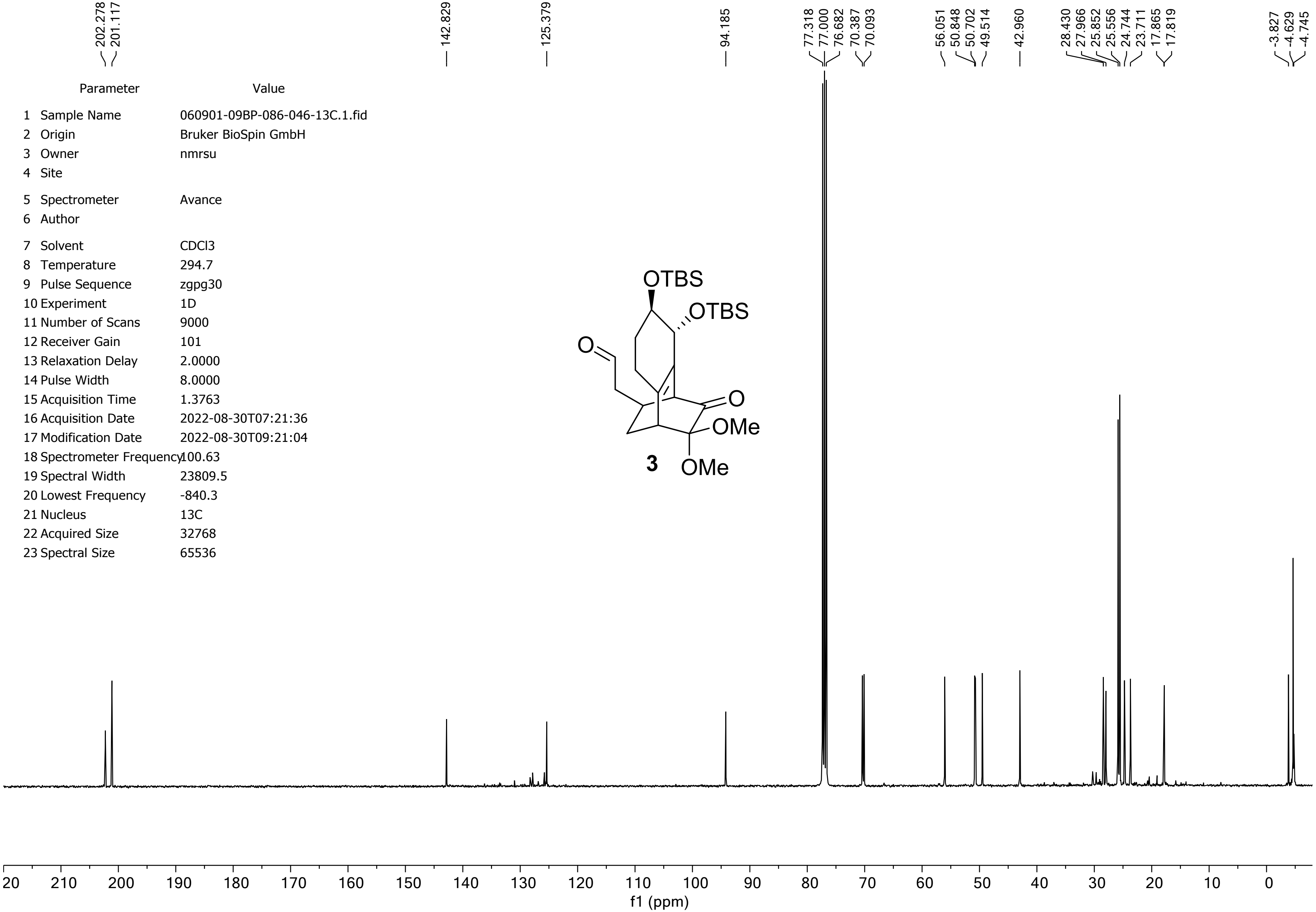

3

	Parameter	值
1	Sample Name	qHTkLExhR46YwsjcDXa$sA.1.fid
2	Origin	Bruker BioSpin GmbH
3	Owner	nmrsu
4	Site	
5	Spectrometer	Avance
6	Author	
7	Solvent	CDCl3
8	Temperature	298.2
9	Pulse Sequence	zg30
10	Experiment	1D
11	Number of Scans	32
12	Receiver Gain	32
13	Relaxation Delay	1.0000
14	Pulse Width	10.0000
15	Acquisition Time	2.7525
16	Acquisition Date	2024-03-14T21:52:43
17	Modification Date	2024-04-01T11:02:59
18	Spectrometer Frequency	600.14
19	Spectral Width	11904.8
20	Lowest Frequency	-2260.3
21	Nucleus	1H
22	Acquired Size	32768
23	Spectral Size	131072

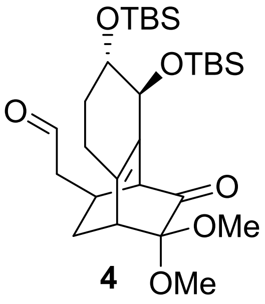

4

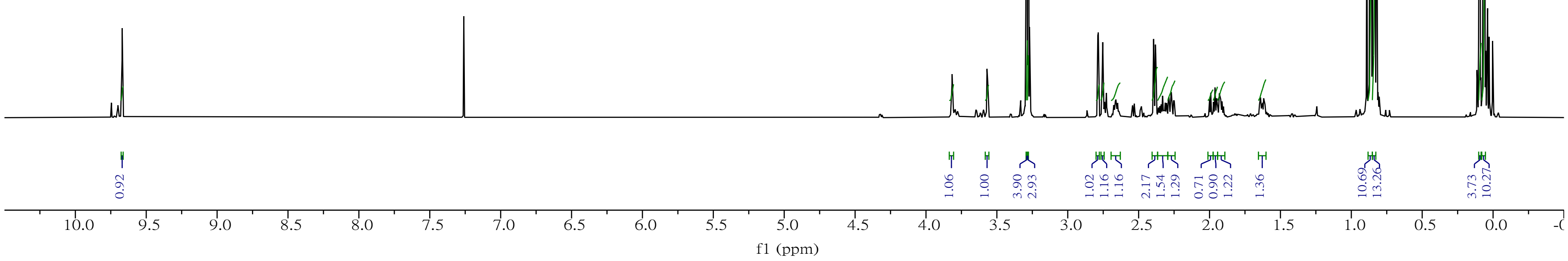

	Parameter	值
1	Sample Name	qHTkLExhR46YwsjcDXa$sA.2.fid
2	Origin	Bruker BioSpin GmbH
3	Owner	nmrsu
4	Site	
5	Spectrometer	Avance
6	Author	
7	Solvent	CDCl3
8	Temperature	298.2
9	Pulse Sequence	zgpg30
10	Experiment	1D
11	Number of Scans	8000
12	Receiver Gain	90
13	Relaxation Delay	2.0000
14	Pulse Width	12.0000
15	Acquisition Time	0.9175
16	Acquisition Date	2024-03-15T04:31:10
17	Modification Date	2024-04-01T11:02:59
18	Spectrometer Frequency	150.92
19	Spectral Width	35714.3
20	Lowest Frequency	-1262.2
21	Nucleus	13C
22	Acquired Size	32768
23	Spectral Size	65536

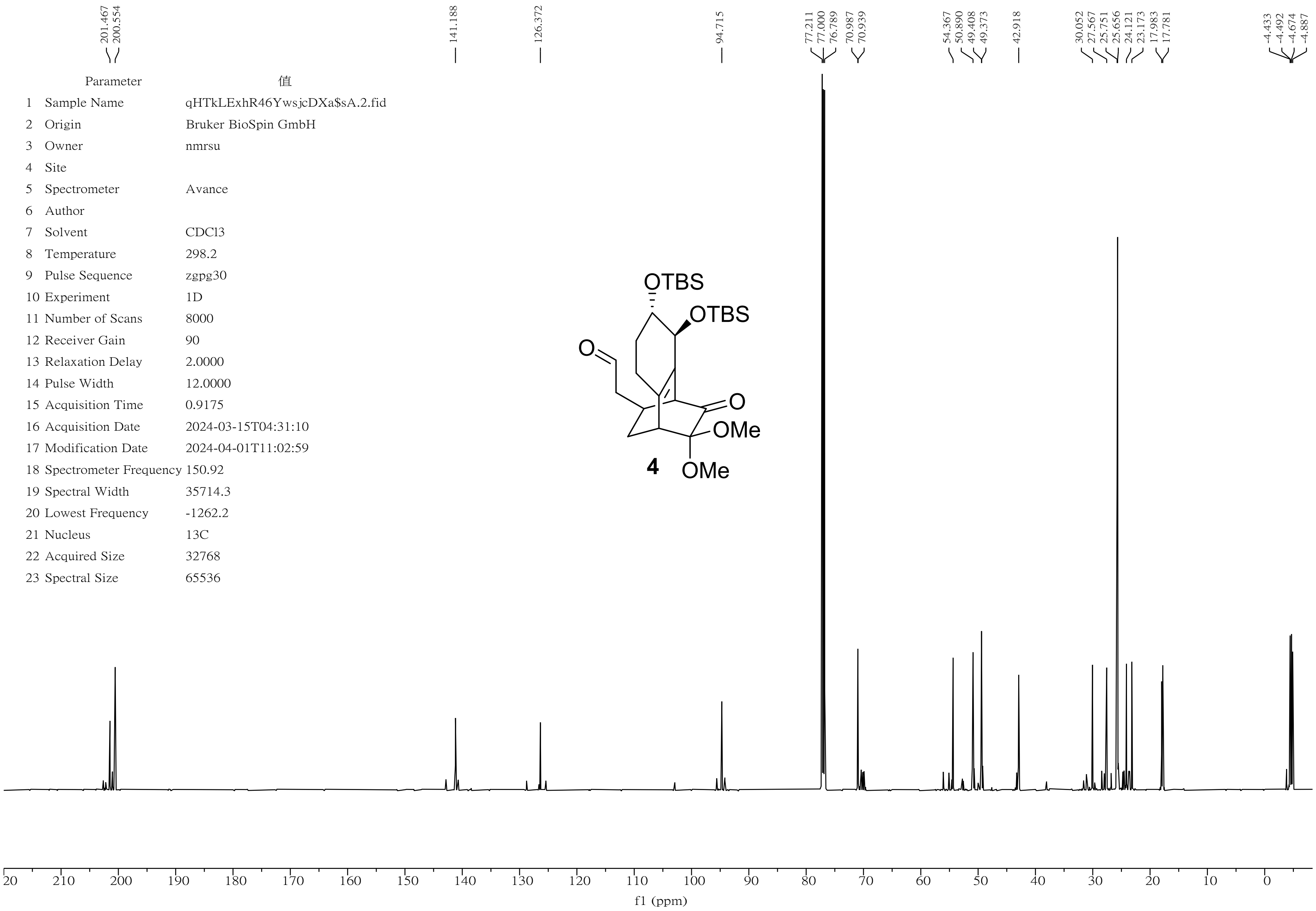

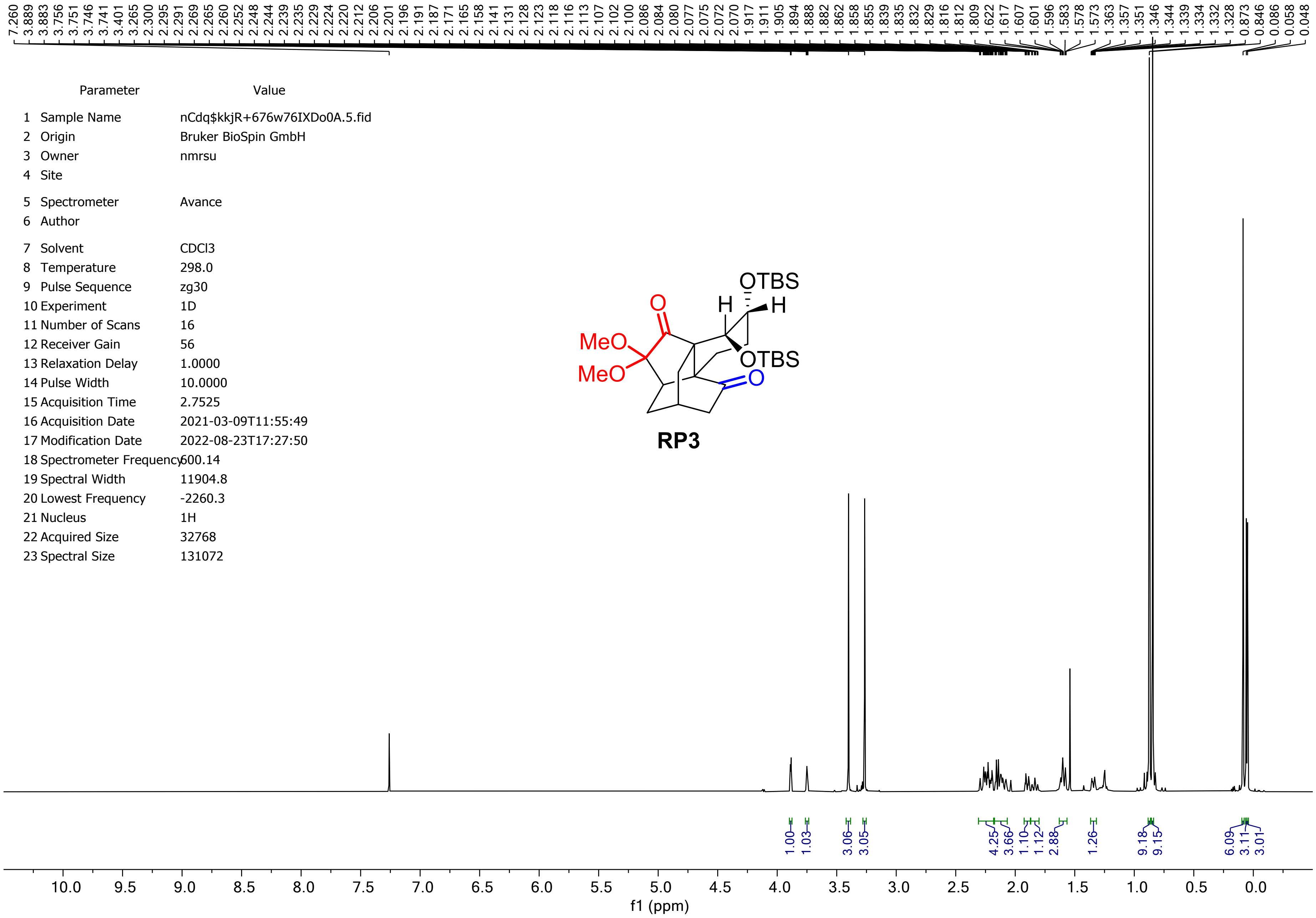

	Parameter	Value
1	Sample Name	nCdq$kkjR+676w76IXDo0A.5.fid
2	Origin	Bruker BioSpin GmbH
3	Owner	nmrsu
4	Site	
5	Spectrometer	Avance
6	Author	
7	Solvent	CDCl3
8	Temperature	298.0
9	Pulse Sequence	zg30
10	Experiment	1D
11	Number of Scans	16
12	Receiver Gain	56
13	Relaxation Delay	1.0000
14	Pulse Width	10.0000
15	Acquisition Time	2.7525
16	Acquisition Date	2021-03-09T11:55:49
17	Modification Date	2022-08-23T17:27:50
18	Spectrometer Frequency	600.14
19	Spectral Width	11904.8
20	Lowest Frequency	-2260.3
21	Nucleus	1H
22	Acquired Size	32768
23	Spectral Size	131072

RP3

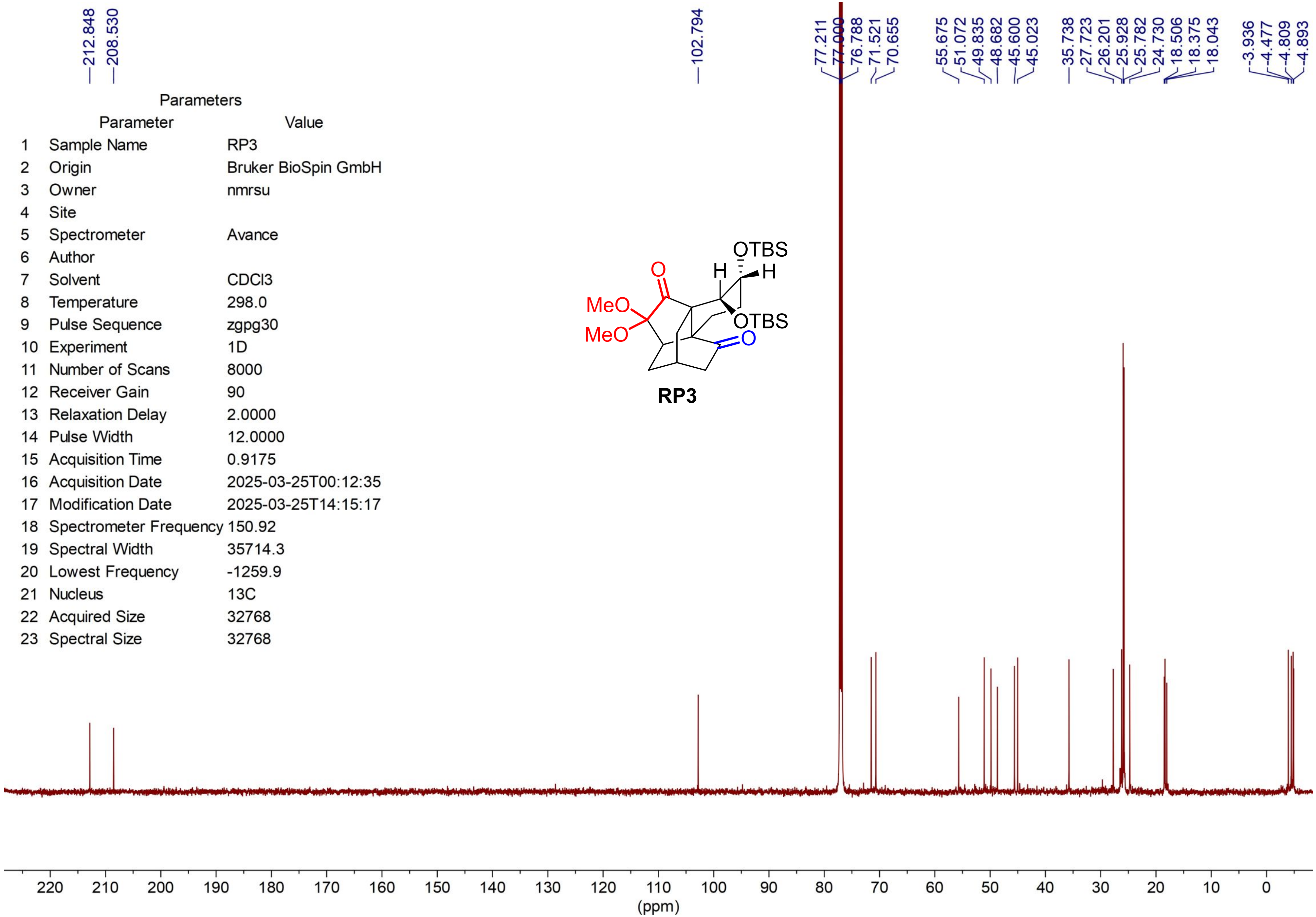

Parameters

	Parameter	Value
1	Sample Name	RP3
2	Origin	Bruker BioSpin GmbH
3	Owner	nmrsu
4	Site	
5	Spectrometer	Avance
6	Author	
7	Solvent	CDCl3
8	Temperature	298.0
9	Pulse Sequence	zgpg30
10	Experiment	1D
11	Number of Scans	8000
12	Receiver Gain	90
13	Relaxation Delay	2.0000
14	Pulse Width	12.0000
15	Acquisition Time	0.9175
16	Acquisition Date	2025-03-25T00:12:35
17	Modification Date	2025-03-25T14:15:17
18	Spectrometer Frequency	150.92
19	Spectral Width	35714.3
20	Lowest Frequency	-1259.9
21	Nucleus	13C
22	Acquired Size	32768
23	Spectral Size	32768

	Parameter	Value
1	Sample Name	060901-10BP-034-086-DMSO.2.fid
2	Origin	Bruker BioSpin GmbH
3	Owner	nmrsu
4	Site	
5	Spectrometer	Avance
6	Author	
7	Solvent	DMSO
8	Temperature	294.7
9	Pulse Sequence	zg30
10	Experiment	1D
11	Number of Scans	32
12	Receiver Gain	101
13	Relaxation Delay	1.0000
14	Pulse Width	8.0000
15	Acquisition Time	4.1943
16	Acquisition Date	2022-08-31T11:53:49
17	Modification Date	2022-08-31T11:57:19
18	Spectrometer Frequency	400.17
19	Spectral Width	7812.5
20	Lowest Frequency	-1438.6
21	Nucleus	1H
22	Acquired Size	32768
23	Spectral Size	65536

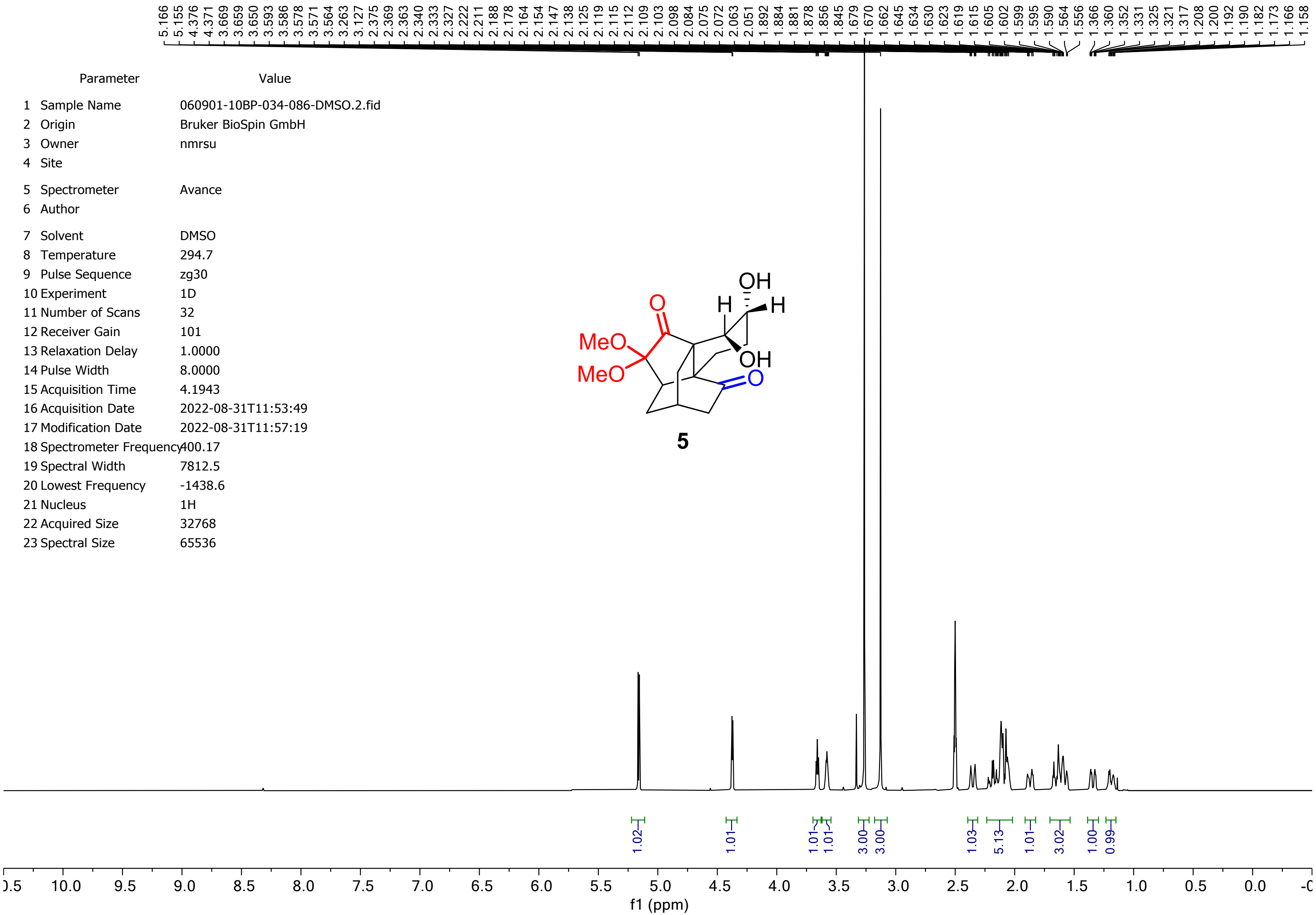

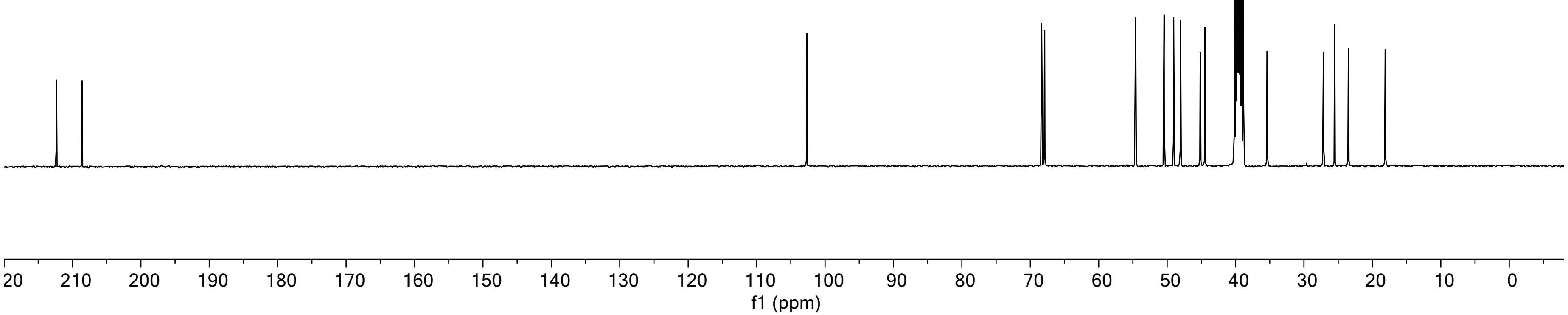

	Parameter	Value
1	Sample Name	060901-10BP-034-086-13C.1.fid
2	Origin	Bruker BioSpin GmbH
3	Owner	nmrsu
4	Site	
5	Spectrometer	Avance
6	Author	
7	Solvent	CDCl3
8	Temperature	294.7
9	Pulse Sequence	zgpg30
10	Experiment	1D
11	Number of Scans	10000
12	Receiver Gain	101
13	Relaxation Delay	2.0000
14	Pulse Width	8.0000
15	Acquisition Time	1.3763
16	Acquisition Date	2022-09-02T04:30:30
17	Modification Date	2022-09-02T09:43:04
18	Spectrometer Frequency	100.63
19	Spectral Width	23809.5
20	Lowest Frequency	-1361.3
21	Nucleus	13C
22	Acquired Size	32768
23	Spectral Size	65536

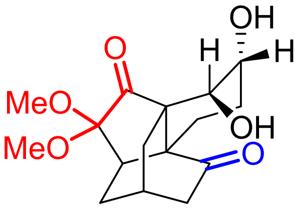

5

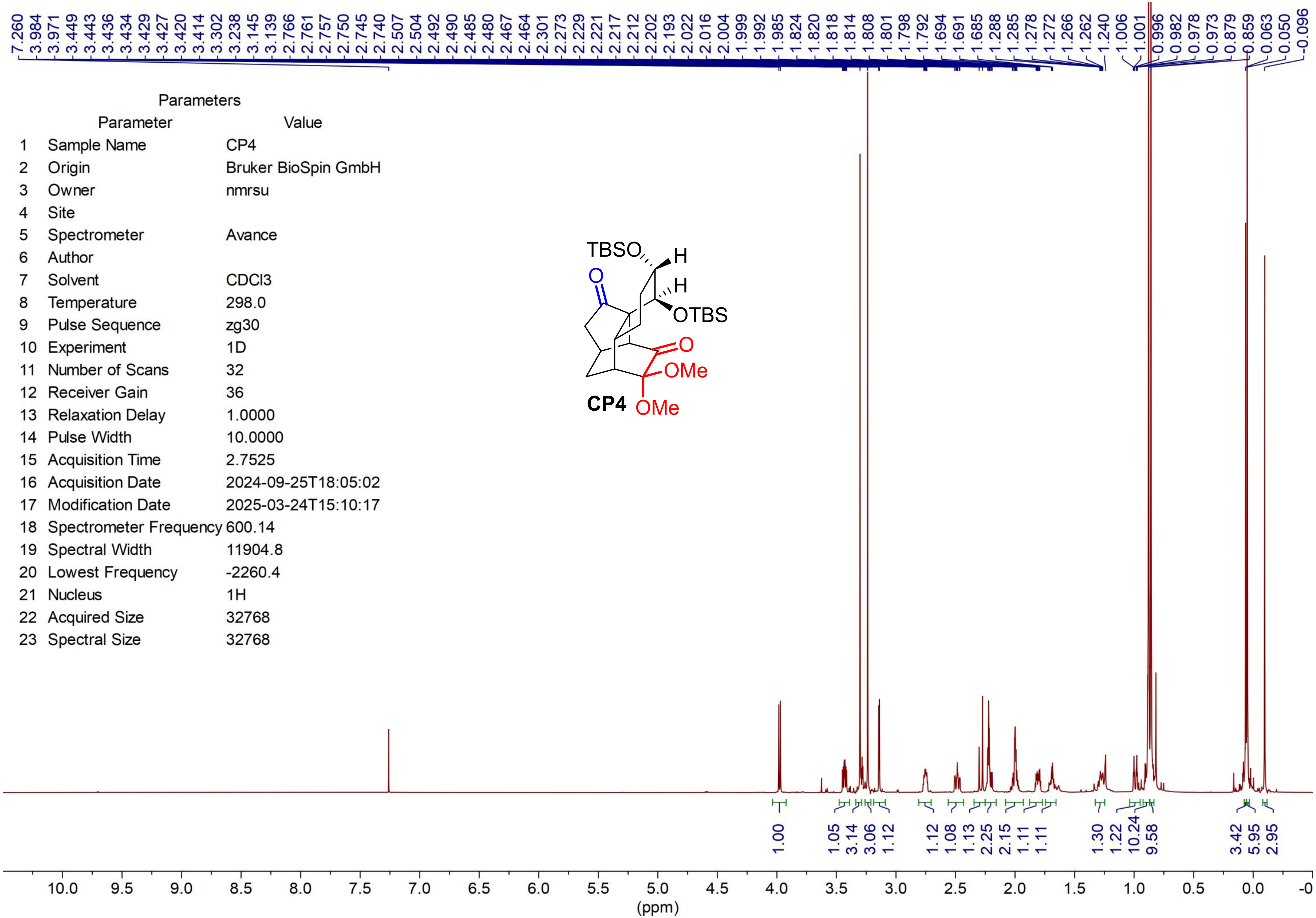

Parameters

	Parameter	Value
1	Sample Name	CP4
2	Origin	Bruker BioSpin GmbH
3	Owner	nmrsu
4	Site	
5	Spectrometer	Avance
6	Author	
7	Solvent	CDCl3
8	Temperature	298.0
9	Pulse Sequence	zg30
10	Experiment	1D
11	Number of Scans	32
12	Receiver Gain	36
13	Relaxation Delay	1.0000
14	Pulse Width	10.0000
15	Acquisition Time	2.7525
16	Acquisition Date	2024-09-25T18:05:02
17	Modification Date	2025-03-24T15:10:17
18	Spectrometer Frequency	600.14
19	Spectral Width	11904.8
20	Lowest Frequency	-2260.4
21	Nucleus	1H
22	Acquired Size	32768
23	Spectral Size	32768

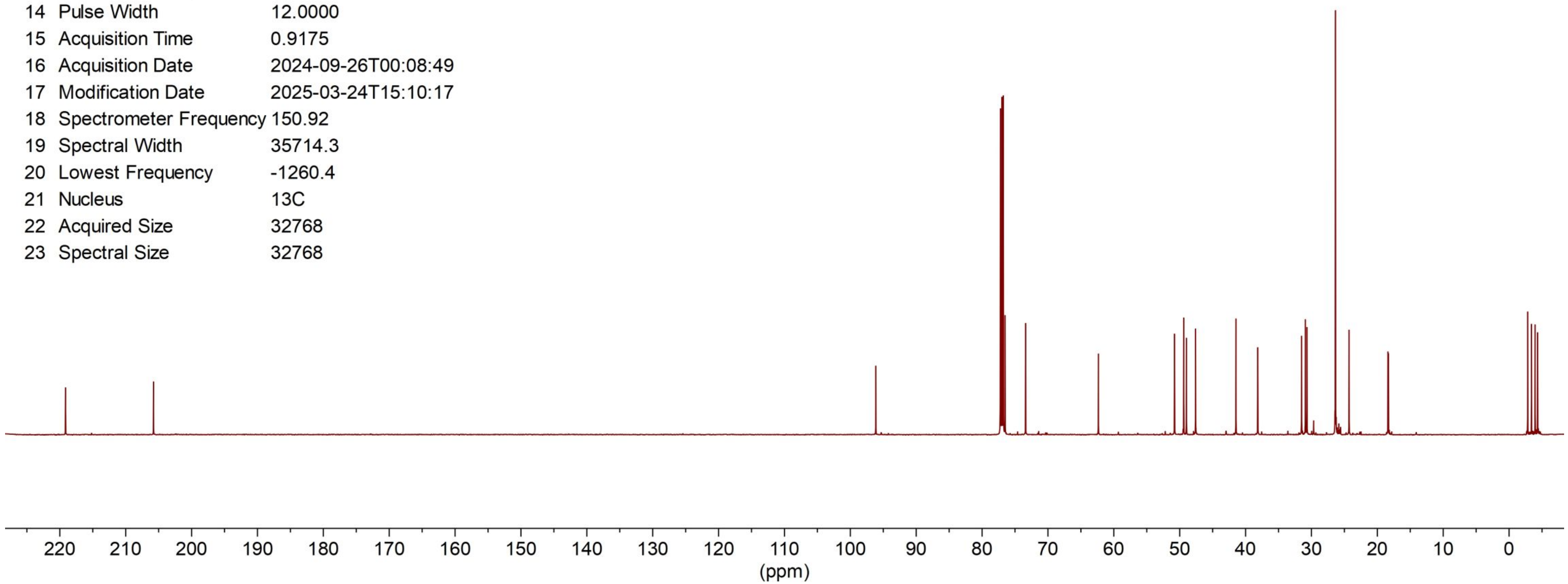

Parameters

	Parameter	Value
1	Sample Name	CP4
2	Origin	Bruker BioSpin GmbH
3	Owner	nmrsu
4	Site	
5	Spectrometer	Avance
6	Author	
7	Solvent	CDCl3
8	Temperature	298.0
9	Pulse Sequence	zgpg30
10	Experiment	1D
11	Number of Scans	7000
12	Receiver Gain	101
13	Relaxation Delay	2.0000
14	Pulse Width	12.0000
15	Acquisition Time	0.9175
16	Acquisition Date	2024-09-26T00:08:49
17	Modification Date	2025-03-24T15:10:17
18	Spectrometer Frequency	150.92
19	Spectral Width	35714.3
20	Lowest Frequency	-1260.4
21	Nucleus	13C
22	Acquired Size	32768
23	Spectral Size	32768

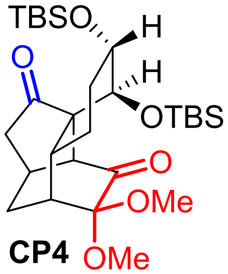

CP4

Parameters

	Parameter	Value
1	Sample Name	olefin 6
2	Origin	Bruker BioSpin GmbH
3	Owner	nmrsu
4	Site	
5	Spectrometer	Avance
6	Author	
7	Solvent	CDCl3
8	Temperature	298.0
9	Pulse Sequence	zg30
10	Experiment	1D
11	Number of Scans	32
12	Receiver Gain	101
13	Relaxation Delay	1.0000
14	Pulse Width	10.0000
15	Acquisition Time	2.7525
16	Acquisition Date	2024-09-28T18:19:02
17	Modification Date	2025-03-24T16:23:59
18	Spectrometer Frequency	600.14
19	Spectral Width	11904.8
20	Lowest Frequency	-2260.3
21	Nucleus	1H
22	Acquired Size	32768
23	Spectral Size	32768

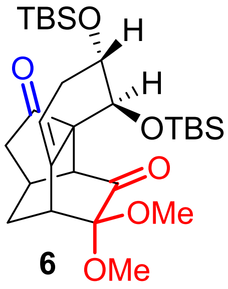

6

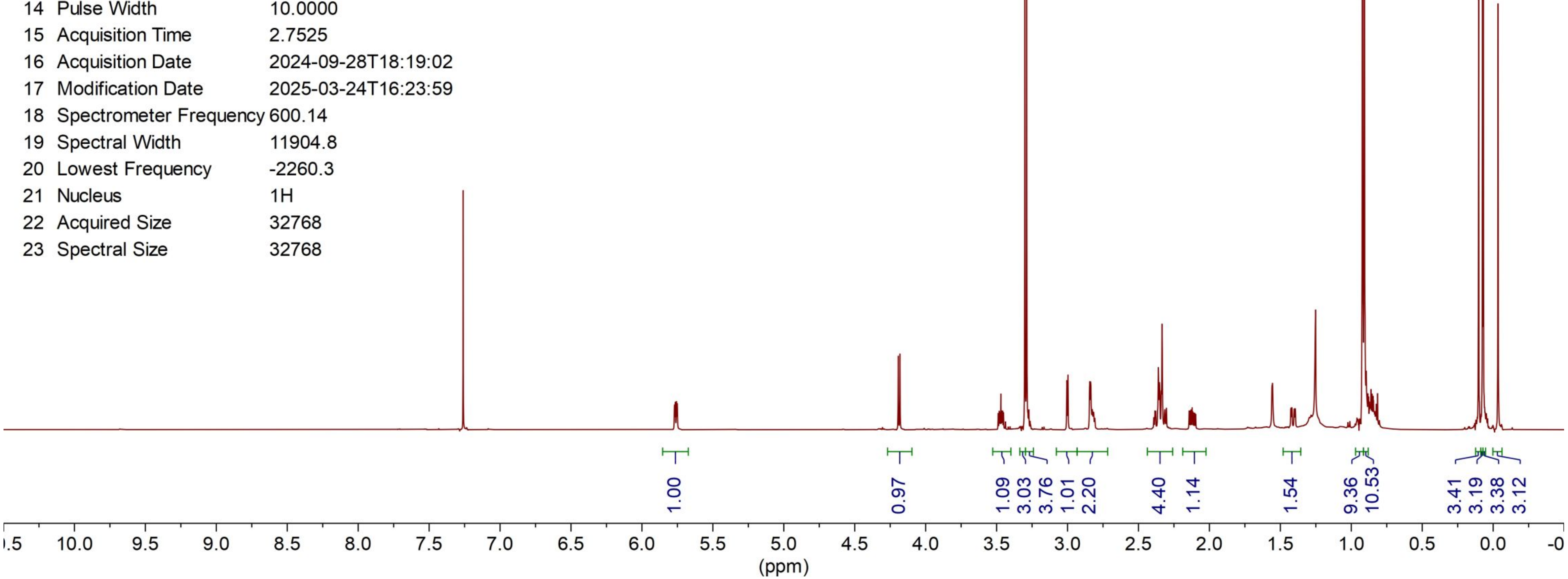

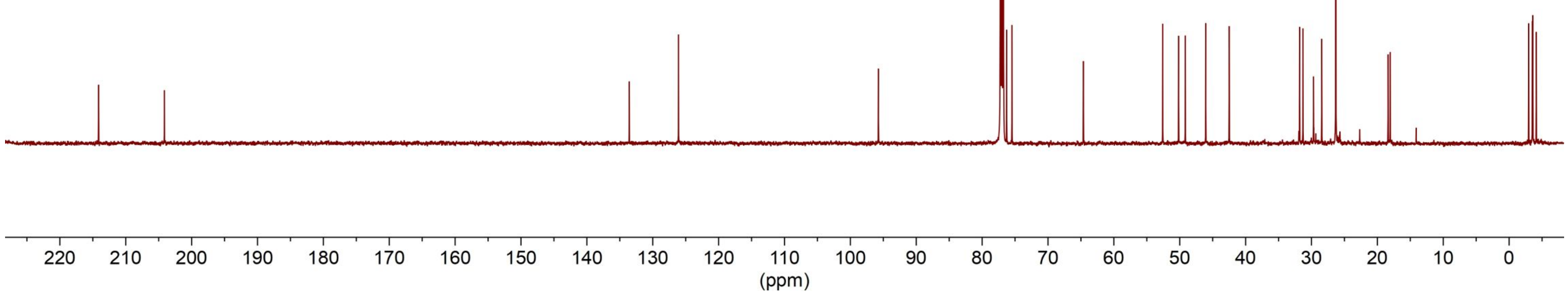

Parameters

	Parameter	Value
1	Sample Name	olefin 6
2	Origin	Bruker BioSpin GmbH
3	Owner	nmrsu
4	Site	
5	Spectrometer	Avance
6	Author	
7	Solvent	CDCl3
8	Temperature	298.0
9	Pulse Sequence	zgpg30
10	Experiment	1D
11	Number of Scans	7000
12	Receiver Gain	101
13	Relaxation Delay	2.0000
14	Pulse Width	12.0000
15	Acquisition Time	0.9175
16	Acquisition Date	2024-09-29T00:09:02
17	Modification Date	2025-03-24T16:23:59
18	Spectrometer Frequency	150.92
19	Spectral Width	35714.3
20	Lowest Frequency	-1259.5
21	Nucleus	13C
22	Acquired Size	32768
23	Spectral Size	32768

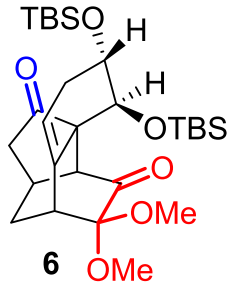

6

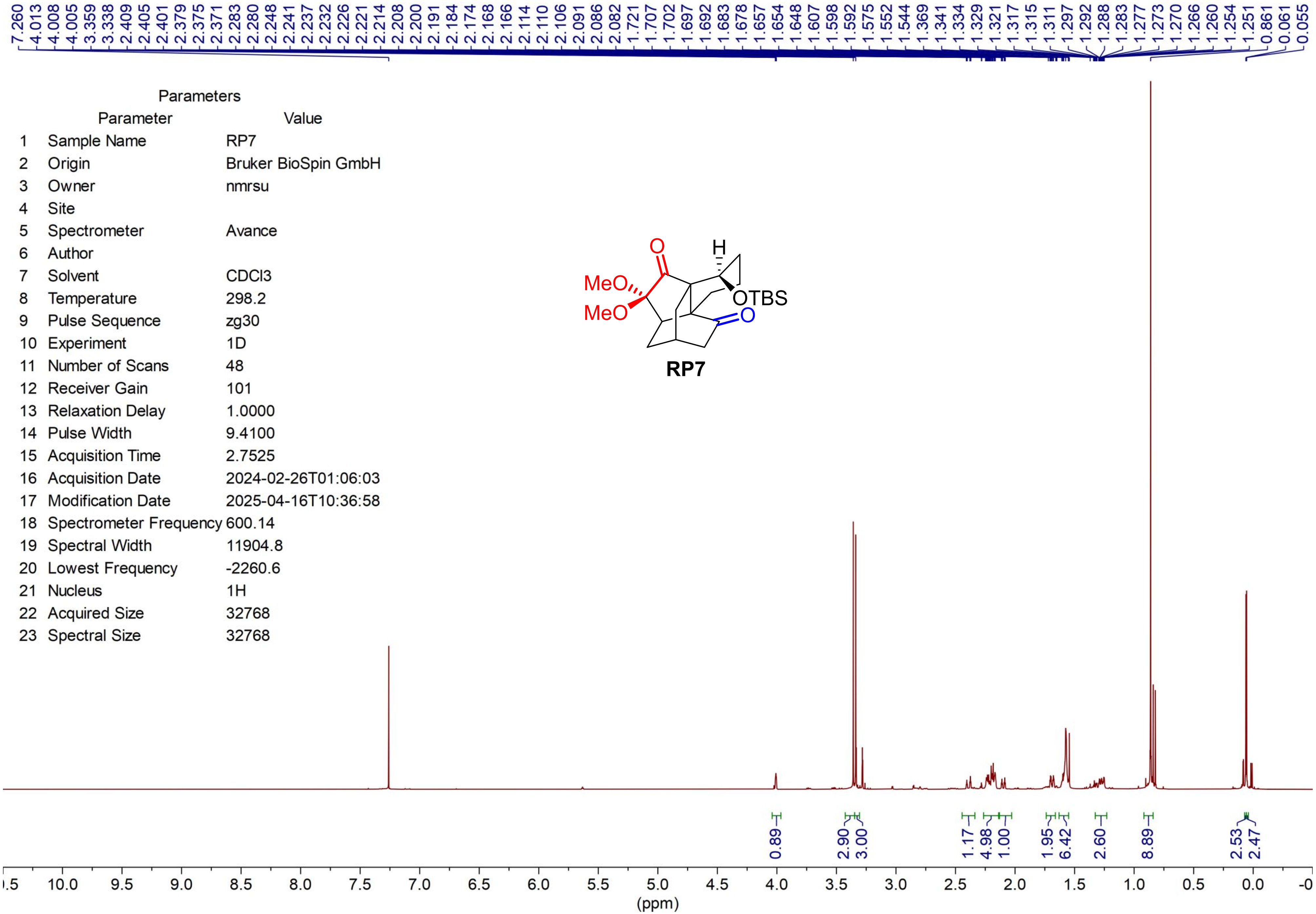

Parameters

	Parameter	Value
1	Sample Name	RP7
2	Origin	Bruker BioSpin GmbH
3	Owner	nmrsu
4	Site	
5	Spectrometer	Avance
6	Author	
7	Solvent	CDCl3
8	Temperature	298.2
9	Pulse Sequence	zg30
10	Experiment	1D
11	Number of Scans	48
12	Receiver Gain	101
13	Relaxation Delay	1.0000
14	Pulse Width	9.4100
15	Acquisition Time	2.7525
16	Acquisition Date	2024-02-26T01:06:03
17	Modification Date	2025-04-16T10:36:58
18	Spectrometer Frequency	600.14
19	Spectral Width	11904.8
20	Lowest Frequency	-2260.6
21	Nucleus	1H
22	Acquired Size	32768
23	Spectral Size	32768

RP7

Parameters

	Parameter	Value
1	Sample Name	JpvitSMmTJyobsB$+cT2ww.1.fid
2	Origin	Bruker BioSpin GmbH
3	Owner	nmrsu
4	Site	
5	Spectrometer	Avance
6	Author	
7	Solvent	CDCl3
8	Temperature	298.2
9	Pulse Sequence	zgpg30
10	Experiment	1D
11	Number of Scans	10000
12	Receiver Gain	90
13	Relaxation Delay	2.0000
14	Pulse Width	12.0000
15	Acquisition Time	0.9175
16	Acquisition Date	2024-02-26T09:34:59
17	Modification Date	2025-04-16T10:36:51
18	Spectrometer Frequency	150.92
19	Spectral Width	35714.3
20	Lowest Frequency	-1258.7
21	Nucleus	13C
22	Acquired Size	32768
23	Spectral Size	32768

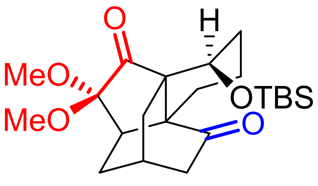

RP7

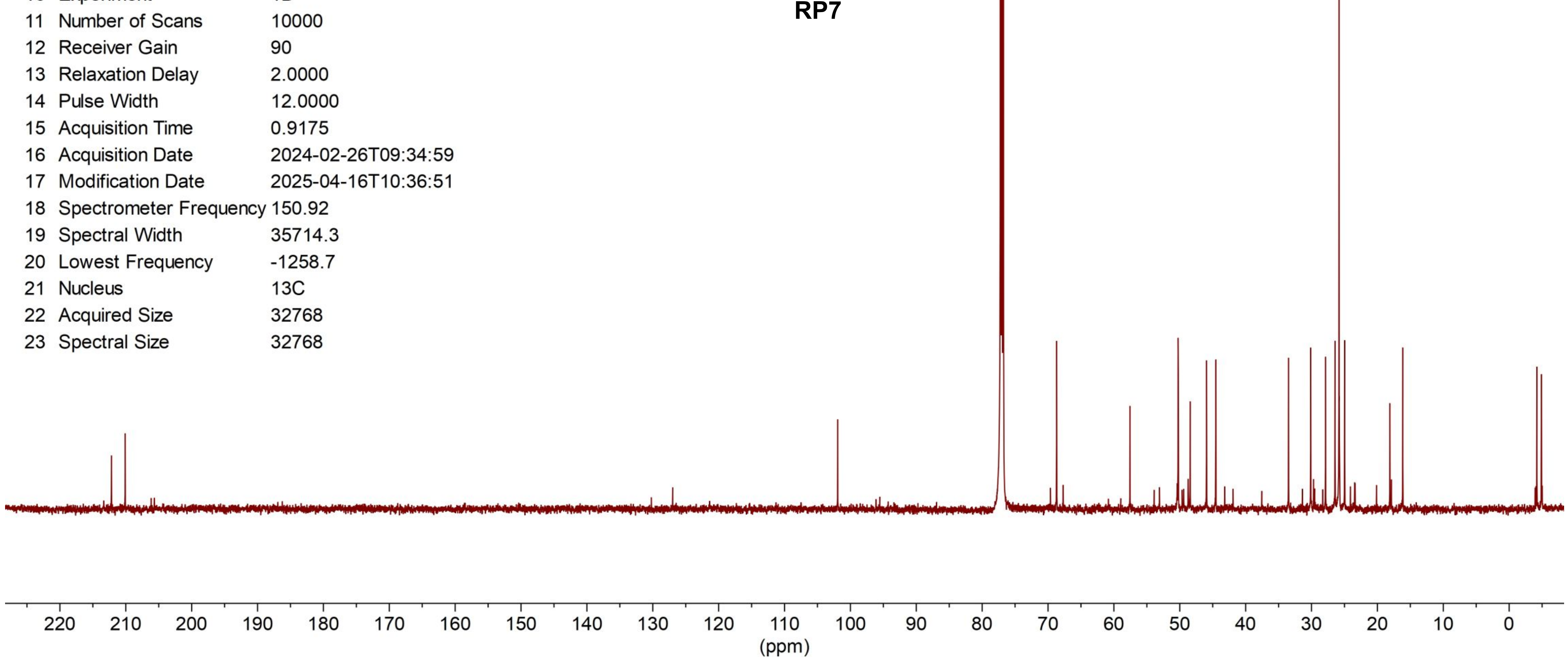

Parameter		Value
1	Sample Name	094716-EXP-22-AB3614-A3_CDCl3.1.fid
2	Origin	Bruker BioSpin GmbH
3	Owner	nmrsu
4	Site	
5	Spectrometer	Avance
6	Author	
7	Solvent	CDCl3
8	Temperature	294.7
9	Pulse Sequence	zg30
10	Experiment	1D
11	Number of Scans	16
12	Receiver Gain	30
13	Relaxation Delay	1.0000
14	Pulse Width	10.6000
15	Acquisition Time	2.7525
16	Acquisition Date	2022-10-20T01:21:08
17	Modification Date	2022-10-20T15:21:39
18	Spectrometer Frequency	600.14
19	Spectral Width	11904.8
20	Lowest Frequency	-2261.3
21	Nucleus	1H
22	Acquired Size	32768
23	Spectral Size	131072

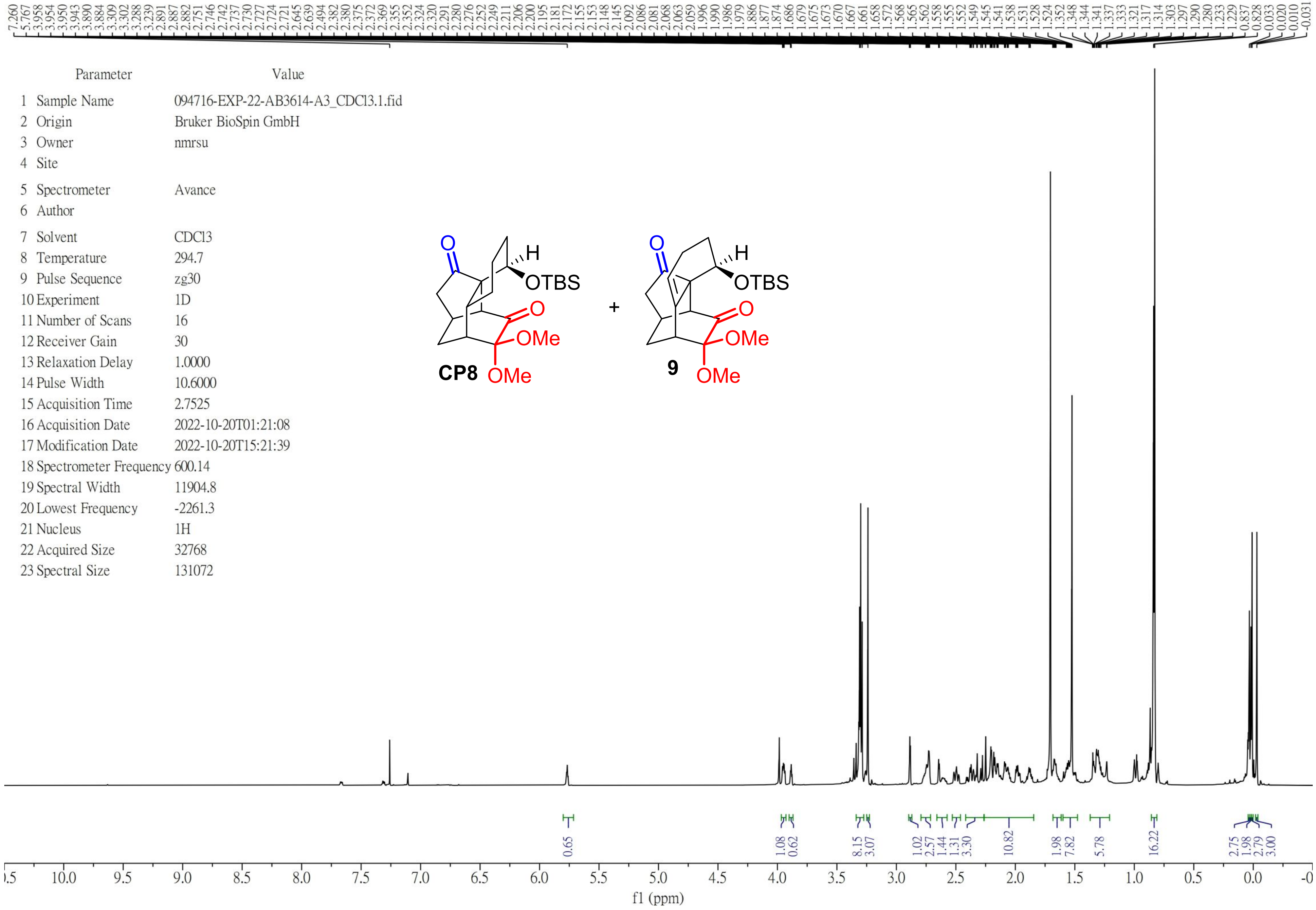

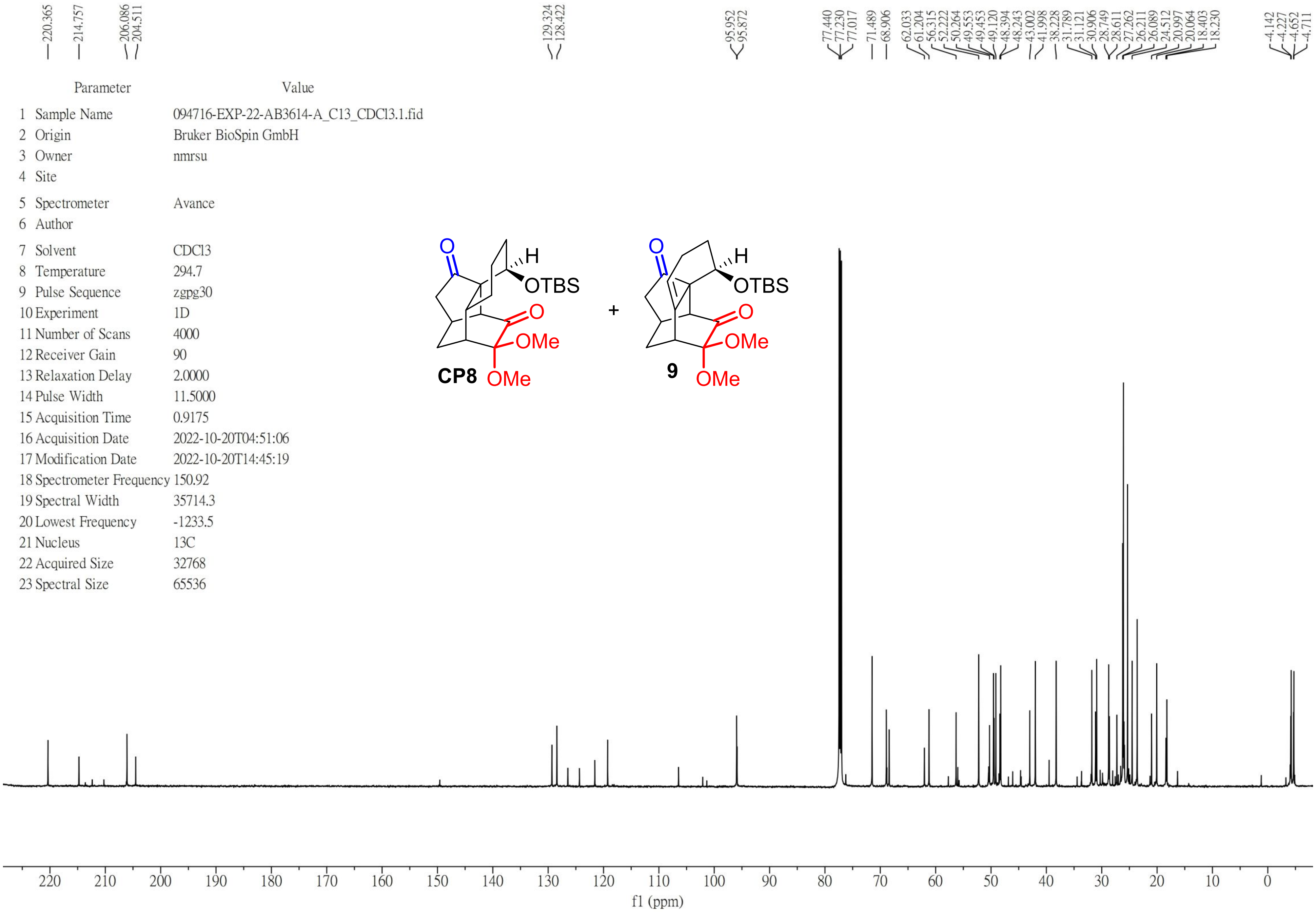

	Parameter	Value
1	Sample Name	094716-EXP-22-AB3614-A_C13_CDCl3.1.fid
2	Origin	Bruker BioSpin GmbH
3	Owner	nmrsu
4	Site	
5	Spectrometer	Avance
6	Author	
7	Solvent	CDCl3
8	Temperature	294.7
9	Pulse Sequence	zgpg30
10	Experiment	1D
11	Number of Scans	4000
12	Receiver Gain	90
13	Relaxation Delay	2.0000
14	Pulse Width	11.5000
15	Acquisition Time	0.9175
16	Acquisition Date	2022-10-20T04:51:06
17	Modification Date	2022-10-20T14:45:19
18	Spectrometer Frequency	150.92
19	Spectral Width	35714.3
20	Lowest Frequency	-1233.5
21	Nucleus	13C
22	Acquired Size	32768
23	Spectral Size	65536

	Parameter	Value
1	Title	PROTON_01
2	Comment	06BP-130-120-7_8
3	Origin	Varian
4	Instrument	mercury
5	Author	
6	Solvent	cdcl3
7	Temperature	25.0
8	Pulse Sequence	s2pul
9	Experiment	1D
10	Probe	Autosw
11	Number of Scans	32
12	Receiver Gain	39
13	Relaxation Delay	1.0000
14	Pulse Width	6.1500
15	Presaturation Frequency	
16	Acquisition Time	1.7064
17	Acquisition Date	2018-05-04T11:14:05
18	Modification Date	2018-05-04T11:14:06
19	Spectrometer Frequency	299.99
20	Spectral Width	4800.8
21	Lowest Frequency	-600.4
22	Nucleus	1H
23	Acquired Size	8192
24	Spectral Size	16384
25	Digital Resolution	0.29

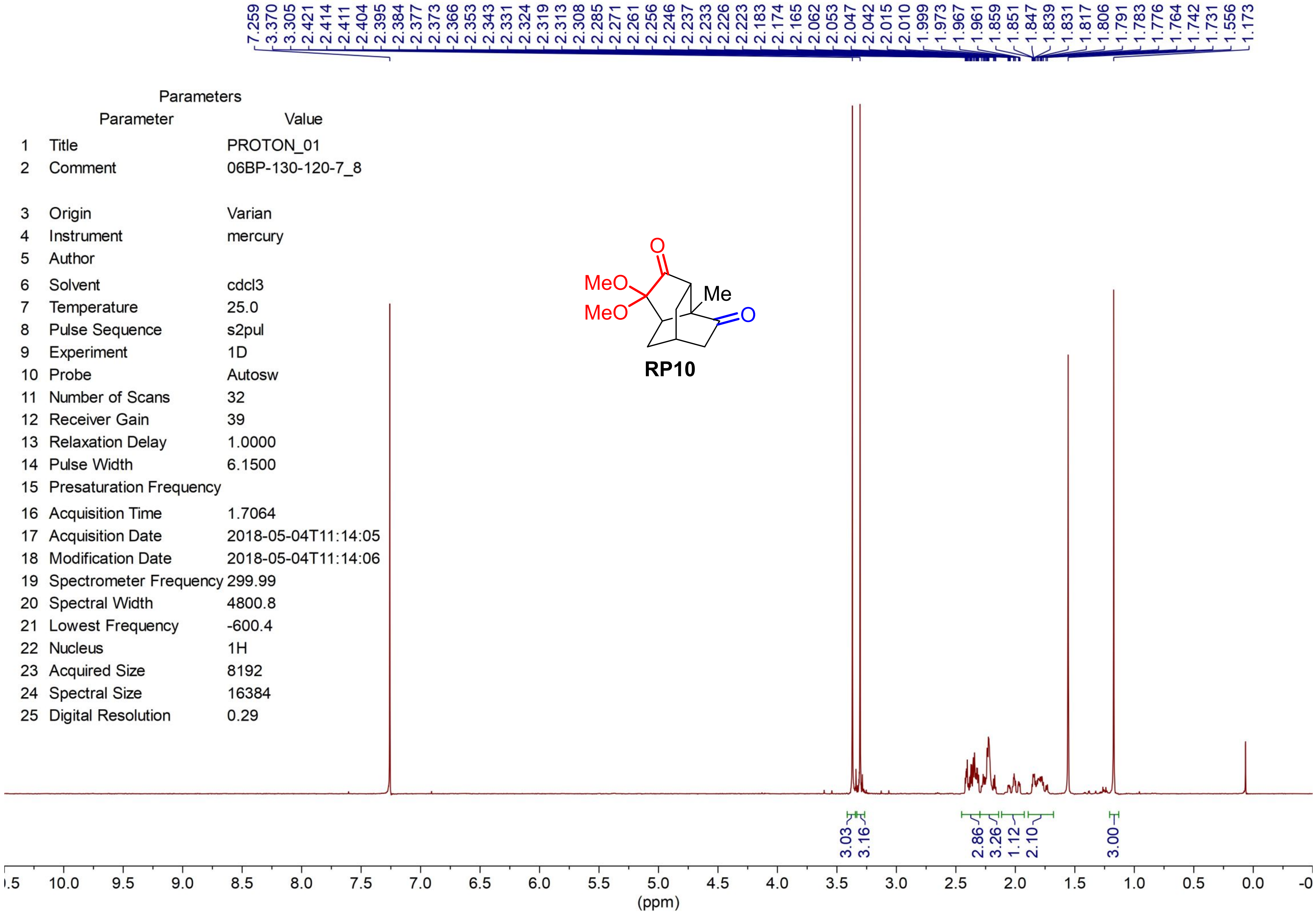

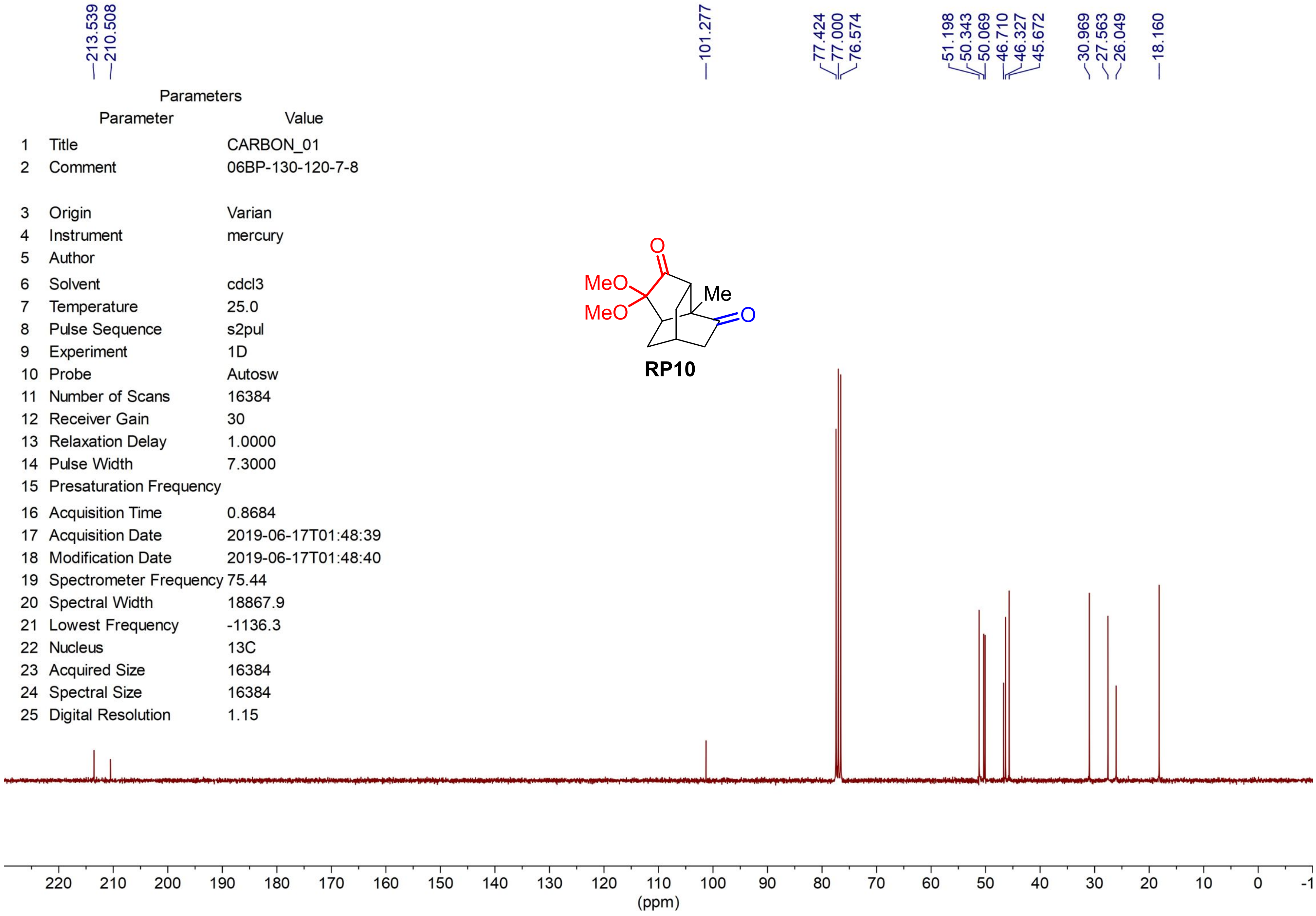

RP10

Parameters

	Parameter	Value
1	Title	CARBON_01
2	Comment	06BP-130-120-7-8
3	Origin	Varian
4	Instrument	mercury
5	Author	
6	Solvent	cdcl3
7	Temperature	25.0
8	Pulse Sequence	s2pul
9	Experiment	1D
10	Probe	Autosw
11	Number of Scans	16384
12	Receiver Gain	30
13	Relaxation Delay	1.0000
14	Pulse Width	7.3000
15	Presaturation Frequency	
16	Acquisition Time	0.8684
17	Acquisition Date	2019-06-17T01:48:39
18	Modification Date	2019-06-17T01:48:40
19	Spectrometer Frequency	75.44
20	Spectral Width	18867.9
21	Lowest Frequency	-1136.3
22	Nucleus	13C
23	Acquired Size	16384
24	Spectral Size	16384
25	Digital Resolution	1.15

Parameters

	Parameter	Value
1	Title	PROTON_01
2	Comment	06BP-130-144
3	Origin	Varian
4	Instrument	mercury
5	Author	
6	Solvent	cdcl3
7	Temperature	25.0
8	Pulse Sequence	s2pul
9	Experiment	1D
10	Probe	Autosw
11	Number of Scans	16
12	Receiver Gain	39
13	Relaxation Delay	1.0000
14	Pulse Width	5.3500
15	Presaturation Frequency	
16	Acquisition Time	1.7064
17	Acquisition Date	2019-08-14T13:48:32
18	Modification Date	2019-08-14T13:50:28
19	Spectrometer Frequency	299.99
20	Spectral Width	4800.8
21	Lowest Frequency	-599.9
22	Nucleus	1H
23	Acquired Size	8192
24	Spectral Size	16384
25	Digital Resolution	0.29

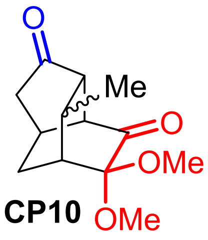

CP10

diastereomeric mixture

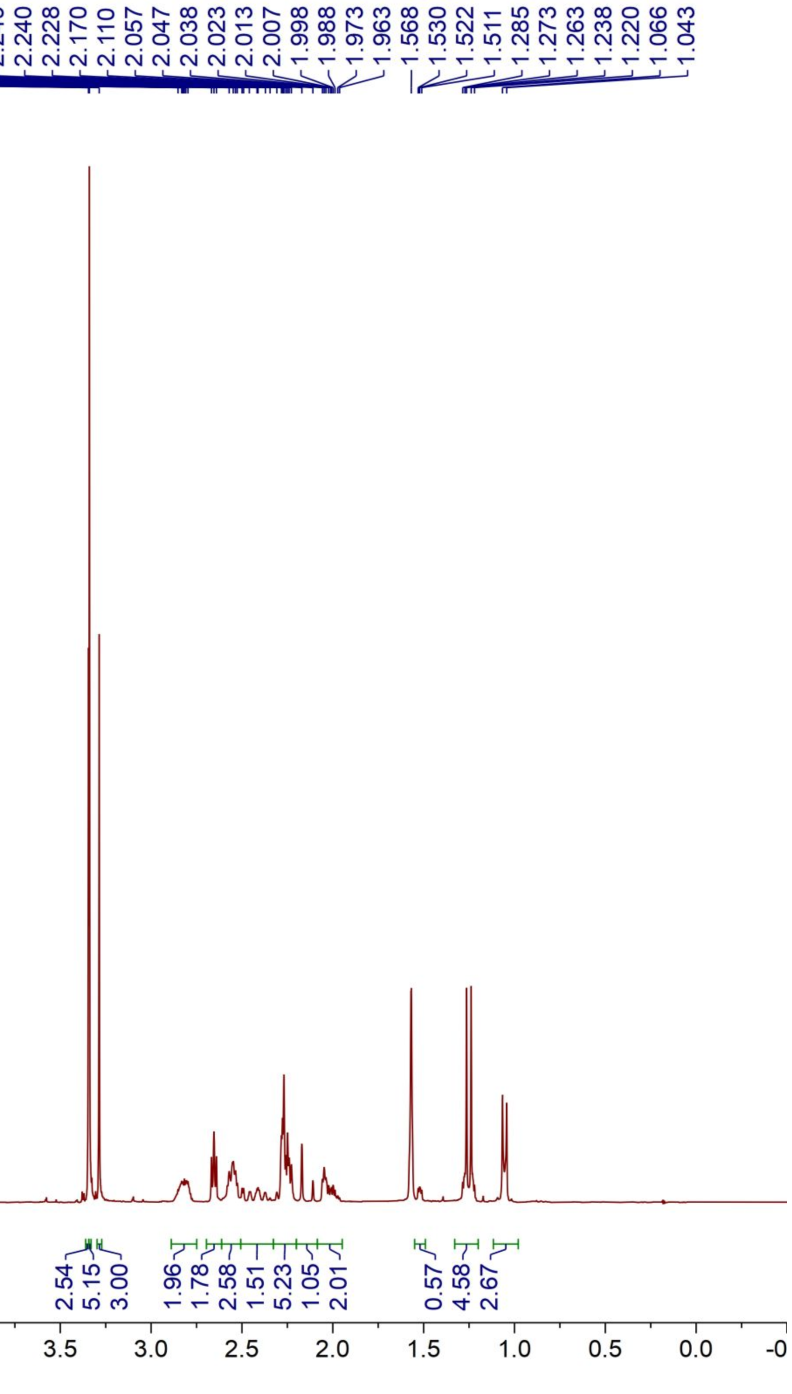

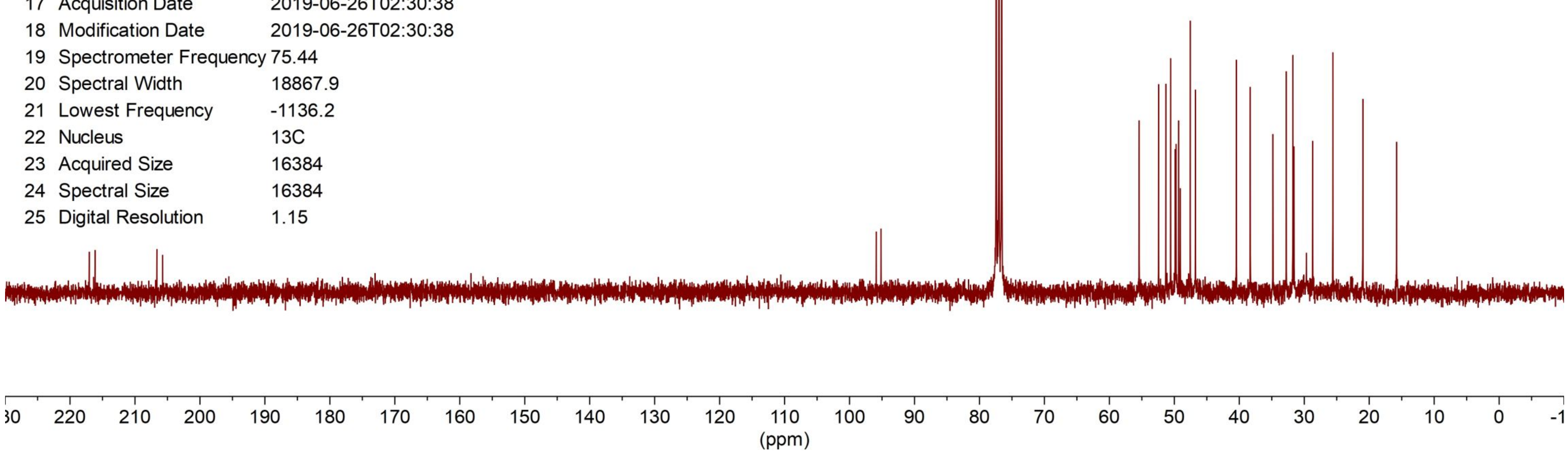

Parameters

	Parameter	Value
1	Title	CARBON_01
2	Comment	06BP-130-144-70
3	Origin	Varian
4	Instrument	mercury
5	Author	
6	Solvent	cdcl3
7	Temperature	25.0
8	Pulse Sequence	s2pul
9	Experiment	1D
10	Probe	Autosw
11	Number of Scans	16384
12	Receiver Gain	30
13	Relaxation Delay	1.0000
14	Pulse Width	7.3000
15	Presaturation Frequency	
16	Acquisition Time	0.8684
17	Acquisition Date	2019-06-26T02:30:38
18	Modification Date	2019-06-26T02:30:38
19	Spectrometer Frequency	75.44
20	Spectral Width	18867.9
21	Lowest Frequency	-1136.2
22	Nucleus	13C
23	Acquired Size	16384
24	Spectral Size	16384
25	Digital Resolution	1.15

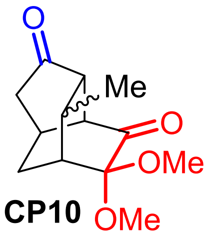

CP10

diastereomeric mixture

	Parameter	Value
1	Title	PROTON_01
2	Comment	07BP-068-158-B
3	Origin	Varian
4	Instrument	mercury
5	Author	
6	Solvent	cdcl3
7	Temperature	25.0
8	Pulse Sequence	s2pul
9	Experiment	1D
10	Probe	Autosw
11	Number of Scans	32
12	Receiver Gain	30
13	Relaxation Delay	1.0000
14	Pulse Width	5.3000
15	Presaturation Frequency	
16	Acquisition Time	1.7064
17	Acquisition Date	2019-01-12T18:49:36
18	Modification Date	2019-01-12T18:49:38
19	Spectrometer Frequency	299.99
20	Spectral Width	4800.8
21	Lowest Frequency	-599.8
22	Nucleus	1H
23	Acquired Size	8192
24	Spectral Size	16384
25	Digital Resolution	0.29

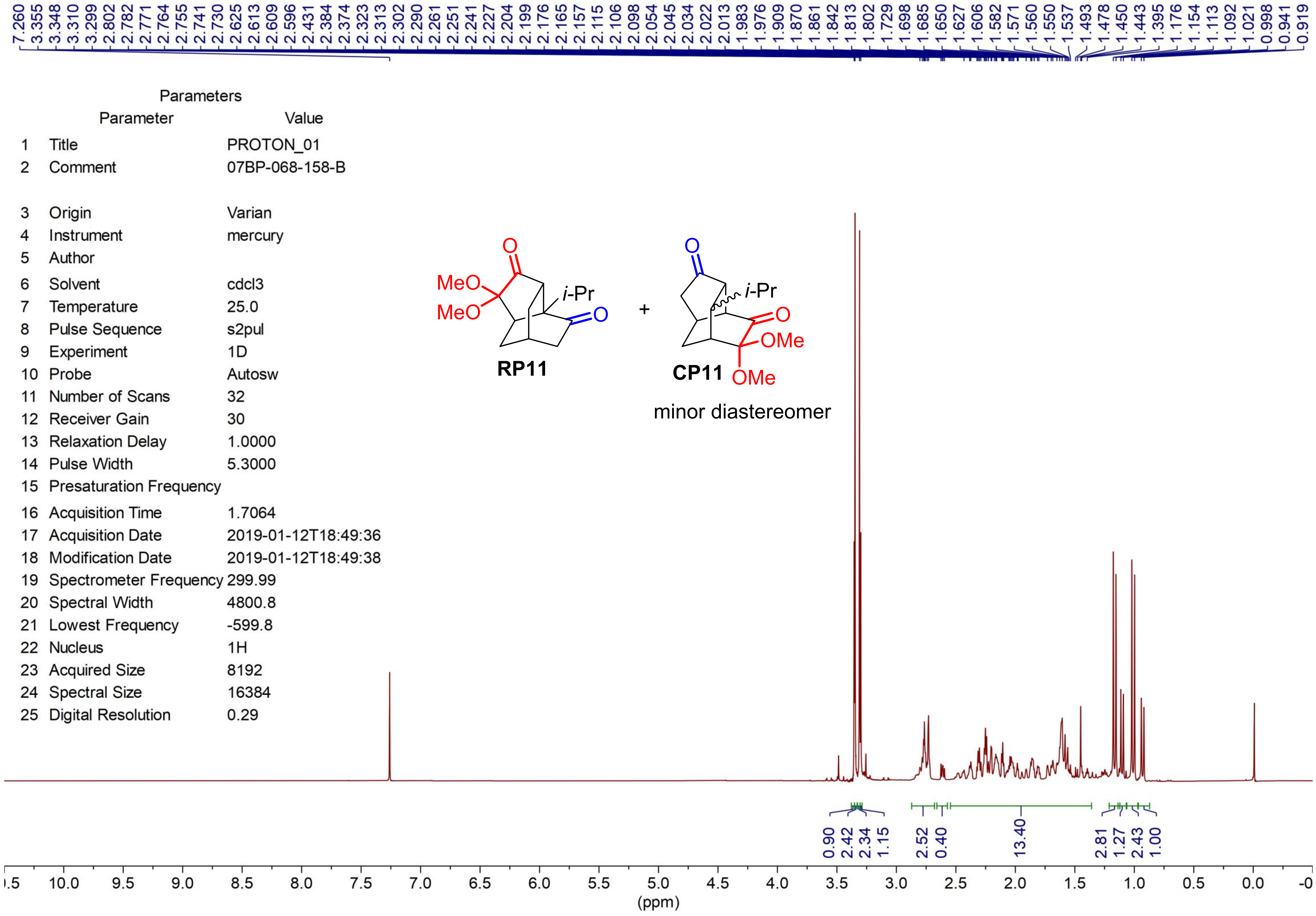

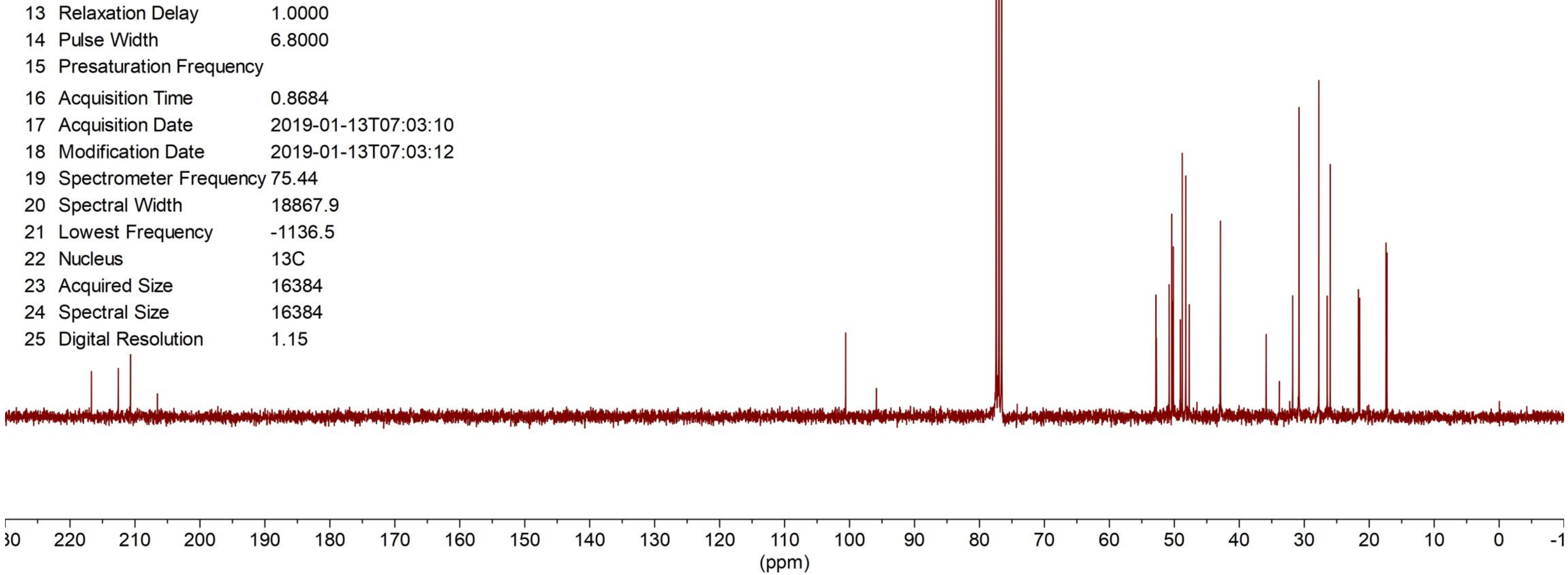

Parameters

	Parameter	Value
1	Title	CARBON_01
2	Comment	07BP-068-158-B
3	Origin	Varian
4	Instrument	mercury
5	Author	
6	Solvent	cdcl3
7	Temperature	25.0
8	Pulse Sequence	s2pul
9	Experiment	1D
10	Probe	Autosw
11	Number of Scans	10000
12	Receiver Gain	30
13	Relaxation Delay	1.0000
14	Pulse Width	6.8000
15	Presaturation Frequency	
16	Acquisition Time	0.8684
17	Acquisition Date	2019-01-13T07:03:10
18	Modification Date	2019-01-13T07:03:12
19	Spectrometer Frequency	75.44
20	Spectral Width	18867.9
21	Lowest Frequency	-1136.5
22	Nucleus	13C
23	Acquired Size	16384
24	Spectral Size	16384
25	Digital Resolution	1.15

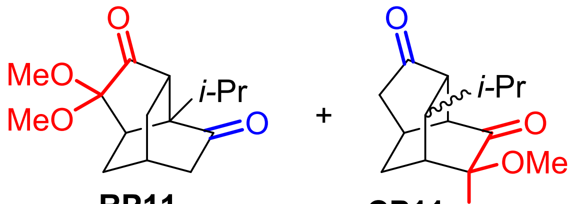

RP11 **CP11**

minor diastereomer

	Parameter	Value
1	Title	PROTON_01
2	Comment	07BP-068-158-54
3	Origin	Varian
4	Instrument	mercury
5	Author	
6	Solvent	cdcl3
7	Temperature	25.0
8	Pulse Sequence	s2pul
9	Experiment	1D
10	Probe	Autosw
11	Number of Scans	32
12	Receiver Gain	30
13	Relaxation Delay	1.0000
14	Pulse Width	5.3000
15	Presaturation Frequency	
16	Acquisition Time	1.7064
17	Acquisition Date	2018-12-06T18:02:29
18	Modification Date	2018-12-06T18:02:30
19	Spectrometer Frequency	299.99
20	Spectral Width	4800.8
21	Lowest Frequency	-599.8
22	Nucleus	1H
23	Acquired Size	8192
24	Spectral Size	16384
25	Digital Resolution	0.29

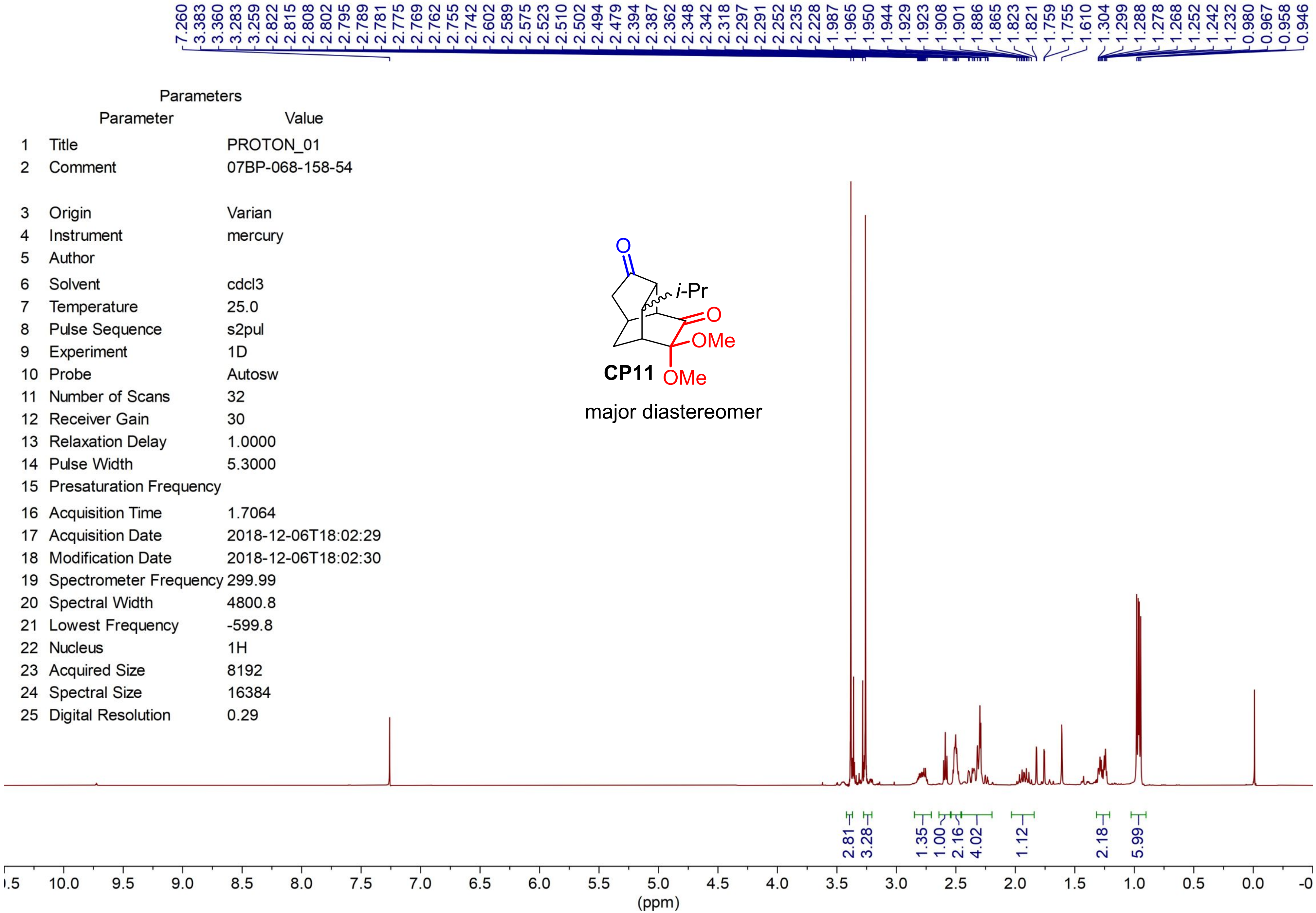

Parameters

	Parameter	Value
1	Title	CARBON_01
2	Comment	07BP-068-158-C_40
3	Origin	Varian
4	Instrument	mercury
5	Author	
6	Solvent	cdcl3
7	Temperature	25.0
8	Pulse Sequence	s2pul
9	Experiment	1D
10	Probe	Autosw
11	Number of Scans	10000
12	Receiver Gain	30
13	Relaxation Delay	1.0000
14	Pulse Width	6.8000
15	Presaturation Frequency	
16	Acquisition Time	0.8684
17	Acquisition Date	2019-01-06T06:19:16
18	Modification Date	2019-01-06T06:19:18
19	Spectrometer Frequency	75.44
20	Spectral Width	18867.9
21	Lowest Frequency	-1137.0
22	Nucleus	13C
23	Acquired Size	16384
24	Spectral Size	16384
25	Digital Resolution	1.15

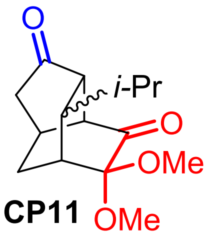

CP11

major diastereomer

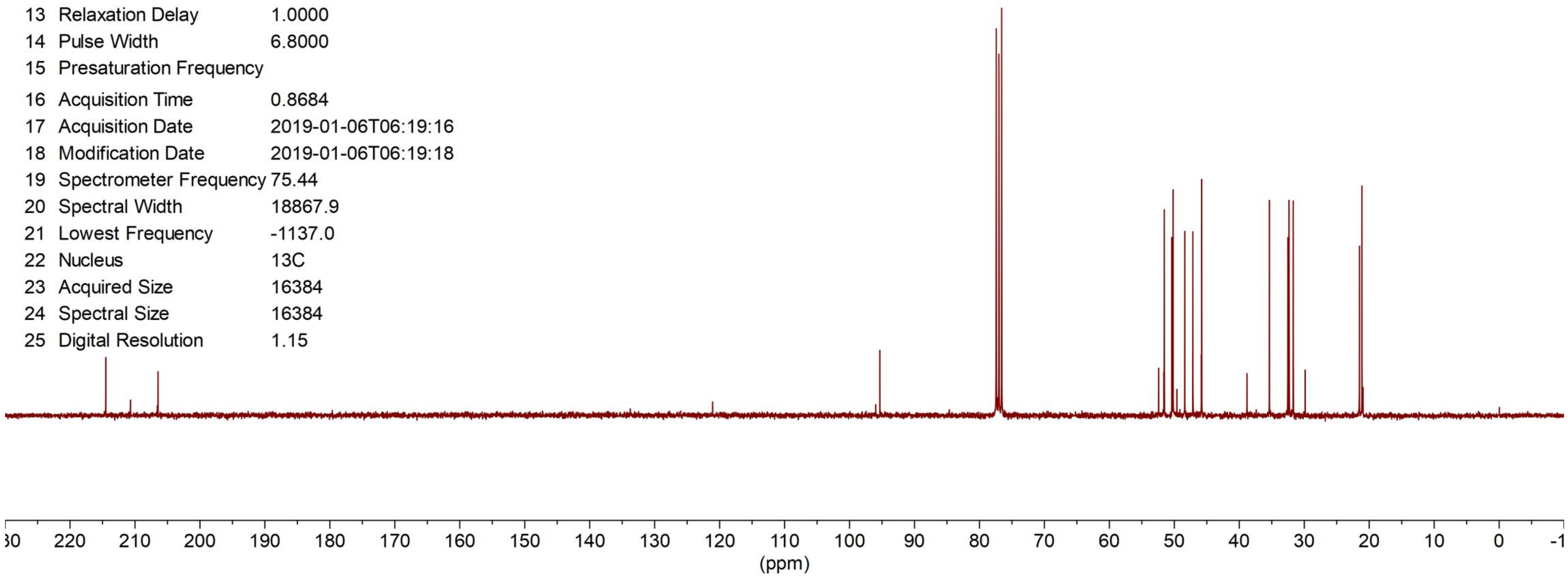

Parameters

	Parameter	Value
1	Title	PROTON_01
2	Comment	07BP-113-088-43-B
3	Origin	Varian
4	Instrument	mercury
5	Author	
6	Solvent	cdcl3
7	Temperature	25.0
8	Pulse Sequence	s2pul
9	Experiment	1D
10	Probe	Autosw
11	Number of Scans	16
12	Receiver Gain	26
13	Relaxation Delay	1.0000
14	Pulse Width	5.3500
15	Presaturation Frequency	
16	Acquisition Time	1.7064
17	Acquisition Date	2019-07-07T18:48:24
18	Modification Date	2019-07-07T18:48:24
19	Spectrometer Frequency	299.99
20	Spectral Width	4800.8
21	Lowest Frequency	-599.8
22	Nucleus	1H
23	Acquired Size	8192
24	Spectral Size	16384
25	Digital Resolution	0.29

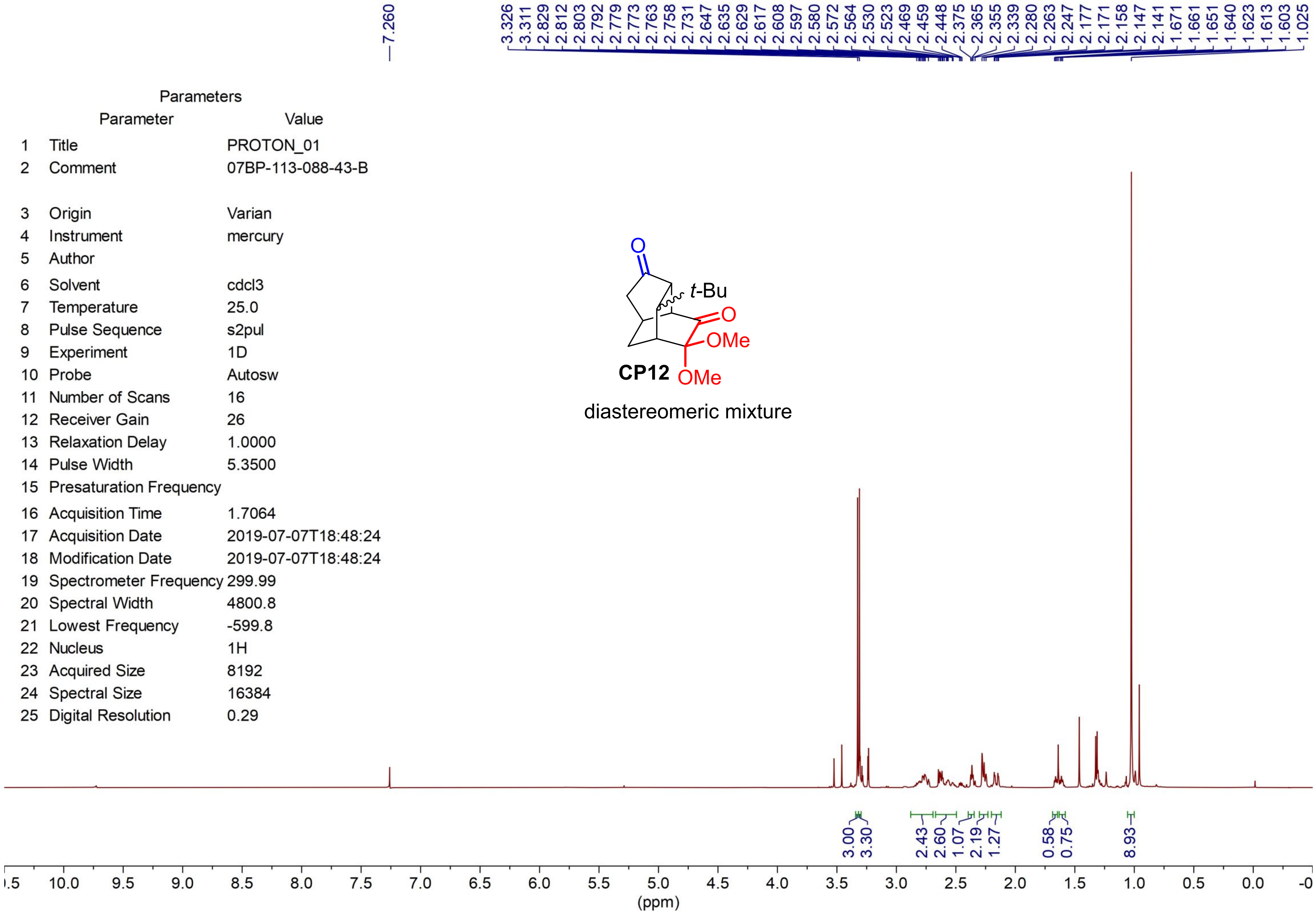

Parameters

	Parameter	Value
1	Title	CARBON_01
2	Comment	07BP-113-088-43-B
3	Origin	Varian
4	Instrument	mercury
5	Author	
6	Solvent	cdcl3
7	Temperature	25.0
8	Pulse Sequence	s2pul
9	Experiment	1D
10	Probe	Autosw
11	Number of Scans	10000
12	Receiver Gain	30
13	Relaxation Delay	1.0000
14	Pulse Width	7.3000
15	Presaturation Frequency	
16	Acquisition Time	0.8684
17	Acquisition Date	2019-07-08T00:03:11
18	Modification Date	2019-07-08T00:03:12
19	Spectrometer Frequency	75.44
20	Spectral Width	18867.9
21	Lowest Frequency	-1136.7
22	Nucleus	13C
23	Acquired Size	16384
24	Spectral Size	16384
25	Digital Resolution	1.15

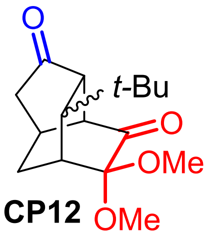

CP12

diastereomeric mixture

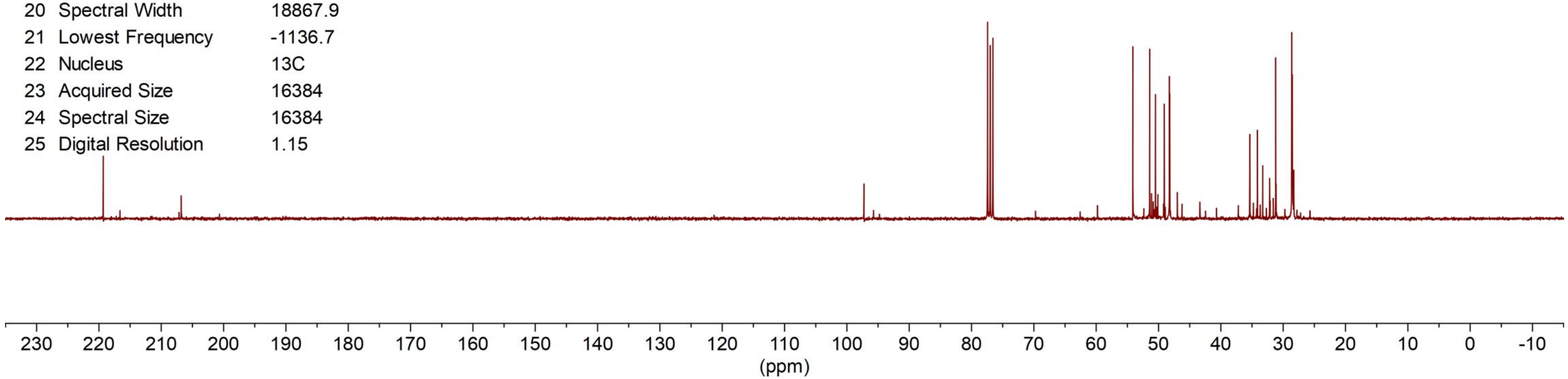

Parameters

	Parameter	Value
1	Title	PROTON_01
2	Comment	07BP-113-130-A
3	Origin	Varian
4	Instrument	mercury
5	Author	
6	Solvent	cdcl3
7	Temperature	25.0
8	Pulse Sequence	s2pul
9	Experiment	1D
10	Probe	Autosw
11	Number of Scans	16
12	Receiver Gain	39
13	Relaxation Delay	1.0000
14	Pulse Width	5.3000
15	Presaturation Frequency	
16	Acquisition Time	1.7064
17	Acquisition Date	2019-04-26T16:13:33
18	Modification Date	2019-04-26T16:13:34
19	Spectrometer Frequency	299.99
20	Spectral Width	4800.8
21	Lowest Frequency	-599.9
22	Nucleus	1H
23	Acquired Size	8192
24	Spectral Size	16384
25	Digital Resolution	0.29

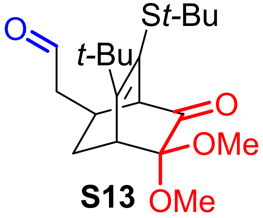

S13

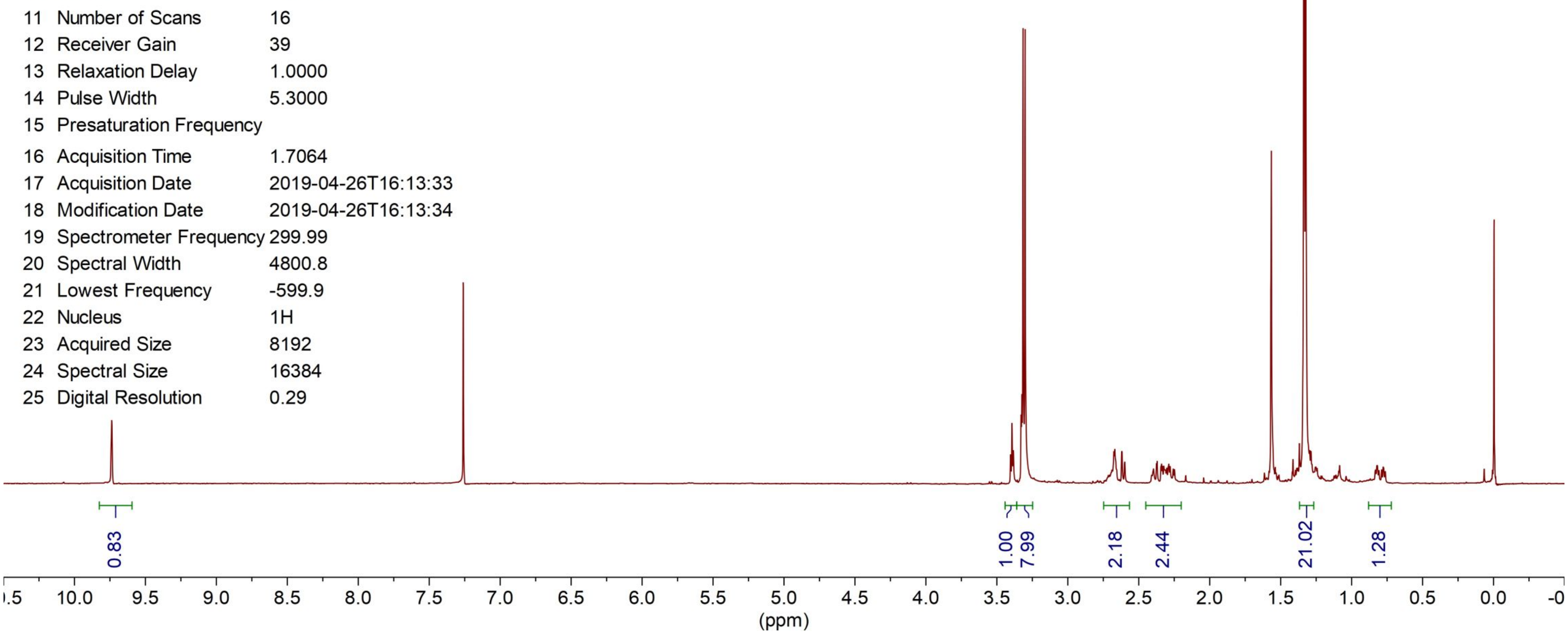

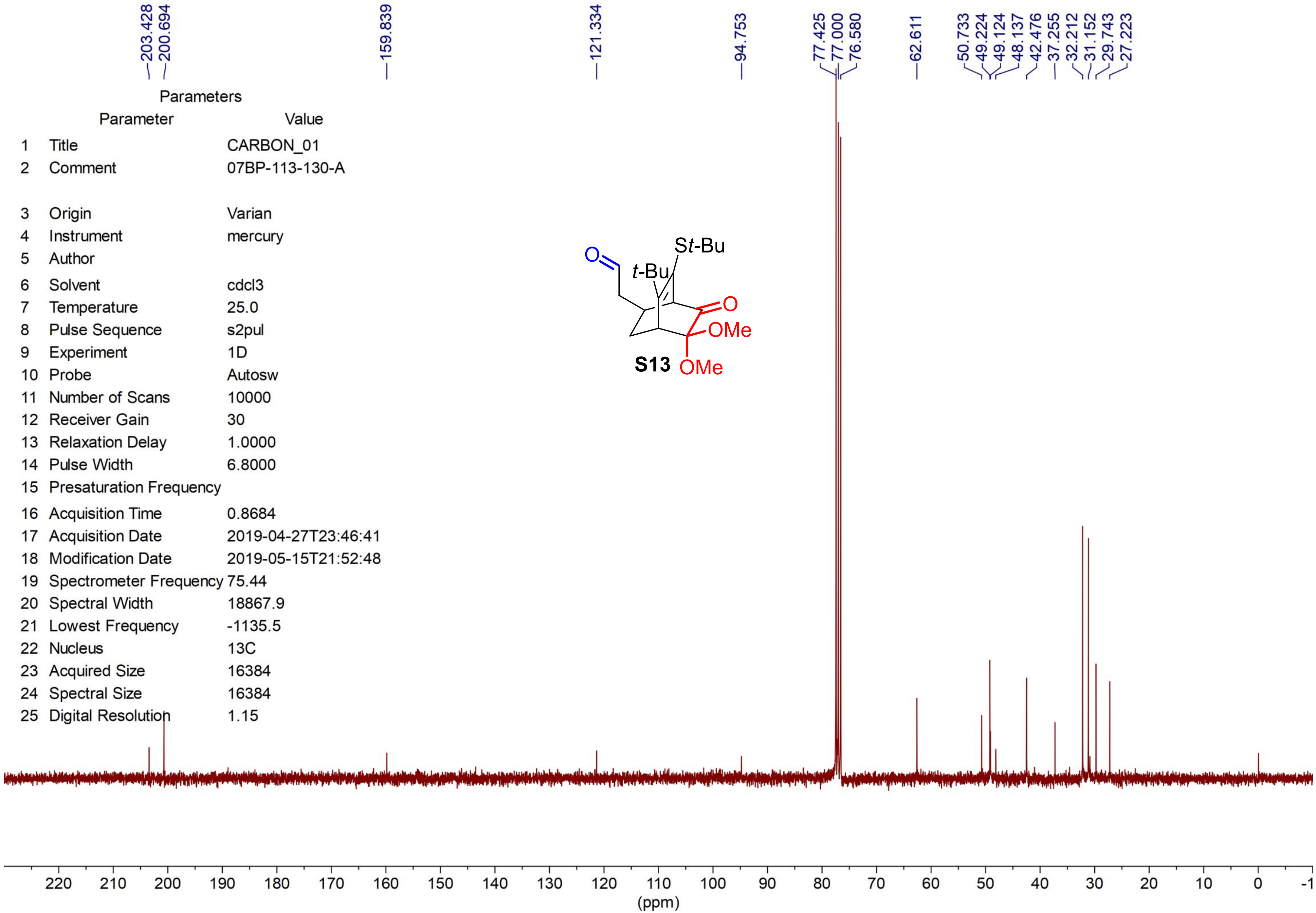

Parameters

	Parameter	Value
1	Title	CARBON_01
2	Comment	07BP-113-130-A
3	Origin	Varian
4	Instrument	mercury
5	Author	
6	Solvent	cdcl3
7	Temperature	25.0
8	Pulse Sequence	s2pul
9	Experiment	1D
10	Probe	Autosw
11	Number of Scans	10000
12	Receiver Gain	30
13	Relaxation Delay	1.0000
14	Pulse Width	6.8000
15	Presaturation Frequency	
16	Acquisition Time	0.8684
17	Acquisition Date	2019-04-27T23:46:41
18	Modification Date	2019-05-15T21:52:48
19	Spectrometer Frequency	75.44
20	Spectral Width	18867.9
21	Lowest Frequency	-1135.5
22	Nucleus	13C
23	Acquired Size	16384
24	Spectral Size	16384
25	Digital Resolution	1.15

S13

Parameters

	Parameter	Value
1	Title	PROTON_01
2	Comment	06BP-130-132-B
3	Origin	Varian
4	Instrument	mercury
5	Author	
6	Solvent	cdcl3
7	Temperature	25.0
8	Pulse Sequence	s2pul
9	Experiment	1D
10	Probe	Autosw
11	Number of Scans	16
12	Receiver Gain	39
13	Relaxation Delay	1.0000
14	Pulse Width	5.3500
15	Presaturation Frequency	
16	Acquisition Time	1.7064
17	Acquisition Date	2019-06-15T16:24:00
18	Modification Date	2019-06-15T16:24:00
19	Spectrometer Frequency	299.99
20	Spectral Width	4800.8
21	Lowest Frequency	-600.0
22	Nucleus	1H
23	Acquired Size	8192
24	Spectral Size	16384
25	Digital Resolution	0.29

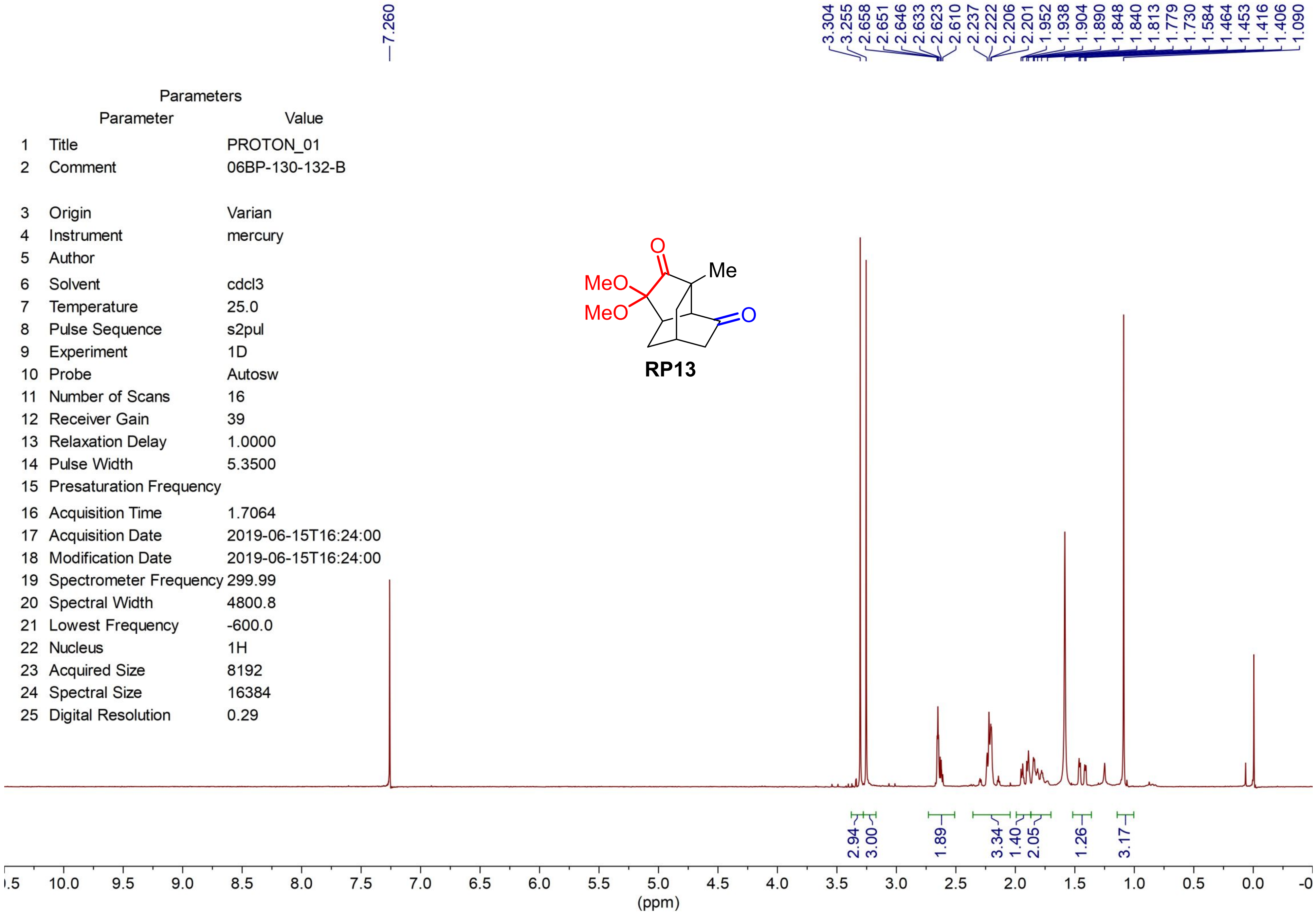

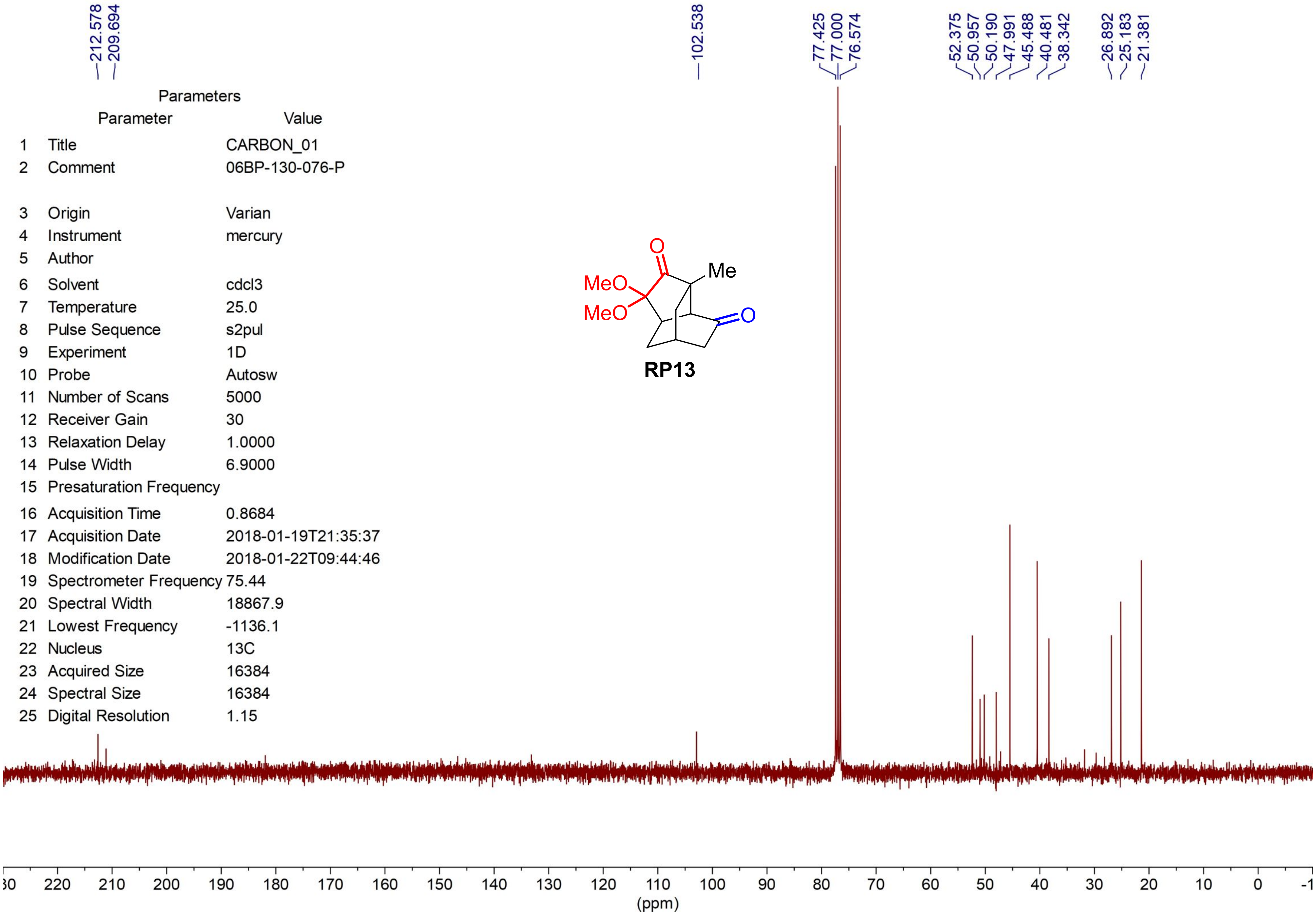

Parameters

	Parameter	Value
1	Title	CARBON_01
2	Comment	06BP-130-076-P
3	Origin	Varian
4	Instrument	mercury
5	Author	
6	Solvent	cdcl3
7	Temperature	25.0
8	Pulse Sequence	s2pul
9	Experiment	1D
10	Probe	Autosw
11	Number of Scans	5000
12	Receiver Gain	30
13	Relaxation Delay	1.0000
14	Pulse Width	6.9000
15	Presaturation Frequency	
16	Acquisition Time	0.8684
17	Acquisition Date	2018-01-19T21:35:37
18	Modification Date	2018-01-22T09:44:46
19	Spectrometer Frequency	75.44
20	Spectral Width	18867.9
21	Lowest Frequency	-1136.1
22	Nucleus	13C
23	Acquired Size	16384
24	Spectral Size	16384
25	Digital Resolution	1.15

RP13

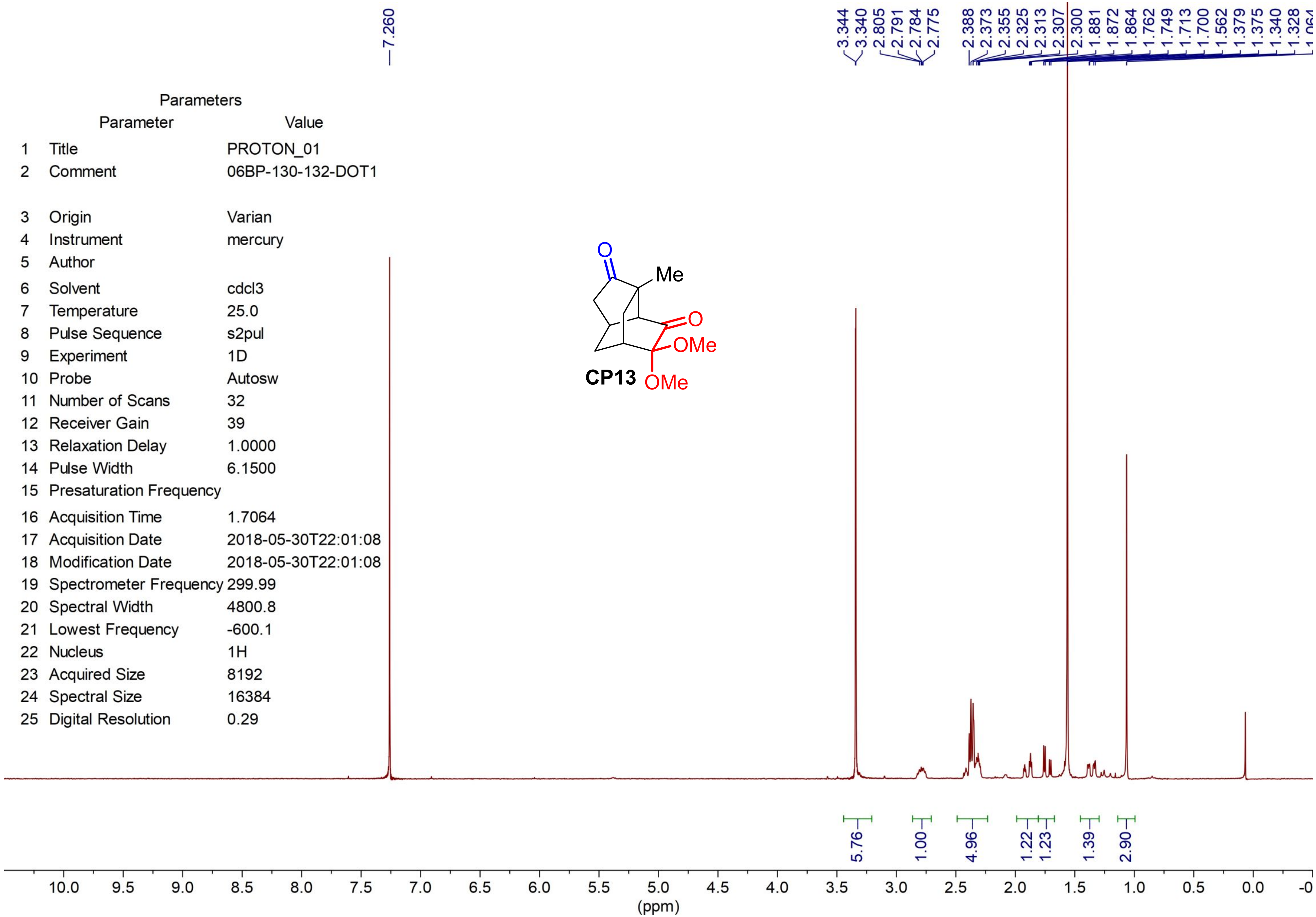

Parameters

	Parameter	Value
1	Title	PROTON_01
2	Comment	06BP-130-132-DOT1
3	Origin	Varian
4	Instrument	mercury
5	Author	
6	Solvent	cdcl3
7	Temperature	25.0
8	Pulse Sequence	s2pul
9	Experiment	1D
10	Probe	Autosw
11	Number of Scans	32
12	Receiver Gain	39
13	Relaxation Delay	1.0000
14	Pulse Width	6.1500
15	Presaturation Frequency	
16	Acquisition Time	1.7064
17	Acquisition Date	2018-05-30T22:01:08
18	Modification Date	2018-05-30T22:01:08
19	Spectrometer Frequency	299.99
20	Spectral Width	4800.8
21	Lowest Frequency	-600.1
22	Nucleus	1H
23	Acquired Size	8192
24	Spectral Size	16384
25	Digital Resolution	0.29

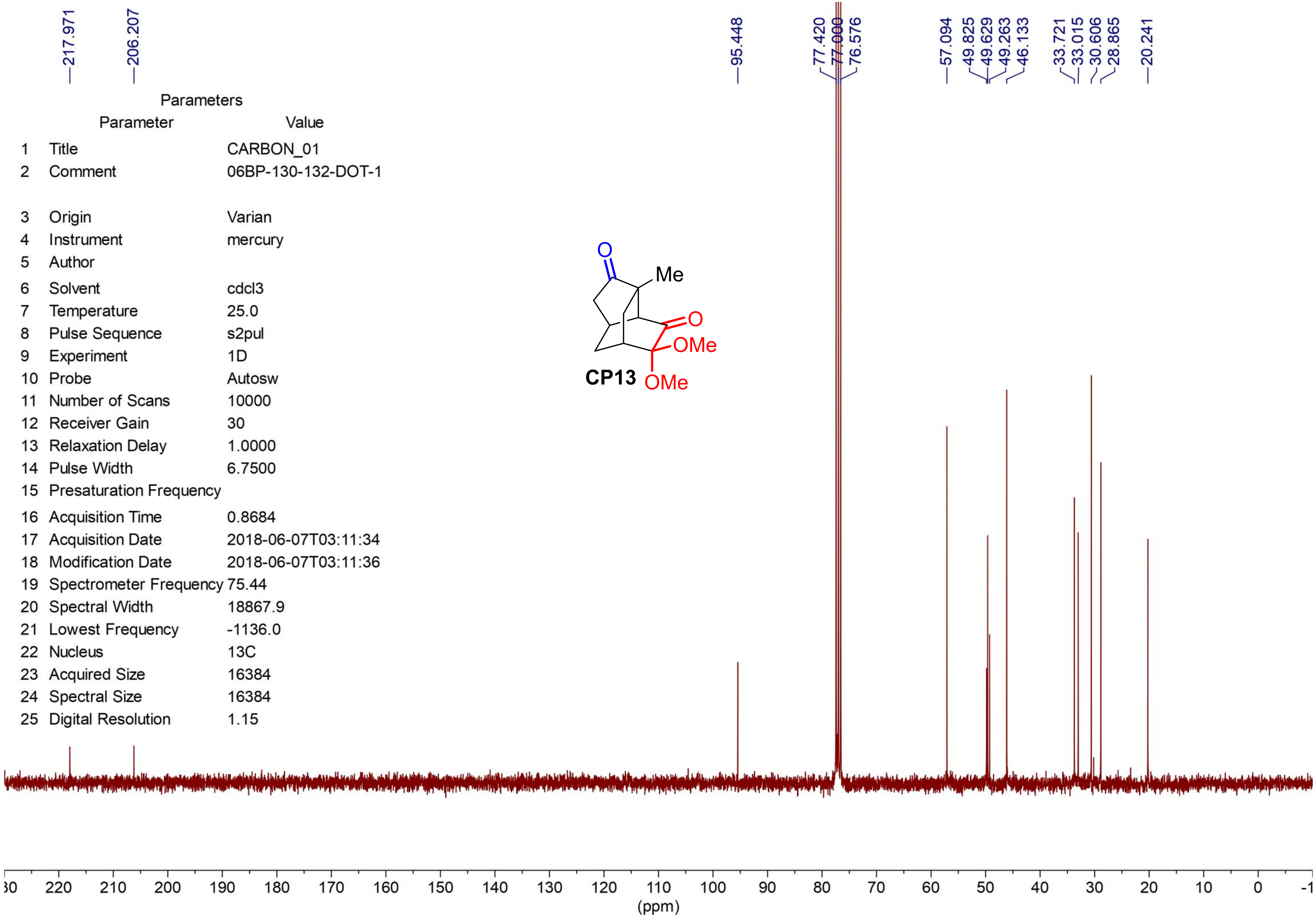

CP13

Parameters

	Parameter	Value
1	Title	CARBON_01
2	Comment	06BP-130-132-DOT-1
3	Origin	Varian
4	Instrument	mercury
5	Author	
6	Solvent	cdcl3
7	Temperature	25.0
8	Pulse Sequence	s2pul
9	Experiment	1D
10	Probe	Autosw
11	Number of Scans	10000
12	Receiver Gain	30
13	Relaxation Delay	1.0000
14	Pulse Width	6.7500
15	Presaturation Frequency	
16	Acquisition Time	0.8684
17	Acquisition Date	2018-06-07T03:11:34
18	Modification Date	2018-06-07T03:11:36
19	Spectrometer Frequency	75.44
20	Spectral Width	18867.9
21	Lowest Frequency	-1136.0
22	Nucleus	13C
23	Acquired Size	16384
24	Spectral Size	16384
25	Digital Resolution	1.15

Parameters

	Parameter	Value
1	Title	PROTON_01
2	Comment	07BP-068-178-B
3	Origin	Varian
4	Instrument	mercury
5	Author	
6	Solvent	cdcl3
7	Temperature	25.0
8	Pulse Sequence	s2pul
9	Experiment	1D
10	Probe	Autosw
11	Number of Scans	16
12	Receiver Gain	30
13	Relaxation Delay	1.0000
14	Pulse Width	5.3000
15	Presaturation Frequency	
16	Acquisition Time	1.7064
17	Acquisition Date	2018-12-29T15:58:03
18	Modification Date	2018-12-29T15:58:04
19	Spectrometer Frequency	299.99
20	Spectral Width	4800.8
21	Lowest Frequency	-599.9
22	Nucleus	1H
23	Acquired Size	8192
24	Spectral Size	16384
25	Digital Resolution	0.29

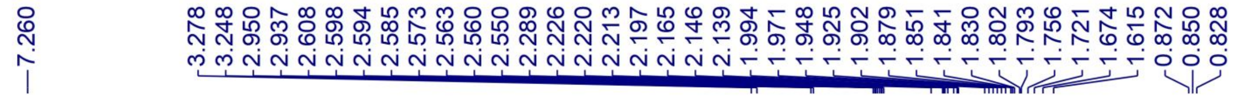

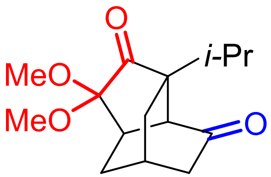

RP14

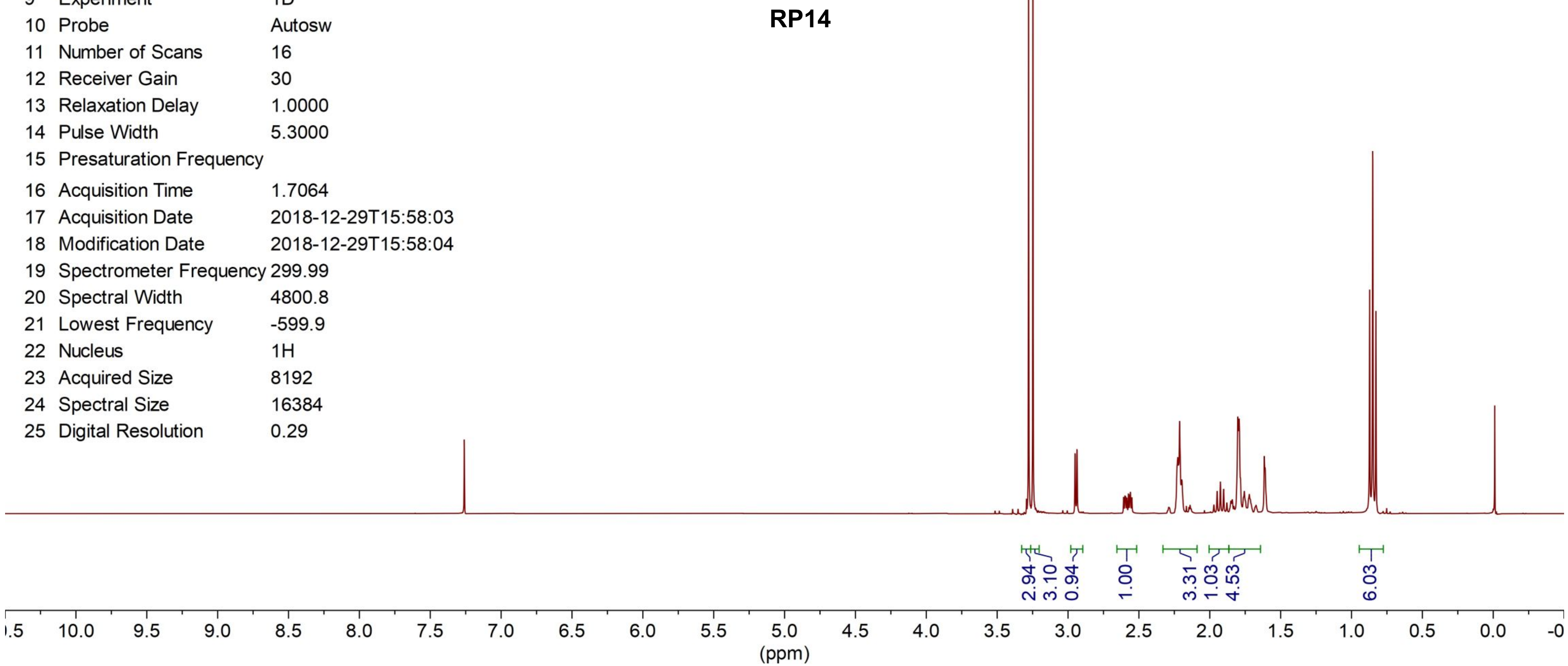

Parameters

	Parameter	Value
1	Title	CARBON_01
2	Comment	07BP-068-178-B
3	Origin	Varian
4	Instrument	mercury
5	Author	
6	Solvent	cdcl3
7	Temperature	25.0
8	Pulse Sequence	s2pul
9	Experiment	1D
10	Probe	Autosw
11	Number of Scans	10000
12	Receiver Gain	30
13	Relaxation Delay	1.0000
14	Pulse Width	6.8000
15	Presaturation Frequency	
16	Acquisition Time	0.8684
17	Acquisition Date	2018-12-30T04:11:04
18	Modification Date	2018-12-30T04:11:06
19	Spectrometer Frequency	75.44
20	Spectral Width	18867.9
21	Lowest Frequency	-1136.9
22	Nucleus	13C
23	Acquired Size	16384
24	Spectral Size	16384
25	Digital Resolution	1.15

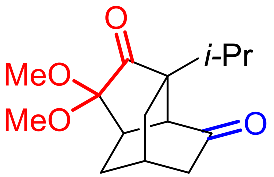

RP14

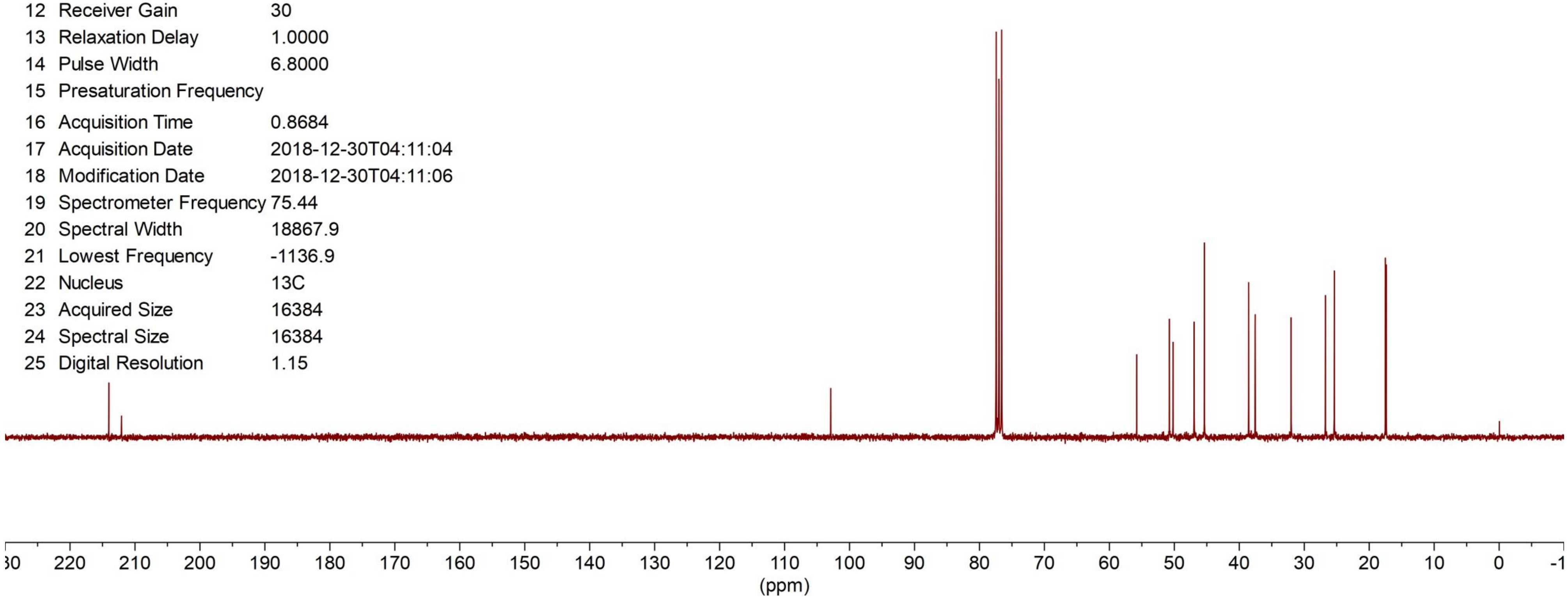

Parameters		
	Parameter	Value
1	Title	PROTON_01
2	Comment	07BP-068-178-A
3	Origin	Varian
4	Instrument	mercury
5	Author	
6	Solvent	cdcl3
7	Temperature	25.0
8	Pulse Sequence	s2pul
9	Experiment	1D
10	Probe	autosw
11	Number of Scans	32
12	Receiver Gain	39
13	Relaxation Delay	1.0000
14	Pulse Width	6.8500
15	Presaturation Frequency	
16	Acquisition Time	2.5608
17	Acquisition Date	2018-12-15T18:06:03
18	Modification Date	2018-12-15T18:06:04
19	Spectrometer Frequency	399.93
20	Spectral Width	6398.0
21	Lowest Frequency	-798.9
22	Nucleus	1H
23	Acquired Size	16384
24	Spectral Size	65536
25	Digital Resolution	0.10

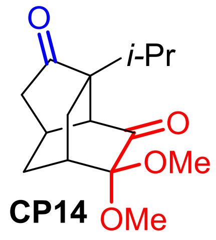

CP14

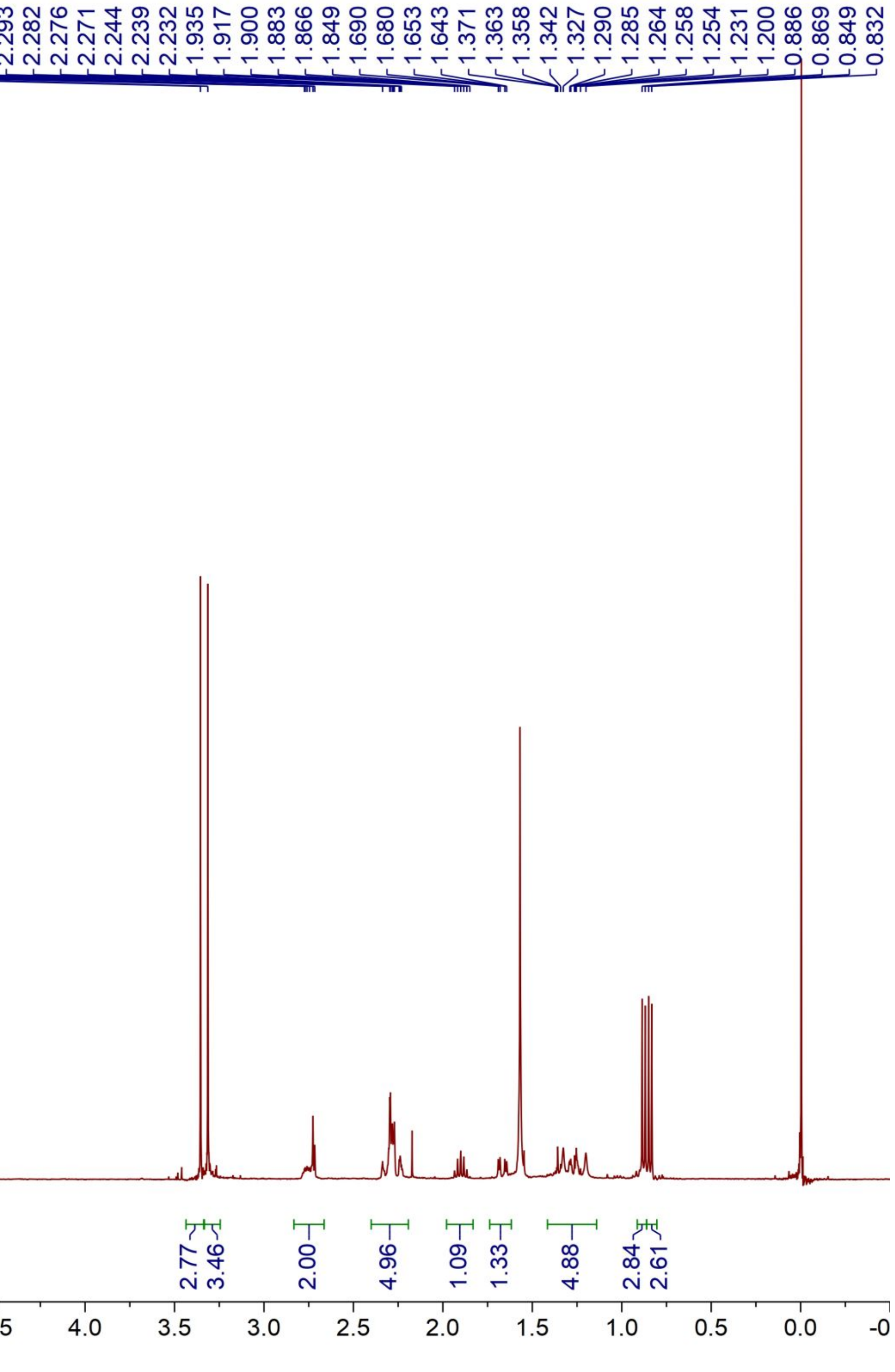

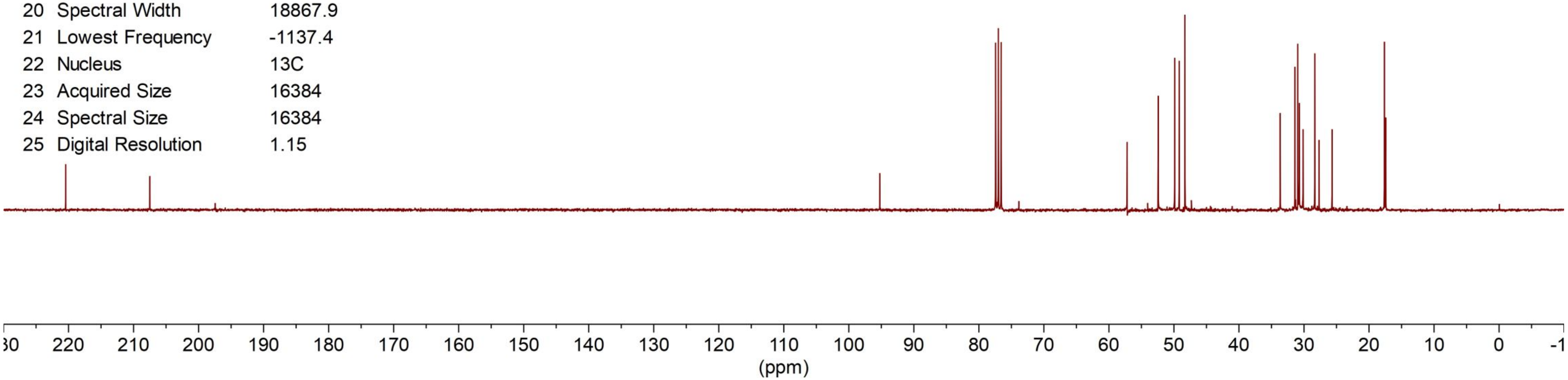

Parameters

	Parameter	Value
1	Title	CARBON_01
2	Comment	07BP-068-178-A
3	Origin	Varian
4	Instrument	mercury
5	Author	
6	Solvent	cdcl3
7	Temperature	25.0
8	Pulse Sequence	s2pul
9	Experiment	1D
10	Probe	Autosw
11	Number of Scans	10000
12	Receiver Gain	30
13	Relaxation Delay	1.0000
14	Pulse Width	6.8000
15	Presaturation Frequency	
16	Acquisition Time	0.8684
17	Acquisition Date	2018-12-16T04:41:57
18	Modification Date	2018-12-16T04:41:58
19	Spectrometer Frequency	75.44
20	Spectral Width	18867.9
21	Lowest Frequency	-1137.4
22	Nucleus	13C
23	Acquired Size	16384
24	Spectral Size	16384
25	Digital Resolution	1.15

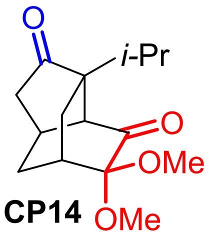

CP14

Parameters

	Parameter	Value
1	Title	PROTON_01
2	Comment	07BP-113-102-C
3	Origin	Varian
4	Instrument	mercury
5	Author	
6	Solvent	cdcl3
7	Temperature	25.0
8	Pulse Sequence	s2pul
9	Experiment	1D
10	Probe	Autosw
11	Number of Scans	16
12	Receiver Gain	39
13	Relaxation Delay	1.0000
14	Pulse Width	5.3000
15	Presaturation Frequency	
16	Acquisition Time	1.7064
17	Acquisition Date	2019-04-02T11:22:52
18	Modification Date	2019-04-02T11:22:52
19	Spectrometer Frequency	299.99
20	Spectral Width	4800.8
21	Lowest Frequency	-599.8
22	Nucleus	1H
23	Acquired Size	8192
24	Spectral Size	16384
25	Digital Resolution	0.29

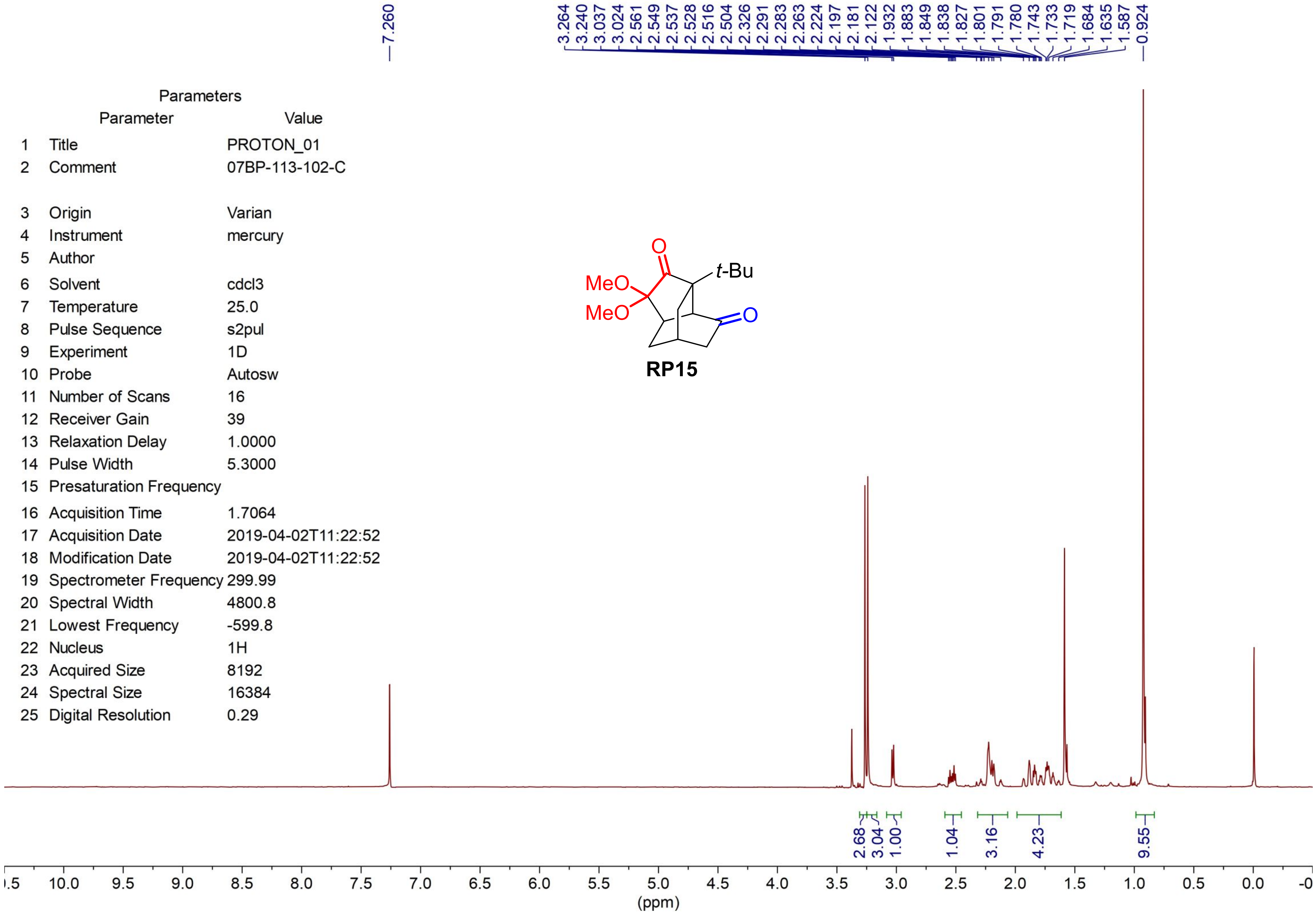

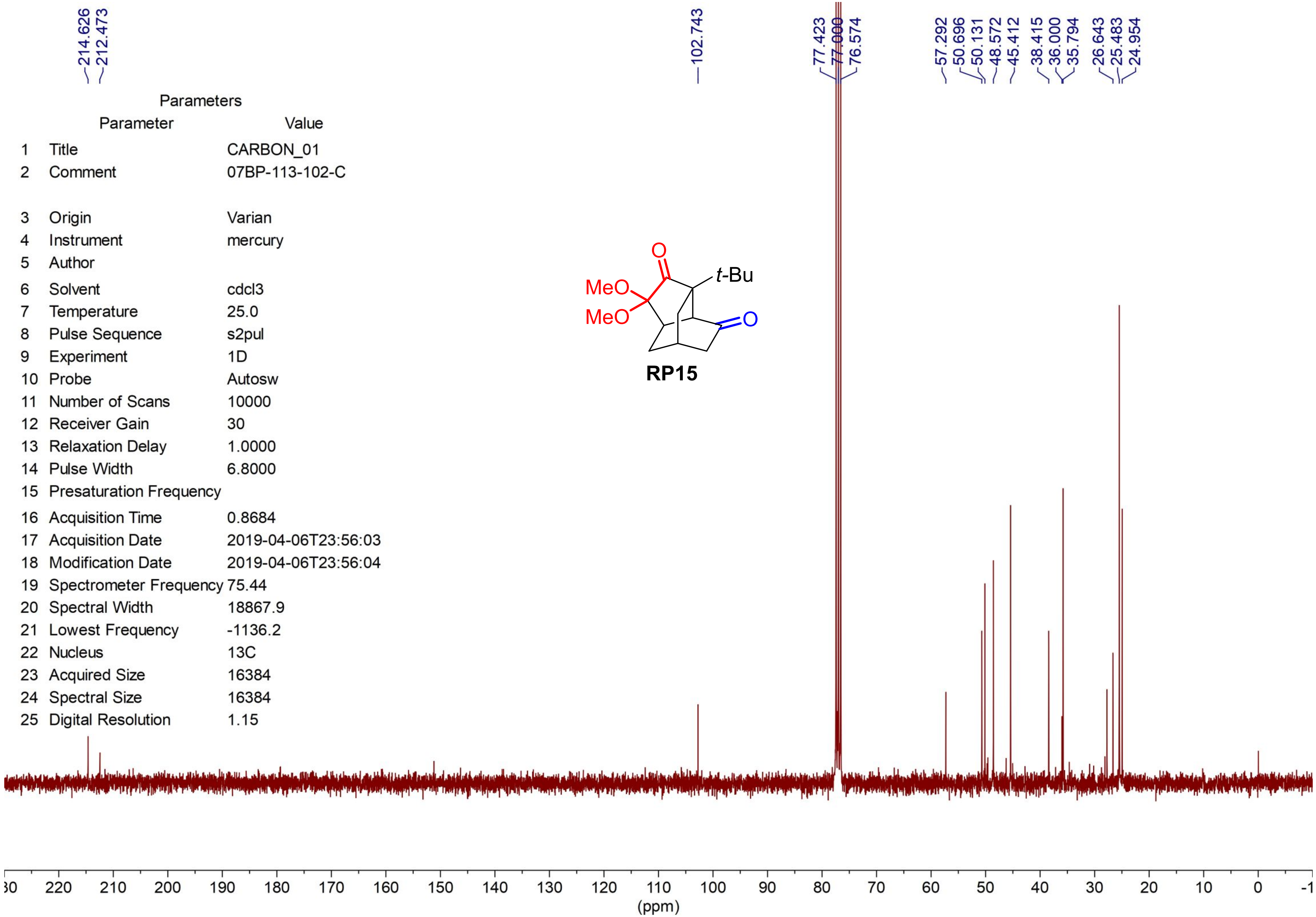

Parameters

	Parameter	Value
1	Title	CARBON_01
2	Comment	07BP-113-102-C
3	Origin	Varian
4	Instrument	mercury
5	Author	
6	Solvent	cdcl3
7	Temperature	25.0
8	Pulse Sequence	s2pul
9	Experiment	1D
10	Probe	Autosw
11	Number of Scans	10000
12	Receiver Gain	30
13	Relaxation Delay	1.0000
14	Pulse Width	6.8000
15	Presaturation Frequency	
16	Acquisition Time	0.8684
17	Acquisition Date	2019-04-06T23:56:03
18	Modification Date	2019-04-06T23:56:04
19	Spectrometer Frequency	75.44
20	Spectral Width	18867.9
21	Lowest Frequency	-1136.2
22	Nucleus	13C
23	Acquired Size	16384
24	Spectral Size	16384
25	Digital Resolution	1.15

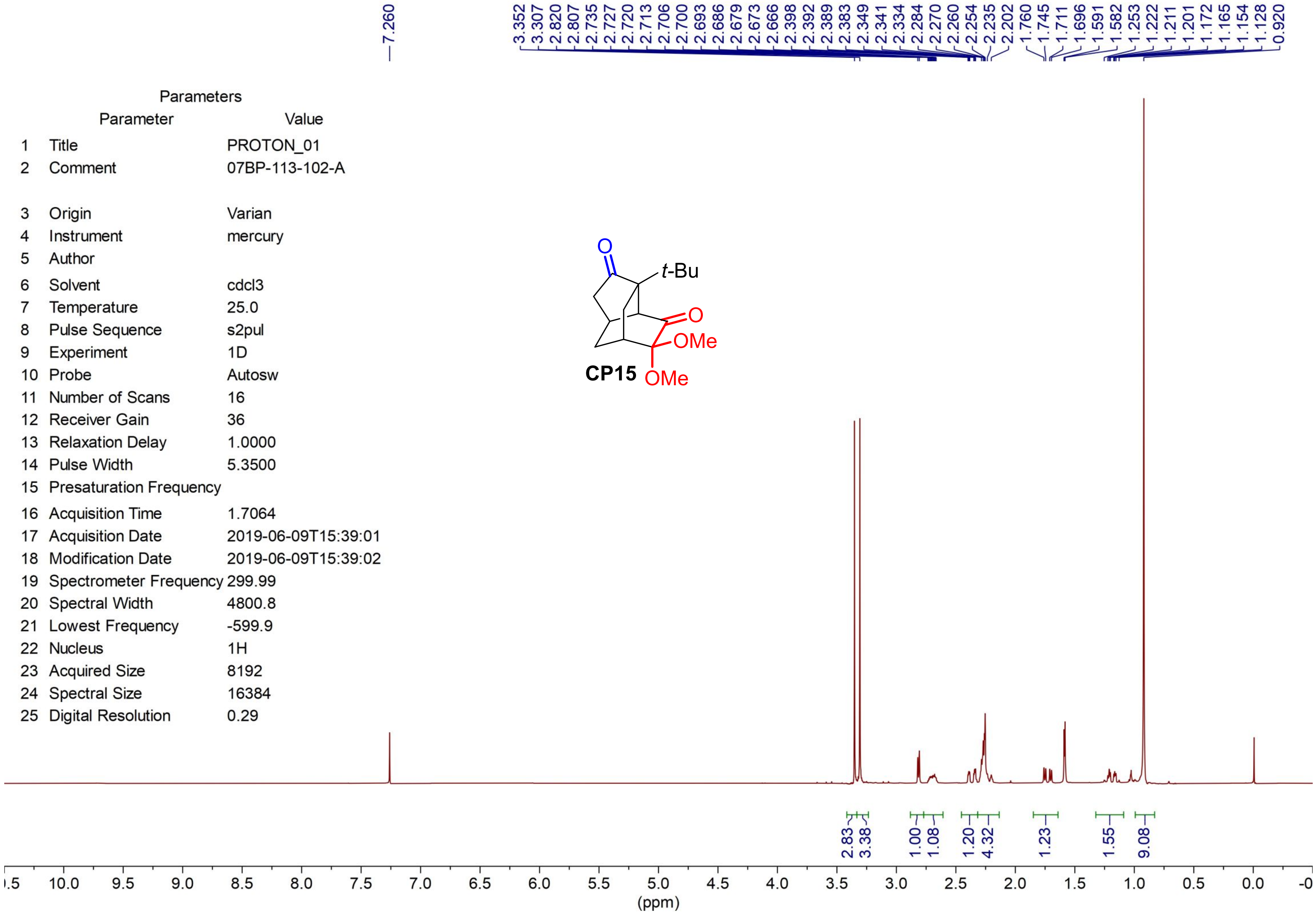

Parameters

	Parameter	Value
1	Title	PROTON_01
2	Comment	07BP-113-102-A
3	Origin	Varian
4	Instrument	mercury
5	Author	
6	Solvent	cdcl3
7	Temperature	25.0
8	Pulse Sequence	s2pul
9	Experiment	1D
10	Probe	Autosw
11	Number of Scans	16
12	Receiver Gain	36
13	Relaxation Delay	1.0000
14	Pulse Width	5.3500
15	Presaturation Frequency	
16	Acquisition Time	1.7064
17	Acquisition Date	2019-06-09T15:39:01
18	Modification Date	2019-06-09T15:39:02
19	Spectrometer Frequency	299.99
20	Spectral Width	4800.8
21	Lowest Frequency	-599.9
22	Nucleus	1H
23	Acquired Size	8192
24	Spectral Size	16384
25	Digital Resolution	0.29

CP15

Parameters

	Parameter	Value
1	Title	CARBON_01
2	Comment	07BP-113-102-A
3	Origin	Varian
4	Instrument	mercury
5	Author	
6	Solvent	cdcl3
7	Temperature	25.0
8	Pulse Sequence	s2pul
9	Experiment	1D
10	Probe	Autosw
11	Number of Scans	16384
12	Receiver Gain	30
13	Relaxation Delay	1.0000
14	Pulse Width	7.3000
15	Presaturation Frequency	
16	Acquisition Time	0.8684
17	Acquisition Date	2019-06-10T00:15:55
18	Modification Date	2019-06-10T00:15:56
19	Spectrometer Frequency	75.44
20	Spectral Width	18867.9
21	Lowest Frequency	-1135.6
22	Nucleus	13C
23	Acquired Size	16384
24	Spectral Size	16384
25	Digital Resolution	1.15

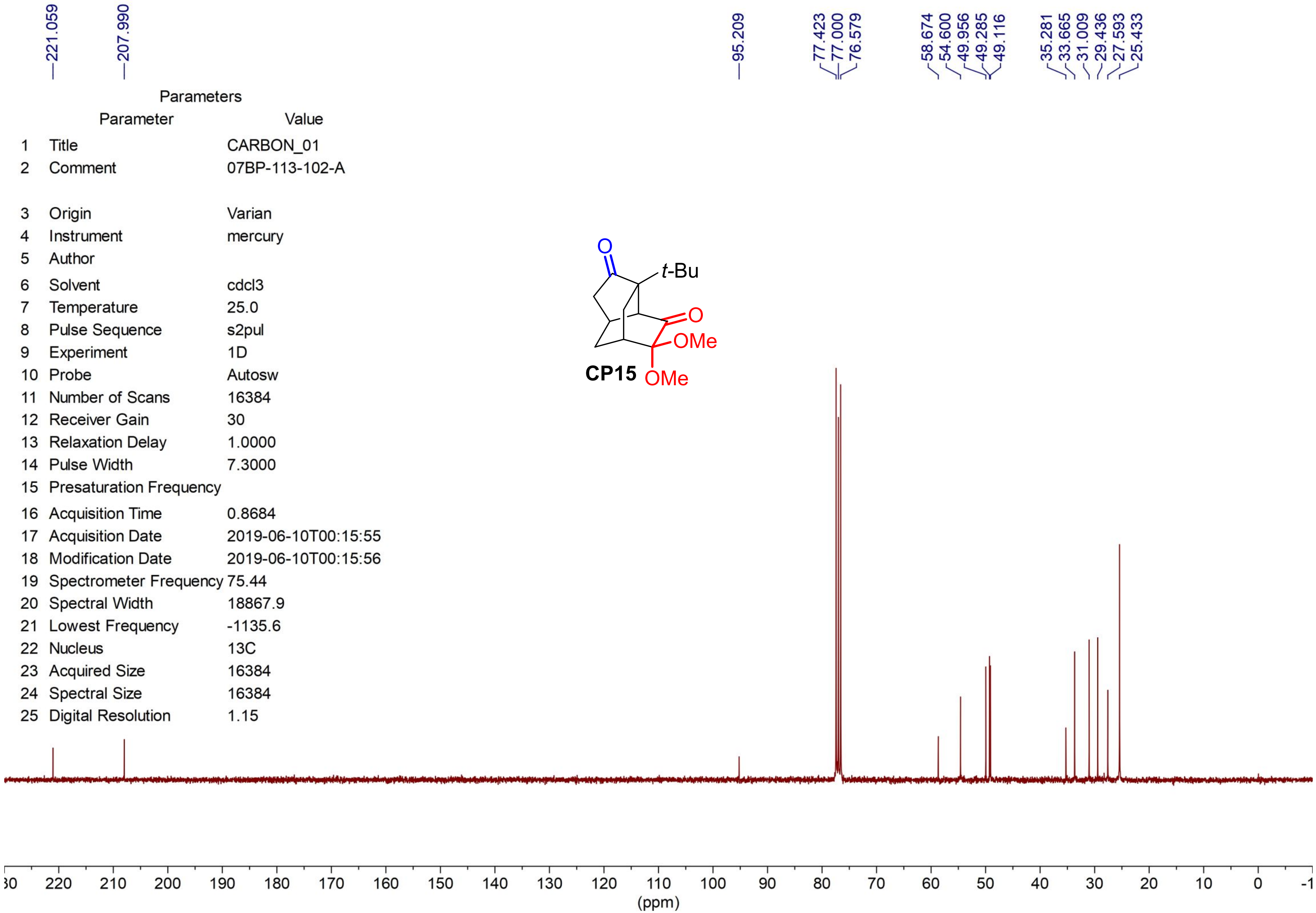

	Parameter	Value
1	Title	PROTON_01
2	Comment	06BP-130-162-B
3	Origin	Varian
4	Instrument	mercury
5	Author	
6	Solvent	cdcl3
7	Temperature	25.0
8	Pulse Sequence	s2pul
9	Experiment	1D
10	Probe	Autosw
11	Number of Scans	16
12	Receiver Gain	36
13	Relaxation Delay	1.0000
14	Pulse Width	5.3000
15	Presaturation Frequency	
16	Acquisition Time	1.7064
17	Acquisition Date	2018-12-04T18:07:08
18	Modification Date	2018-12-04T18:07:10
19	Spectrometer Frequency	299.99
20	Spectral Width	4800.8
21	Lowest Frequency	-599.9
22	Nucleus	1H
23	Acquired Size	8192
24	Spectral Size	16384
25	Digital Resolution	0.29

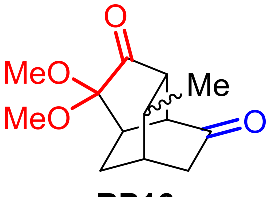

RP16
diastereomeric mixture

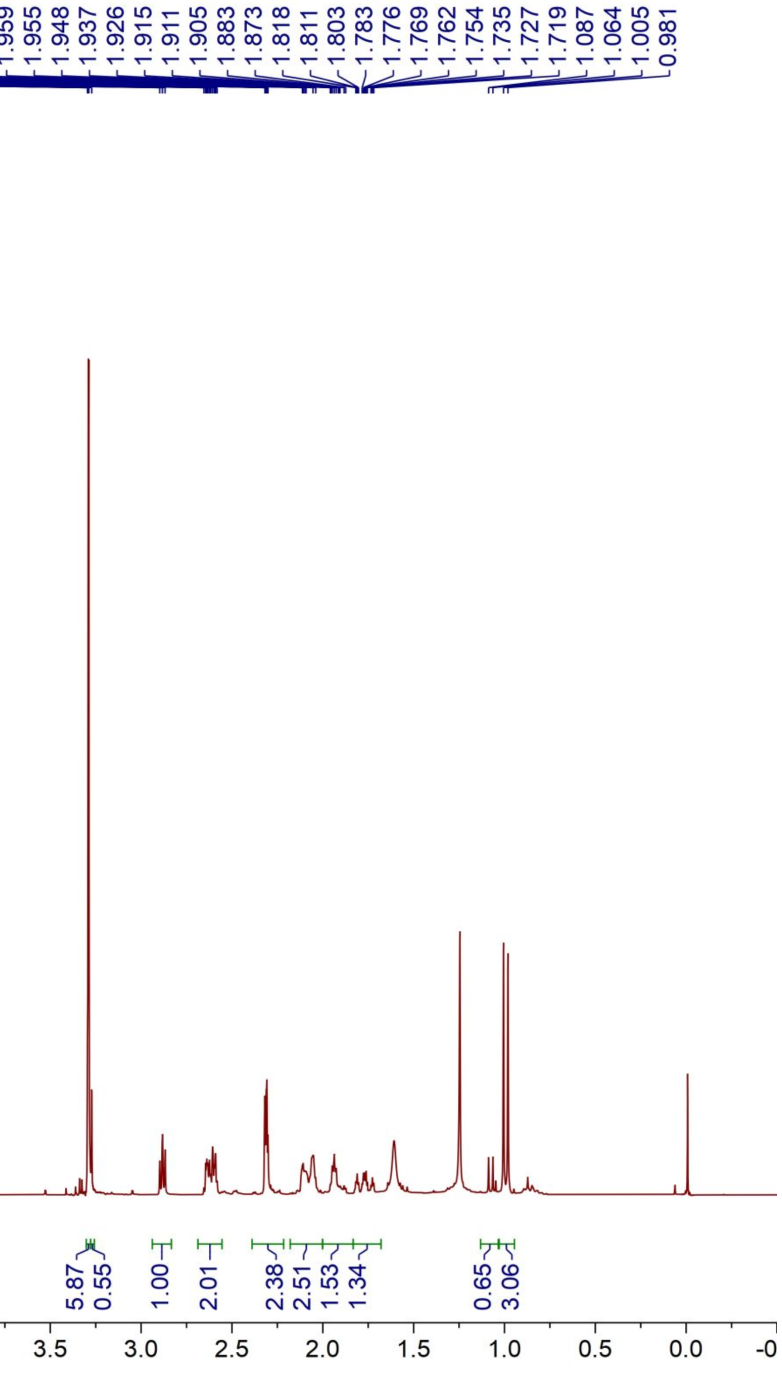

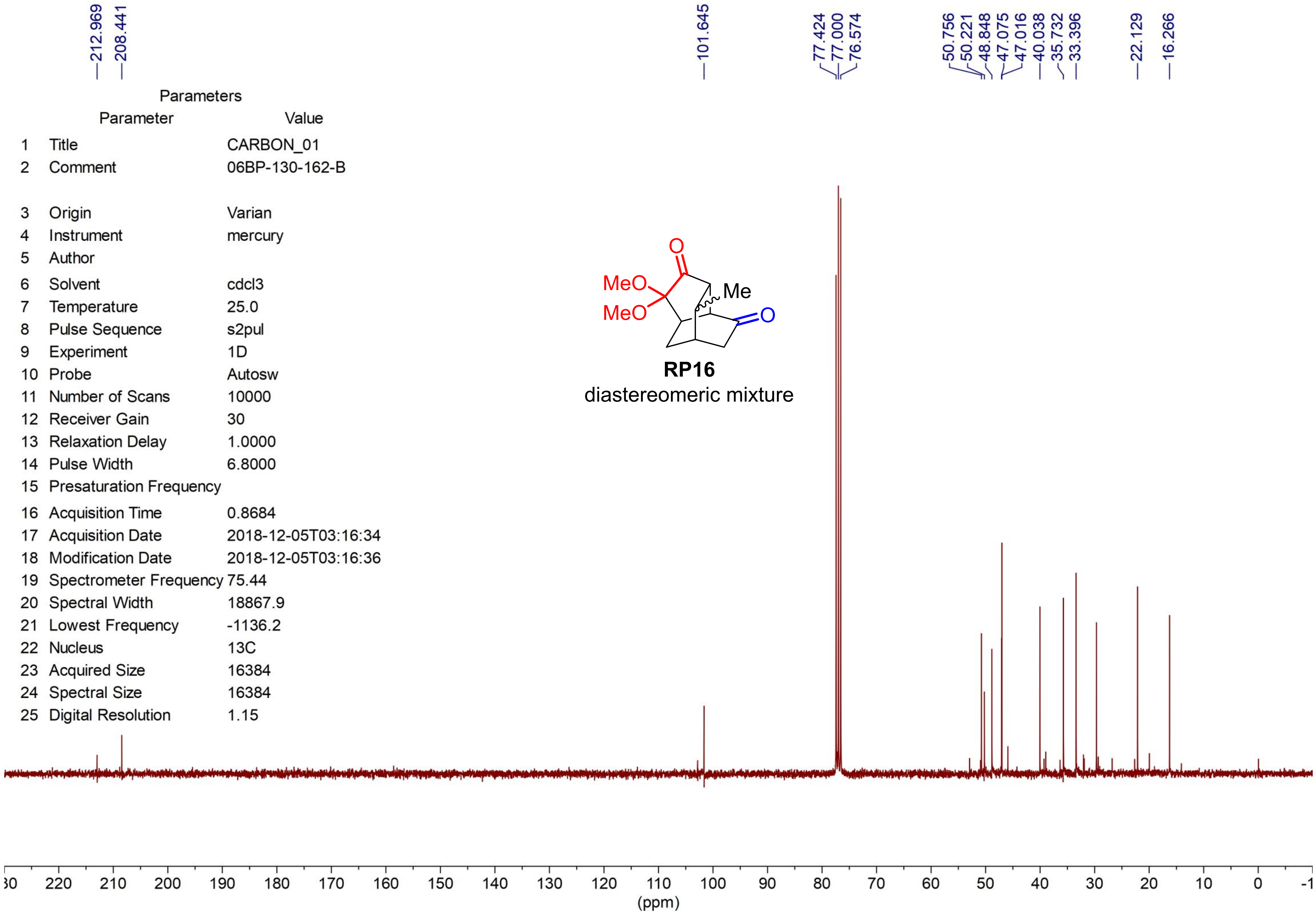

RP16
diastereomeric mixture

Parameters

	Parameter	Value
1	Title	CARBON_01
2	Comment	06BP-130-162-B
3	Origin	Varian
4	Instrument	mercury
5	Author	
6	Solvent	cdcl3
7	Temperature	25.0
8	Pulse Sequence	s2pul
9	Experiment	1D
10	Probe	Autosw
11	Number of Scans	10000
12	Receiver Gain	30
13	Relaxation Delay	1.0000
14	Pulse Width	6.8000
15	Presaturation Frequency	
16	Acquisition Time	0.8684
17	Acquisition Date	2018-12-05T03:16:34
18	Modification Date	2018-12-05T03:16:36
19	Spectrometer Frequency	75.44
20	Spectral Width	18867.9
21	Lowest Frequency	-1136.2
22	Nucleus	13C
23	Acquired Size	16384
24	Spectral Size	16384
25	Digital Resolution	1.15

Parameters

	Parameter	Value
1	Title	PROTON_01
2	Comment	06BP-130-162-A
3	Origin	Varian
4	Instrument	mercury
5	Author	
6	Solvent	cdcl3
7	Temperature	25.0
8	Pulse Sequence	s2pul
9	Experiment	1D
10	Probe	Autosw
11	Number of Scans	16
12	Receiver Gain	30
13	Relaxation Delay	1.0000
14	Pulse Width	5.3000
15	Presaturation Frequency	
16	Acquisition Time	1.7064
17	Acquisition Date	2018-12-08T19:10:18
18	Modification Date	2018-12-08T19:10:18
19	Spectrometer Frequency	299.99
20	Spectral Width	4800.8
21	Lowest Frequency	-599.8
22	Nucleus	1H
23	Acquired Size	8192
24	Spectral Size	16384
25	Digital Resolution	0.29

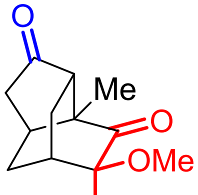

CP16

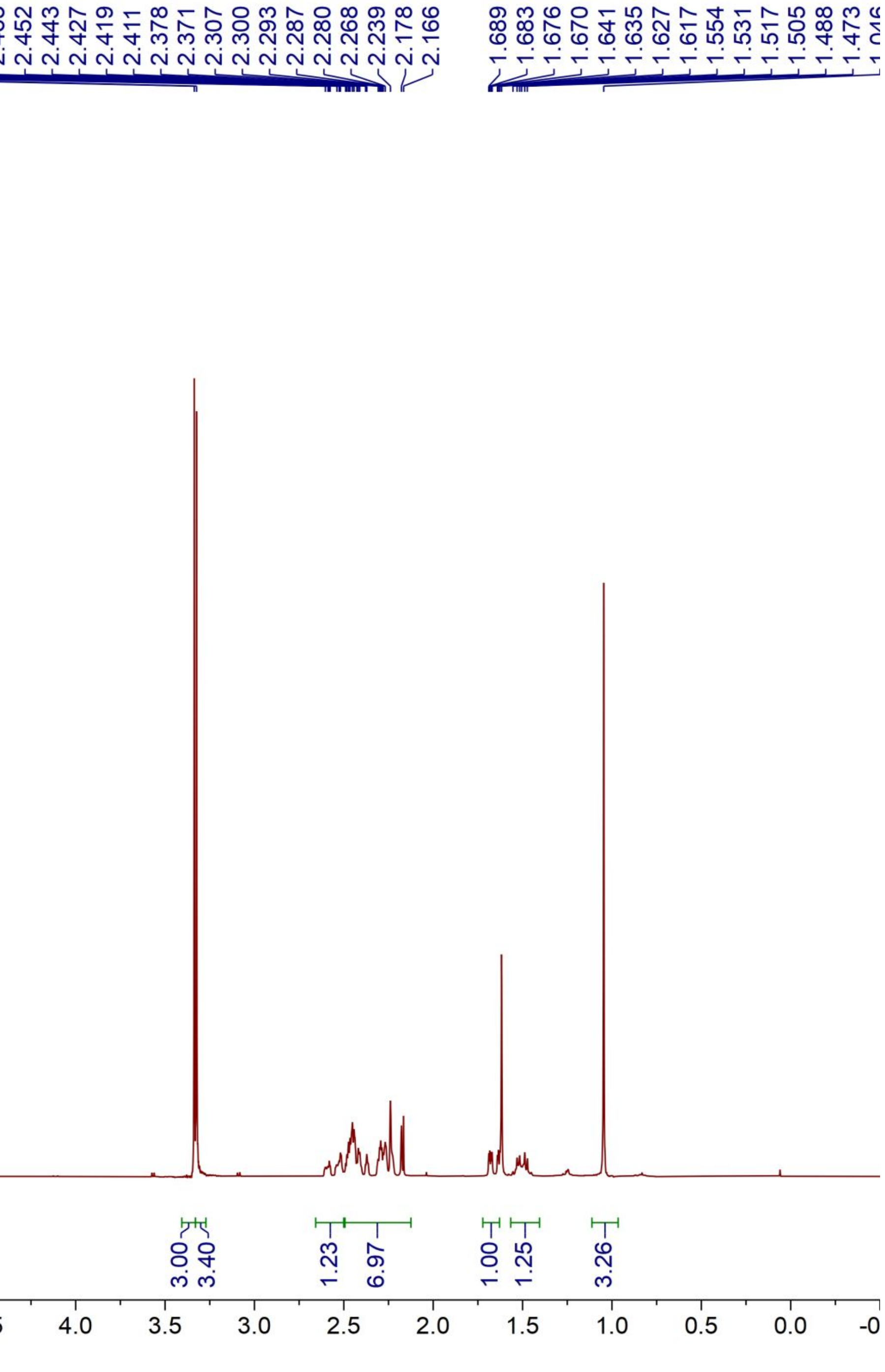

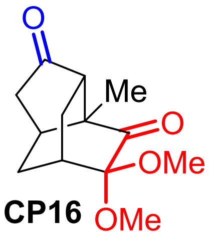

CP16

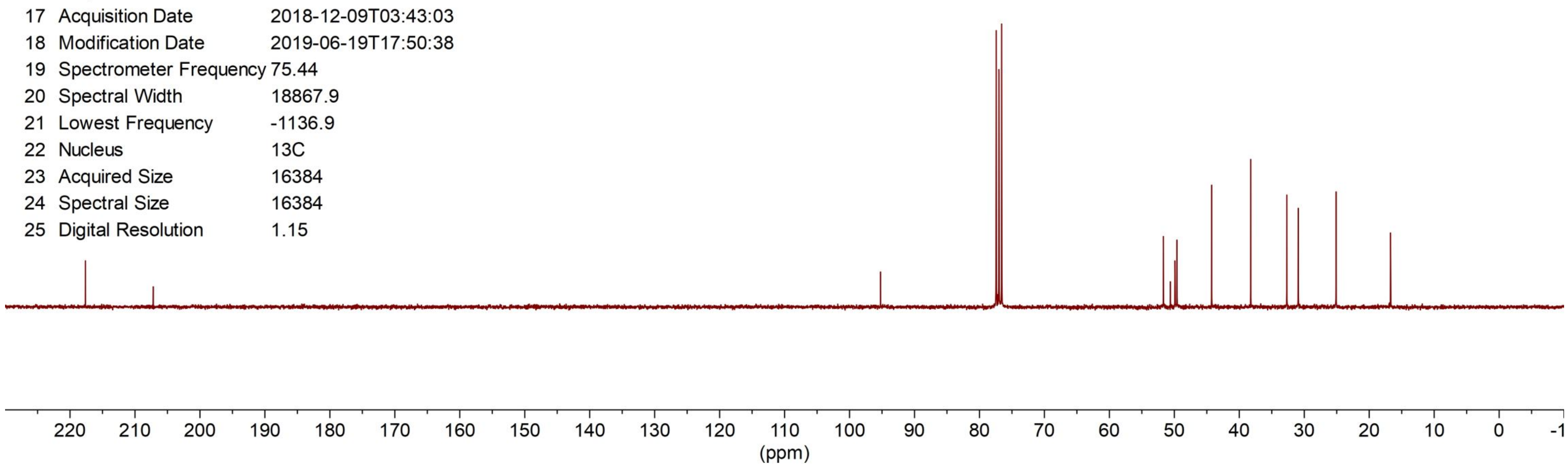

Parameters

	Parameter	Value
1	Title	CARBON_01
2	Comment	06BP-130-162-A
3	Origin	Varian
4	Instrument	mercury
5	Author	
6	Solvent	cdcl3
7	Temperature	25.0
8	Pulse Sequence	s2pul
9	Experiment	1D
10	Probe	Autosw
11	Number of Scans	10000
12	Receiver Gain	30
13	Relaxation Delay	1.0000
14	Pulse Width	6.8000
15	Presaturation Frequency	
16	Acquisition Time	0.8684
17	Acquisition Date	2018-12-09T03:43:03
18	Modification Date	2019-06-19T17:50:38
19	Spectrometer Frequency	75.44
20	Spectral Width	18867.9
21	Lowest Frequency	-1136.9
22	Nucleus	13C
23	Acquired Size	16384
24	Spectral Size	16384
25	Digital Resolution	1.15

Parameters

	Parameter	Value
1	Title	PROTON_01
2	Comment	07BP-068-082-B
3	Origin	Varian
4	Instrument	mercury
5	Author	
6	Solvent	cdcl3
7	Temperature	25.0
8	Pulse Sequence	s2pul
9	Experiment	1D
10	Probe	Autosw
11	Number of Scans	32
12	Receiver Gain	36
13	Relaxation Delay	1.0000
14	Pulse Width	5.3000
15	Presaturation Frequency	
16	Acquisition Time	1.7064
17	Acquisition Date	2019-01-04T18:25:21
18	Modification Date	2019-01-04T18:25:22
19	Spectrometer Frequency	299.99
20	Spectral Width	4800.8
21	Lowest Frequency	-599.8
22	Nucleus	1H
23	Acquired Size	8192
24	Spectral Size	16384
25	Digital Resolution	0.29

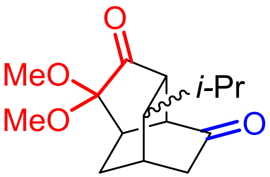

RP17
major diastereomer

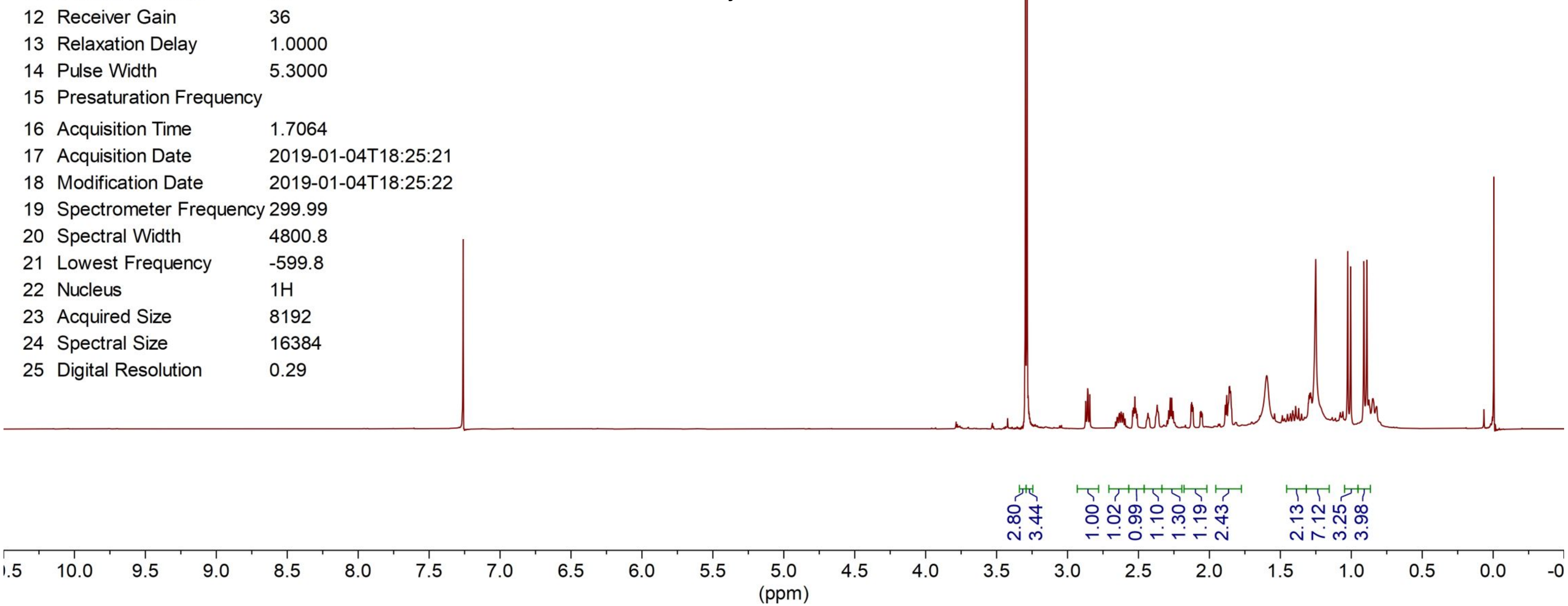

Parameters

	Parameter	Value
1	Title	CARBON_01
2	Comment	07BP-068-082-B
3	Origin	Varian
4	Instrument	mercury
5	Author	
6	Solvent	cdcl3
7	Temperature	25.0
8	Pulse Sequence	s2pul
9	Experiment	1D
10	Probe	Autosw
11	Number of Scans	10000
12	Receiver Gain	30
13	Relaxation Delay	1.0000
14	Pulse Width	6.8000
15	Presaturation Frequency	
16	Acquisition Time	0.8684
17	Acquisition Date	2019-01-05T06:39:02
18	Modification Date	2019-01-05T06:39:04
19	Spectrometer Frequency	75.44
20	Spectral Width	18867.9
21	Lowest Frequency	-1136.3
22	Nucleus	13C
23	Acquired Size	16384
24	Spectral Size	16384
25	Digital Resolution	1.15

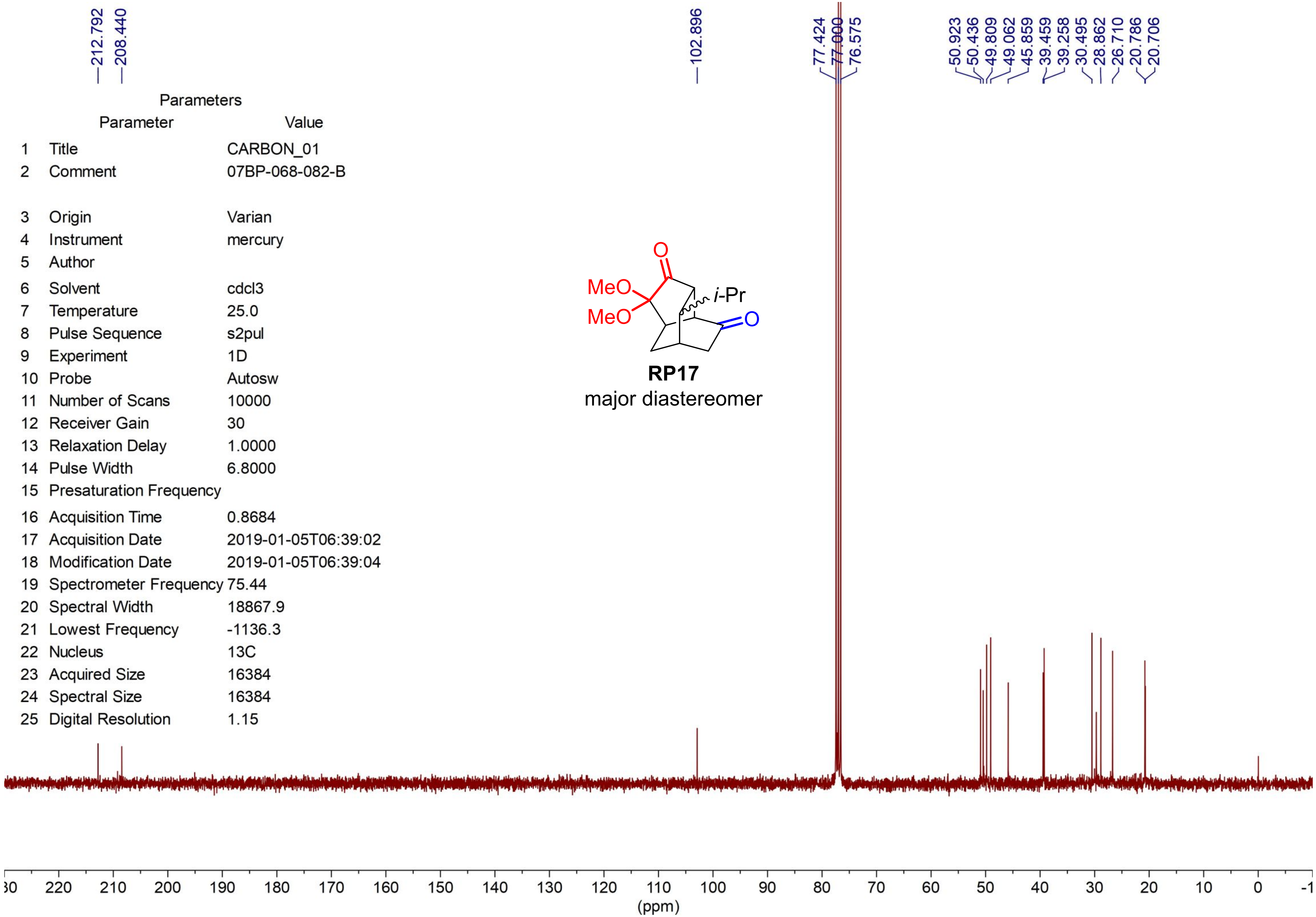

RP17
major diastereomer

	Parameter	Value
1	Title	PROTON_01
2	Comment	07BP-068-082-14
3	Origin	Varian
4	Instrument	mercury
5	Author	
6	Solvent	cdcl3
7	Temperature	25.0
8	Pulse Sequence	s2pul
9	Experiment	1D
10	Probe	Autosw
11	Number of Scans	16
12	Receiver Gain	30
13	Relaxation Delay	1.0000
14	Pulse Width	5.3500
15	Presaturation Frequency	
16	Acquisition Time	1.7064
17	Acquisition Date	2019-06-30T16:46:30
18	Modification Date	2019-06-30T16:46:32
19	Spectrometer Frequency	299.99
20	Spectral Width	4800.8
21	Lowest Frequency	-599.5
22	Nucleus	1H
23	Acquired Size	8192
24	Spectral Size	16384
25	Digital Resolution	0.29

Parameters

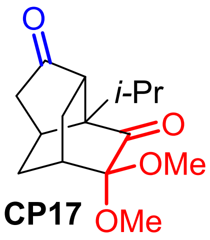

CP17

+

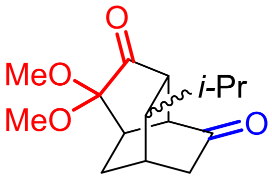

RP17
minro diastereomer

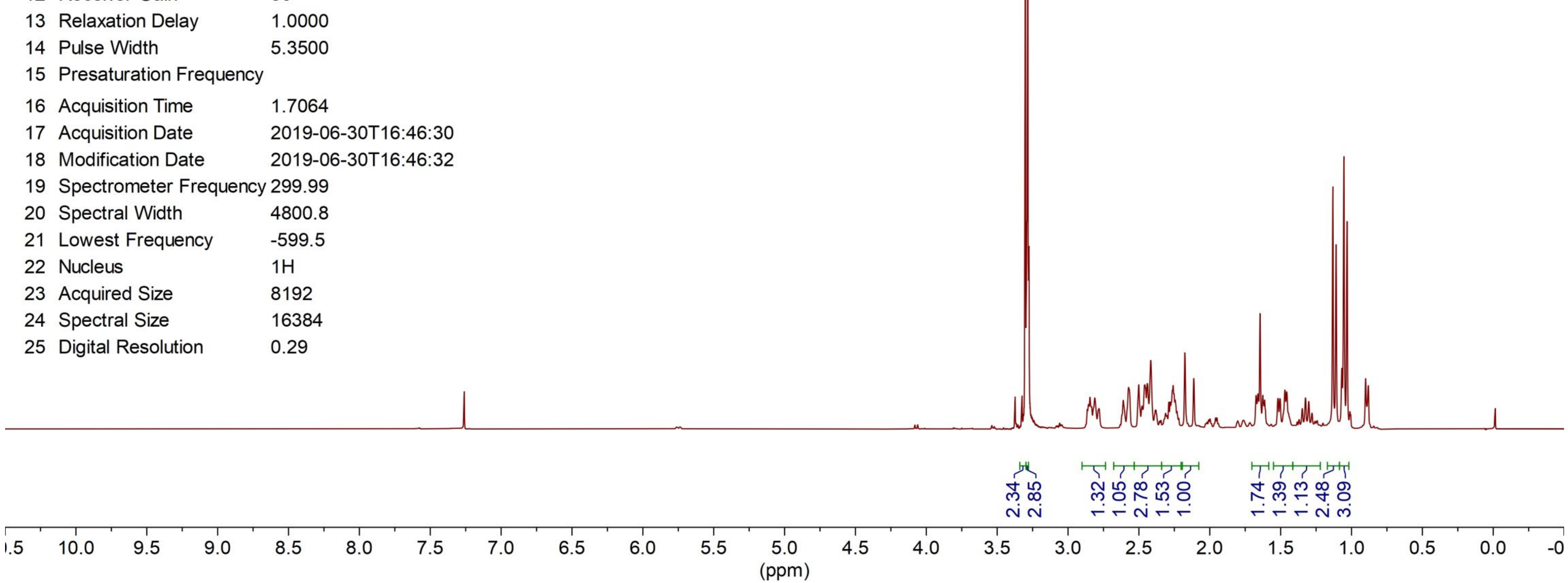

	Parameter	Value
1	Title	CARBON_01
2	Comment	07BP-068-082-14
3	Origin	Varian
4	Instrument	mercury
5	Author	
6	Solvent	cdcl3
7	Temperature	25.0
8	Pulse Sequence	s2pul
9	Experiment	1D
10	Probe	Autosw
11	Number of Scans	16384
12	Receiver Gain	30
13	Relaxation Delay	1.0000
14	Pulse Width	7.3000
15	Presaturation Frequency	
16	Acquisition Time	0.8684
17	Acquisition Date	2019-07-01T01:21:04
18	Modification Date	2019-07-01T01:21:06
19	Spectrometer Frequency	75.44
20	Spectral Width	18867.9
21	Lowest Frequency	-1136.4
22	Nucleus	13C
23	Acquired Size	16384
24	Spectral Size	16384
25	Digital Resolution	1.15

Parameters

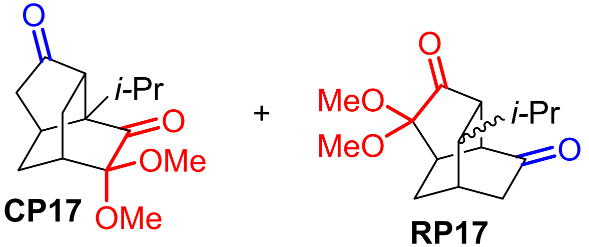

CP17

RP17
minro diastereomer

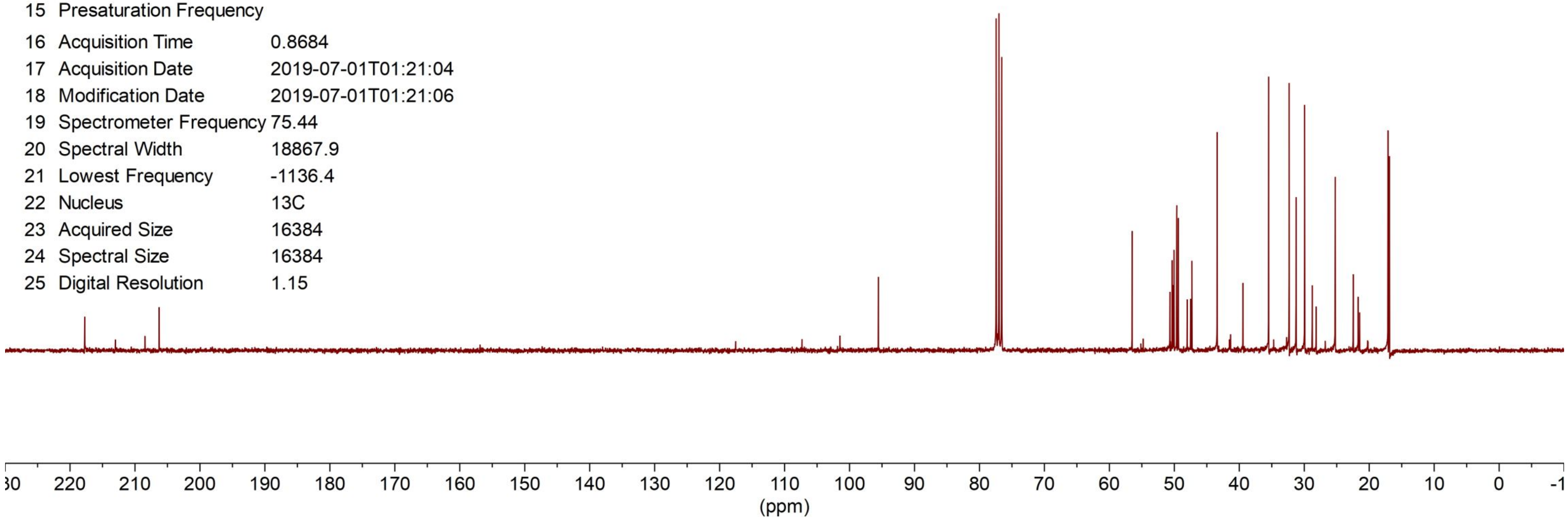

Parameters

	Parameter	Value
1	Title	PROTON_01
2	Comment	07BP-068-082-17
3	Origin	Varian
4	Instrument	mercury
5	Author	
6	Solvent	cdcl3
7	Temperature	25.0
8	Pulse Sequence	s2pul
9	Experiment	1D
10	Probe	Autosw
11	Number of Scans	16
12	Receiver Gain	30
13	Relaxation Delay	1.0000
14	Pulse Width	5.3500
15	Presaturation Frequency	
16	Acquisition Time	1.7064
17	Acquisition Date	2019-06-28T18:27:05
18	Modification Date	2019-06-28T18:27:06
19	Spectrometer Frequency	299.99
20	Spectral Width	4800.8
21	Lowest Frequency	-599.8
22	Nucleus	1H
23	Acquired Size	8192
24	Spectral Size	16384
25	Digital Resolution	0.29

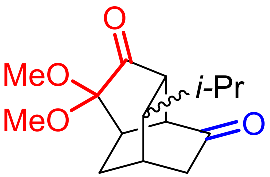

RP17
minro diastereomer

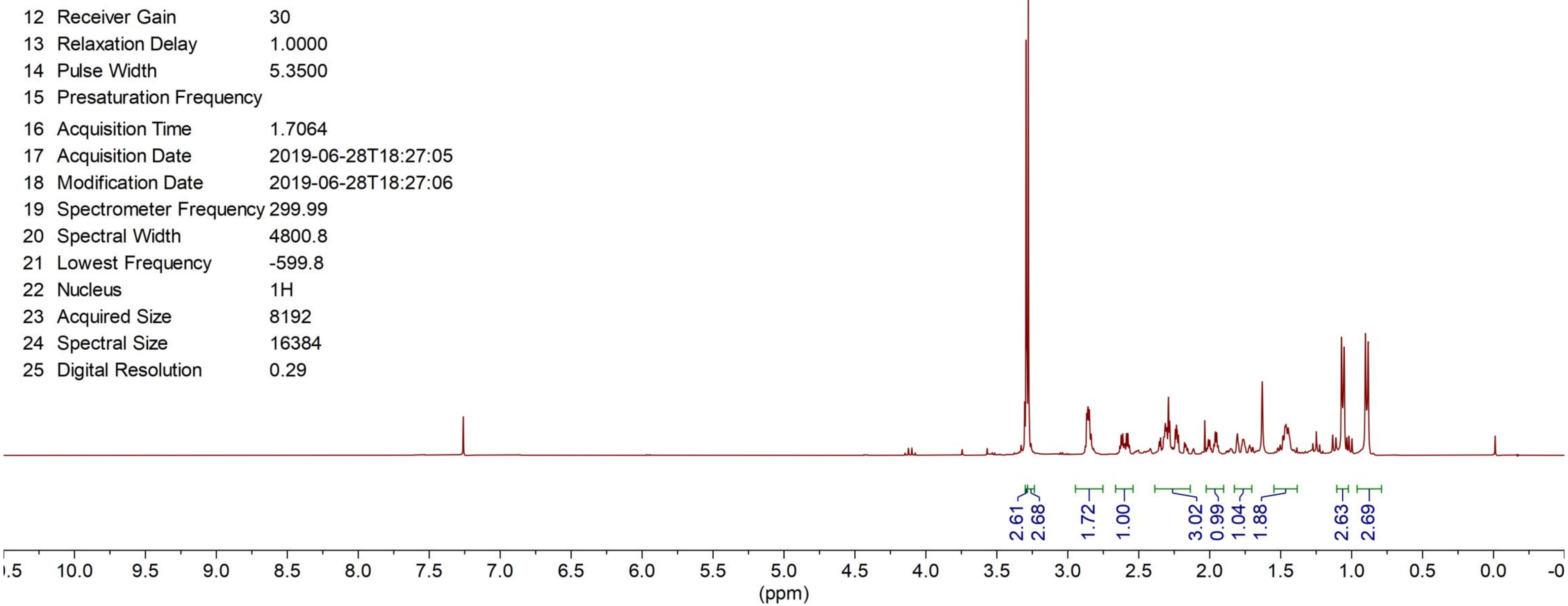

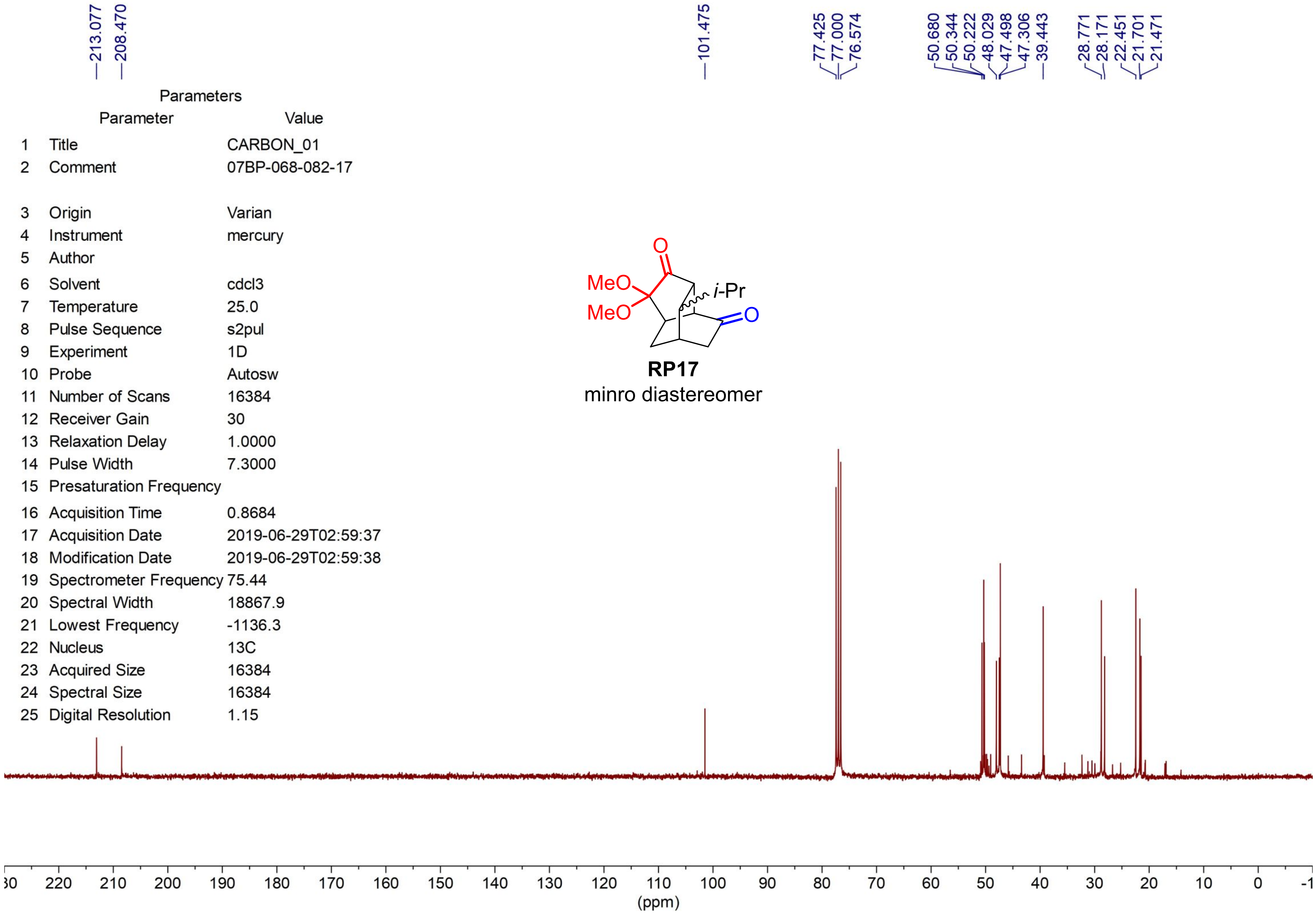

Parameters

	Parameter	Value
1	Title	CARBON_01
2	Comment	07BP-068-082-17
3	Origin	Varian
4	Instrument	mercury
5	Author	
6	Solvent	cdcl3
7	Temperature	25.0
8	Pulse Sequence	s2pul
9	Experiment	1D
10	Probe	Autosw
11	Number of Scans	16384
12	Receiver Gain	30
13	Relaxation Delay	1.0000
14	Pulse Width	7.3000
15	Presaturation Frequency	
16	Acquisition Time	0.8684
17	Acquisition Date	2019-06-29T02:59:37
18	Modification Date	2019-06-29T02:59:38
19	Spectrometer Frequency	75.44
20	Spectral Width	18867.9
21	Lowest Frequency	-1136.3
22	Nucleus	13C
23	Acquired Size	16384
24	Spectral Size	16384
25	Digital Resolution	1.15

	Parameter	Value
1	Title	PROTON_01
2	Comment	07BP-113-148
3	Origin	Varian
4	Instrument	mercury
5	Author	
6	Solvent	cdcl3
7	Temperature	25.0
8	Pulse Sequence	s2pul
9	Experiment	1D
10	Probe	Autosw
11	Number of Scans	16
12	Receiver Gain	39
13	Relaxation Delay	1.0000
14	Pulse Width	5.3500
15	Presaturation Frequency	
16	Acquisition Time	1.7064
17	Acquisition Date	2019-08-15T17:18:56
18	Modification Date	2019-08-15T17:18:56
19	Spectrometer Frequency	299.99
20	Spectral Width	4800.8
21	Lowest Frequency	-599.8
22	Nucleus	1H
23	Acquired Size	8192
24	Spectral Size	16384
25	Digital Resolution	0.29

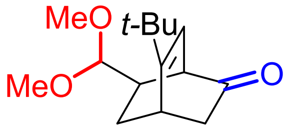

rearrangement/decarbonylation
side product **22**

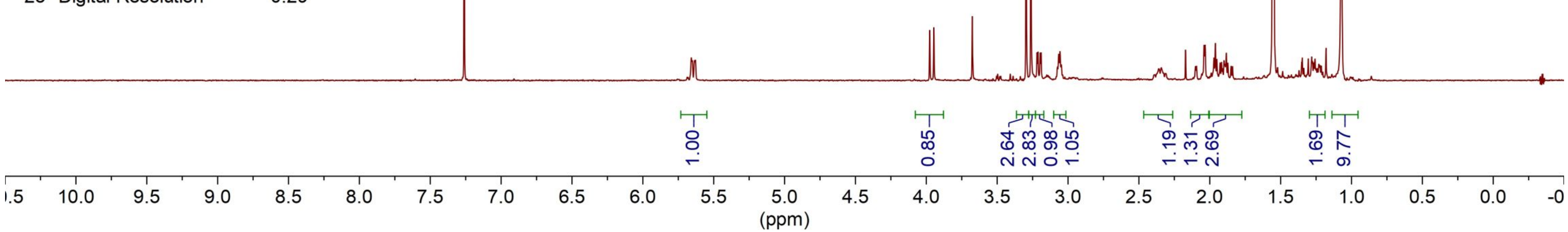

Parameters

	Parameter	Value
1	Title	CARBON_01
2	Comment	07BP-113-148-A
3	Origin	Varian
4	Instrument	mercury
5	Author	
6	Solvent	cdcl3
7	Temperature	25.0
8	Pulse Sequence	s2pul
9	Experiment	1D
10	Probe	autosw
11	Number of Scans	10000
12	Receiver Gain	30
13	Relaxation Delay	1.0000
14	Pulse Width	6.8000
15	Presaturation Frequency	
16	Acquisition Time	1.3042
17	Acquisition Date	2019-05-07T00:50:20
18	Modification Date	2019-05-16T11:39:22
19	Spectrometer Frequency	100.57
20	Spectral Width	25125.6
21	Lowest Frequency	-1501.1
22	Nucleus	13C
23	Acquired Size	32768
24	Spectral Size	32768
25	Digital Resolution	0.77

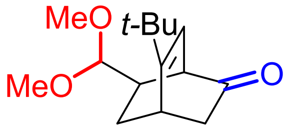

rearrangement/decarbonylation
side product **22**

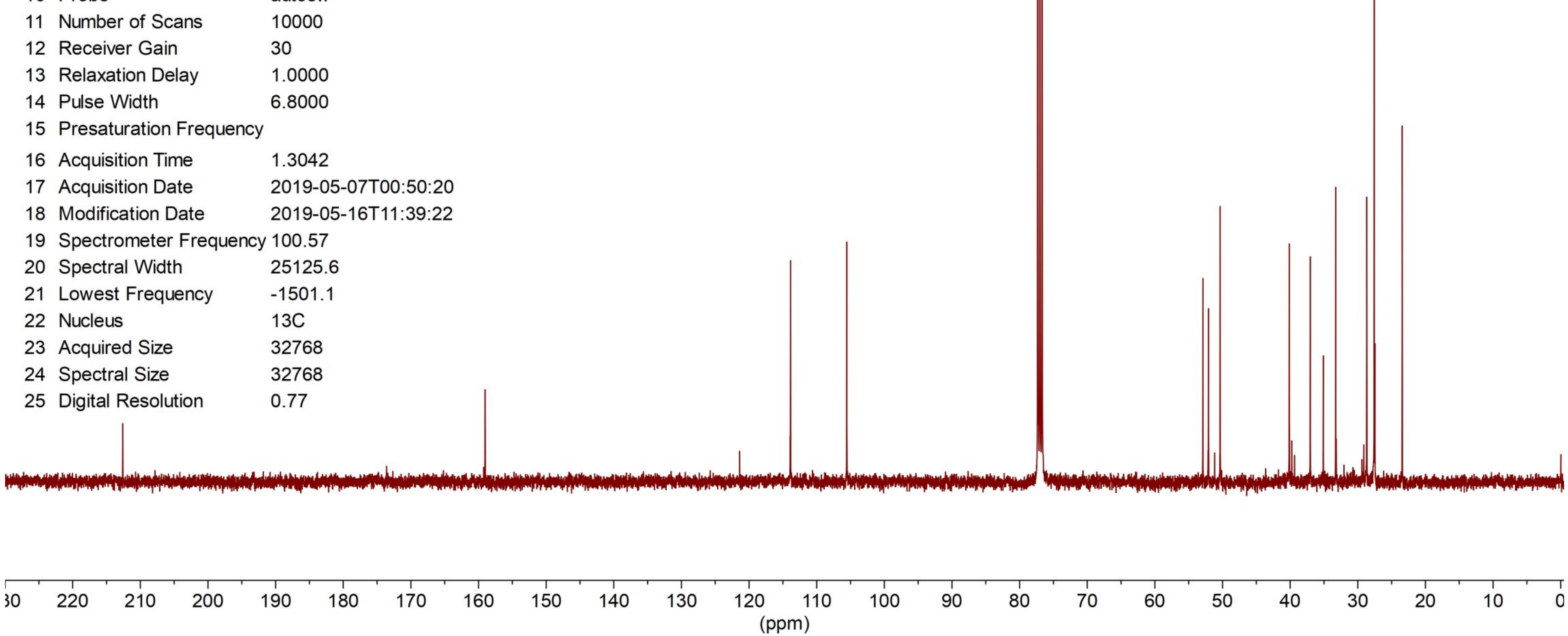

Parameters

	Parameter	Value
1	Title	PROTON_01
2	Comment	07BP-113-026-ZUO4
3	Origin	Varian
4	Instrument	mercury
5	Author	
6	Solvent	cdcl3
7	Temperature	25.0
8	Pulse Sequence	s2pul
9	Experiment	1D
10	Probe	autosw
11	Number of Scans	32
12	Receiver Gain	32
13	Relaxation Delay	1.0000
14	Pulse Width	6.8000
15	Presaturation Frequency	
16	Acquisition Time	2.5608
17	Acquisition Date	2019-08-28T15:54:53
18	Modification Date	2019-08-28T15:54:56
19	Spectrometer Frequency	399.93
20	Spectral Width	6398.0
21	Lowest Frequency	-798.8
22	Nucleus	1H
23	Acquired Size	16384
24	Spectral Size	65536
25	Digital Resolution	0.10

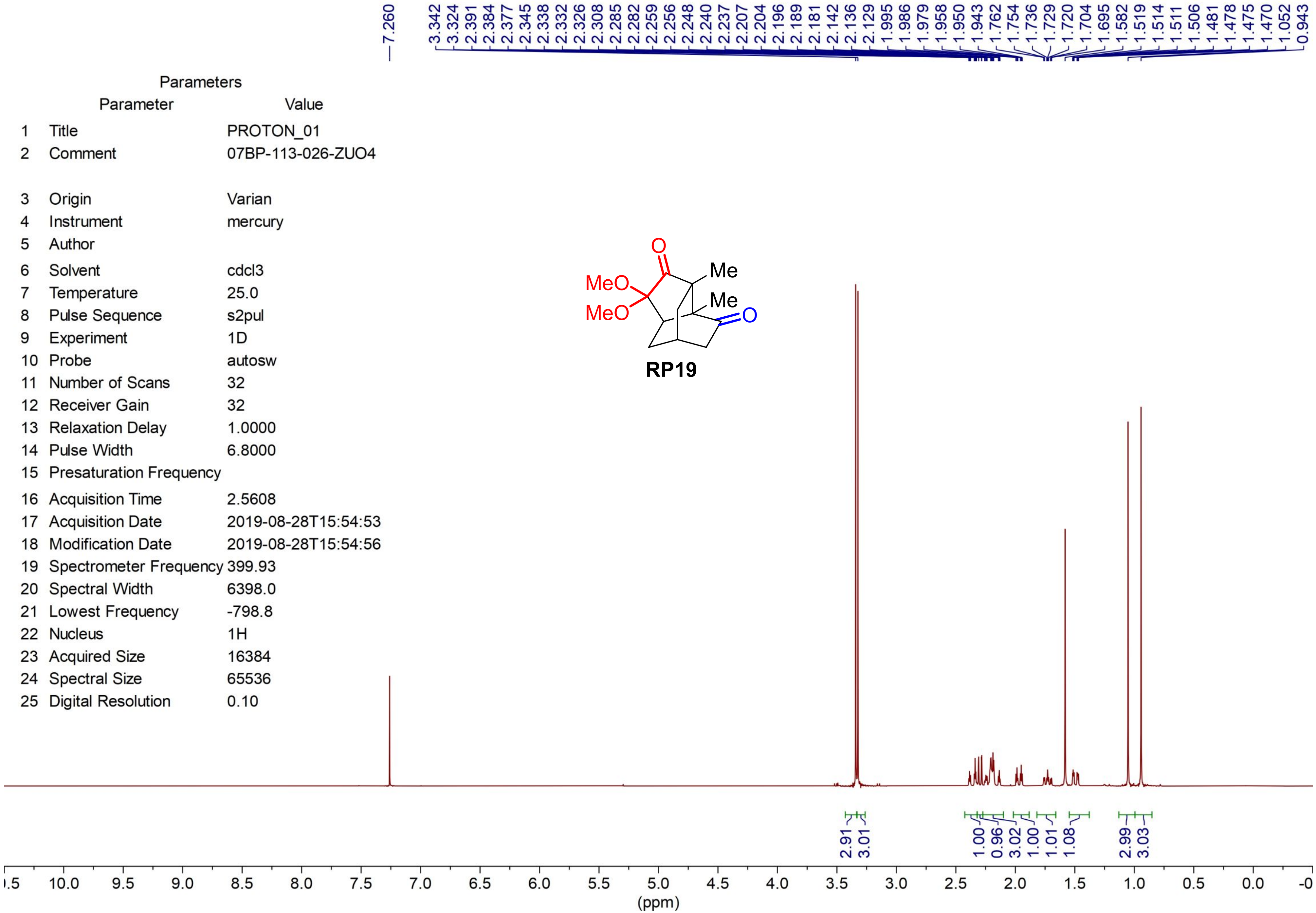

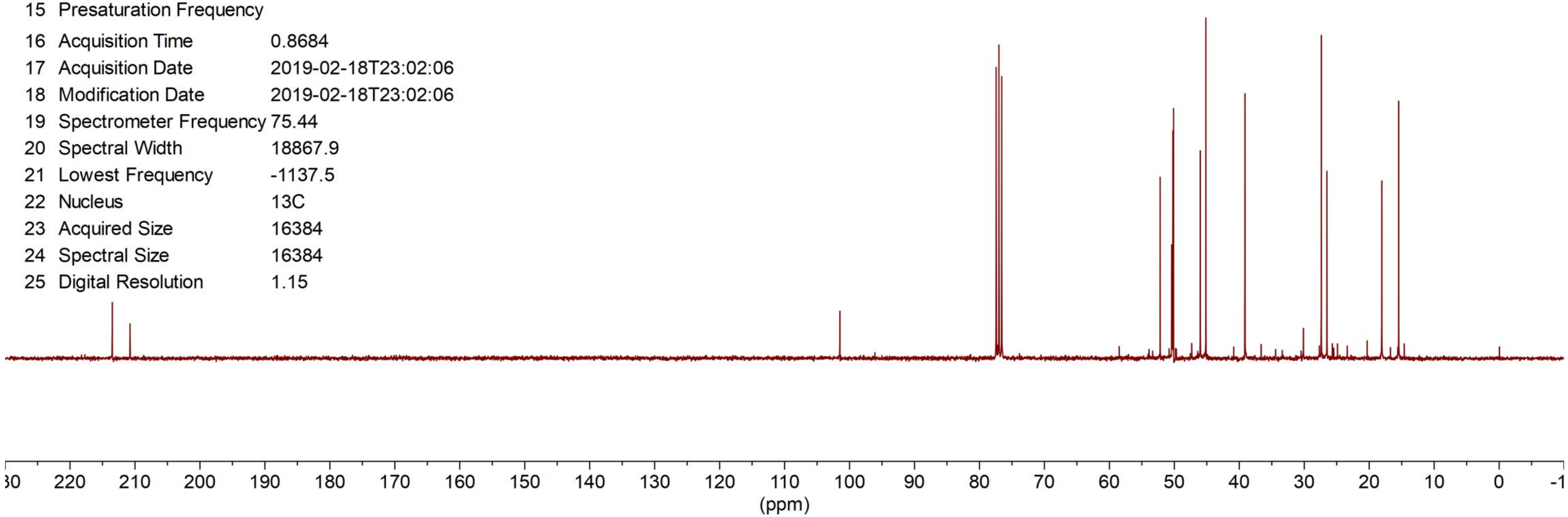

Parameters

	Parameter	Value
1	Title	CARBON_01
2	Comment	07BP-113-026-15
3	Origin	Varian
4	Instrument	mercury
5	Author	
6	Solvent	cdcl3
7	Temperature	25.0
8	Pulse Sequence	s2pul
9	Experiment	1D
10	Probe	Autosw
11	Number of Scans	10000
12	Receiver Gain	30
13	Relaxation Delay	1.0000
14	Pulse Width	6.8000
15	Presaturation Frequency	
16	Acquisition Time	0.8684
17	Acquisition Date	2019-02-18T23:02:06
18	Modification Date	2019-02-18T23:02:06
19	Spectrometer Frequency	75.44
20	Spectral Width	18867.9
21	Lowest Frequency	-1137.5
22	Nucleus	13C
23	Acquired Size	16384
24	Spectral Size	16384
25	Digital Resolution	1.15

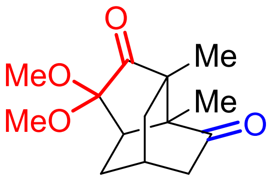

RP19

Parameters

	Parameter	Value
1	Title	PROTON_01
2	Comment	07BP-113-026-35
3	Origin	Varian
4	Instrument	mercury
5	Author	
6	Solvent	cdcl3
7	Temperature	25.0
8	Pulse Sequence	s2pul
9	Experiment	1D
10	Probe	autosw
11	Number of Scans	32
12	Receiver Gain	38
13	Relaxation Delay	1.0000
14	Pulse Width	6.8000
15	Presaturation Frequency	
16	Acquisition Time	2.5608
17	Acquisition Date	2019-08-28T16:52:50
18	Modification Date	2019-08-28T16:52:52
19	Spectrometer Frequency	399.93
20	Spectral Width	6398.0
21	Lowest Frequency	-798.8
22	Nucleus	1H
23	Acquired Size	16384
24	Spectral Size	65536
25	Digital Resolution	0.10

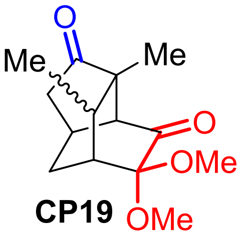

CP19

diastereomeric mixture

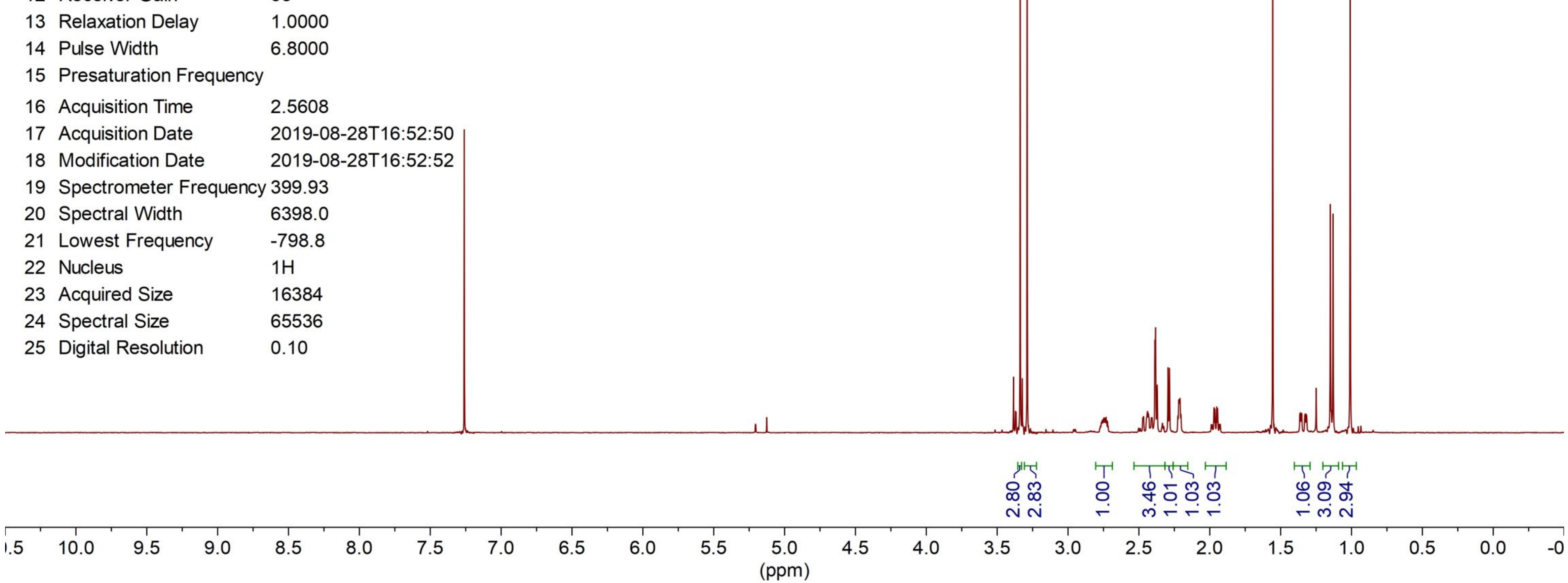

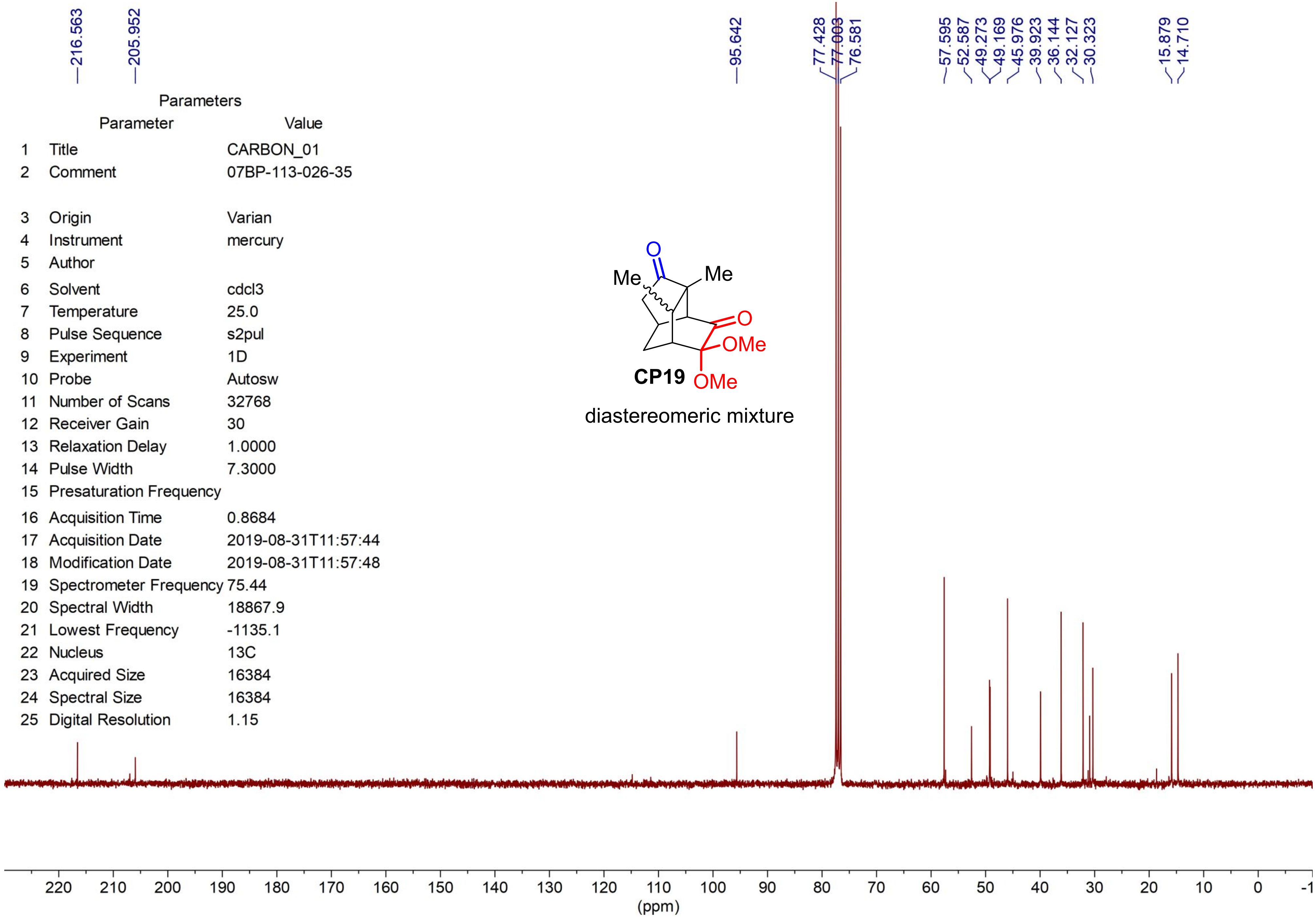

Parameters

	Parameter	Value
1	Title	CARBON_01
2	Comment	07BP-113-026-35
3	Origin	Varian
4	Instrument	mercury
5	Author	
6	Solvent	cdcl3
7	Temperature	25.0
8	Pulse Sequence	s2pul
9	Experiment	1D
10	Probe	Autosw
11	Number of Scans	32768
12	Receiver Gain	30
13	Relaxation Delay	1.0000
14	Pulse Width	7.3000
15	Presaturation Frequency	
16	Acquisition Time	0.8684
17	Acquisition Date	2019-08-31T11:57:44
18	Modification Date	2019-08-31T11:57:48
19	Spectrometer Frequency	75.44
20	Spectral Width	18867.9
21	Lowest Frequency	-1135.1
22	Nucleus	13C
23	Acquired Size	16384
24	Spectral Size	16384
25	Digital Resolution	1.15

CP19

diastereomeric mixture

Parameters

	Parameter	Value
1	Title	PROTON_01
2	Comment	06BP-130-188-23
3	Origin	Varian
4	Instrument	mercury
5	Author	
6	Solvent	cdcl3
7	Temperature	25.0
8	Pulse Sequence	s2pul
9	Experiment	1D
10	Probe	Autosw
11	Number of Scans	16
12	Receiver Gain	36
13	Relaxation Delay	1.0000
14	Pulse Width	5.3000
15	Presaturation Frequency	
16	Acquisition Time	1.7064
17	Acquisition Date	2019-05-15T23:56:05
18	Modification Date	2019-05-15T23:56:06
19	Spectrometer Frequency	299.99
20	Spectral Width	4800.8
21	Lowest Frequency	-599.9
22	Nucleus	1H
23	Acquired Size	8192
24	Spectral Size	16384
25	Digital Resolution	0.29

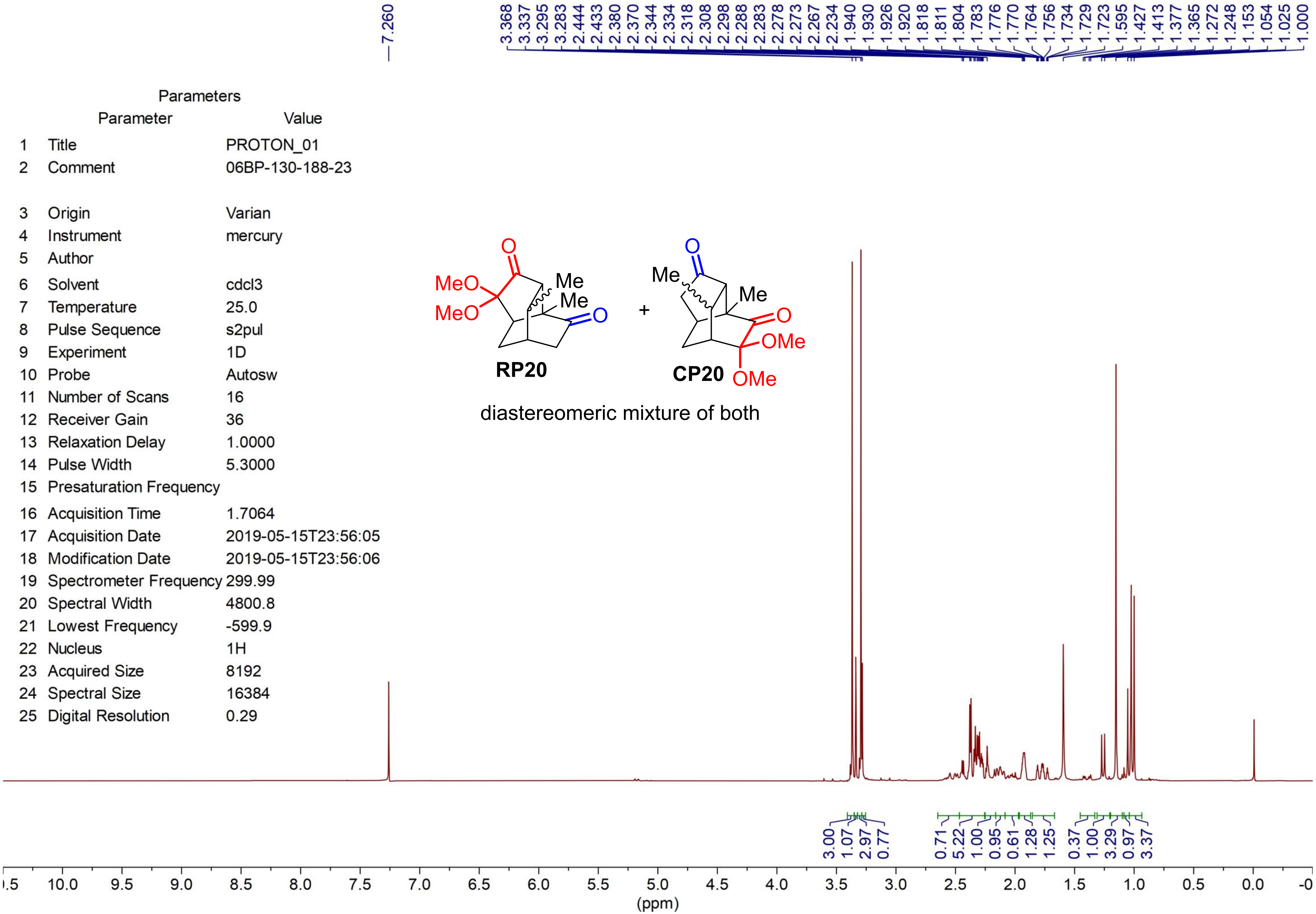

Parameters

	Parameter	Value
1	Title	CARBON_01
2	Comment	06BP-130-188-23
3	Origin	Varian
4	Instrument	mercury
5	Author	
6	Solvent	cdcl3
7	Temperature	25.0
8	Pulse Sequence	s2pul
9	Experiment	1D
10	Probe	Autosw
11	Number of Scans	10000
12	Receiver Gain	30
13	Relaxation Delay	1.0000
14	Pulse Width	6.8000
15	Presaturation Frequency	
16	Acquisition Time	0.8684
17	Acquisition Date	2019-05-16T05:09:02
18	Modification Date	2019-05-16T05:09:02
19	Spectrometer Frequency	75.44
20	Spectral Width	18867.9
21	Lowest Frequency	-1135.8
22	Nucleus	13C
23	Acquired Size	16384
24	Spectral Size	16384
25	Digital Resolution	1.15

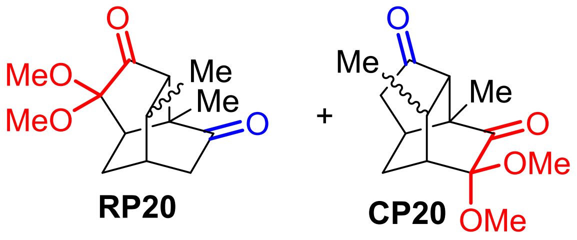

RP20 CP20

diastereomeric mixture of both

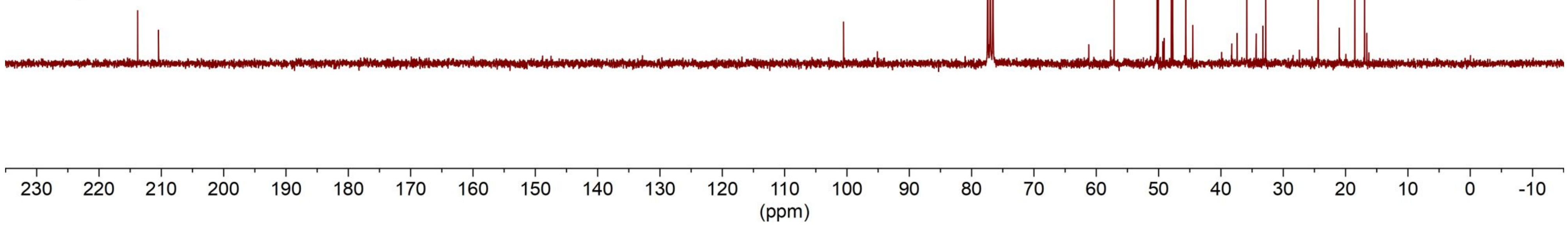

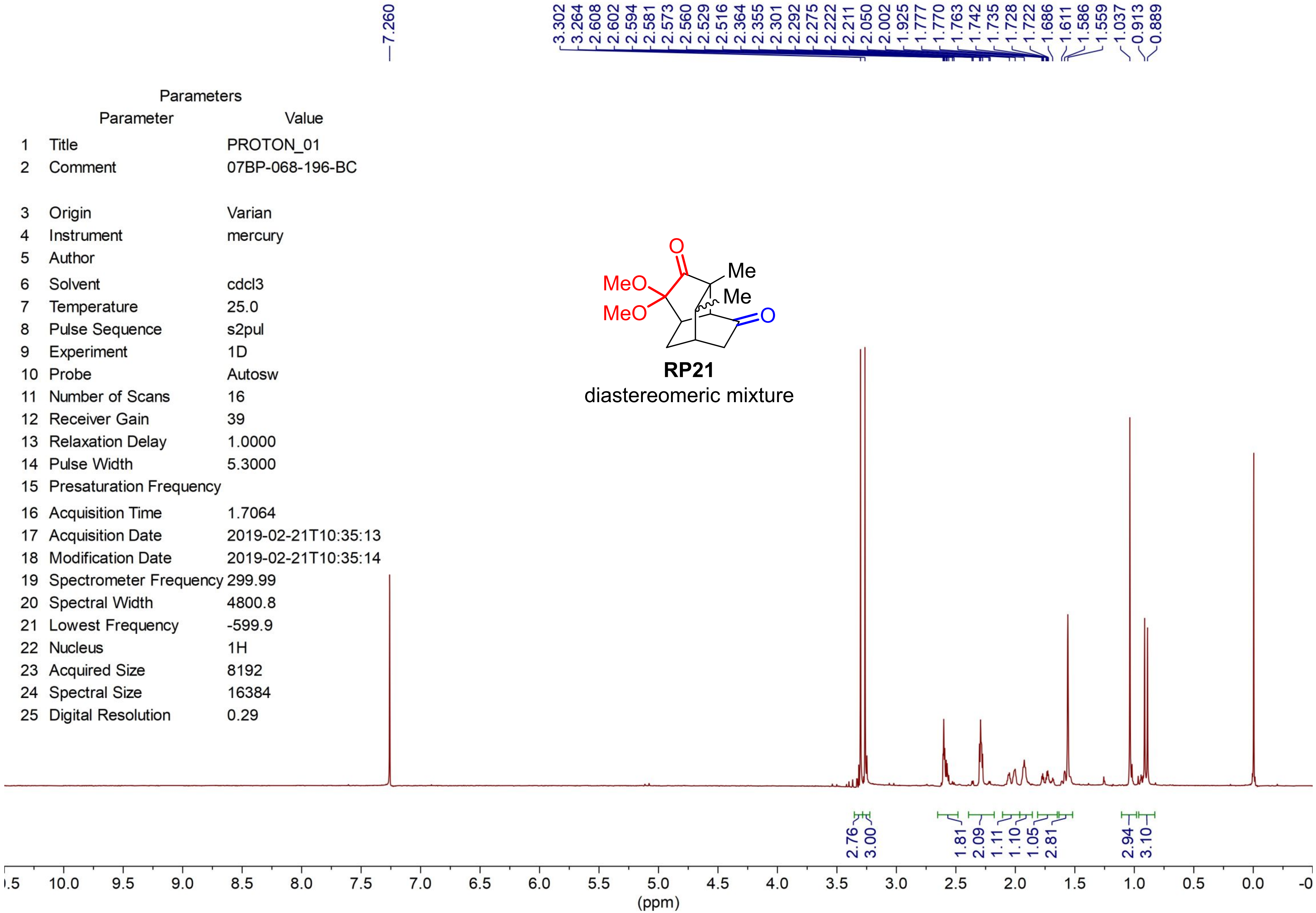

Parameters

	Parameter	Value
1	Title	PROTON_01
2	Comment	07BP-068-196-BC
3	Origin	Varian
4	Instrument	mercury
5	Author	
6	Solvent	cdcl3
7	Temperature	25.0
8	Pulse Sequence	s2pul
9	Experiment	1D
10	Probe	Autosw
11	Number of Scans	16
12	Receiver Gain	39
13	Relaxation Delay	1.0000
14	Pulse Width	5.3000
15	Presaturation Frequency	
16	Acquisition Time	1.7064
17	Acquisition Date	2019-02-21T10:35:13
18	Modification Date	2019-02-21T10:35:14
19	Spectrometer Frequency	299.99
20	Spectral Width	4800.8
21	Lowest Frequency	-599.9
22	Nucleus	1H
23	Acquired Size	8192
24	Spectral Size	16384
25	Digital Resolution	0.29

RP21
diastereomeric mixture

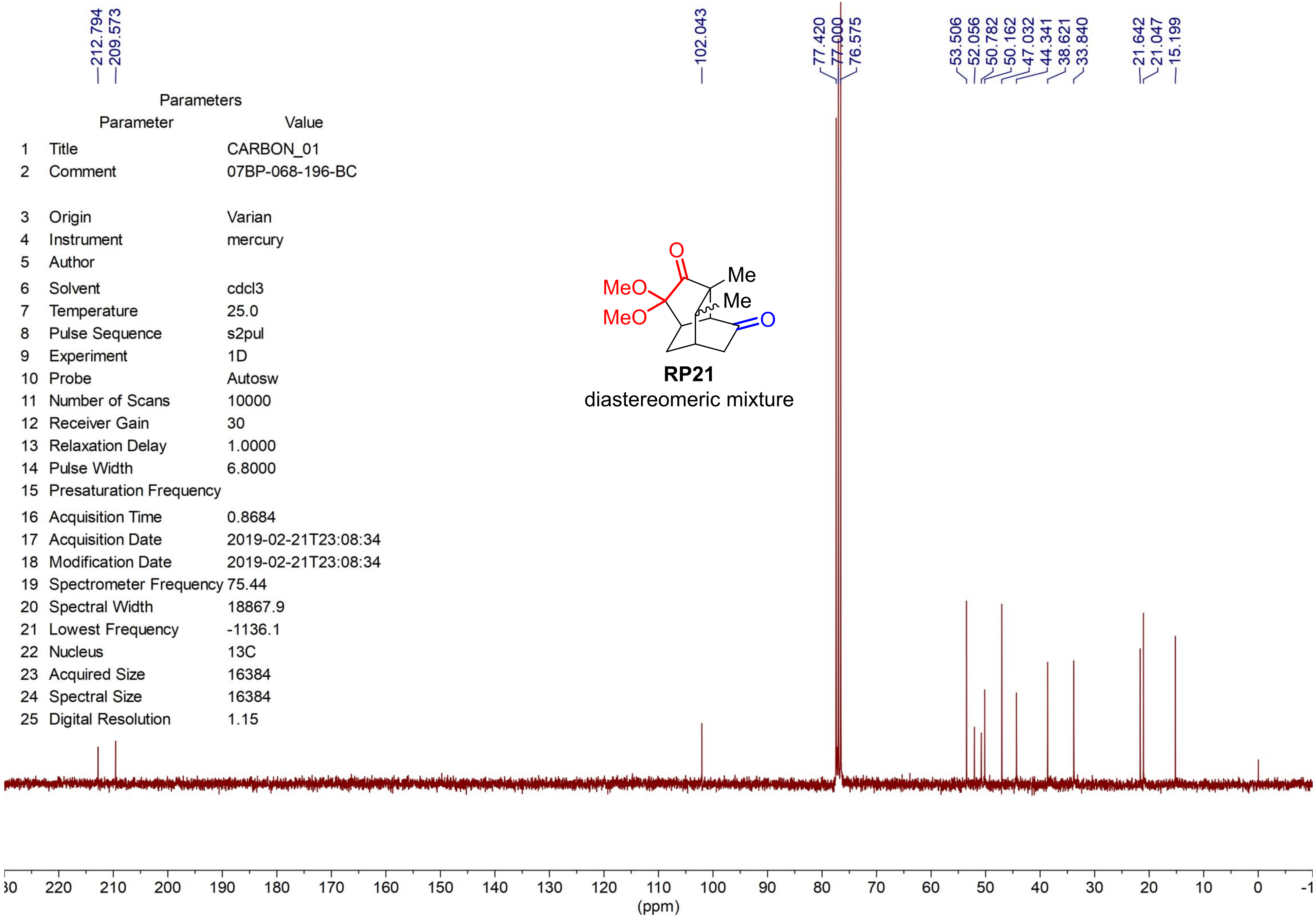

Parameters

	Parameter	Value
1	Title	CARBON_01
2	Comment	07BP-068-196-BC
3	Origin	Varian
4	Instrument	mercury
5	Author	
6	Solvent	cdcl3
7	Temperature	25.0
8	Pulse Sequence	s2pul
9	Experiment	1D
10	Probe	Autosw
11	Number of Scans	10000
12	Receiver Gain	30
13	Relaxation Delay	1.0000
14	Pulse Width	6.8000
15	Presaturation Frequency	
16	Acquisition Time	0.8684
17	Acquisition Date	2019-02-21T23:08:34
18	Modification Date	2019-02-21T23:08:34
19	Spectrometer Frequency	75.44
20	Spectral Width	18867.9
21	Lowest Frequency	-1136.1
22	Nucleus	13C
23	Acquired Size	16384
24	Spectral Size	16384
25	Digital Resolution	1.15

RP21
diastereomeric mixture

Parameters

	Parameter	Value
1	Title	PROTON_01
2	Comment	07BP-068-196-A
3	Origin	Varian
4	Instrument	mercury
5	Author	
6	Solvent	cdcl3
7	Temperature	25.0
8	Pulse Sequence	s2pul
9	Experiment	1D
10	Probe	Autosw
11	Number of Scans	16
12	Receiver Gain	30
13	Relaxation Delay	1.0000
14	Pulse Width	5.3000
15	Presaturation Frequency	
16	Acquisition Time	1.7064
17	Acquisition Date	2018-12-28T17:57:28
18	Modification Date	2018-12-28T17:57:30
19	Spectrometer Frequency	299.99
20	Spectral Width	4800.8
21	Lowest Frequency	-599.8
22	Nucleus	1H
23	Acquired Size	8192
24	Spectral Size	16384
25	Digital Resolution	0.29

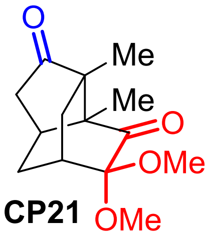

CP21

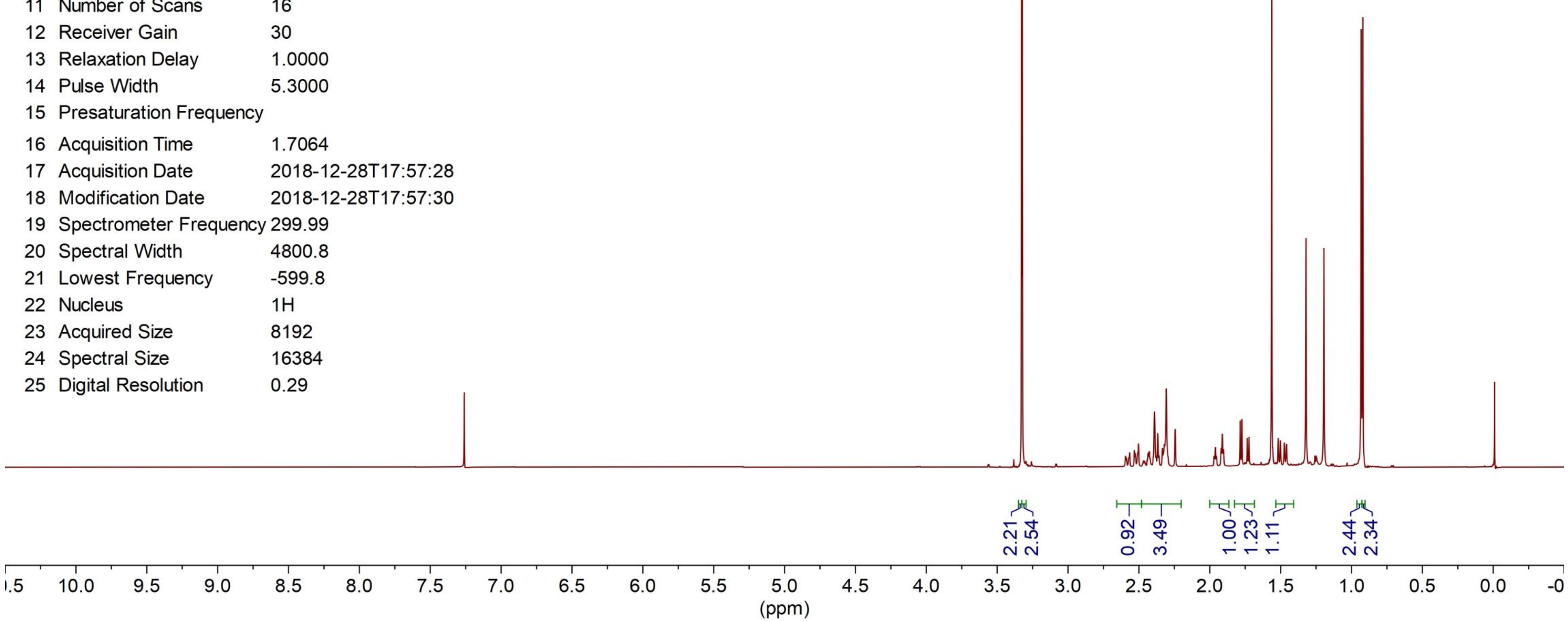

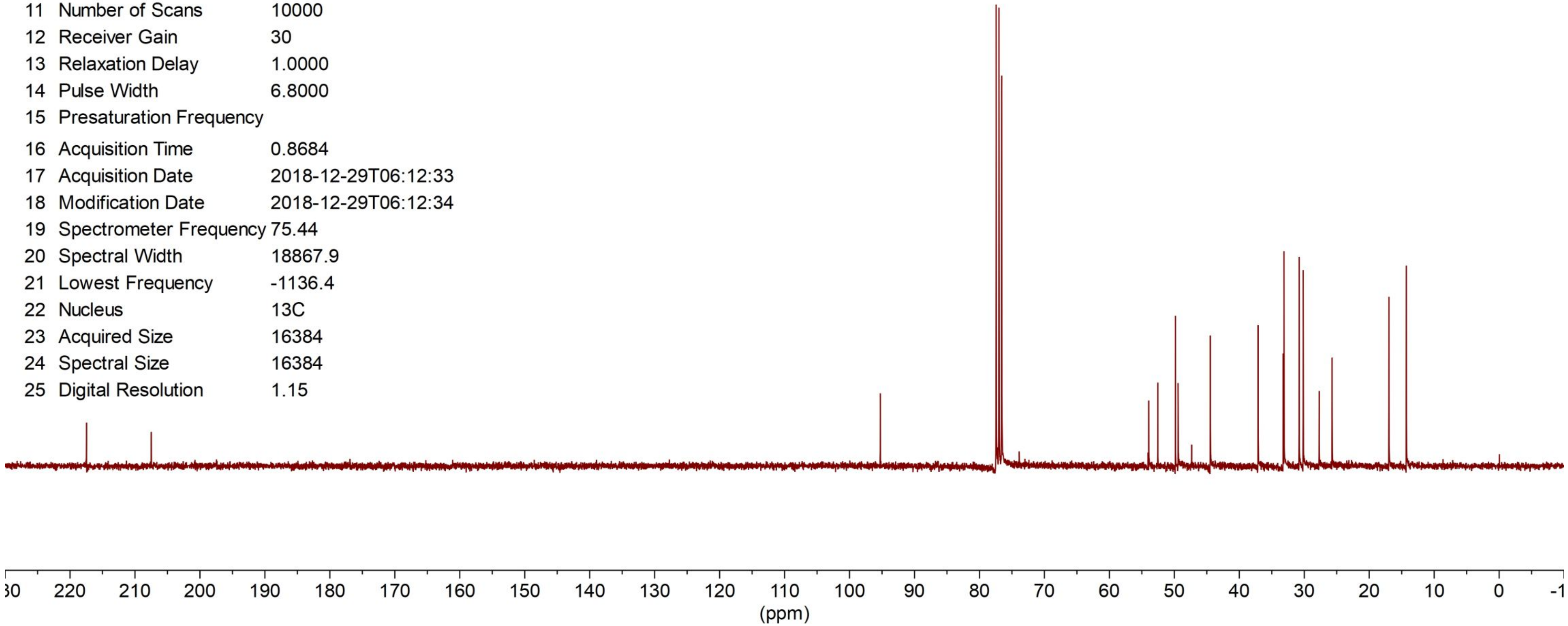

Parameters

	Parameter	Value
1	Title	CARBON_01
2	Comment	07BP-068-196-A
3	Origin	Varian
4	Instrument	mercury
5	Author	
6	Solvent	cdcl3
7	Temperature	25.0
8	Pulse Sequence	s2pul
9	Experiment	1D
10	Probe	Autosw
11	Number of Scans	10000
12	Receiver Gain	30
13	Relaxation Delay	1.0000
14	Pulse Width	6.8000
15	Presaturation Frequency	
16	Acquisition Time	0.8684
17	Acquisition Date	2018-12-29T06:12:33
18	Modification Date	2018-12-29T06:12:34
19	Spectrometer Frequency	75.44
20	Spectral Width	18867.9
21	Lowest Frequency	-1136.4
22	Nucleus	13C
23	Acquired Size	16384
24	Spectral Size	16384
25	Digital Resolution	1.15

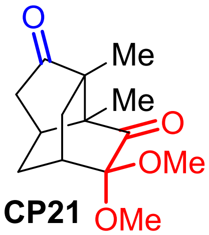

CP21
